# THAP1 is a maternal effect factor required for the first cell cycle via *Rrm1* in early mouse embryos

**DOI:** 10.1038/s44319-026-00712-9

**Published:** 2026-02-23

**Authors:** Qiang Fan, Xi Wu, Yanna Dang, Lijun Dong, Wenying Wang, Feng Kong, Lijuan Wang, Xukun Lu, Boyang Liu, Shuyan Ji, Wei Xie

**Affiliations:** 1 Center for Stem Cell Biology and Regenerative Medicine, MOE Key Laboratory of Bioinformatics, School of Life Sciences, New Cornerstone Science Laboratory, Tsinghua University, 100084 Beijing, China; 2 Tsinghua-Peking Center for Life Sciences, Tsinghua University, 100084 Beijing, China; 3 Peking University-Tsinghua University-National Institute of Biological Sciences (PTN) Joint Graduate Program, Academy for Advanced Interdisciplinary Studies, Peking University, 100871 Beijing, China; 4 National Research Center for Assisted Reproductive Technology and Reproductive Genetics, Shandong University, 250012 Jinan, Shandong China; 5 Institute of Medical Genetics and Development, Key Laboratory of Reproductive Genetics (Ministry of Education) and Women’s Hospital, Zhejiang University School of Medicine, 310006 Zhejiang, China

**Keywords:** MEG, THAP1, RRM1, dNTP, Cell Cycle, Chromatin, Transcription & Genomics, Development

## Abstract

Maternal effect genes (MEGs) produce factors that accumulate in oocytes and play critical roles in embryo development. Mutations of MEGs are frequently linked to reproductive and congenital disorders. The majority of identified mammalian MEGs encode epigenetic factors and RNA regulators. Here, we identify a MEG encoding the transcription factor Thanatos-associated protein 1 (*Thap1*). *Thap1* is highly expressed in mouse oocytes and early embryos. Oocyte-specific deletion of *Thap1* results in delayed progression of mouse embryos from the 1-cell to the 2-cell stage and 1-2-cell arrest, accompanied by defective zygotic genome activation (ZGA) and strongly impaired female fertility. Mechanistically, THAP1 activates a critical subset of genes in oocytes, including *Rrm1*, which produces ribonucleotide reductase required for generating deoxynucleotide triphosphates (dNTPs). Low-input metabolome profiling across 7 stages during the oocyte-to-embryo transition shows gradual, THAP1-dependent dNTP accumulation that peaks in MII oocytes. Overexpression of *Rrm1* in zygotes almost fully restores the 2-cell progression and ZGA in *Thap1* maternal-knockout embryos. Our findings identify THAP1 as a key maternal effector critical for the earliest stage of mammalian development.

## Synopsis

The maternal transcription factor THAP1 regulates the first cell cycle via *Rrm1* in early mouse embryos.

- The loss of maternal THAP1 arrests mouse embryos at the 1-2 cell stage.
- THAP1 is required for the expression of a small number of critical genes, including *Rrm1*, in oocytes.
- Insufficient RRM1 and the resulting reduction of dNDPs disrupt the first embryonic cell cycle.

## Abstract (web summary)

The maternal transcription factor THAP1 regulates the first cell cycle via *Rrm1* in early mouse embryos.

## Graphical abstract

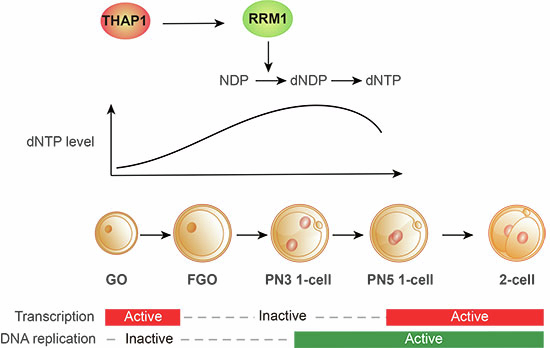

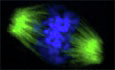

## Introduction

The growing oocyte accumulates large amounts of maternal proteins and RNAs during oogenesis to support a series of crucial events during the oocyte-to-embryo transition (OET), including meiotic resumption, maternal mRNA degradation, and ZGA, which enable the fully differentiated oocyte to transit into a totipotent embryo (Schultz et al, 2018). MEGs encode factors that are accumulated in oocytes, yet their deficiency impairs early development, causing embryonic defects or congenital disorders without necessarily affecting oocyte maturation (Innocenti et al, 2022; Kim and Lee, 2014; Mitchell, 2022). For instance, mouse embryos with defective maternal BTG4, a maternal mRNA decay licensor (Liu et al, 2016; Yu et al, 2016), or SCMC (subcortical maternal complex), an apparatus associated with oocyte protein storage (Jentoft et al, 2023; Li et al, 2008), manifest preimplantation development arrest. Mutations in human *BTG4* and SCMC also cause early embryonic developmental arrest (Lu et al, 2017; Zheng et al, 2020). To date, around 80 MEGs have been identified in mammals, among which the majority encode epigenetic factors and regulators of RNA metabolism (Mitchell, 2022). The defects of epigenetic regulators in oocytes can cause alterations of the epigenetic landscapes that are compatible with oocyte maturation. Such changes however can be inherited to early embryos and impair embryonic development. For example, the deficiency of maternal DNMT1 or DNMT3A causes loss of DNA methylation in oocytes, which does not affect oocyte maturation but leads to embryonic lethality after implantation due to loss of genomic imprints (Howell et al, 2001; Kaneda et al, 2004).

Interestingly, transcription factors (TFs) have rarely been identified as maternal effect factors. Deficiency in TFs expressed in oocytes often cause severe defects in folliculogenesis or oocyte maturation (usually when they are highly expressed) (e.g., *Nobox*, *Lhx8, Tcf3/12*) (Choi et al, 2008; Liu et al, 2024; Rajkovic et al, 2004). This is perhaps not surprising, as given the widespread targets of a TF, its mutation likely exerts profound impacts on oocyte development when it is fully functional. In other cases, maternal loss of TFs is compatible with embryonic development (usually when they are only lowly expressed in oocytes) (e.g., *Oct4*, *Tfap2c*, and *Nr5a2*) (Festuccia et al, 2024; Frum et al, 2013; Le Bin et al, 2014; Winger et al, 2006; Wu et al, 2013). *Cdx2* and *Oct4* were previously implicated as maternal effect factors (Jedrusik et al, 2010; Li et al, 2010; Zuccotti et al, 2008). However, their classification was argued given maternal KO of these genes did not affect female fertility (Blij et al, 2012; Frum et al, 2013; Le Bin et al, 2014; Mitchell, 2022; Wu et al, 2013). Recently, emerging evidence suggests that TFs can indeed function as MEGs. For example, maternal loss of PRDM10 (Seah et al, 2025) or TCF12 (Cao et al, 2025) caused defective mouse OET and early development.

THAP1, a member of the Thanatos-associated protein family, is a transcription factor characterized by the THAP domain, a protein motif similar to the P-element transposase (Roussigne et al, 2003b). The THAP domain is a C2CH zinc-dependent DNA binding domain that recognizes an 11-nucleotide sequence (AGTACGGGCAA) coined THABS (THAP1-binding sites) (Clouaire et al, 2005). Mutations in human *THAP1* cause Dystonia type 6 (DYT6), a neurologic movement disorder (Fuchs et al, 2009). Mice with deficient *Thap1* or pathogenic mutation C54Y (found in DYT6) in THAP1 display early embryonic lethality at embryonic day 10-14 (E10-14) stage (Ruiz et al, 2015). However, the role of THAP1 in the oocytes and early embryos remains to be elucidated.

In this study, we found that THAP1 is highly expressed in mouse oocytes and early embryos. By generating *Thap1* oocyte-specific knockout mice (*Thap1* Gdf9-cre conditional knockout female cross with wild-type (WT) male, named mKO hereafter), we discovered that *Thap1* mKO did not obviously affect oocyte development but resulted in 2-cell (2 C) arrest and defective ZGA, ultimately leading to severe female subfertility. Mechanically, depletion of *Thap1* led to reduced expression of a small set of genes in oocytes, including *Rrm1* which encoded a ribonucleotide reductase, resulting in reduced dNTPs. This is accompanied by delayed S phase exit in 1-cell (1 C) embryos with increased DNA damage, cell cycle check point activation, and delayed 1C-to-2C progression. Remarkably, overexpression of *Rrm1* or supplementation of its catalytic products, dNDPs, partially rescued the ZGA and developmental defects in *Thap1*-mKO embryos. These findings suggest that THAP1 is a maternal effect factor that plays a critical role in early embryonic development in part by regulating the first cell cycle of early embryo.

## Results

### Maternal *Thap1* knockout caused severe female infertility

**Figure 1:**
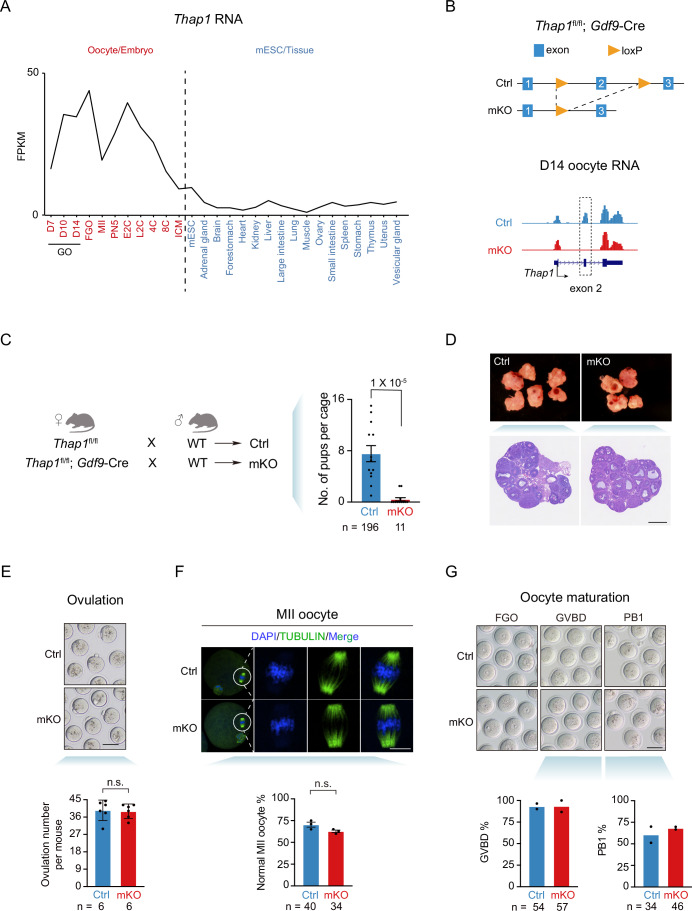
Maternal *Thap1* knockout leads to mouse infertility without causing apparent defects in oocyte maturation. (**A**) Line plot showing the gene expression of *Thap1* in mouse oocytes, embryos, mESCs, and somatic tissues. D7 day 7 after born, GO growing oocyte, FGO fully-grown oocyte, MII metaphase II oocyte, PN5 pronucleus stage 5, E2C early two-cell embryo, L2C late two-cell embryo, 4C/8C four-/eight-cell embryo, ICM inner cell mass. (**B**) Top: schematic of *Thap1* maternal KO (*Thap1*-mKO). Bottom: UCSC genome browsers showing the RNA-seq signals of *Thap1* in control and *Thap1* KO day 14 (D14) oocytes. A dashed box shows the exon 2 of *Thap1*. (**C**) Left: schematic of fertility test of control and *Thap1*-mKO mice. Right: bar charts showing the fertility test results. Dots represent the number of pups per cage in a month. *n*, the total number of pups. Error bars, standard error of the mean. *P* value, unpaired *t* test. (**D**) HE staining of control and *Thap1*-mKO ovaries (two biological replicates). Scale bar, 0.25 mm. (**E**) Bright-field images (top) and bar charts (bottom) showing oocyte morphology and the number of ovulated oocytes per mouse, respectively. Each dot represents a biological replicate. *n* the total number of mice. Error bars, standard error of the mean. *P* value, unpaired *t* test. Scale bar, 75 μm. (**F**) Top: immunofluorescence of TUBULIN in control and *Thap1*-mKO MII oocytes. Scale bar, 5 μm. Bottom: bar charts showing the percentages of MII oocytes with normal spindle in control and *Thap1*-mKO mice. Each dot represents a biological replicate. Error bars, standard error of the mean. *n* the total number of MII oocytes. *P* value, unpaired *t* test. (**G**) Bright-field images (top) and bar charts (bottom) showing oocyte morphology and maturation (GVBD and PB1) percentages upon maternal deletion of *Thap1*, respectively. Each dot represents a biological replicate. *n* the total number of GVBD or PB1 oocytes, GVBD germinal vesicle breakdown, PB1 the first polar body. Scale bar, 75 μm. *P* value, unpaired *t* test. Source data are available online for this figure.

To identify possible MEGs, we first investigated the transcriptomes in mouse oocytes and early embryos using a published dataset (Zhang et al, 2016). We noticed that *Thap1* was highly expressed in mouse oocytes and early embryos from the 1 C stage to the 4-cell (4 C) stage, before its expression declined in 8 C embryos and blastocysts (Fig. 1A, red). *Thap1* was lowly expressed in most adult tissues (Li et al, 2017) (Fig. 1A, blue; Dataset EV1). Analysis of the allelic RNA-seq data (Zhang et al, 2016) from mouse embryos showed that paternal *Thap1* was detected at the late 2C (L2C) stage, and the transcripts from the two alleles were equalized from the 8C stage (Appendix Fig. S1). These data suggest zygotic *Thap1* starts to be expressed from the L2C stage. We then asked whether THAP1 functions in mouse oocytes and early embryos. Considering that exon 2 of *Thap1* is part of the DNA-binding domain and a point mutation in this exon led to DYT6 disease in human (Fuchs et al, 2009), we deleted exon 2 of *Thap1* leading to a frameshift mutation through *Gdf9*-Cre (*Thap1*-mKO), which is expressed staring from primary follicles (Lan et al, 2004) (Fig. 1B, top; Table EV1). RNA-seq confirmed the absence of expression for exon 2 of *Thap1* in D14 (postnatal day 14) oocytes (Fig. 1B, bottom). Remarkably, *Thap1*-mKO female mice exhibited severe defects in fertility (0.4 pups per cage for *Thap1*-mKO mice compared to 7.5 pups per cage for control mice in a month) (Fig. 1C). We asked if such defects originated from abnormalities in oocytes. The ovaries of *Thap1-*mKO mice were morphologically normal with similar sizes compared to those of control (Fig. 1D). Depletion of *Thap1* did not affect ovulation (37.8 per *Thap1*-mKO mouse compared to 38.3 per mouse for control) (Fig. 1E), and *Thap1*-mKO oocytes underwent normal meiosis with correct spindle configuration (Fig. 1F,G). These data indicate that the deficiency of THAP1 does not apparently affect oocyte maturation but severely impairs female fertility.

### Maternal *Thap1* knockout caused defective DNA replication, increased DNA damage in 1C embryos, as well as 1-2C arrest

**Figure 2:**
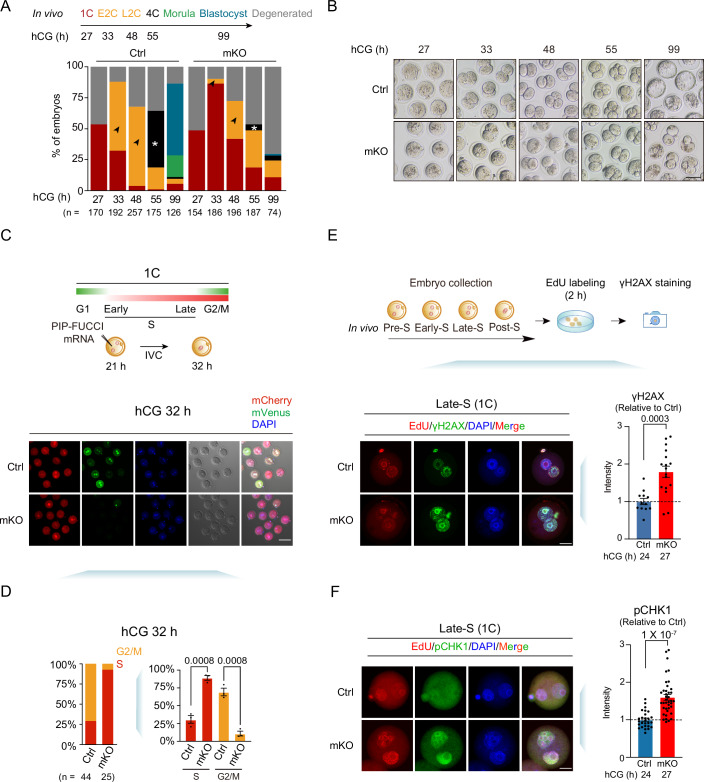
Maternal *Thap1* knockout leads to 1–2C arrest. (**A**) Developmental rates of control and maternal *Thap1* knockout embryos. Arrows indicate the percentages of 2-cell embryos. Asterisks indicate the percentages of 4-cell embryos (three biological replicates). *n*, the total number of embryos. (**B**) Embryo morphology of control and *Thap1*-mKO embryos dissected in vivo 27, 33, 48, 55, and 99 h post-hCG (three biological replicates). Scale bar, 75 μm. (**C**) Top: schematic of the PIP-FUCCI assay to mark G1-, S-, and G2/M-phase cells. Bottom, fluorescence of mCherry and mVenus of PIP-FUCCI assay in control and *Thap1*-mKO embryos at hCG 32 h. Bright field is also shown (three biological replicates). Scale bar, 100 μm. (**D**) Left: the percentages of mCherry and mVenus of PIP-FUCCI assay in control and *Thap1*-mKO zygotes at 32 h post-hCG. *n*, the total number of embryos. *P* value, unpaired *t* test. Right: bar charts showing the percentages of G2/M- and S-phase embryos in control and *Thap1*-mKO zygotes at 32 h post-hCG. Each dot represents a biological replicate. Error bars, standard error of the mean. (**E**) Top: a model illustrating EdU incorporation and γH2AX staining. Bottom (left): immunofluorescence of EdU and γH2AX in control Late-S embryos (hCG 24 h) and *Thap1*-mKO Late-S embryos (hCG 27 h) (three biological replicates). Scale bar, 20 μm. Bottom (right), bar charts showing the relative intensities of γH2AX in control embryos and *Thap1*-mKO embryos at the Late-S stage. The intensities of nuclear signals are normalized to those in cytoplasm; the ratio for each embryo is further normalized to the average values of control embryos. Each dot represents a single embryo. Error bars, standard error of the mean. *P* value, unpaired *t* test. (**F**) Left: immunofluorescence of pCHK1 and EdU in control Late-S embryos (hCG 24 h) and *Thap1*-mKO Late-S embryos (hCG 27 h) (two biological replicates). Scale bar, 75 μm. Right: bar charts show the relative intensities of pCHK1 in control and *Thap1*-mKO embryos at the Late-S stage (two biological replicates). The intensities of nuclear signals are normalized to those in cytoplasm; the ratio for each embryo is further normalized to the average values of control embryos. n.s. not significant. Each dot represents a single embryo. Error bars, standard error of the mean. *P* value, unpaired *t* test. Source data are available online for this figure.

We next asked if *Thap1* maternal knockout affected early development by isolating early embryos in vivo at various stages. To identify the stage when developmental defects commenced, embryos were collected at 27 h, 33 h, 48 h, 55 h, and 99 h post-hCG, corresponding to 1C, early two-cell (E2C), L2C, 4C, and blastocyst stages for control embryos, respectively (Fig. 2A,B). At the E2C stage (33 h post-hCG), while 56% control embryos developed to 2C, only 11% *Thap1*-mKO embryos developed to this stage. When 64% control embryos already reached the 2C stage at 48 h post-hCG, 42% of the *Thap1*-mKO embryos still retained the 1C morphology (Fig. 2A,B). Finally, when the majority of control embryos (57.9%) developed to blastocysts, most *Thap1*-mKO embryos were still arrested at the 1–2C stage, with only 1.3% reaching the blastocyst stage (Fig. 2A,B, hCG 99 h). These data indicate that most *Thap1*-mKO embryos are arrested at the 1–2C stages.

The defects in the 1–2C transition in *Thap1*-mKO embryos prompted us to investigate if the first cell cycle was affected. To monitor the cell cycle progression, the mRNA of PIP-mVenus-P2A-mCherry-Gem1-110 (PIP-FUCCI) (Grant et al, 2018) was introduced into the pronuclear stage 3 (PN3) mouse zygotes when DNA replication was initiated (Ferreira and Carmo-Fonseca, 1997). mVenus, mCherry, and mCherry/mVenus double-positive signals marked cells at G1 phase, S phase, and the G2/M phase, respectively (Fig. 2C, green, mVenus; red, mCherry). Indeed, while the majority of control embryos (71%) had already progressed to the G2/M phase, most *Thap1*-mKO embryos (92%) were still in the S phase (Fig. 2C,D). To further investigate the possible defects in DNA replication in *Thap1*-mKO embryos, we performed EdU staining during the 1C to 2C embryo progression. We determined the replication stages of embryos based on their characteristic EdU staining patterns previously reported (Bouniol et al, 1995) (Appendix Fig. S2A, “EdU”, red): “Pre-S” phase was defined by the absence of EdU signals in both the nucleus and the second polar body; “Early-S” phase was characterized by diffuse EdU signals throughout the nucleoplasm of both pronuclei; “Late-S” phase was identified by a characteristic peri-nucleolar EdU fluorescent ring formed in the pronuclei; “Post-S” phase was marked by lack of EdU signals in the nucleus but the persistence of EdU staining in the second polar body. We then examined embryos collected in vivo at 21, 24, and 27 h post-hCG when most control embryos developed to Early-S, Late-S, and Post-S phase, respectively (Appendix Fig. S2B–D). EdU incorporation was largely comparable between control and *Thap1*-mKO embryos at 21 h (Appendix Fig. S2B, “EdU”, red). At 24 h, the overall intensity of EdU was also comparable (Appendix Fig. S2C, “EdU”, red). However, while almost half (49%) of control zygotes entered the Late-S phase (indicated by the appearance of the peri-nucleolar fluorescent ring), the majority (67%) of *Thap1*-mKO embryos were still at the Early-S phase (indicated by the diffuse EdU fluorescent foci) (Appendix Fig. S2C, middle). At 27 h, while 56% of control zygotes already completed DNA replication (indicated by no EdU incorporation in the nucleus) and entered the Post-S phase, *Thap1*-mKO embryos still exhibited strong EdU signals (Appendix Fig. S2D, “EdU”, red) with the majority (59%) of embryos at the Late-S phase (Appendix Fig. S2D, middle), suggesting a delay in DNA replication exit. Therefore, the *Thap1*-mKO embryos exhibited defective cell cycle progression in 1C embryos, followed by delayed 1C-to-2C transition.

We further asked if such defective DNA replication exit upon loss of THAP1 may be associated with DNA damage. The control zygotes displayed elevated γH2A.X levels at 21–24 h which decreased at 27 h (Appendix Fig. S2A, “γH2A.X”, green), consistent with previous studies that DNA damage increased in the S phase but decreased after the S phase exit (Wossidlo et al, 2010; Xu et al, 2015). By contrast, *Thap1*-mKO embryos exhibited persistently higher γH2A.X levels from 21 to 27 h compared to controls (Appendix Fig. S2B–D, “γH2A.X”, green). Considering the developmental delay of *Thap1*-mKO embryos (for example, the majority of mKO embryos entered the Late-S phase at 27 h, 3 h later than control embryos), we identified embryos at an equivalent replication stage (Late-S) based on EdU staining. This analysis still revealed significantly higher γH2AX signals in *Thap1*-mKO embryos compared to control embryos (Fig. 2E). Consistently, the signals of phosphorylated Checkpoint kinase 1 (pCHK1) (Jeong et al, 2003) increased significantly in *Thap1*-mKO Late-S embryos (Fig. 2F), suggesting activation of cell cycle checkpoint (Min et al, 2013; Smits and Gillespie, 2015). Thus, these data suggest that maternal THAP1 depletion leads to increased DNA damage, activation of cell cycle checkpoints, and delayed DNA replication exit.

### Proper DNA replication is required for the integrity of ZGA

**Figure 3:**
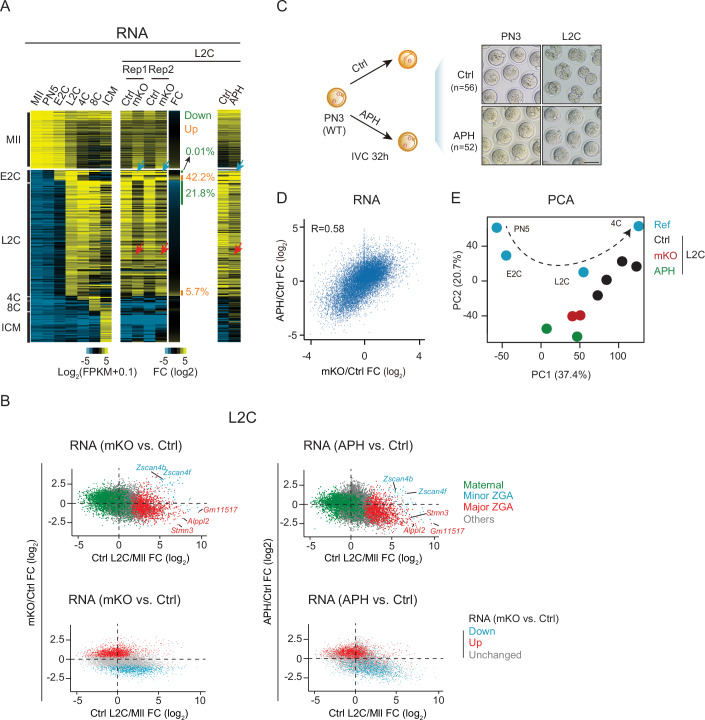
Comparison between transcriptomes of APH-treated embryos and *Thap1*-mKO embryos. (**A**) Heatmaps showing the stage-specific gene expression in control, *Thap1*-mKO embryos, and embryos treated with or without aphidicolin (APH). Blue and red arrows indicate the E2C-specific and L2C-specific genes, respectively. (**B**) Scatter plots showing gene expression fold-changes upon maternal deletion of *Thap1* (left) and APH treatment (right) at the L2C stage (*y* axis) compared to gene expression changes from control MII oocytes to control L2C embryos (*x* axis) (two biological replicates). FC fold change. Top: maternal genes, major zygotic genome activation (ZGA) genes, and minor ZGA genes are color-coded. Bottom: differentially expressed genes (DEGs) upon maternal deletion of *Thap1* are color-coded. (**C**) Schematic of treating the PN3 zygotes with or without APH for 32 h followed by RNA-seq. IVC, in vitro culture. Embryo morphology of control and APH-treated embryos at the PN3 and L2C stages (two biological replicates) are shown. *n*, the total number of embryos. Scale bar, 75 μm. (**D**) Scatter plots showing gene expression fold-changes upon maternal deletion of *Thap1* (left) and APH treatment (right) at the L2C stage. FC fold change. Maternal genes, major zygotic genome activation (ZGA) genes, and minor ZGA genes are color-coded (two biological replicates). (**E**) PCA analysis of the transcriptome data of control (black), mKO (red), APH-treated (green) embryos. Reference RNA-seq data (WT, blue) from PN5 to 4C embryos (Zhang et al, 2016) were used to show the developmental trajectory. Source data are available online for this figure.

As ZGA occurs during the 1-2 C stage, we then investigated the effect of THAP1 deficiency on ZGA. From the mid-1-cell to E2C stage, a small subset of genes is activated, known as minor ZGA. This is followed by major ZGA at the L2C stage, during which thousands of genes are activated (Aoki et al, 1997; Bouniol et al, 1995; Schulz and Harrison, 2019). At L2C, *Thap1*-mKO embryos exhibited downregulation of 242 major ZGA genes (out of 1106, 21.8%) (fold change >2; *P* value < 0.05, Wald test, Method) (Fig. 3A, red arrows and 3B, top left, red; Dataset EV2). Additionally, substantial up-regulation of minor ZGA genes (27 out of 64, 42.2%) (Fig. 3A, blue arrows and 3B, top left, blue; Dataset EV2) was also observed, which may reflect the delayed downregulation of these genes as their transcription typically declined rapidly after the 2C stage. Given that *Thap1*-mKO embryos exhibited defective cell cycle progression as early as the S phase at the 1C stage (Fig. 2C,D), we asked if dysregulated DNA replication and cell cycle in 1C embryos can impair ZGA. Therefore, we treated the control embryos with aphidicolin (APH), an inhibitor of DNA replication, from the PN3 stage, followed by in vitro culture. After 32 h treatment when control embryos developed to the L2C stage, APH-treated embryos were still arrested at the 1C stage (Fig. 3C). RNA-seq analyses showed that despite both minor and major ZGA being initiated after APH treatment as reported (Hamatani et al, 2004), the global transcriptome was altered (Fig. 3A). Specifically, major ZGA genes tended to be downregulated (490 out of 1106, 44.3%) (Fig. 3A, right, red arrow, and 3B, top right, red; Dataset EV3), while part of minor ZGA genes were upregulated (9 out of 65, 13.8%) (Fig. 3A, right, blue arrow, and 3B, top right, blue; Dataset EV3). These data are in line with the notion that proper DNA replication is required for the integrity of ZGA (Aoki et al, 1997; Davis et al, 1996; Davis and Schultz, 1997; Forlani et al, 1998). These defects resembled those in the *Thap1*-mKO 2C embryos. Indeed, the transcriptional changes in *Thap1*-mKO embryos well correlated with those in the APH-treated embryos (*R* = 0.58) (Fig. 3B, bottom and  3D,E). These data indicate that proper DNA replication is required for the integrity of ZGA, supporting the possibility that the ZGA defects in *Thap1*-mKO embryos were partially caused by the compromised DNA replication.

### THAP1 regulates transcription in growing oocytes

**Figure 4:**
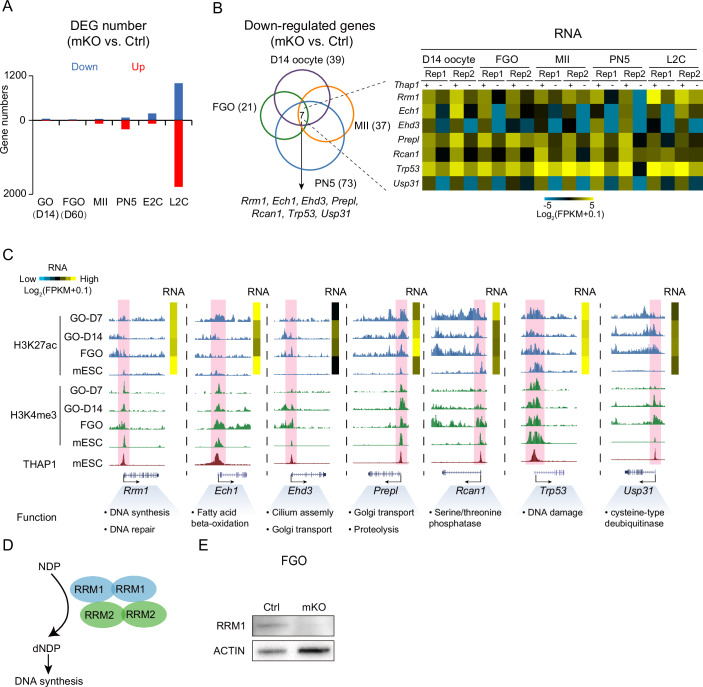
THAP1 regulates *Rrm1* expression in oocyte. (**A**) Bar charts showing the numbers of down- and upregulated genes in *Thap1*-mKO embryos compared with control embryos. (**B**) Left: Venn diagram showing the overlap of downregulated genes in D14 oocytes, FGOs, MII oocytes, PN5 zygotes, and L2C embryos upon maternal deletion of *Thap1*. Gene numbers are shown. Right: heatmaps show the gene expression of the downregulated genes in control and *Thap1*-mKO embryos. *Thap1* (+/−), control or *Thap1*-mKO. (**C**) UCSC genome browsers showing H3K27ac, H3K4me3 signals in D7 oocytes, D14 oocytes, FGOs, and mESCs, and THAP1 binding in mESCs at the promoters of the downregulated genes. The expression and brief function of each gene are also shown. (**D**) Schematic illustration showing that RRM1 catalyzes the conversion of NDPs to dNDPs through binding to RRM2. (**E**) Western blot showing protein levels of RRM1 in control and *Thap1*-mKO FGOs (*n* = 150 oocytes). ACTIN is shown as control. Source data are available online for this figure.

Given the essential role of THAP1 in early development, we then sought to pinpoint its exact functional stage. The fact that THAP1 deficiency causes DNA replication defects in 1C embryos prior to major transcription activation in 2C mouse embryos (Aoki et al, 1997; Schulz and Harrison, 2019) suggests that part of these defects may originate from oocytes. Indeed, reintroducing *Thap1* mRNA into *Thap1*-mKO zygotes could not rescue the 1–2C developmental arrest (Appendix Fig. S3A–D) or the ZGA defects (Appendix Fig. S3E). A similar observation was made when expressing *Thap1* mRNA in *Thap1*-mKO FGOs or MII oocytes, as the developmental defects could not be rescued by reintroducing *Thap1* mRNA at these stages (Appendix Fig. S3F–I). These data are perhaps not surprising given that transcription is silenced in late-stage FGOs, MII oocytes, and 1C embryos (Bouniol-Baly et al, 1999; Schultz et al, 2018), suggesting that THAP1 may function at an earlier developmental stage. We then investigated whether *Thap1* deficiency leads to aberrant transcription in oocytes, ultimately impairing embryo development. Transcriptome analyses of WT oocytes at various stages, including growing oocytes at postnatal day 7 (D7), D10, D14, and FGOs showed that the *Thap1* mRNA level increased during oocyte growth (Fig. 1A; Dataset EV1). Surprisingly, RNA-seq analysis revealed that only 39, 21, 37, and 73 genes were downregulated after deletion of *Thap1* in D14 oocytes, FGOs, MII oocytes, and the PN5 zygotes, respectively (Fig. 4A and Appendix Fig. S4; Dataset EV4). Even smaller numbers of genes were upregulated in D14 oocytes (*n* = 11) and FGOs (*n* = 10). The number of upregulated genes increased in PN5 zygotes (*n* = 240), although the majority (74%) corresponded to genes with maternal transcripts, suggesting delayed clearance of oocyte RNAs considering transcription activities were still very low at this stage (Abe et al, 2015; Jukam et al, 2017; Lee et al, 2014). We also performed functional enrichment analysis on differentially expressed genes throughout the stages (Appendix Fig. S4). In D14 oocytes, downregulated genes were preferentially involved in proteolysis, while the upregulated genes were not enriched for any GO terms. No enriched terms were found for FGO and MII oocytes. In PN5 embryos, downregulated genes were enriched for those involved in transcription, apoptosis, and chromatin remodeling, while the upregulated genes were associated with translation and mitochondrial organization. In L2C embryos, downregulated genes were preferentially involved in rRNA processing, translation, DNA repair, and cell division, while the upregulated genes were associated with transcription, cell differentiation, and cell migration. We then focused on the downregulated genes as THAP1 was reported to primarily function in transcription activation (Cayrol et al, 2007). Among them, only seven genes (*Rrm1*, *Ech1*, *Ehd3*, *Prepl*, *Rcan1*, *Trp53*, and *Usp31*) were consistently downregulated across all stages examined (Fig. 4B, left). The promoters of these genes carried active marks (H3K27ac (Liu et al, 2024) and H3K4me3 (Zhang et al, 2016)) in oocytes, confirming their active transcription state (Fig. 4C). Re-analysis of THAP1 ChIP-seq (Aguilo et al, 2017) in mESCs showed that THAP1 bound to the promoters of these genes (Fig. 4C). Although genes regulated by THAP1 in oocyte are limited, these genes play critical roles in DNA synthesis, DNA repair, Golgi transportation, and fatty acid oxidation etc. (Fig. 4C, bottom). Such transcription defects persisted in early embryos as the expression of these genes was still downregulated in 1C and L2C embryos (Fig. 4B, right). In summary, these results suggest THAP1 regulates the transcription of a small yet critical subset of genes in oocytes.

### RRM1 partially rescues developmental defects of *Thap1*-mKO embryos

**Figure 5:**
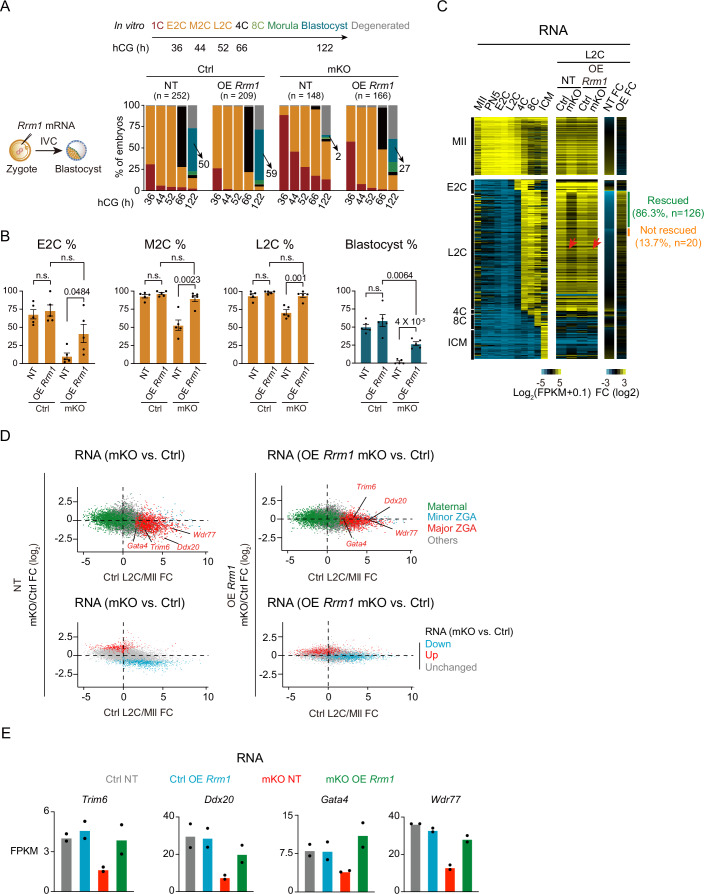
Overexpression of *Rrm1* could partially rescue the defects caused by THAP1 loss. (**A**) Left: schematic of *Rrm1* (100 ng/μl) rescue in *Thap1*-mKO zygotes followed by in vitro culture. Right: developmental rates of control and *Thap1*-mKO embryos with or without the overexpression of *Rrm1*. Arrows indicate the percentage of blastocyst. M2C mid two-cell embryo. NT no treatment (three biological replicates). *n*, the total number of embryos. (**B**) Bar charts showing the percentage of E2C, M2C, L2C, and blastocyst in control and *Thap1*-mKO embryos with or without the overexpression of *Rrm1*. Each dot represents a biological replicate. *N* = five biological replicates. Error bars, standard error of the mean. n.s. not significant; *P* value, unpaired *t* test. (**C**) Heatmaps showing the stage-specific gene expression in control and *Thap1*-mKO embryos with or without the overexpression of *Rrm1* (two biological replicates). Arrows, major ZGA genes. (**D**) Scatter plots showing gene expression fold-changes upon maternal deletion of *Thap1* (left) and *Rrm1* rescue (right) at the L2C stage (*y* axis) compared to gene expression changes from control MII oocytes to control L2C embryos (*x* axis) (two biological replicates). FC fold change. Top: maternal genes, major zygotic genome activation (ZGA) genes, and minor ZGA genes are color-coded. Bottom: differentially expressed genes (DEGs) upon maternal deletion of *Thap1* at the L2C stage are color-coded. NT, no treatment. (**E**) Bar charts showing the expression of various ZGA genes in control and *Thap1*-mKO L2C embryos with or without the overexpression of *Rrm1*. Each dot represents a biological replicate. Source data are available online for this figure.

Notably, among all the downregulated genes in oocytes, RRM1, a subunit of ribonucleotide reductase (RNR) that catalyzes the biosynthesis of dNDPs from corresponding NDPs together with RRM2 (a smaller subunit) (Fig. 4D), is indispensable for DNA replication and repair particularly in the S phase (Bjorklund et al, 1993; Thelander and Reichard, 1979). *Rrm1*’s expression was detected as early as in growing oocytes and persisted in early embryos, being further upregulated after major ZGA (Appendix Fig. S5A; Dataset EV1). Therefore, we investigated whether loss of RRM1 contributed to the cell cycle defects and developmental arrest of *Thap1*-mKO embryos. We first confirmed the downregulation of RRM1 protein in *Thap1-*mKO oocytes (Fig. 4E). Remarkably, when *Rrm1* mRNA was introduced into *Thap1*-mKO zygotes (Appendix Fig. S5B), the developmental defects from 1 C to 2C were successfully rescued (Fig. 5A,B; Appendix Fig. S5C). At the E2C stage (36 h post-hCG), the percentage of *Thap1*-mKO embryos that developed to the 2C embryos increased from 10% to 42% after restoring *Rrm1* mRNA, compared to 68% of non-treated (NT) WT controls (Fig. 5A,B; Appendix Fig. S5C). The percentage further increased from 53% to 89% at the M2C stage (44 h post-hCG) and from 71% to 94% at the L2C stage (52 h post-hCG) (Fig. 5A,B; Appendix Fig. S5C), closely matching 93% and 94% of WT controls, respectively, indicating the 1–2C delay of *Thap1*-mKO embryos was fully rescued by the overexpression of *Rrm1*. Moreover, ZGA defects upon depletion of maternal *Thap1* were also largely rescued by overexpression of *Rrm1* (Fig. 5C–E; Appendix Fig. S5D; Dataset EV5), with 86.3% (126 out of 146) of the downregulated major genes upon THAP1 depletion partially recovered (Fig. 5C). The rates of *Thap1*-mKO embryos developing to 4C at 66 h and blastocyst at 122 h post-hCG were also markedly increased upon *Rrm1* overexpression (from 4% to 49% for 4C and from 2% to 27% for blastocyst, Fig. 5A,B; Appendix Fig. S5C), although these rates were still lower than the NT controls (71% and 51%, respectively). The residue W684 of RRM1 was reported to be essential for the binding of RRM1 to RRM2 to form a complete RNR complex (Fig. 4D) (Specks et al, 2015). As a control, RRM1 with a mutation in W684 (RRM1^W684G^) failed to rescue the development of *Thap1*-mKO embryos (Appendix Fig. S6A–C). Thus, the defective expression of *Rrm1* is one of the key factors contributing to the 1–2C developmental arrest and ZGA defects in *Thap1*-mKO embryos.

### dNDPs partially rescue the defects of *Thap1*-mKO embryos

**Figure 6:**
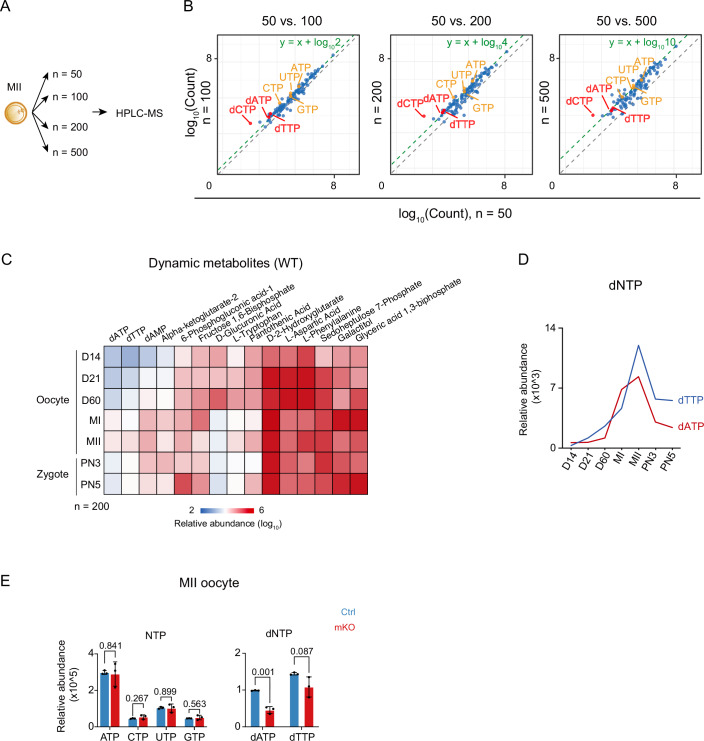
Reduced abundance of dNTPs in *Thap1*-mKO MII oocytes. (**A**) Schematic of HPLC-MS with various numbers of MII oocytes. *n*, MII oocyte number. (**B**) Scatter plots comparing log_10_ transformed metabolites’ abundance of 100, 200, and 500 MII oocytes against 50 MII oocytes. Green dashed lines representing the log_10_ metabolites’ abundance with expected fold-changes comparing with that of 50 MII oocytes. The reference lines (*y*  =  x) are shown in grey. NTP and dNTP are color-coded. *n*, number of MII oocytes. (**C**) Heatmaps showing the dynamic metabolites’ abundance in WT mouse oocytes and zygotes. *n*, number of MII oocytes or embryos. (**D**) Line plots showing the abundance of dNTPs in mouse oocytes and embryos. (**E**) Bar charts showing the relative abundance of dNTPs (top) and NTPs (bottom) in control and *Thap1*-mKO MII oocytes. Each dot represents a biological replicate. *N* = three biological replicates, 100 embryos for each replicate. Error bars, standard error of the mean. *P* value, unpaired *t* test. Source data are available online for this figure.

Considering RRM1 functions in converting NDPs to dNDPs, which allows the production of dNTPs required for DNA replication, we asked if *Thap1*-mKO caused insufficient dNTPs in embryos. Therefore, we measured the levels of metabolites in mouse oocytes using an optimized, low-input label-free HPLC-MS approach (Kulak et al, 2014). To test its quantification capability, we first measured the metabolite abundance in MII oocytes with varying cell numbers (Fig. 6A,B). A total of 113, 114, 115, and 116 metabolites were identified from 50, 100, 200, and 500 MII oocytes, respectively, suggesting the detection of metabolites was robust in this input cell range. Notably, the abundance of a subset of metabolites (*n* = 79, Dataset EV6) showed excellent linearity with cell numbers (“quantifiable metabolites”) (Fig. 6A,B, “Methods”). For example, compared to metabolites from 50 MII oocytes, those from 100, 200, and 500 MII oocytes increased correspondingly by roughly two-, four-, and tenfold, respectively (Fig. 6B). These metabolites included NTPs, dATP, and dTTP. The concentration of dGTP was not measured because the HPLC-MS was unable to distinguish its signal from that of its isomer ATP, which is typically present at much higher concentrations in cells (Traut, 1994). We then focused on these 79 metabolites and examined their dynamics across 7 stages during normal mouse oocyte growth and OET (from D14 growing oocytes to PN5 embryos), using 200 oocytes or embryos at each stage. Among these metabolites, a subset exhibited substantial changes in this process (Fig. 6C; Dataset EV7). The levels of dNTPs (dATP and dTTP) increased during oocyte growth and peaked at the MII oocyte stage (Fig. 6D; Appendix Fig. S7A,B), indicating accumulation of dNTPs presumably for the coming DNA replication after fertilization. The levels of dNTPs decreased in PN3 and PN5 zygotes (Fig. 6D), likely due to their consumption upon DNA replication. Importantly, the abundance of dNTPs was significantly reduced in *Thap1*-mKO MII oocytes compared to control (Fig. 6E, left; Dataset EV8). As a control, no significant changes were observed for NTPs (Fig. 6E, right; Dataset EV8), indicating loss of *Thap1* impairs dNTP production.

**Figure 7:**
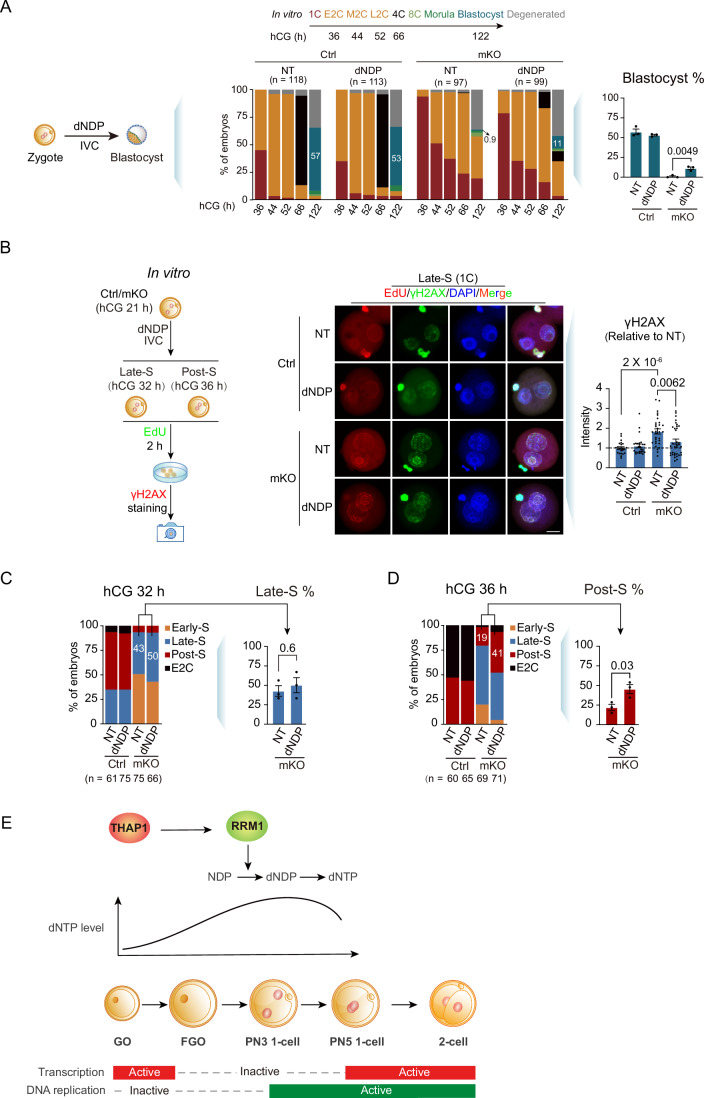
dNDPs could partially rescue the defects of *Thap1*-mKO embryos. (**A**) Left: schematic of culturing embryos in medium supplied with four dNDPs (125 μM each). Middle, developmental rates of control and *Thap1*-mKO embryos cultured in medium supplied with or without dNDPs. Percentages of blastocyst embryos are labeled. Right, bar charts show the percentages of blastocyst embryos in control and *Thap1*-mKO embryos cultured in a medium supplied with or without dNDPs. *n*, the total number of embryos. Each dot represents a biological replicate. *N* = three biological replicates. Error bars, standard error of the mean. *P* value, unpaired *t* test. (**B**) Left: schematic of culturing embryos in medium supplied with four dNDPs (125 μM each). Middle, immunofluorescence of EdU and γH2AX in control and *Thap1*-mKO embryos cultured in medium supplied with or without dNDPs at the Late-S stage. Right: bar charts show the relative intensities of γH2AX in control and *Thap1*-mKO embryos cultured in a medium supplied with or without dNDPs at the Late-S stage. The intensities of nuclear signals are normalized to those in the cytoplasm; the ratio for each embryo is further normalized to the average values of NT embryos. NT no treatment. Each dot represents a single embryo. *N* = three biological replicates. In total, 24 and 25 embryos for NT and dNDP in control group, 31 and 35 embryos for NT and dNDP in mKO group. Error bars, standard error of the mean. *P* value, unpaired *t* test. Scale bar, 20 μm. (**C**, **D**) Left: developmental rates of control and *Thap1*-mKO embryos cultured in medium supplied with or without dNDPs at hCG 32 h (left) and 36 h (right), respectively. *n*, the total number of embryos. Right: bar charts show the percentages of Late-S and Post-S embryos in *Thap1*-mKO embryos cultured in a medium supplied with or without dNDPs at hCG 32 h (left) and 36 h (right), respectively. Each dot represents a biological replicate. Error bars, standard error of the mean. *P* value, unpaired *t* test. (**E**) A model illustrating that THAP1 activates the transcription of *Rrm1* in growing oocytes, which would catalyze NDPs to dNDPs during the maturation of oocytes to fulfill the dNTP demands of zygotes after fertilization. Source data are available online for this figure.

We next investigated whether supplementing zygotes with dNDPs, the catalytic products of RRM1, could alleviate the defects caused by THAP1 deficiency. When the four dNDPs (125 µM each) were added to the in vitro culture medium, the percentage of *Thap1*-mKO embryos that developed to the blastocyst embryos increased from 0.9 to 11% (Fig. 7A; Appendix Fig. S8). Meanwhile, the elevated γH2AX signals in *Thap1*-mKO Late-S 1 C embryo were reduced upon dNDP supplement (Fig. 7B), suggesting partial alleviation of DNA damage in *Thap1*-mKO embryos. The development of *Thap1*-mKO embryos to the Late-S stage was slightly improved at 32 h (from 43 to 50%) upon the addition of dNDPs (Fig. 7C, right). The development improvement became apparent at 36 h, with 41% *Thap1*-mKO embryos having completed DNA replication, compared to 19% without dNDP addition (Fig. 7D, right). The milder rescue by dNDPs compared to that by *Rrm1* overexpression was perhaps not surprising, given that it is challenging to achieve the optimal concentrations (higher concentrations affected normal development in control embryos) and ratios of dNDPs comparable to their endogenous levels in embryos. In sum, these data support the possibility that loss of *Thap1* causes downregulation of *Rrm1* and dNTPs in oocytes, which further leads to defective cell cycle in early embryos.

### THAP1 is specifically required for the expression of *Rrm1* in mouse oocytes

Homozygous *Thap1* null mice display lethality at the E10-14 stage (Ruiz et al, 2015). The fact that early embryos can survive without zygotic *Thap1* prior to E10 indicates that either the maternal THAP1 protein supports early embryos up to E10, or other TFs may help activate *Rrm1* in early embryos until the accumulated defects cause lethality after E10. Notably, *Thap1*-deficient mESCs grew slower but remained viable (Aguilo et al, 2017). While the expression of *Rrm1* strongly depended on THAP1 in FGOs, re-analysis of the previous datasets (Aguilo et al, 2017; Shinoda et al, 2021) revealed *Rrm1* expression was unaffected in *Thap1* KO mESCs and MEFs (Appendix Fig. S9). Taken together, these data indicate that THAP1 is specifically required for the expression of *Rrm1* in oocytes, while other transcription factors may regulate *Rrm1* at later stages of embryos, which warrants future studies.

## Discussion

The genome of the mammalian zygote is initially inactive and early developmental processes are regulated by maternal factors that are present in the egg before fertilization. In this study, we found that *Thap1* is highly expressed throughout OET, and loss of maternal THAP1 causes DNA replication defects in 1 C embryos, 1-2 C transition delay, and ultimately 2 C arrest with defective ZGA, highlighting a critical role of maternal THAP1 in early development.

Previously, it was found that THAP1 can directly activate *Rrm1* in HUVECs and regulate cell cycle progression (Cayrol et al, 2007). However, its physiological significance remains elusive. In this study, we found loss of THAP1 and the downregulation of RRM1 led to insufficient supplement of dNTPs for the DNA synthesis in the first cell cycle after fertilization (Fig. 7E). Naturally, deficiency of *Thap1* did not apparently affect the growth of oocytes, which do not require DNA replication. However, a 37% reduction of dNTPs in *Thap1*-mKO MII oocytes is sufficient to cause severe phenotype in early embryos, suggesting that dNTPs in the nucleus of early mouse embryos are highly limited, and their production is tightly regulated. These results echo the finding in *Drosophila* early embryos, where maternal stocks of dNTPs provide less than half of the amount required to reach gastrulation, with the remaining dNTPs being synthesized de novo (Song et al, 2017). In fact, both high and low levels of dNTPs are toxic to cells and inhibit cell cycle progression (Chabes and Stillman, 2007; Pai and Kearsey, 2017). Interestingly, the ZGA defects were also almost fully rescued by *Rrm1* overexpression, suggesting intact DNA replication is required for the fidelity of ZGA, even though ZGA can still initiate in the absence of DNA replication and cell division (Hamatani et al, 2004). The exact mechanisms by which DNA replication regulates ZGA remain elusive and warrant further investigations.

Finally, while *Rrm1* rescued the 2 C development and ZGA defects in *Thap1*-mKO embryos, the blastocyst development rate was not fully recovered. This is perhaps not surprising, given that THAP1 also regulates other genes in oocytes and is involved in essential cellular processes including apoptosis (Roussigne et al, 2003a) and DNA repair (Shinoda et al, 2021). Further studies are warranted to elucidate the functions of other THAP1 targets in oocytes and early embryos. Taken together, our study identified an essential role of THAP1, a factor highly expressed during OET, in regulating the first DNA replication in the embryos. Moreover, these data present a unique example of how a maternal TF can function as a maternal effect factor by regulating a small but crucial subset of genes in oocytes that in turn play critical roles for early mammalian development.

## Methods

**Table:** Reagents and tools table

Reagent/resource	Reference or source	Identifier or catalog number
**Experimental models**		
C57BL/6 (*M. musculus)*	Tsinghua Animal Center	000664
**Recombinant DNA**		
PIP-FUCCI	Addgene	118621
**Antibodies**		
RRM1	Proteintech	10526-1-AP
TUBULIN-FITC	Sigma	F2168
γH2AX	Cell Signaling Technology	9718S
pCHK1	Cell Signaling Technology	2348 T
ACTIN	Yeasen	30101ES50
Donkey anti-Rabbit 594	Thermo Fisher Scientific	A-21207
Donkey anti-Mouse 488	Jackson ImmunoResearch Laboratories	715-545-150
**Oligonucleotides and other sequence-based reagents**		
Genotyping primers	This study	Table EV1
sgRNA	This study	Table EV1
**Chemicals, enzymes and other reagents**		
Pregnant mare serum gonadotropin	Ningbo Second Hormone Factory	110254564
Human chorionic gonadotrophin	Ningbo Second Hormone Factory	110251283
M2 medium	Sigma-Aldrich	M7167
KSOM medium	Milipore	Mr121d
TrypLE	Invitrogene	12605010
Tyrode’s buffer	Sigma	T1788
Cytochalasin B	Sigma	C6762
T3 mMESSAGE Kit	Invitrogen	AM1348
RNA Clean XP beads	Beckman	A63987
RIPA Lysis Buffer	Beyotime	P0013B
Protease Inhibitor Cocktail	Roche	4693132001
Microporous polyvinylidene fluoride membranes	Millipore	IPVH00010
SuperSignal West Femto	Thermo Scientific Pierce	34076
Phosphate buffered solution	Corning	21-040-CV
ProLong Diamond	Thermo Fisher	P36965
Paraformaldehyde	Sigma	P6148
Triton X-100	Ameresco	0694-1 L
Hochest	Sigma	B2261
VaClick 594-EdU Cell Proliferation Test Kit	Vazyme	A412-01
Aphidicolin	Sigma	A0781
dNDPs	MCE	HY-131576
dADP	MCE	HY-W010854
**Software**		
TopHat (v2.1.1.63)	Trapnell et al, 2012	
Cufflinks (v2.2.163)	Trapnell et al, 2012	
DEseq2 (v1.34.0)	Love et al, 2014	
**Other**		
4-well plate	Thermo Fisher	176740
U-bottom 96-well plate	Thermo Fisher	163320
TSQ Quantiva	Thermo Fisher	
LSM880	Zeiss	

### Animal maintenance

Wild-type C57BL/6 mice were purchased from Vital River and Tsinghua Animal Center. Both control and *Thap1*-knockout mice were raised at Tsinghua Animal Center. Mice were maintained under specific-pathogen-free conditions with a 12/12 h light/dark cycle in an environment of 20–22 °C and 55  ±  10% humidity. All animals were maintained according to the guidelines of the Institutional Animal Care and Use Committee of Tsinghua University, Beijing, China.

### Oocyte and early embryo collection

Growing and fully-grown oocytes (diameters over 60 μm and 70 μm, respectively) were collected from the ovaries of D14- and D21- or D60- female mice. The fully-grown oocytes were collected from female mice injected with 5 IU pregnant mare serum gonadotropin (PMSG) after 46–48 h (Ningbo Second Hormone Factory, 110254564). For MII oocyte and embryo collection, C57BL/6 female mice were injected with PMSG followed 48 h by 5 IU human chorionic gonadotrophin (hCG) (Ningbo Second Hormone Factory, 110251283). For embryo collection, females were mated with C57BL/6 males following hCG administration. Pronuclear stage 3 (PN3) zygotes, pronuclear stage 5 (PN5) zygotes, early two-cell embryos (E2C), late two-cell embryos (L2C), four-cell embryos (4C), and blastocyst-stage embryos were collected at 20, 27, 33, 48, 55, and 99 h post hCG in vivo, respectively. For ex vivo embryo culture, PN5 zygotes, E2C, middle two-cell embryos (M2C), L2C, 4C, and blastocyst-stage embryos were collected at 27, 36, 44, 52, 66, and 122 h post hCG as embryos tended to develop slower. Oocytes and embryos were collected in the M2 medium (Sigma-Aldrich, M7167). The embryos were cultured in KSOM medium (Milipore, Mr121d).

### Generation of *Thap1* knockout mice

The *Thap1*^fl/fl^ mice were generated by Cyagen Biosciences. Cas9 mRNA (100 ng/μl) and four single guide RNA (50 ng/μl each) were injected into the cytoplasm of zygotes (Table EV1). Following injection, zygotes were cultured in KSOM to the 2C stage at 37 °C under 5% CO_2_ in air. 2C embryos were transferred into the oviducts of surrogate ICR strain mothers. *Gdf9*-Cre mice were a gift from F. Gao. *Thap1*^fl/fl^; *Gdf9*-Cre mice were obtained by crossing *Thap1*^fl/fl^ mice with *Gdf9*-Cre mice to specifically ablate *Thap1* in the primordial follicle. mKO embryos were harvested by mating *Thap1*^fl/fl^; *Gdf9*-Cre females with WT C57BL/6 males. The *Thap1*^fl/fl^; *Gdf9*-Cre mice were genotyped by PCR (Table EV1).

### Immunostaining

Mouse oocytes and embryos were fixed with 4% paraformaldehyde (Sigma-Aldrich, P6148) for 30 min and then permeabilized with 0.5% Triton X-100 in PBS for 15 min. Samples were blocked with 1% BSA for 1 h and incubated at 4 °C overnight with primary antibodies: RRM1 (Proteintech, 10526-1-AP, 1:100 diluted), TUBULIN (Sigma, F2168, 1:100 diluted), γH2AX (Cell Signaling Technology, 9718S), pCHK1 (Cell Signaling Technology, 2348T). The primary antibody was washed away with 1× PBST (0.1% Triton X-100 in PBS) three times and samples were then incubated with secondary antibody (Thermo Fisher Scientific, A-21207, 1:400 diluted) and Hoechst 33342 (Sigma, B2261, 1:1000 diluted) for 1 h. Samples were then washed with 1× PBST three times. For EdU staining, embryos were collected and cultured in KSOM containing 50 mM EdU (Vazyme, A412-01) at 37 °C for 2 h prior to fixation, followed by immunofluorescent staining, which was described above. All immunofluorescence images were recorded using a Zeiss LSM880 confocal microscope.

### In vitro transcription and microinjection

For mRNA samples, pRK5 vectors containing a T3 promoter were linearized and transcribed with the T3 mMESSAGE Kit (Invitrogen, AM1348) following the manufacturer’s instructions. mRNAs were recovered by RNA Clean XP beads (Beckman, A63987). For *Thap1*-mKO rescue experiments, the concentration of *Thap1*, *Rrm1*, and *Rrm1* W684G mRNA was 100 ng/μl each. For PIP-FUCCI experiment, the concentration of FUCCI mRNA was 125 ng/μl. For microinjection, FGOs were injected at 46–48 h post PMSG. MII oocytes and zygotes were injected at 14–16 h, and 21–23 h post hCG in M2 medium (Sigma, M7167), respectively. All injections were performed with an Eppendorf Transferman NK2 micromanipulator. Samples were injected at 5–10 pl per zygote.

### APH treatment

To block DNA synthesis, PN3 zygotes were collected and cultured in KSOM medium supplemented with aphidicolin (Sigma, A0781, 3 μg/μl) for 32 h.

### dNDP rescue

dNDPs (MCE, HY-131576, HY-W010854) were mixed in equal proportions and diluted in KSOM medium to the final total concentration of 500 μM (each with 125 μM). The zygotes were collected and cultured in KSOM medium with the diluted dNDPs.

### Western blotting

Oocytes were prepared by lysing and denaturing in 5× sodium dodecyl sulfate (SDS) sample buffer (containing β-mercaptoethanol) and heated for 15 min at 95 °C. Proteins were separated by SDS-polyacrylamide gel electrophoresis and electrophoretically blotted onto microporous polyvinylidene fluoride (PVDF) membranes (Millipore, IPVH00010) under a constant current, followed by blocking in 1× TBST containing 5% skim milk for 1 h at room temperature and incubated at 4 °C overnight with primary antibodies: RRM1 (Proteintech, 10526-1-AP; 1:1000 diluted) or ACTIN (Yeasen, 30101ES50; 1:5000 diluted). After washing with 1× TBST, the membrane was incubated with secondary antibody for 1 h at room temperature, followed by three washes with 1× TBST. Bound antibodies were detected using an enhanced chemiluminescence western blotting substrate (Pierce, cat. no. 34076).

### HPLC-MS

HPLC-grade methanol 100 μl 80% (v/v) (pre-chilled at −80 °C) was added to the sample tube, followed by vortexing for 1 min and incubating at −80 °C for 1 h. The sample tube was then centrifuged at 14,000× *g* for 20 min using a refrigerated centrifuge at 4 °C and 90 μl supernatant was transferred to a new Eppendorf tube. The pellet was lyophilized to dry and re-diluted in 25 μl water. Targeted metabolomic experiment was performed by TSQ Quantiva (Thermo Fisher Scientific, CA). This experiment focused on the TCA cycle, glycolysis pathway, pentose phosphate pathway, amino acids, and purine metabolism. The injection volume was 15 μl. C18 based reverse phase chromatography was utilized with 10 mM tributylamine, 15 mM acetate in water, and 100% methanol as mobile phase A and B, respectively. In this experiment, 25-min gradient from 5% to 90% mobile B was used. A positive-negative ion switching mode was performed for data acquisition. The resolution for Q1 and Q3 were both 0.7 FWHM. The source voltage was 3500 v for positive and 2500 v for negative ion mode. The source parameters were as follows: spray voltage: 3000 v; capillary temperature: 320 °C; heater temperature: 300 °C; sheath gas flow rate: 35; auxiliary gas flow rate: 10. Metabolite identification was based on Tracefinder search with home-built database containing about 300 compounds.

### RNA-seq

All RNA-seq libraries were generated following the Smart-seq2 protocol as described previously (Picelli et al, 2014). The zona pellucida was gently removed by treatment with Tyrode’s solution (Sigma, T1788). Oocytes and embryos were washed three times in M2 medium and then lysed in 2 μl of lysis buffer containing RNase inhibitor.

### Data analysis

#### RNA-seq analysis

Paired-end RNA-seq reads were trimmed and mapped to the mm9 genome by TopHat (v2.1.1.63) (Trapnell et al, 2012). Cufflinks (v2.2.163) (Trapnell et al, 2012) was used to calculate the FPKM per gene based on mm9 refFlat from the UCSC genome annotation database. Differential expression analysis was performed with DEseq2 (v1.34.0) (Love et al, 2014). Genes with *P* value < 0.05 and log2 (fold change) ≤ −1 were considered as downregulated genes. Genes with *P* value < 0.05 and log2 (fold change) ≥1 were considered as upregulated genes.

#### Identification of quantifiable metabolites

To identify metabolites of which the levels could be quantitatively measured by mass spectrometry, HPLC-MS was performed in 50, 100, 200, and 500 MII oocytes. Compared to the metabolite abundance in 50 MII oocytes, if the abundance of a metabolite increased correspondingly by two-, four-, and tenfold, respectively, with a deviation within 0.5, the metabolites were considered as “quantifiable metabolites”.

#### Identification of dynamic metabolites

Each metabolite’s coefficient of variation (CV) across stages (the D14, D21, and D60 oocytes, PN3 one-cell embryos, and PN5 one-cell embryos) was calculated (CV = standard variation/mean). The metabolites with an empirical CV > 0.08 were defined as dynamic metabolites (*n* = 18).

## Supplementary information

Table EV1

Appendix

Peer Review File

Data Set EV1

Data Set EV2

Data Set EV3

Data Set EV4

Data Set EV5

Data Set EV6

Data Set EV7

Data Set EV8

Source data Fig. 1

Source data Fig. 2

Source data Fig. 3

Source data Fig. 4

Source data Fig. 5

Source data Fig. 6

Source data Fig. 7

Appendix Source Data

## Data Availability

The generated and analyzed data are available in the Gene Expression Omnibus with accession number GSE283275. The source data of this paper are collected in the following database record: biostudies:S-SCDT-10_1038-S44319-026-00712-9.
