# Supplementary material for: THAP1 is a maternal effect factor required for the first cell cycle via Rrm1 in early mouse embryos
Source: EMBO Rep. 2026 Feb 23;27(7):1813–29. doi: 10.1038/s44319-026-00712-9 (PMC13077089; doi:10.1038/s44319-026-00712-9)
Supplement: Supplementary file 2 — Appendix [file 44319_2026_712_MOESM2_ESM.pdf]

Appendix

THAP1 is a maternal effect factor required for the first cell cycle via *Rrm1* in early mouse embryos

Qiang Fan[1,2,*], Xi Wu[1,2,3,*], Yanna Dang[1,2,*], Lijun Dong[1,2], Wenying Wang[1,2,] Feng Kong[1,2], Lijuan Wang[1,2], Xukun Lu[4], Boyang Liu[4], Shuyan Ji[1,2,5#], Wei Xie[1,2,#]

# Correspondence to: 0025039@zju.edu.cn (Shuyan Ji); xiewei121@tsinghua.edu.cn (Wei Xie)
* These authors contributed equally.

List of Appendix Figures:

Appendix Figure S1………………..page 2
Appendix Figure S2………………..page 3
Appendix Figure S3………………..page 5
Appendix Figure S4………………..page 7
Appendix Figure S5………………..page 8
Appendix Figure S6………………..page 9
Appendix Figure S7………………..page 11
Appendix Figure S8………………..page 12
Appendix Figure S9………………..page 13

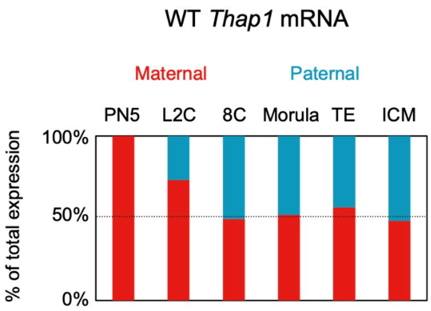

Appendix Figure S1. Expression of *Thap1* from the mouse maternal or paternal allele. Bar charts showing the percentages of the *Thap1* mRNA in mouse embryos from the maternal or the paternal allele.

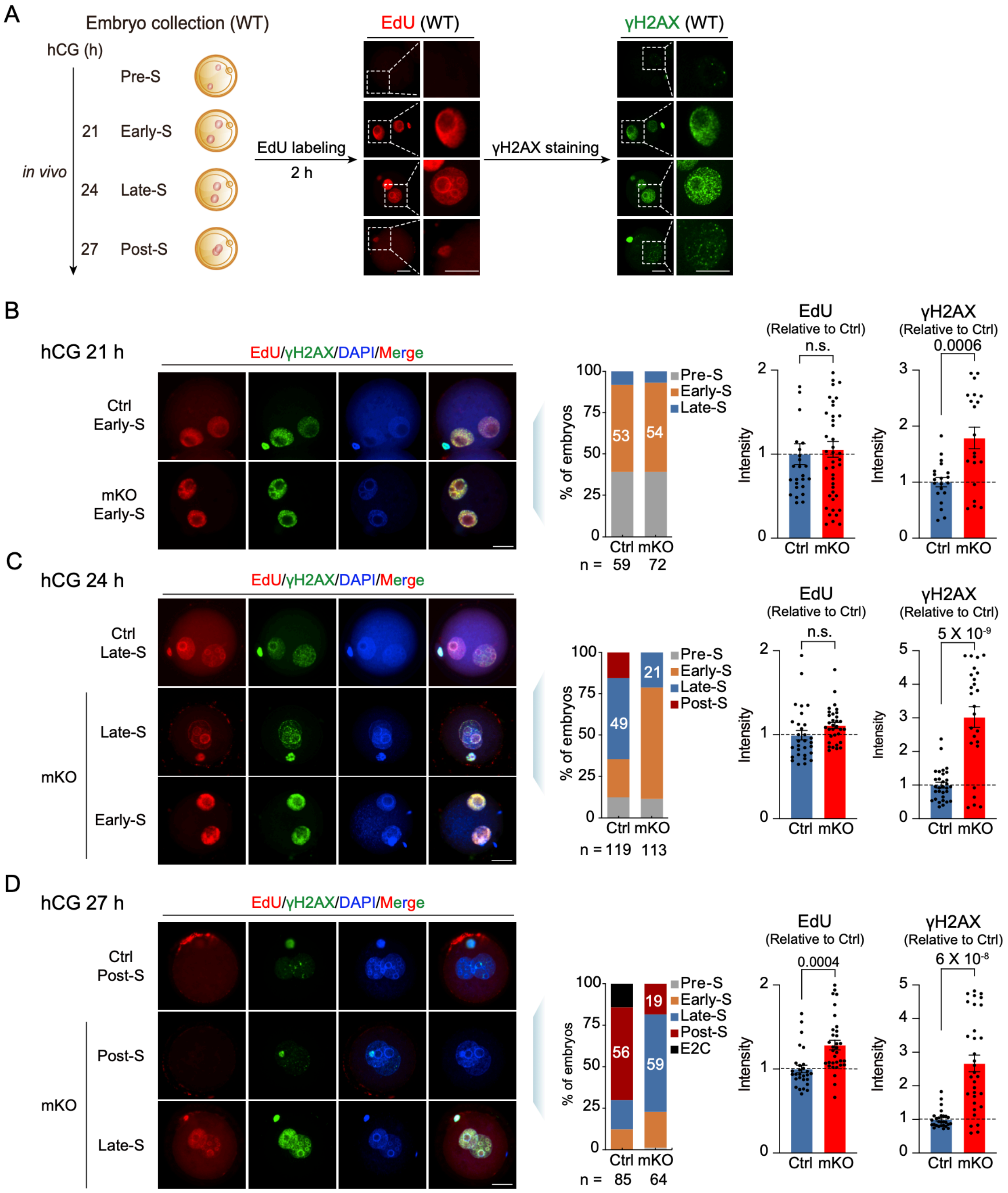

Appendix Figure S2. Maternal *Thap1* depletion led to DNA replication defects and elevated DNA damage. (**A**) EdU incorporation (red) and γH2AX (green) staining in wild-type zygotes at different time points. Scale bar, 20 µm. (**B-D**) Left, immunofluorescence of EdU and γH2AX in control and *Thap1*-mKO embryos at 21 h, 24 h, and 27 h post-hCG, respectively (three biological replicates). Scale bar, 20 µm. Middle, bar charts showing the percentages of embryos in control and *Thap1*-mKO embryos at 21 h, 24 h, and 27 h post-hCG, respectively (three biological replicates). Right, bar charts showing the relative intensities of EdU and γH2AX in control and *Thap1*-mKO embryos at 21 h, 24 h, and 27 h post-hCG, respectively (three biological replicates). The intensities of nuclear signals are normalized to those in cytoplasm; the ratio for each embryo is further normalized to the average values

of control embryos. Each dot represents a single embryo. n, the total number of embryos. Error bars, standard error of the mean. n.s., not significant; unpaired t-test.

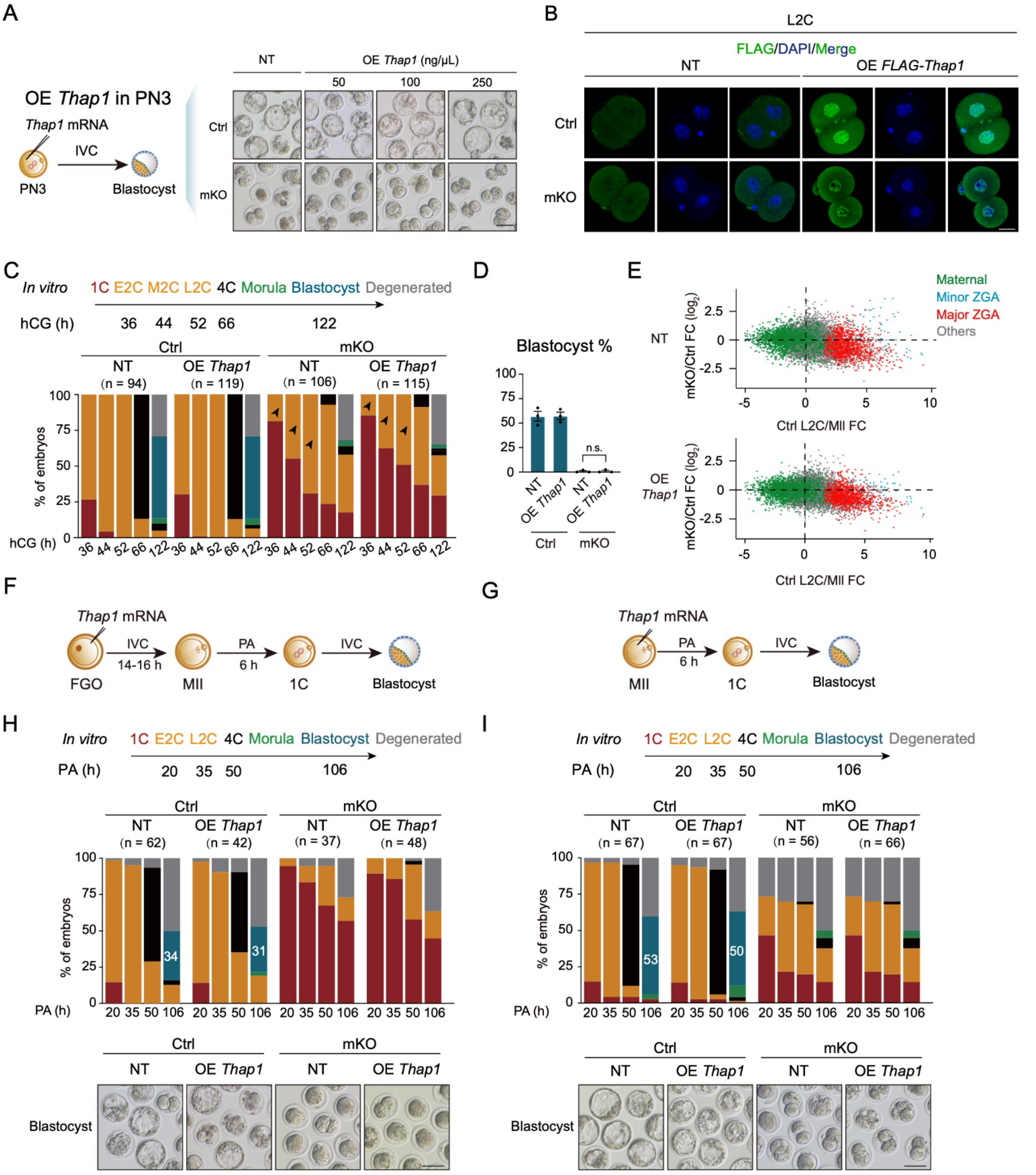

Appendix Figure S3. Overexpression of THAP1 in zygote, FGO or MII oocyte could not rescue *Thap1*-mKO defects. (**A**) Left, schematic of *Thap1* rescue in *Thap1*-mKO zygotes followed by *in vitro* culture (IVC). Right, morphology of control and *Thap1*-mKO embryos with or without the overexpression of *Thap1* in zygotes. (**B**) Immunofluorescence of FLAG-THAP1 in mouse 2C embryos with or without the overexpression of FLAG-*Thap1* in zygotes. Scale bar, 20 μm. (**C**) Developmental rates of control and *Thap1*-mKO embryos with or without the overexpression of *Thap1* mRNA (100 ng/μl) in zygotes (three biological replicates). Arrows indicate the percentages of 2C embryos. NT, no treatment. n, the total number of embryos. Error bars, standard error of the mean. (**D**) Bar charts

showing the percentages of blastocyst in control and *Thap1*-mKO embryos with or without the overexpression of *Thap1* mRNA (100 ng/μl) in zygotes. n.s., non-significant (p-value = 0.9, unpaired t-test). Scale bar, 75 μm. Each dot represents a biological replicate. (**E**) Scatter plots showing gene expression fold-changes upon maternal deletion of *Thap1* with (top) or without (bottom) the overexpression of *Thap1* in L2C embryos. Maternal genes, major ZGA genes, and minor ZGA genes are color-coded (two biological replicates). (**F-G**) Schematic of *Thap1* overexpression in *Thap1*-mKO FGO (**F**) or MII (**G**) followed by *in vitro* culture (IVC). PA, Parthenogenetic activation. (**H-I**) Developmental rates (top) and embryonic morphology (bottom) of control and *Thap1*-mKO embryos with or without the overexpression of *Thap1* mRNA, respectively, in FGO (**H**) or MII oocyte (**I**) (two biological replicates). n, the total number of embryos. Scale bar, 75 μm.

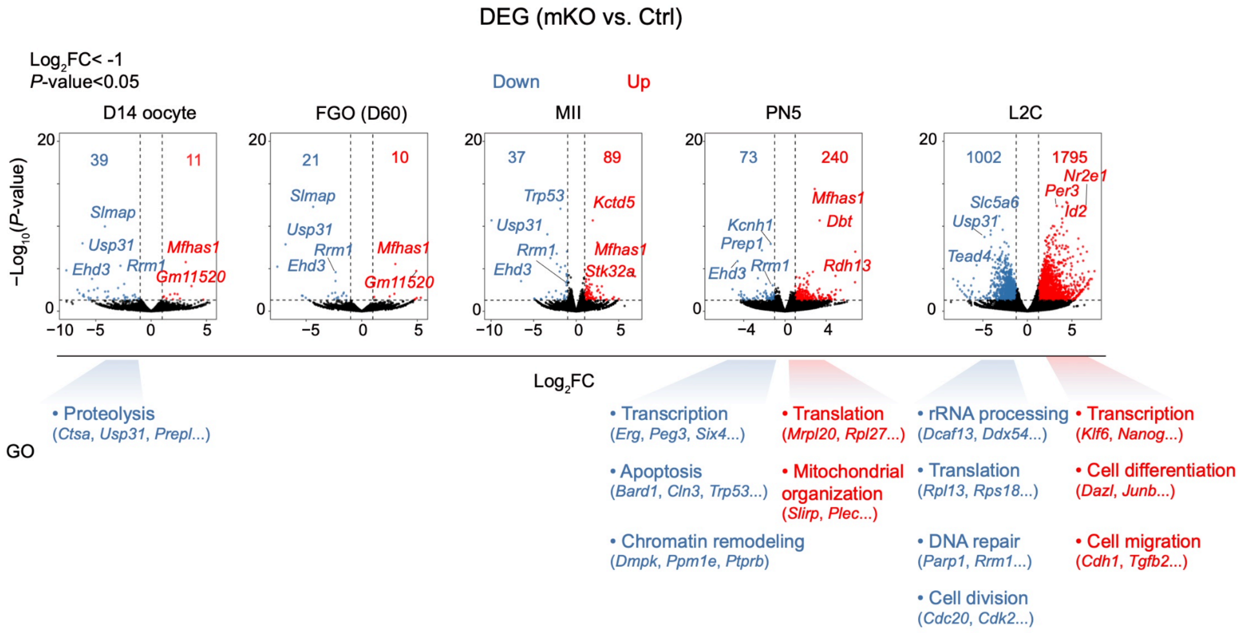

Appendix Figure S4. THAP1 regulates a small subset of genes in oocytes. Volcano plots showing gene expression changes upon *Thap1* depletion (two biological replicates for each stage). Horizontal dashed line, *P*-value threshold 0.05. Left vertical dashed line, $\log_2$ fold change threshold -1. Right vertical dashed line, $\log_2$ fold change threshold 1. Up- and down-regulated genes are color-coded. GO terms and example genes are indicated.

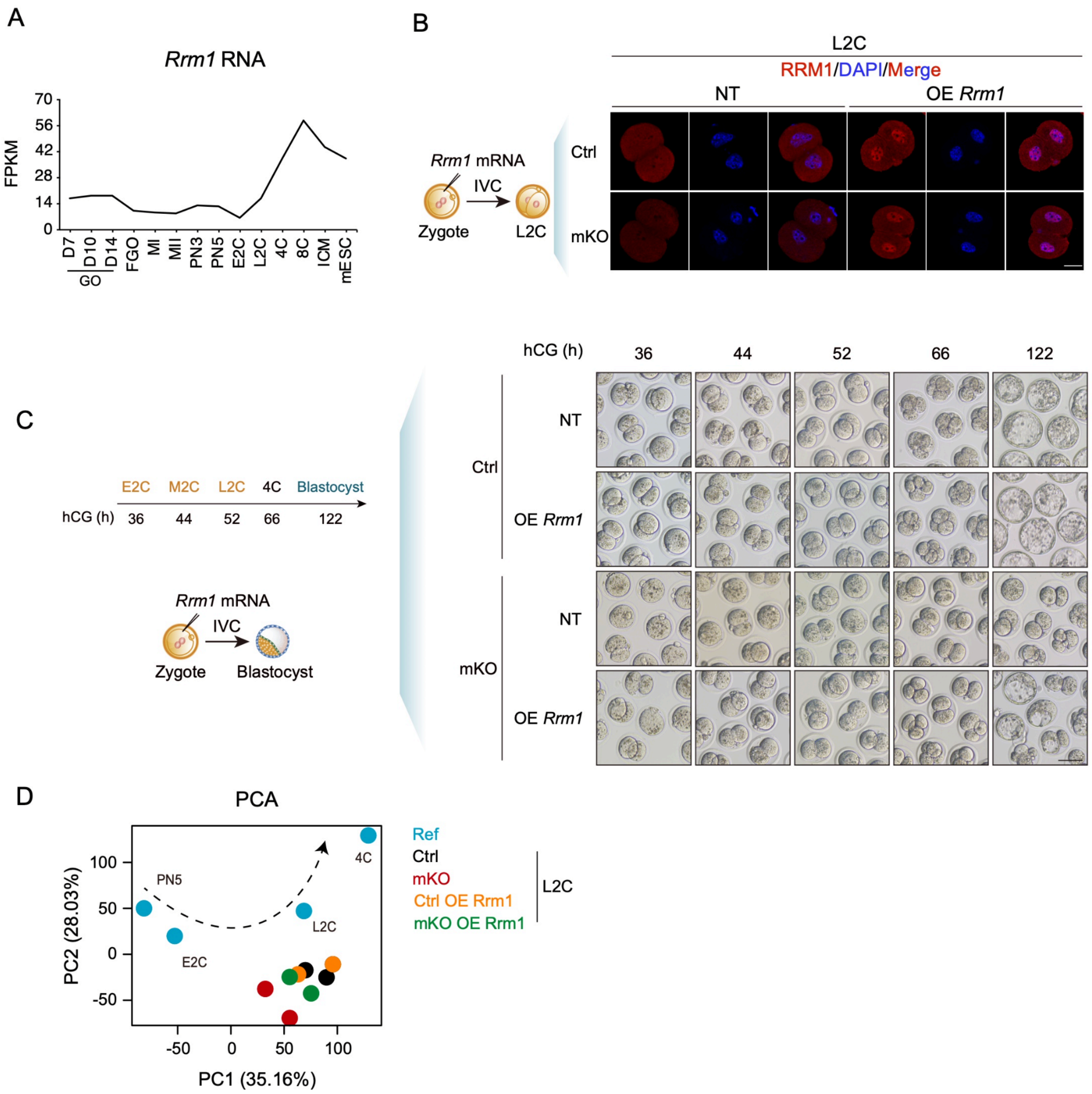

Appendix Figure S5. RRM1 could partially rescue the developmental defects in *Thap1*-mKO embryos. (**A**) Line plot showing the gene expression of *Rrm1* in mouse oocytes, embryos, and mESCs (Zhang et al., 2016). (**B**) Left, schematic of *Rrm1* rescue in *Thap1*-mKO zygotes followed by *in vitro* culture. Right, immunofluorescence of RRM1 in mouse 2C embryos with or without the overexpression of *Rrm1* in zygotes (two biological replicates). Scale bar, 20 μm. (**C**) Left, schematic of *Rrm1* rescue in *Thap1*-mKO zygotes followed by *in vitro* culture. Right, embryo morphology of control and *Thap1*-mKO embryos with or without the overexpression of *Rrm1* in zygotes. NT, no treatment (three biological replicates). Scale bar, 75 μm. (**D**) PCA analysis of the transcriptiome data of control (black), mKO (red), control OE *Rrm1* (orange), and mKO OE *Rrm1* (green) embryos. Reference RNA-seq data (WT, blue) from the PN5 to 4C embryos (Zhang et al., 2016) are used to show the developmental trajectory.

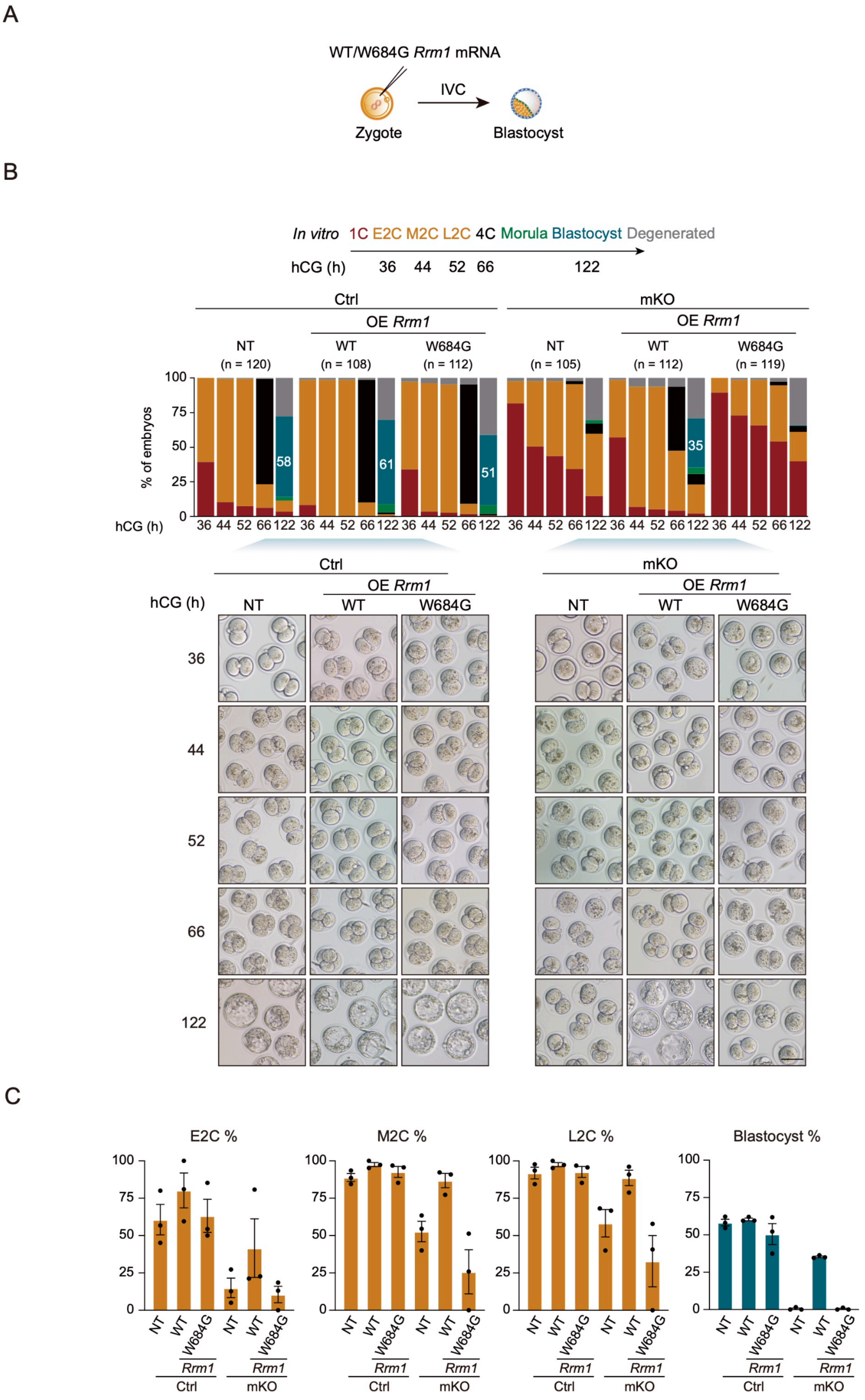

Appendix Figure S6. RRM1 mutant could not rescue the developmental defects in *Thap1*-mKO embryos. (**A**) Schematic of the overexpression of wild-type (WT) and mutant (W684G) *Rrm1* mRNA

in mouse zygotes followed by *in vitro* culture. (**B**) Developmental rates (top) and embryonic morphology (bottom) of control and *Thap1*-mKO zygotes with or without the overexpression of *Rrm1*^{WT} or *Rrm1*^{W684G} mRNA, respectively (three biological replicates). n, the total number of embryos. Scale bar, 75 μm. (**C**) Bar charts showing the percentages of the E2C (36 h), M2C (44 h), L2C (52 h), and blastocyst (122 h) embryos in control and *Thap1*-mKO embryos with or without the overexpression of *Rrm1* WT or W684G. Each dot represents a biological replicate. Error bars, standard error of the mean.

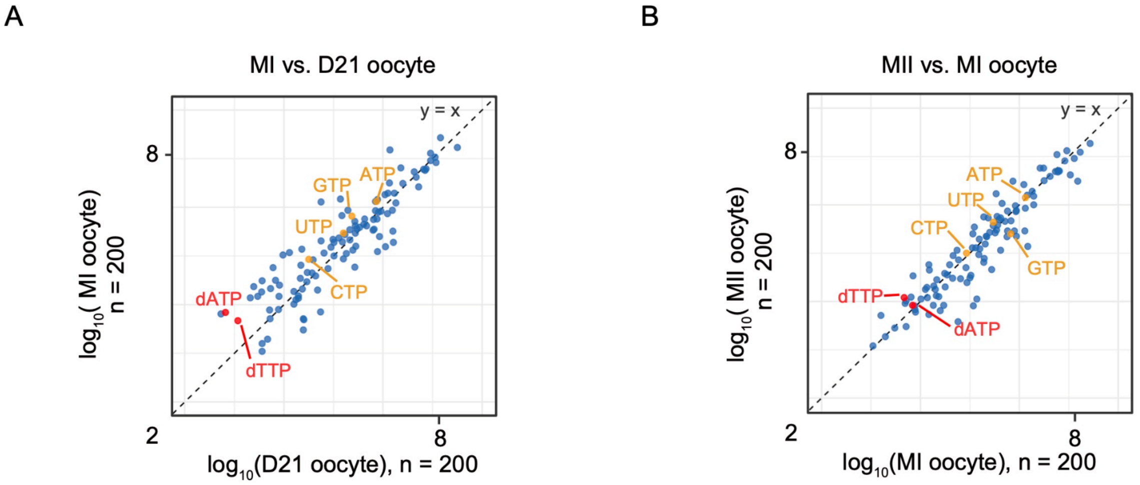

Appendix Figure S7. Dynamic metabolite abundance during oocyte maturation. (**A-B**) Scatter plot comparing metabolites' abundance (log_{10} transformed) between D21 oocytes and that of MI oocytes (**A**) or between MI oocytes and that of MII oocytes (**B**). n indicates oocyte number. NTP and dNTP are color-coded.

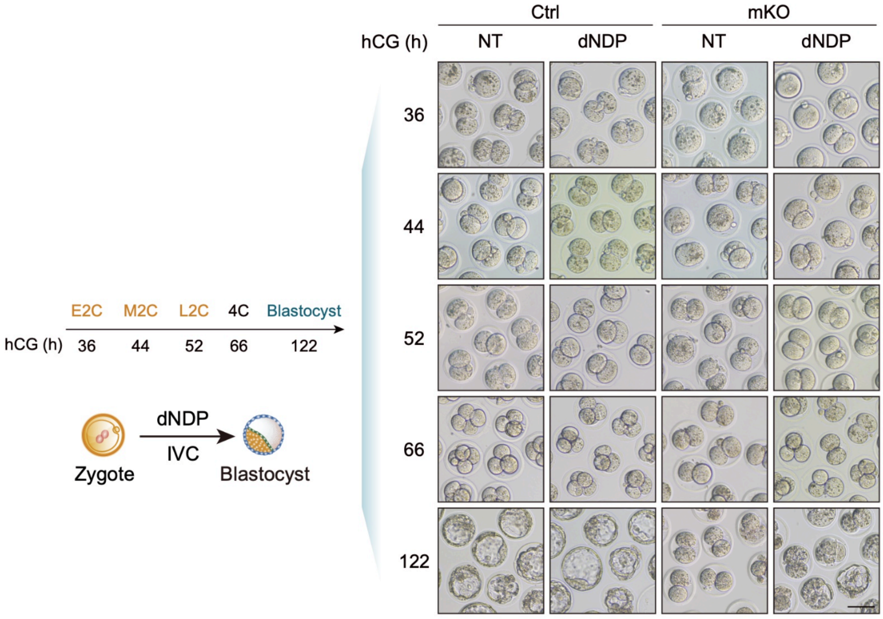

Appendix Figure S8. dNDPs could partially rescue the developmental deficiency of *Thap1*-mKO embryos. Schematic (left) and embryonic morphology (right) of control and *Thap1*-mKO embryos culturing in medium with or without dNDPs (three biological replicates). Scale bar, 75 μm.

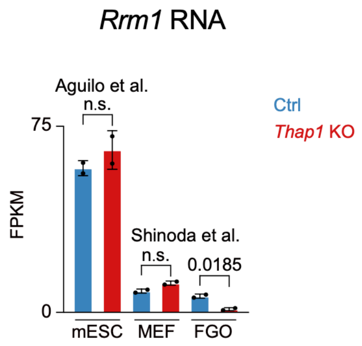

Appendix Figure S9. Expression of *Rrm1* in mouse oocytes and cell lines with or without *Thap1*. Bar charts showing gene expression of *Rrm1* in control and *Thap1*-KO mESCs (Aguilo et al., 2017), MEFs (Shinoda et al., 2021), and FGOs. Error bars, standard error of the mean. p-value (unpaired *t*-test) are also indicated. n.s., not significant; unpaired t-test.

Reference

Aguilo, F., Zakirova, Z., Nolan, K., Wagner, R., Sharma, R., Hogan, M., Wei, C., Sun, Y., Walsh, M.J., Kelley, K., *et al.* (2017). THAP1: Role in Mouse Embryonic Stem Cell Survival and Differentiation. Stem Cell Reports *9*, 92-107.

Shinoda, K., Zong, D., Callen, E., Wu, W., Dumitrache, L.C., Belinky, F., Chari, R., Wong, N., Ishikawa, M., Stanlie, A., *et al.* (2021). The dystonia gene THAP1 controls DNA double-strand break repair choice. Mol Cell *81*, 2611-2624 e2610.

Zhang, B., Zheng, H., Huang, B., Li, W., Xiang, Y., Peng, X., Ming, J., Wu, X., Zhang, Y., Xu, Q., *et al.* (2016). Allelic reprogramming of the histone modification H3K4me3 in early mammalian development. Nature *537*, 553-557.
