# Supplementary figures and images for: THAP1 is a maternal effect factor required for the first cell cycle via Rrm1 in early mouse embryos (part 1 of 2)
Source: EMBO Rep. 2026 Feb 23;27(7):1813–29. doi: 10.1038/s44319-026-00712-9 (PMC13077089; doi:10.1038/s44319-026-00712-9)

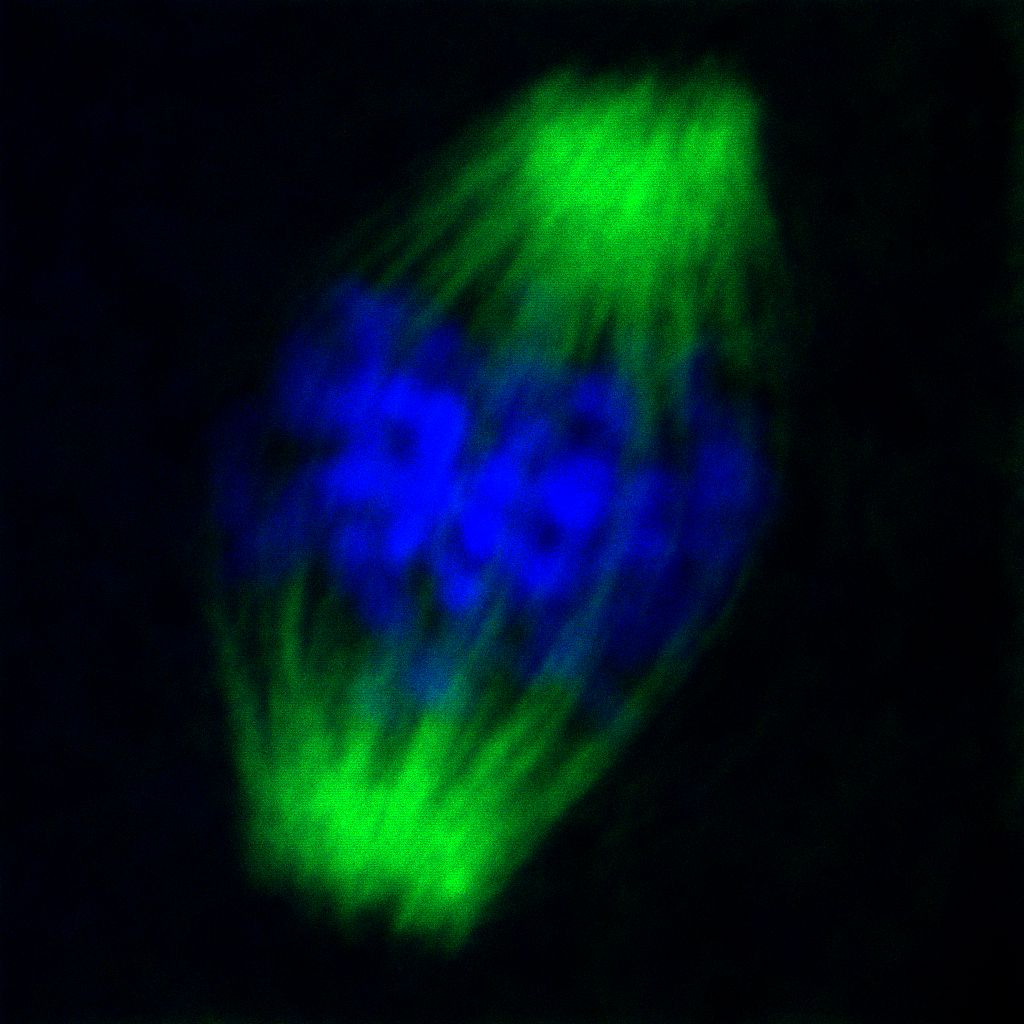

Supplement: Supplementary file 12 — Source data Fig. 1 [file 44319_2026_712_MOESM12_ESM.zip › Figure1 Source Data/1F/Ctrl_zoom in_merge.tif]

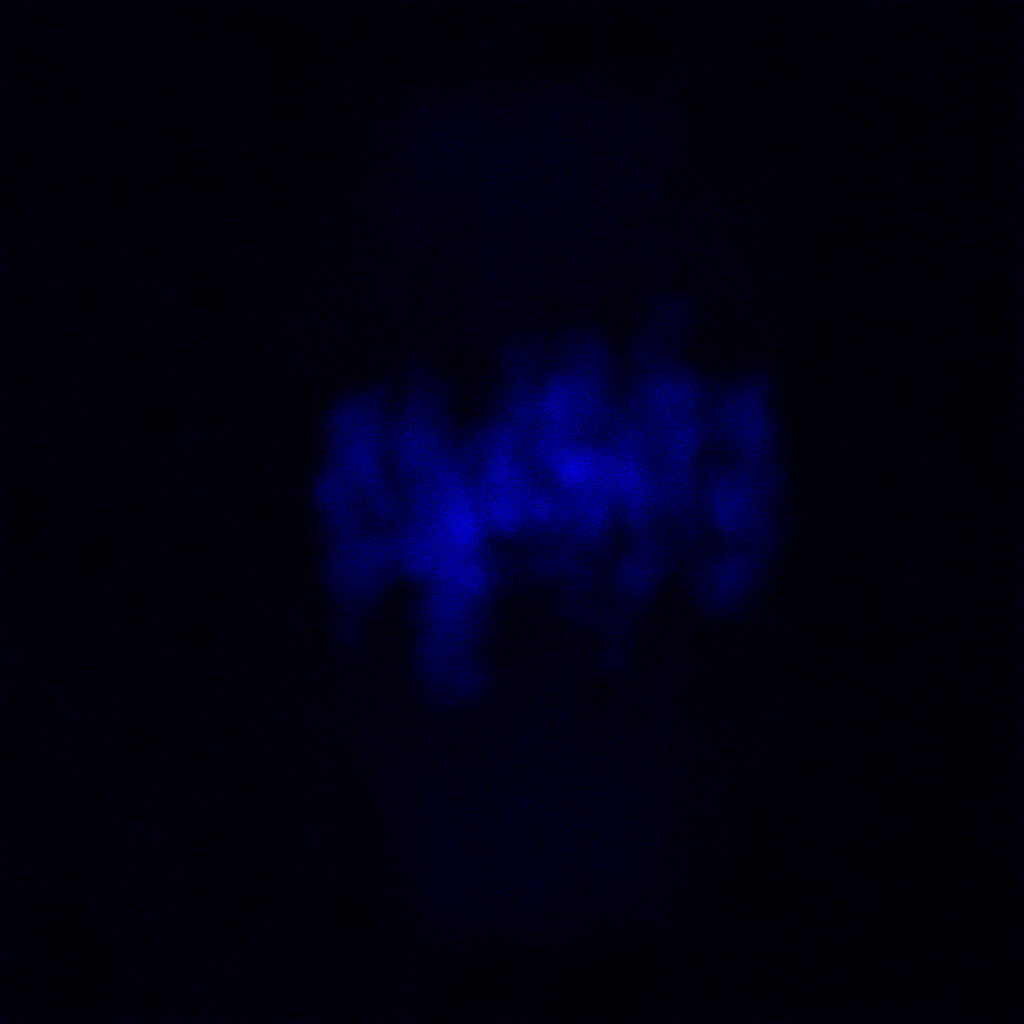

Supplement: Supplementary file 12 — Source data Fig. 1 [file 44319_2026_712_MOESM12_ESM.zip › Figure1 Source Data/1F/mKO_zoom in_DAPI.tif]

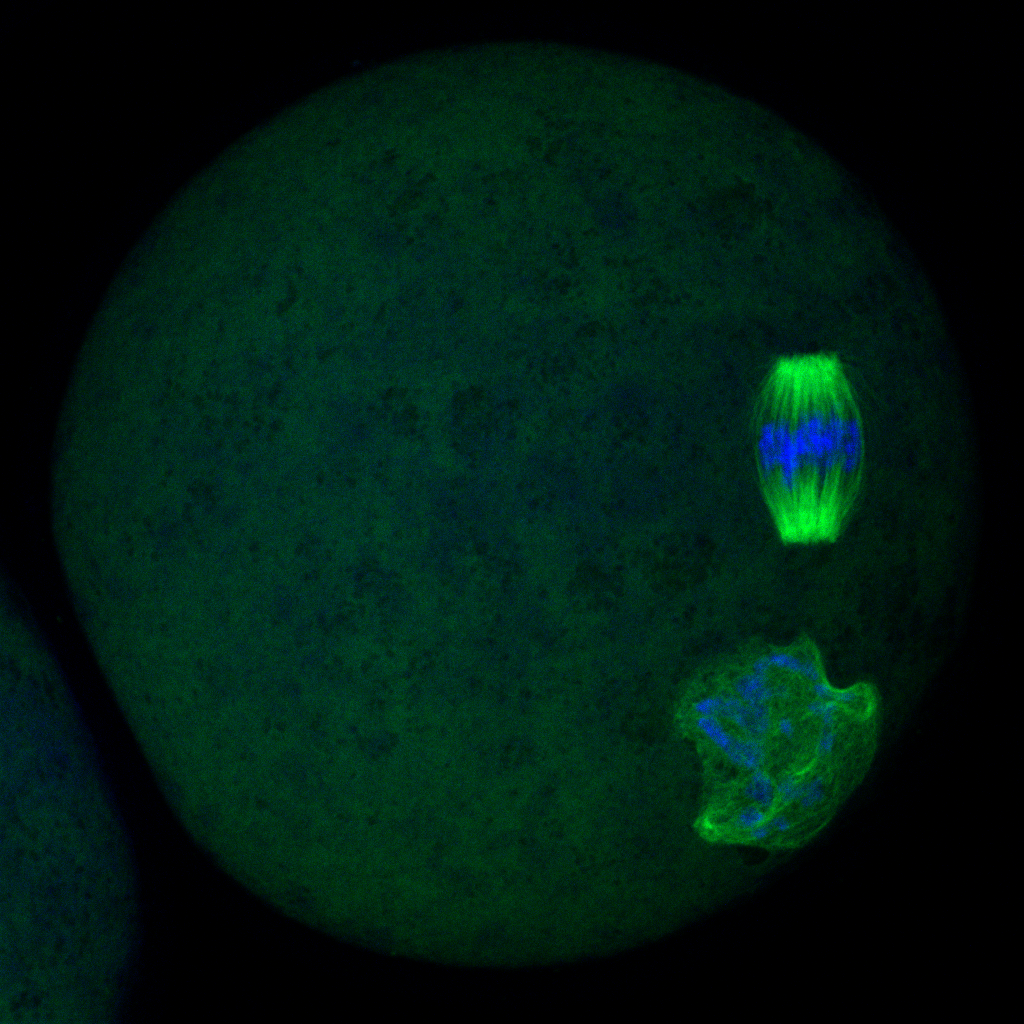

Supplement: Supplementary file 12 — Source data Fig. 1 [file 44319_2026_712_MOESM12_ESM.zip › Figure1 Source Data/1F/mKO_merge.tif]

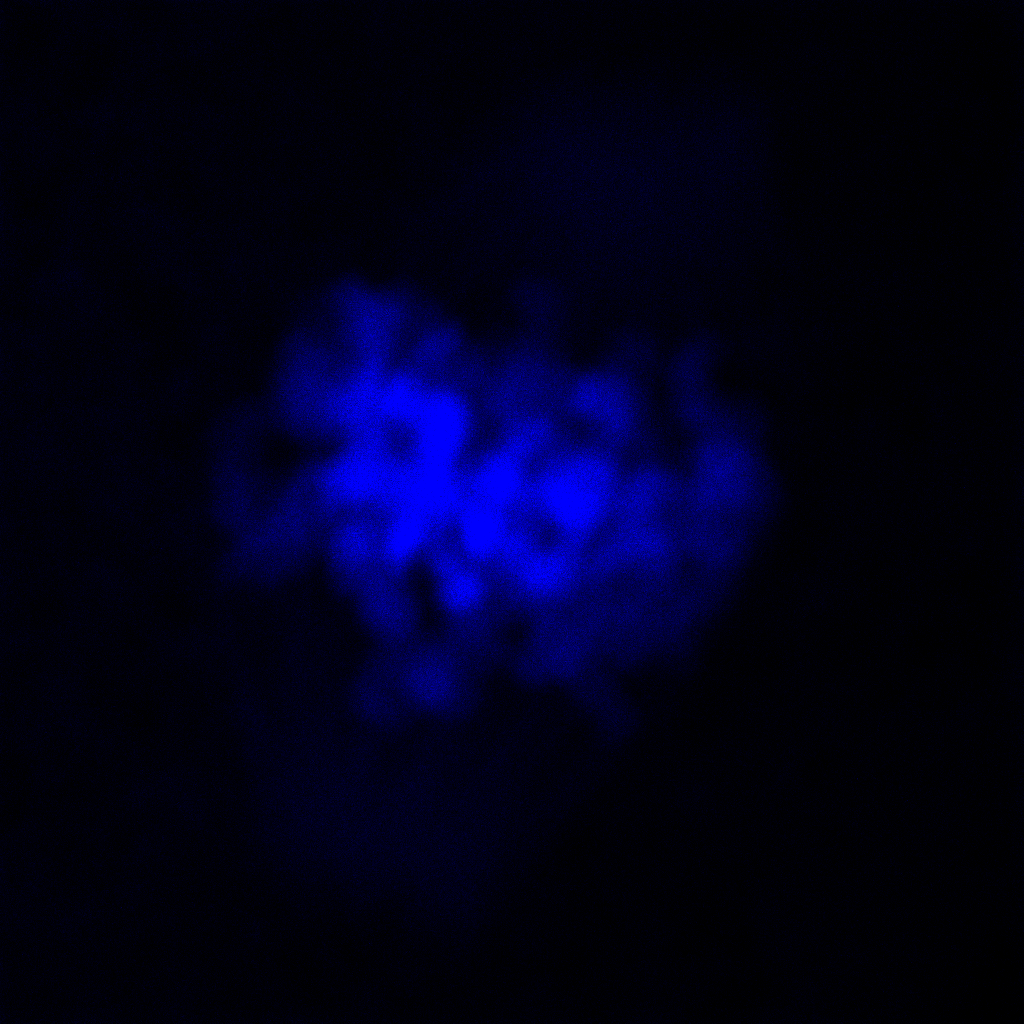

Supplement: Supplementary file 12 — Source data Fig. 1 [file 44319_2026_712_MOESM12_ESM.zip › Figure1 Source Data/1F/Ctrl_zoom in_DAPI.tif]

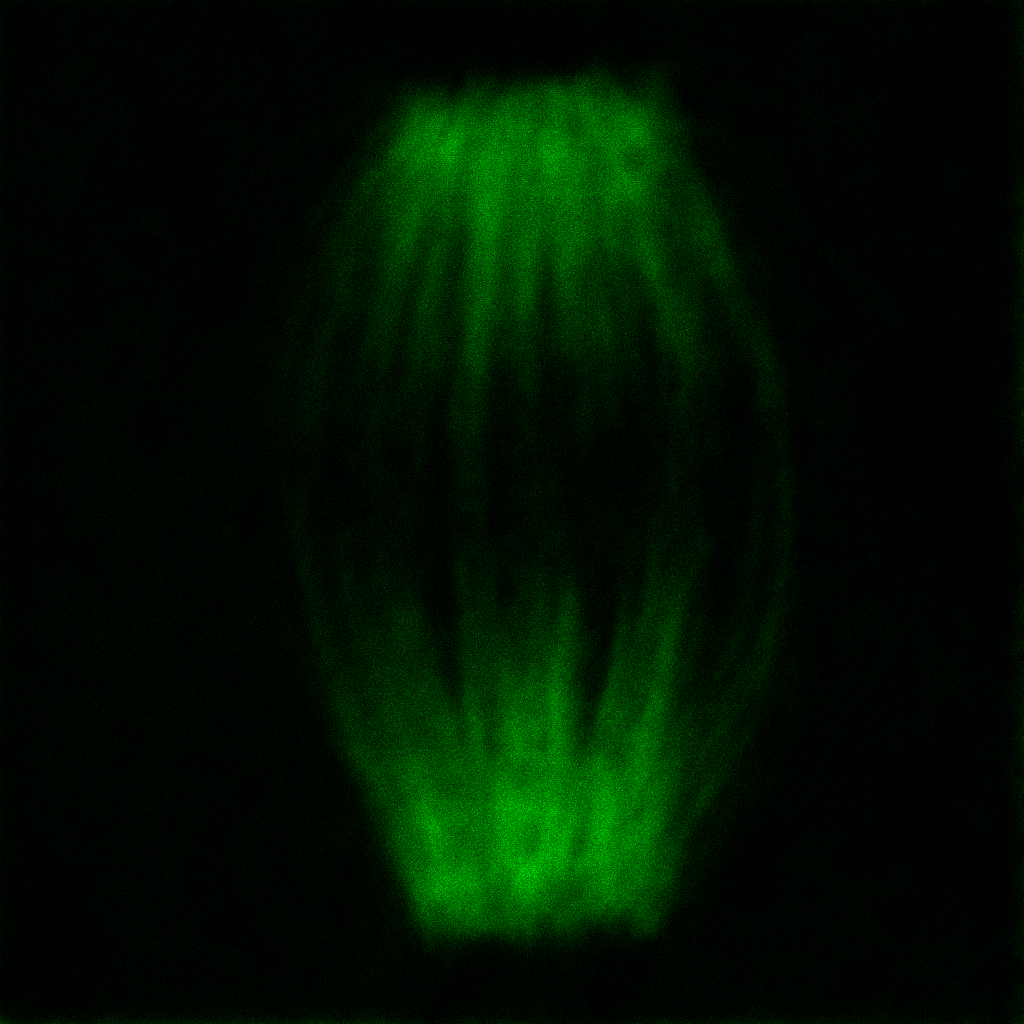

Supplement: Supplementary file 12 — Source data Fig. 1 [file 44319_2026_712_MOESM12_ESM.zip › Figure1 Source Data/1F/mKO_zoom in_tubulin.tif]

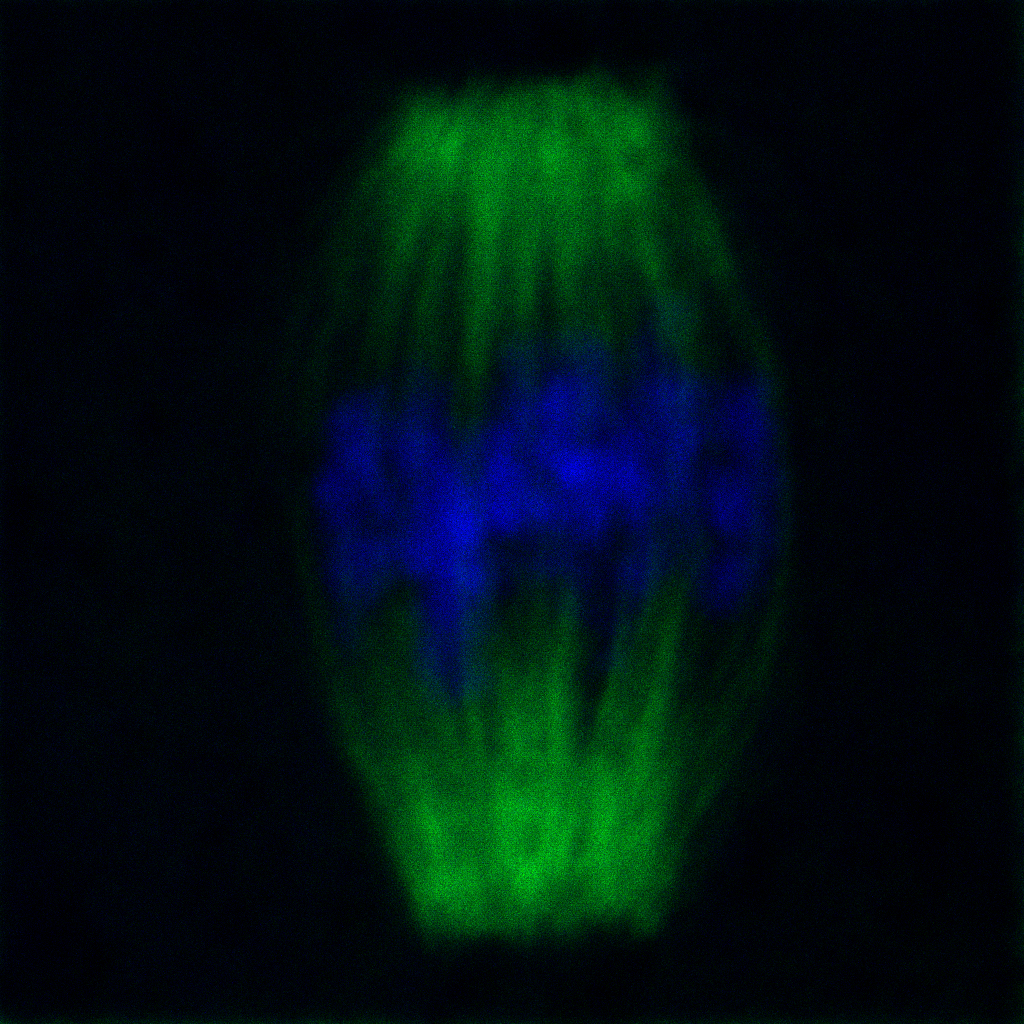

Supplement: Supplementary file 12 — Source data Fig. 1 [file 44319_2026_712_MOESM12_ESM.zip › Figure1 Source Data/1F/mKO_zoom in_merge.tif]

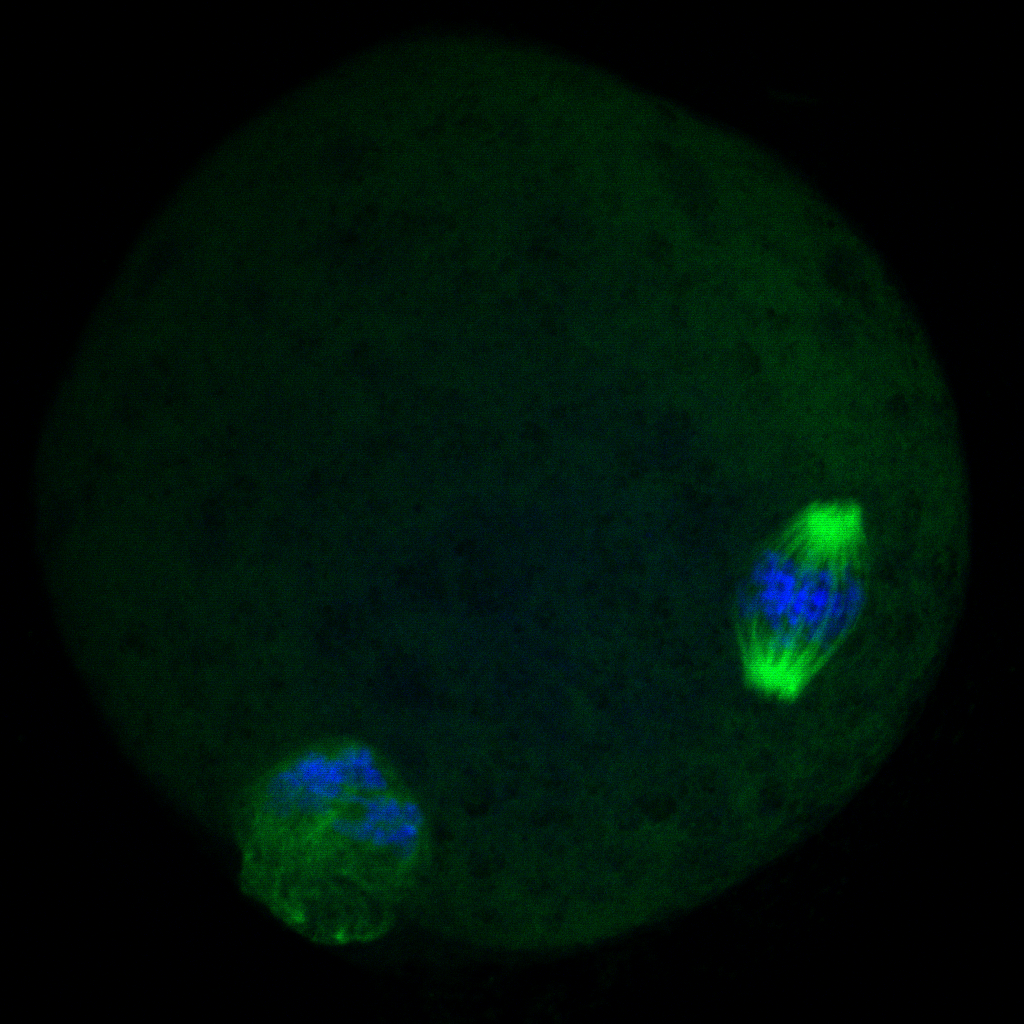

Supplement: Supplementary file 12 — Source data Fig. 1 [file 44319_2026_712_MOESM12_ESM.zip › Figure1 Source Data/1F/Ctrl_merge.tif]

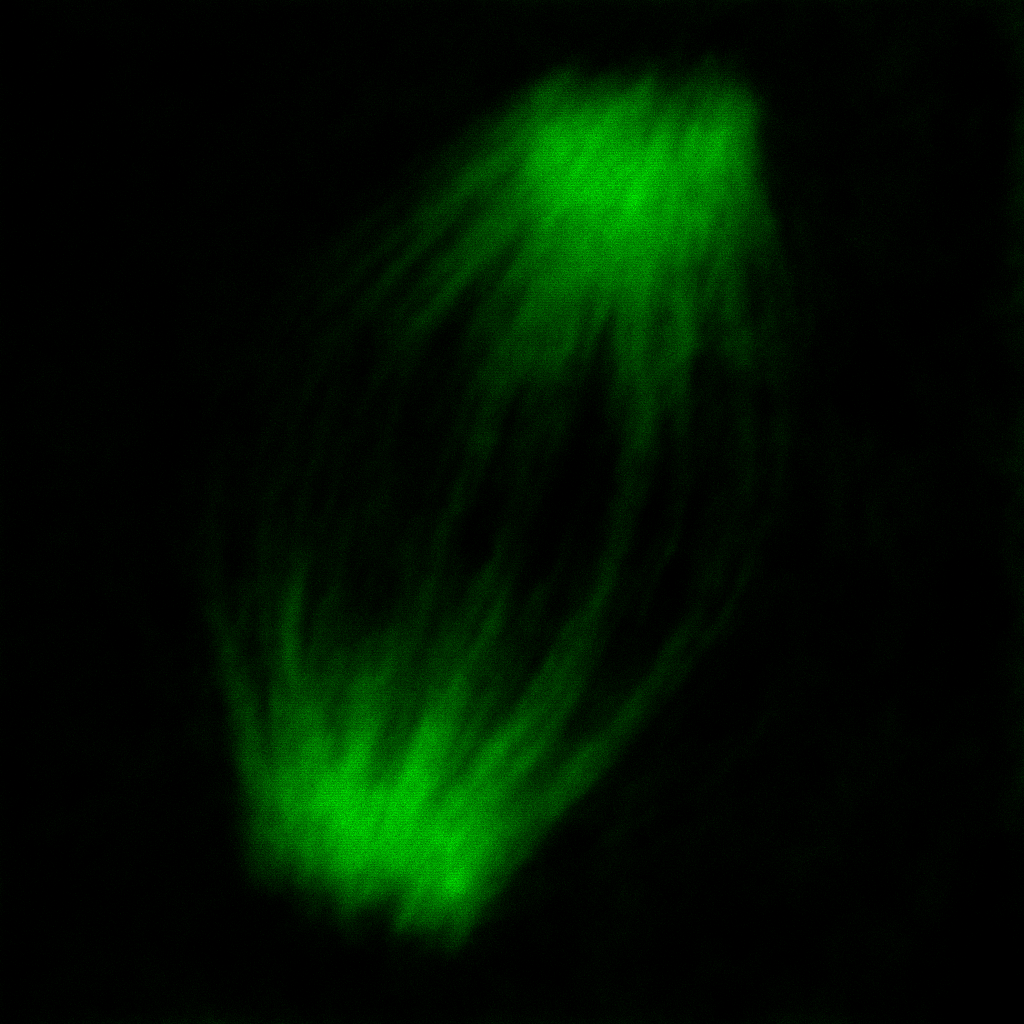

Supplement: Supplementary file 12 — Source data Fig. 1 [file 44319_2026_712_MOESM12_ESM.zip › Figure1 Source Data/1F/Ctrl_zoom in_tubulin.tif]

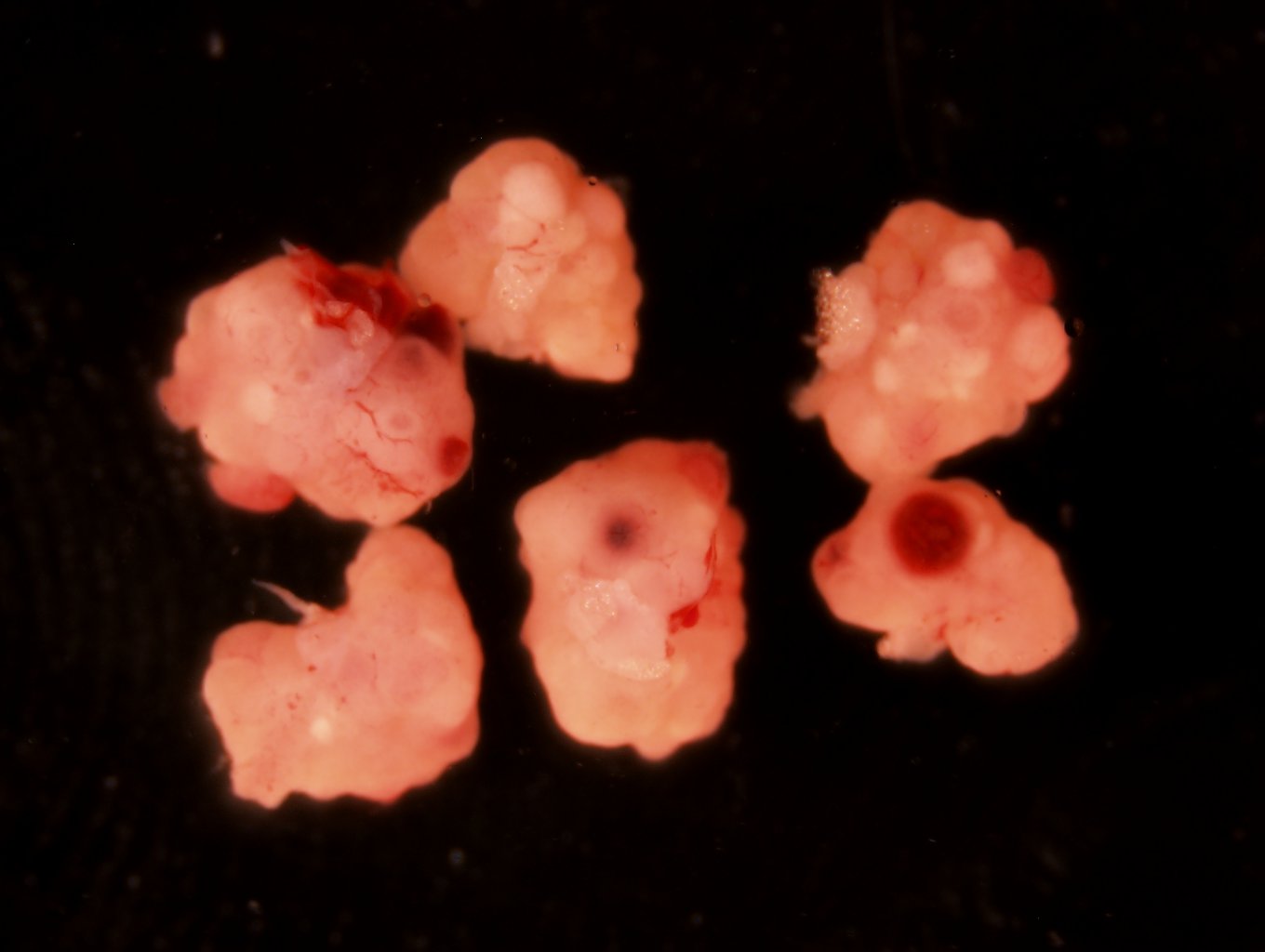

Supplement: Supplementary file 12 — Source data Fig. 1 [file 44319_2026_712_MOESM12_ESM.zip › Figure1 Source Data/1D/Ctrl_ovary.jpg]

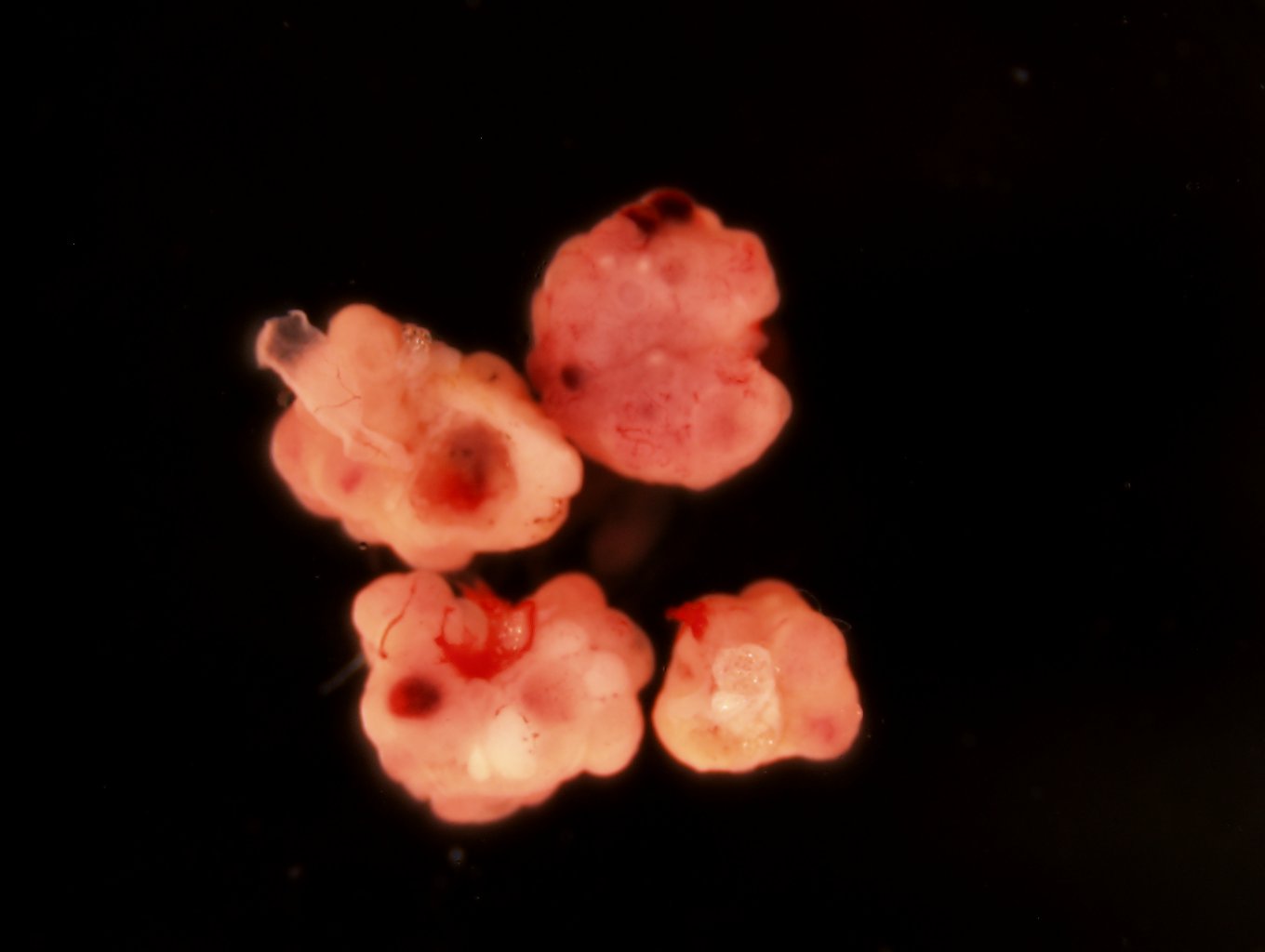

Supplement: Supplementary file 12 — Source data Fig. 1 [file 44319_2026_712_MOESM12_ESM.zip › Figure1 Source Data/1D/mKO_ko ovary.jpg]

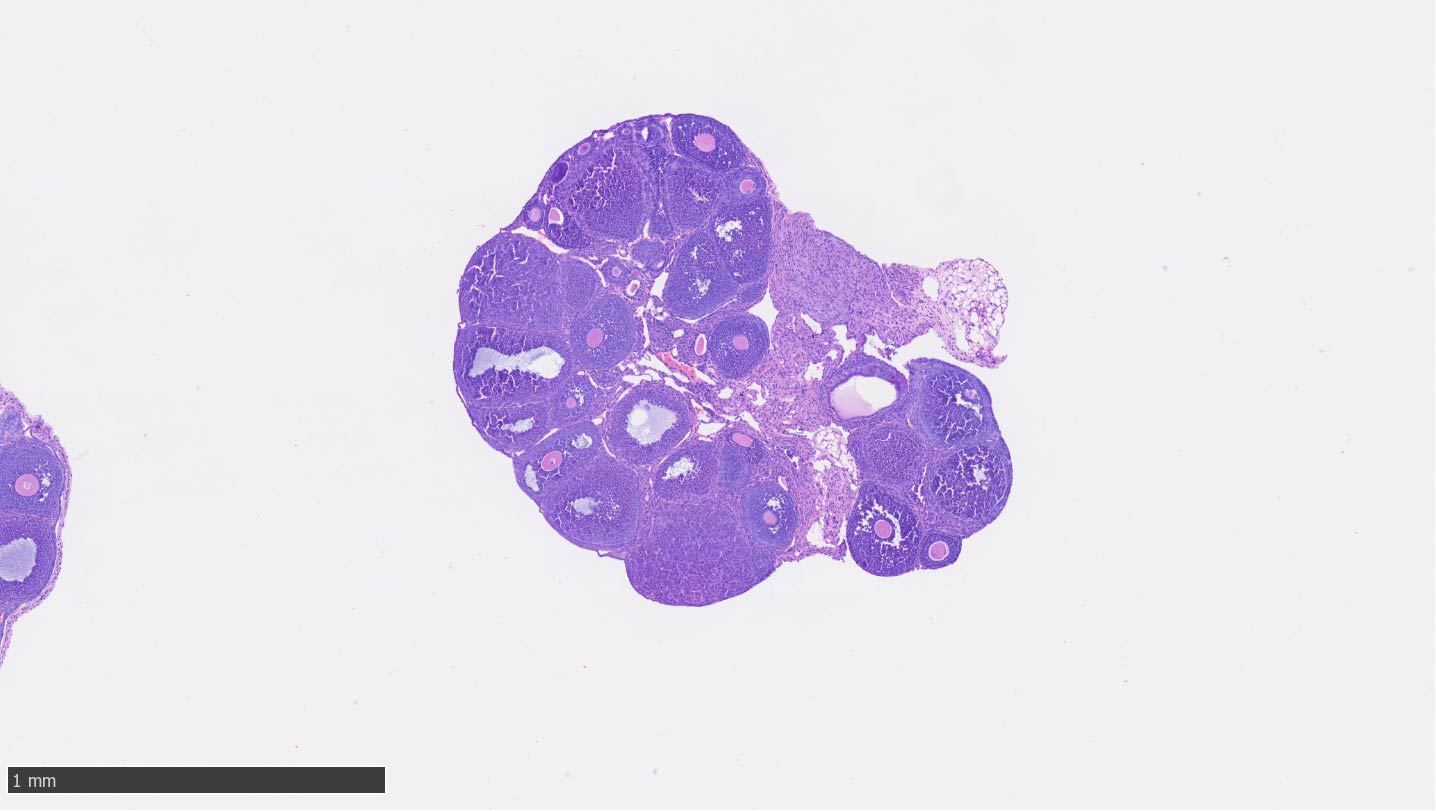

Supplement: Supplementary file 12 — Source data Fig. 1 [file 44319_2026_712_MOESM12_ESM.zip › Figure1 Source Data/1D/Ctrl_HE.jpg]

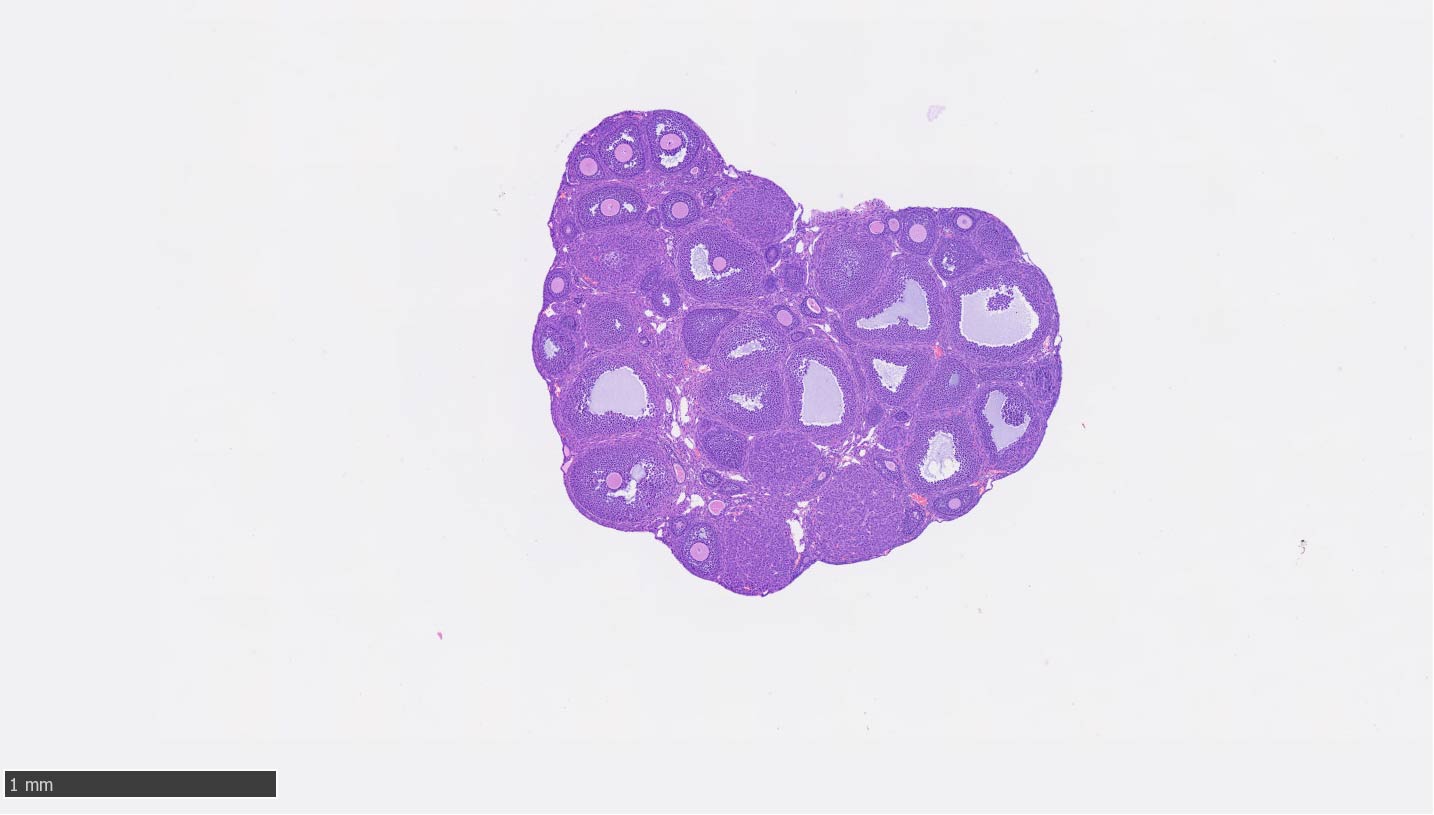

Supplement: Supplementary file 12 — Source data Fig. 1 [file 44319_2026_712_MOESM12_ESM.zip › Figure1 Source Data/1D/mKO_HE.jpg]

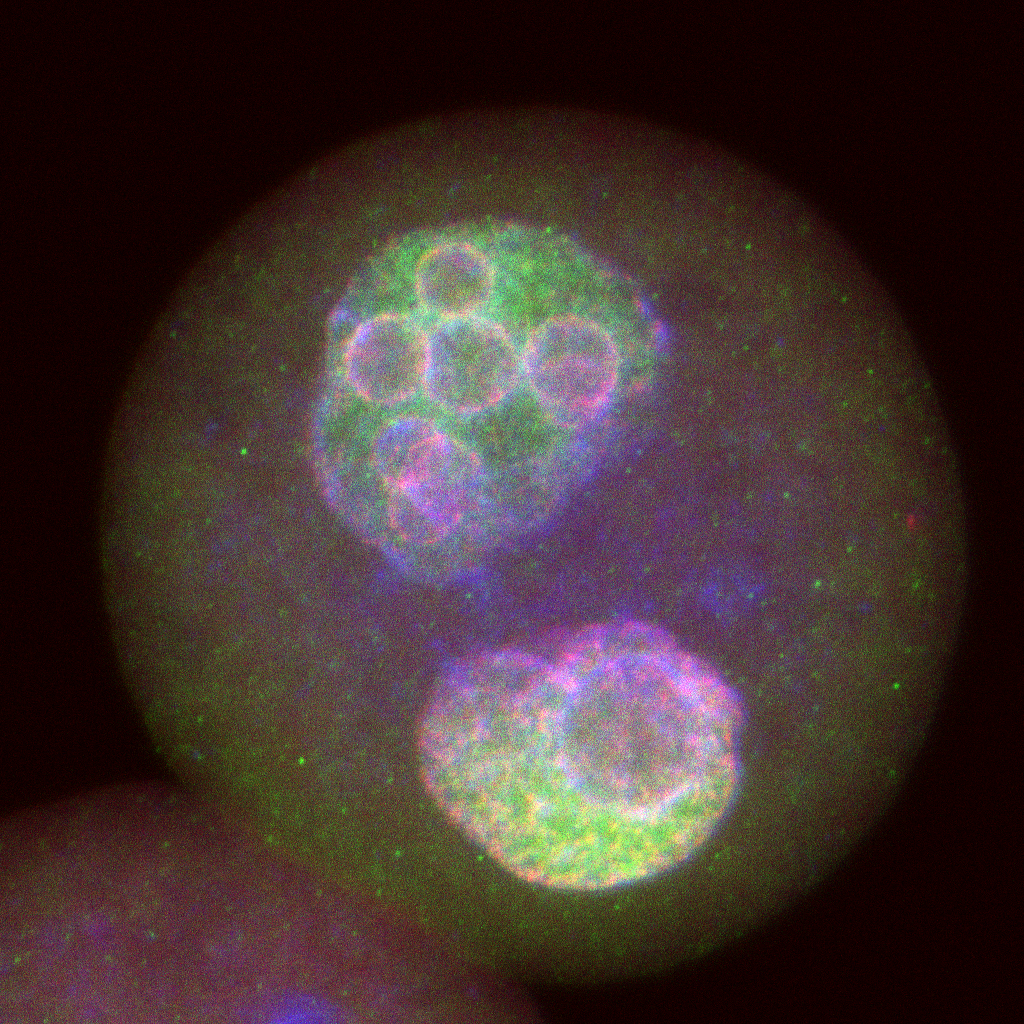

Supplement: Supplementary file 13 — Source data Fig. 2 [file 44319_2026_712_MOESM13_ESM.zip › Figure2 Source Data/2F/mKO_PN4_Merge.tif]

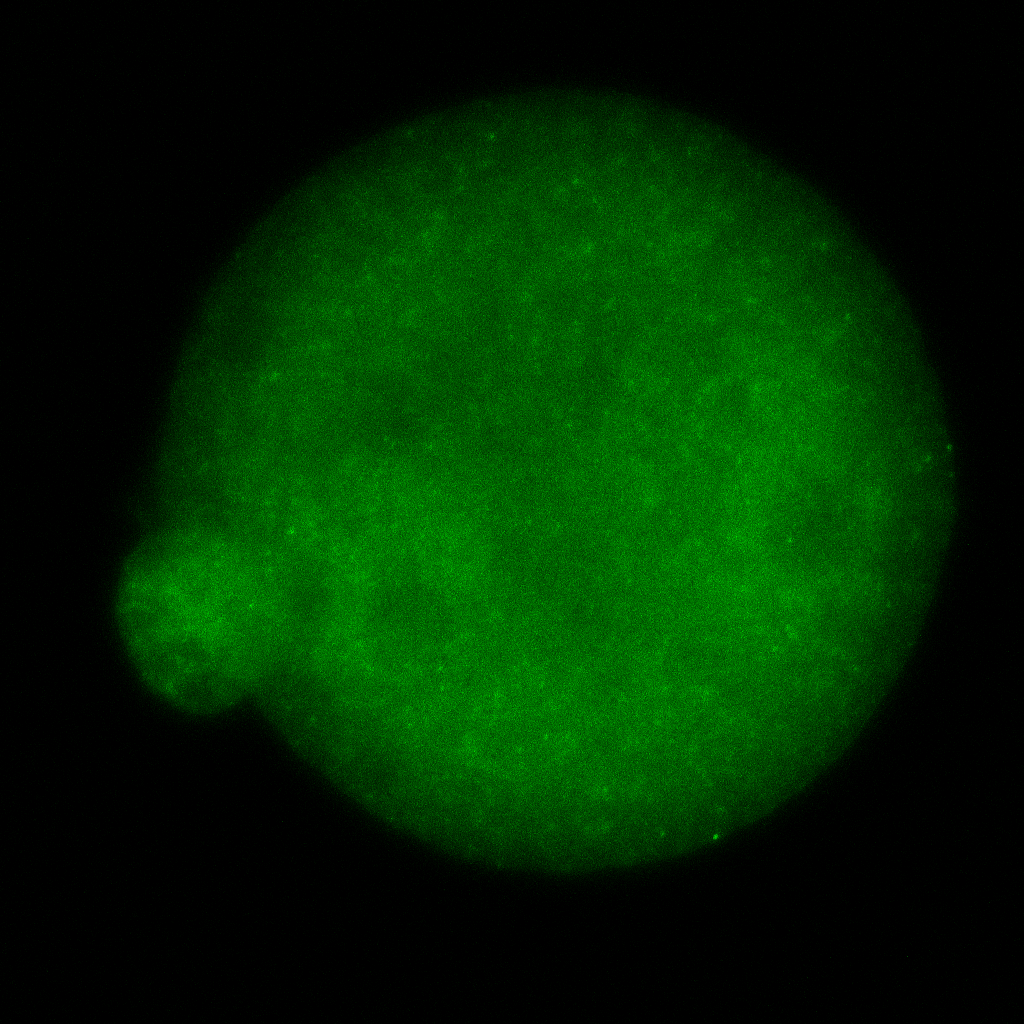

Supplement: Supplementary file 13 — Source data Fig. 2 [file 44319_2026_712_MOESM13_ESM.zip › Figure2 Source Data/2F/Ctrl_PN4_pCHK1.tif]

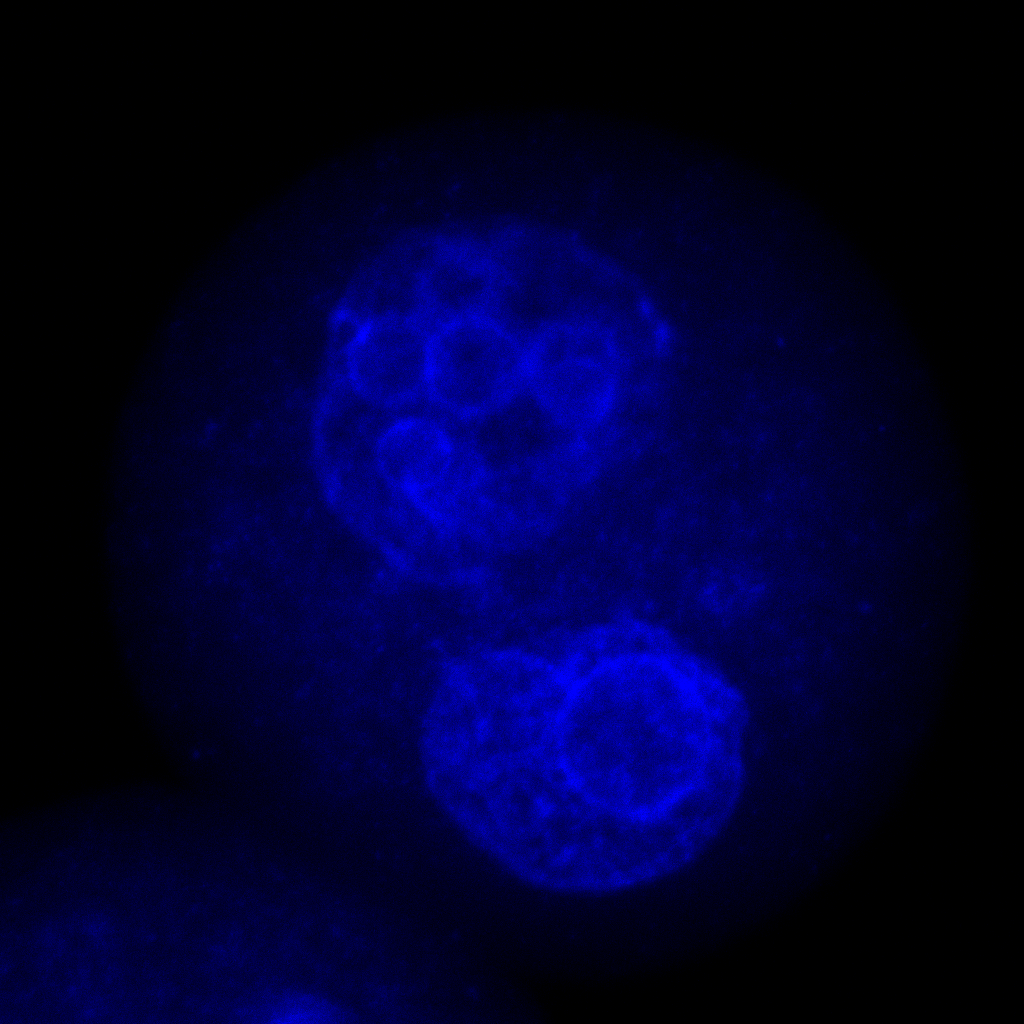

Supplement: Supplementary file 13 — Source data Fig. 2 [file 44319_2026_712_MOESM13_ESM.zip › Figure2 Source Data/2F/mKO_PN4_DAPI.tif]

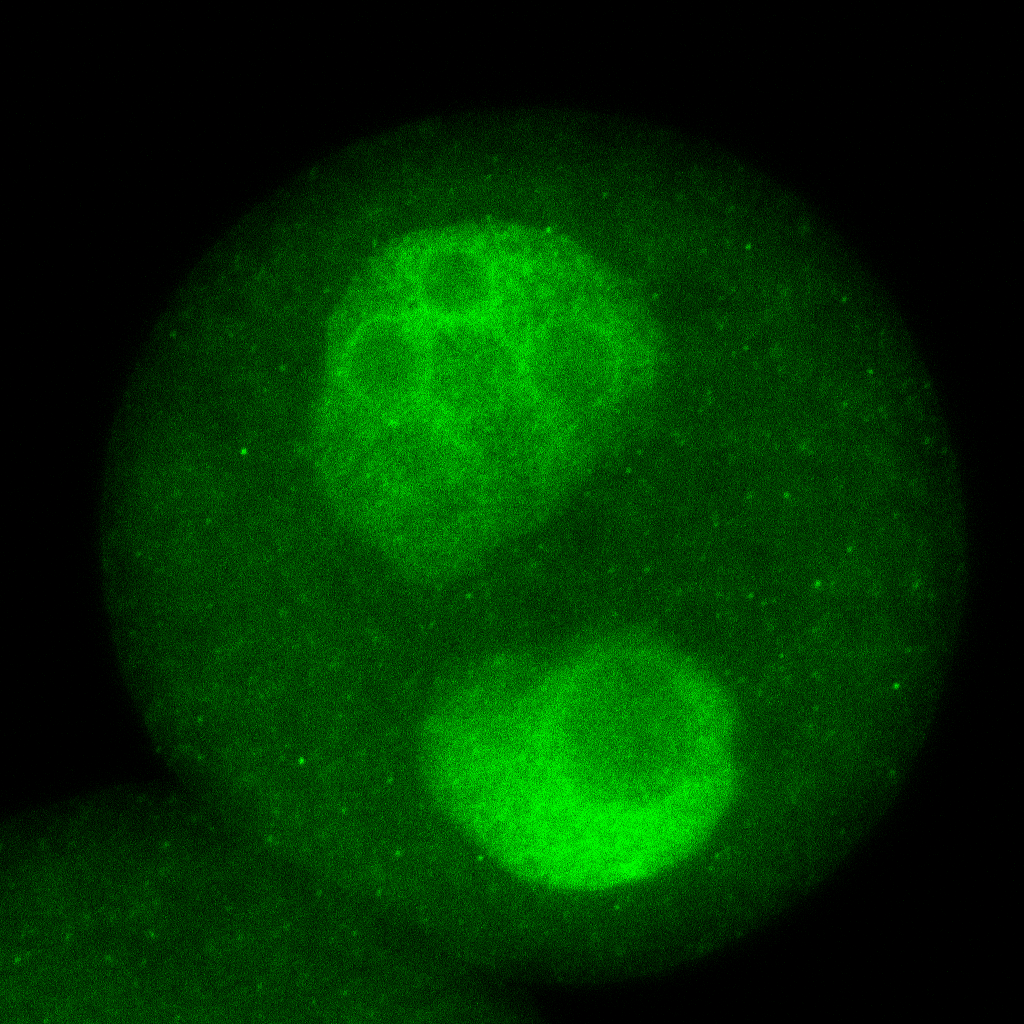

Supplement: Supplementary file 13 — Source data Fig. 2 [file 44319_2026_712_MOESM13_ESM.zip › Figure2 Source Data/2F/mKO_PN4_pCHK1.tif]

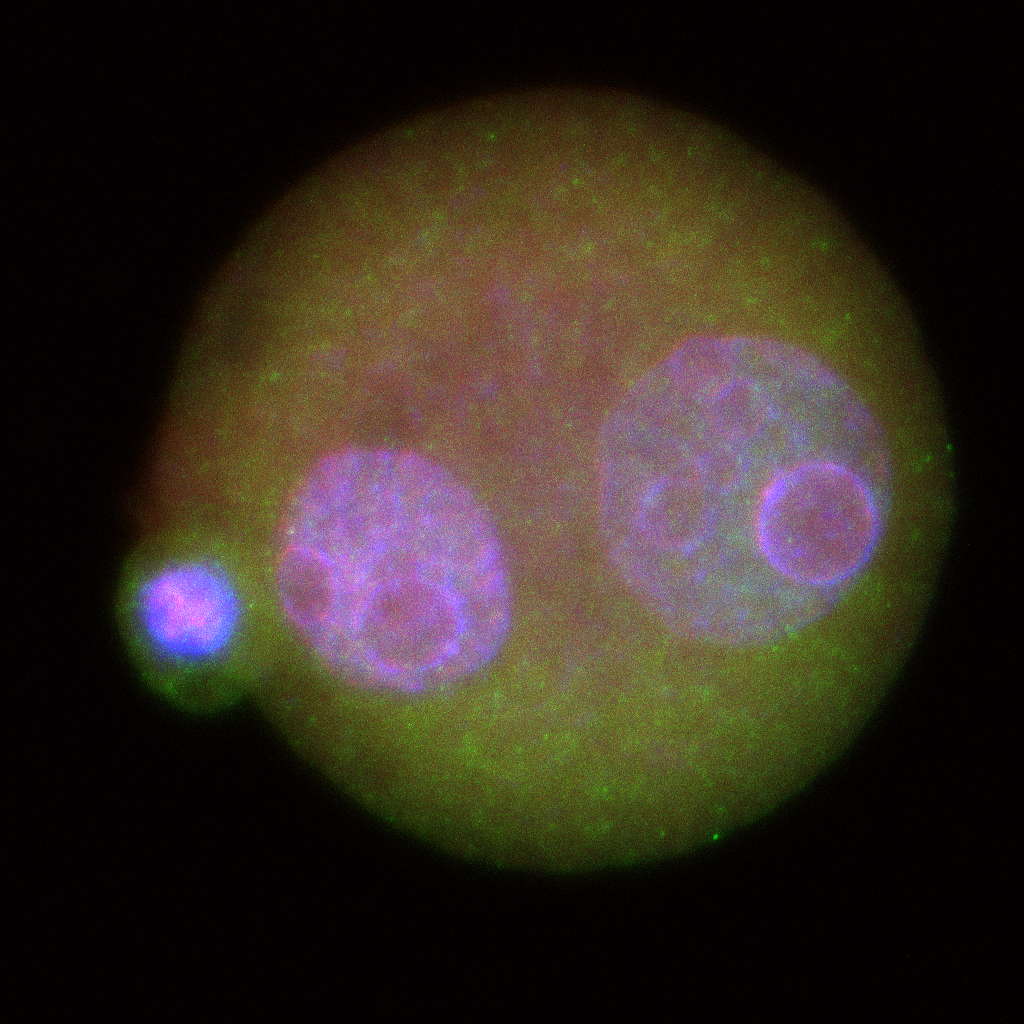

Supplement: Supplementary file 13 — Source data Fig. 2 [file 44319_2026_712_MOESM13_ESM.zip › Figure2 Source Data/2F/Ctrl_PN4_Merge.tif]

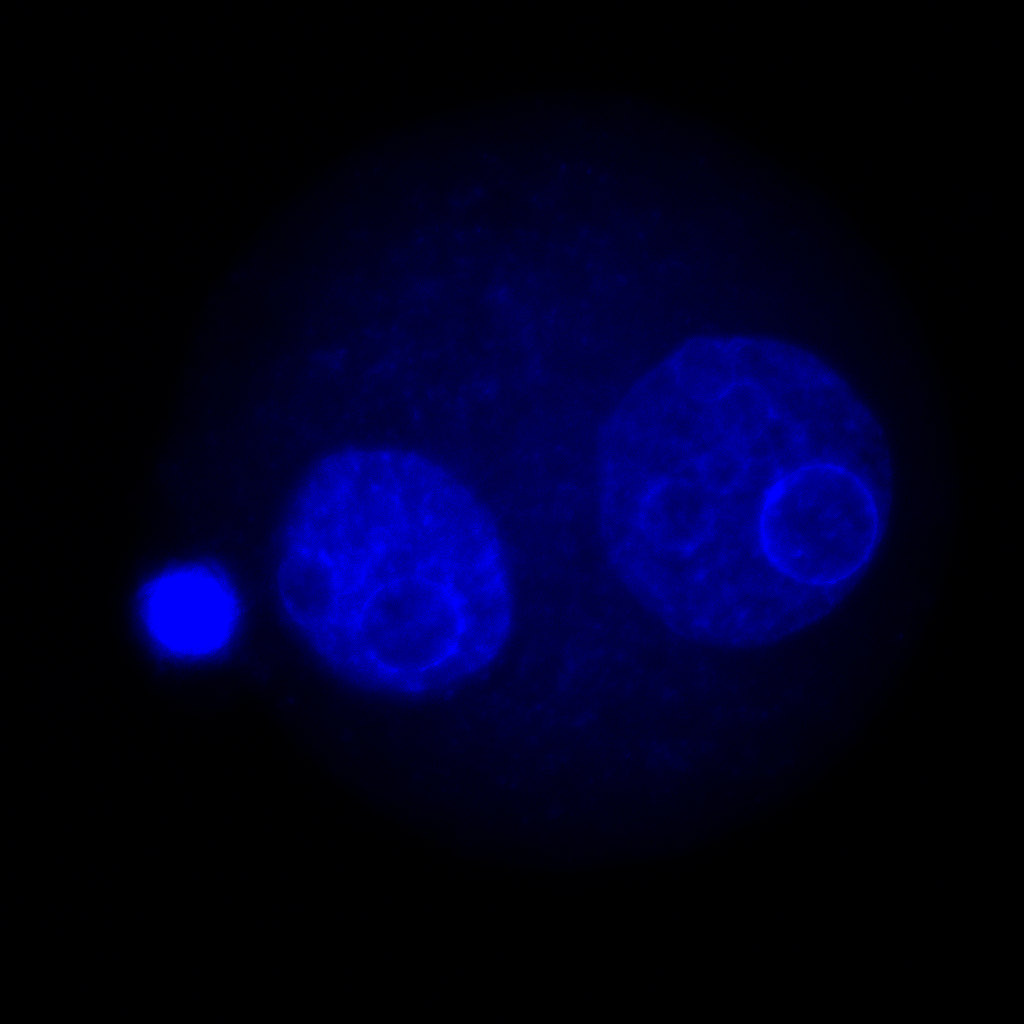

Supplement: Supplementary file 13 — Source data Fig. 2 [file 44319_2026_712_MOESM13_ESM.zip › Figure2 Source Data/2F/Ctrl_PN4_DAPI.tif]

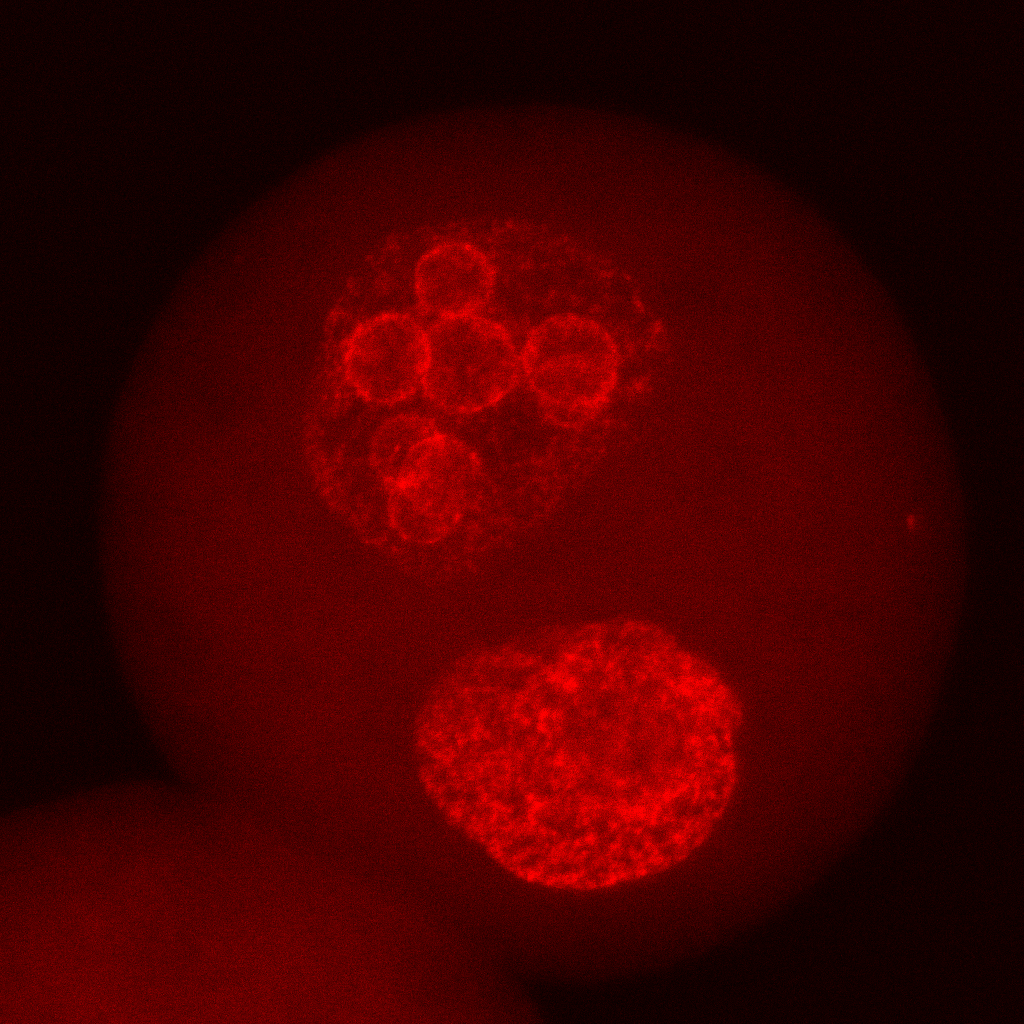

Supplement: Supplementary file 13 — Source data Fig. 2 [file 44319_2026_712_MOESM13_ESM.zip › Figure2 Source Data/2F/mKO_PN4_EdU.tif]

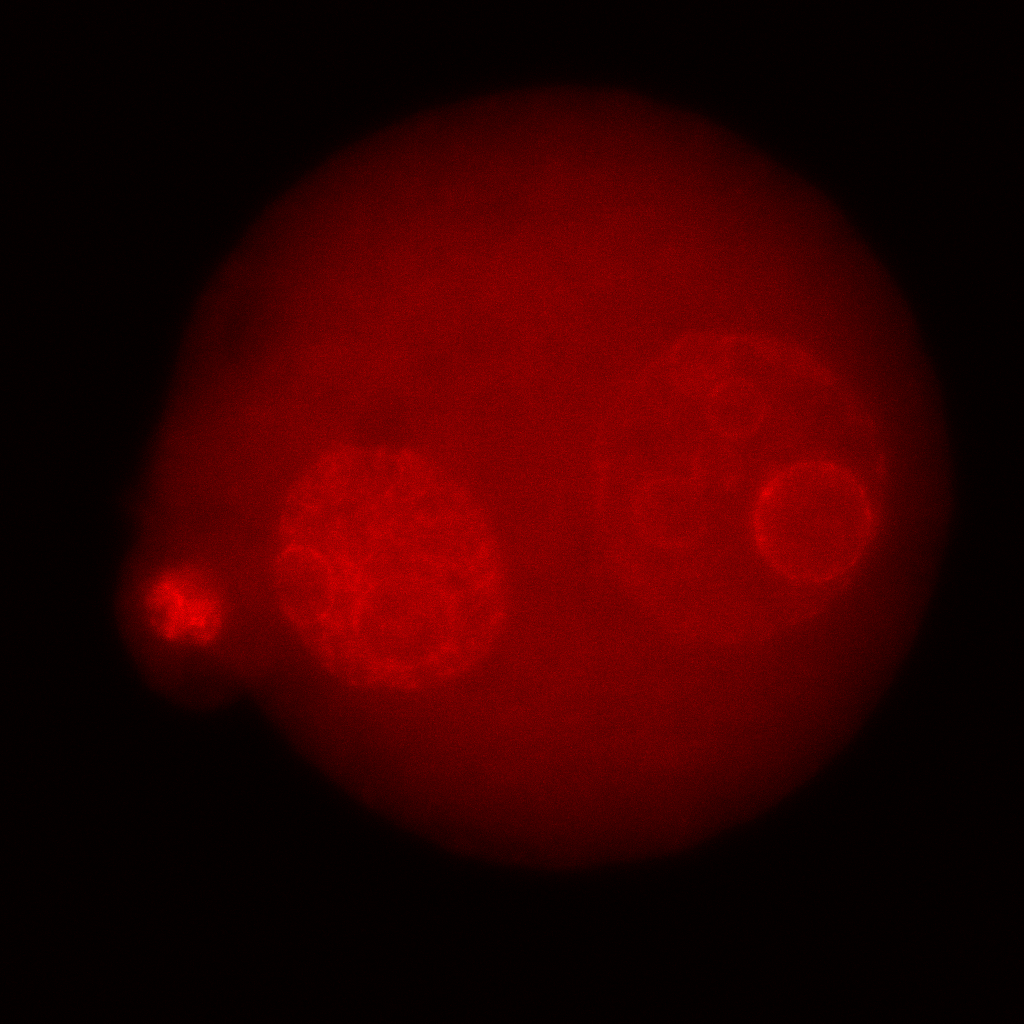

Supplement: Supplementary file 13 — Source data Fig. 2 [file 44319_2026_712_MOESM13_ESM.zip › Figure2 Source Data/2F/Ctrl_PN4_EdU.tif]

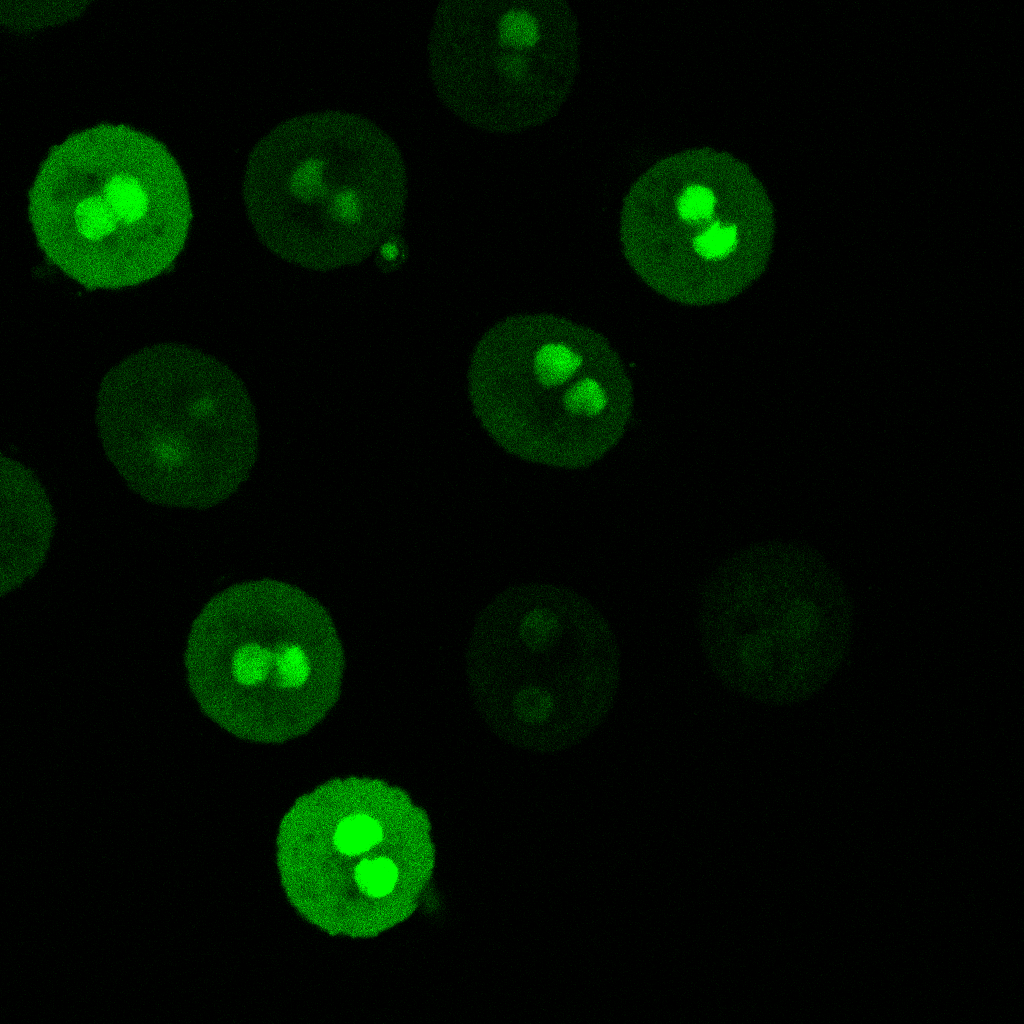

Supplement: Supplementary file 13 — Source data Fig. 2 [file 44319_2026_712_MOESM13_ESM.zip › Figure2 Source Data/2C/Ctrl.hCG32h.mVenus.tif]

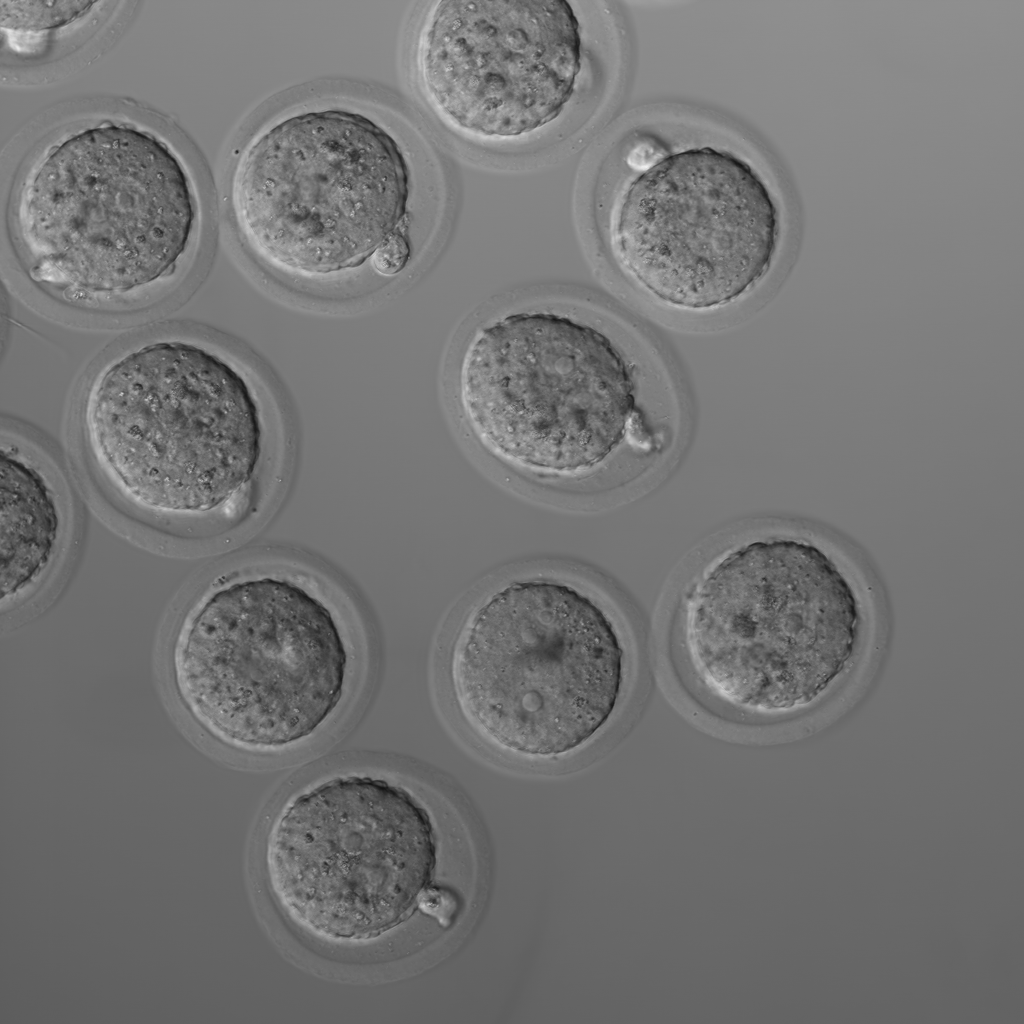

Supplement: Supplementary file 13 — Source data Fig. 2 [file 44319_2026_712_MOESM13_ESM.zip › Figure2 Source Data/2C/Ctrl.hCG32h.bright field.tif]

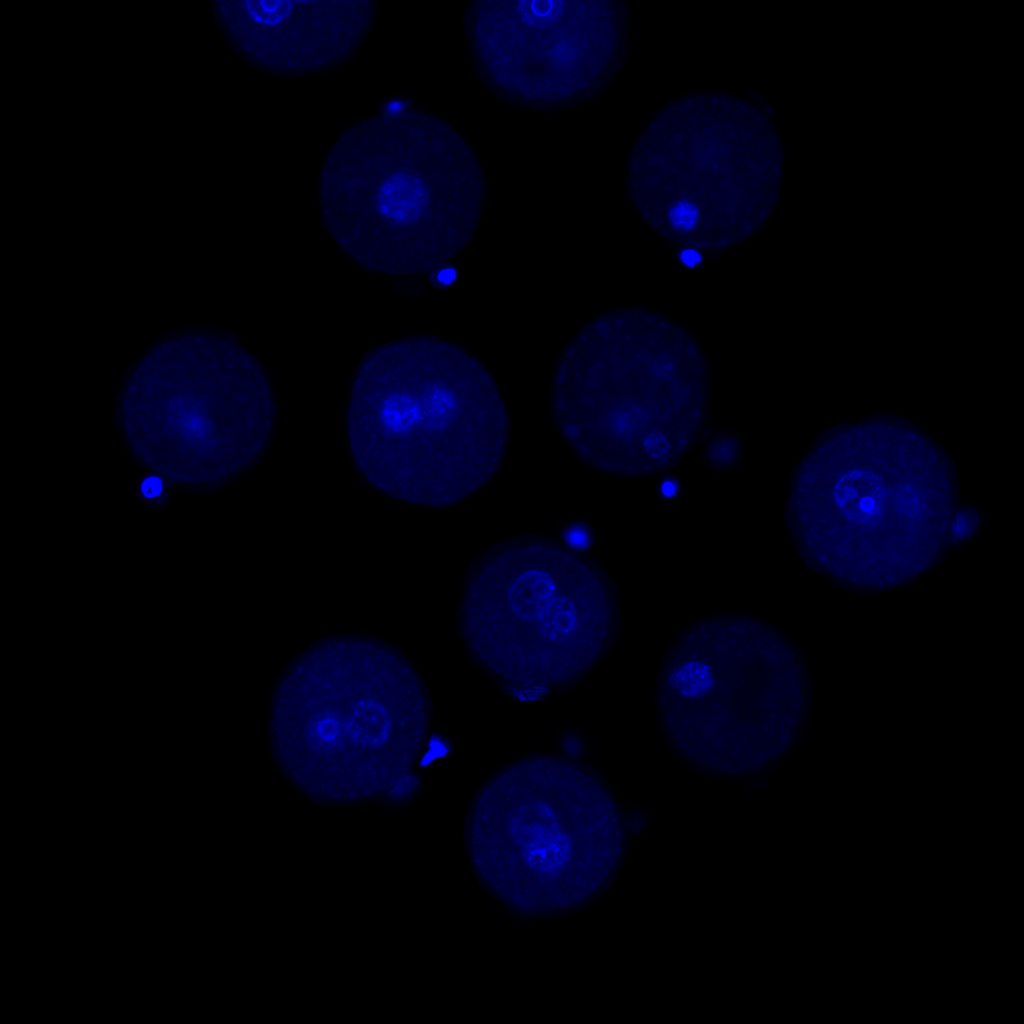

Supplement: Supplementary file 13 — Source data Fig. 2 [file 44319_2026_712_MOESM13_ESM.zip › Figure2 Source Data/2C/mKO.hCG32h.DAPI.tif]

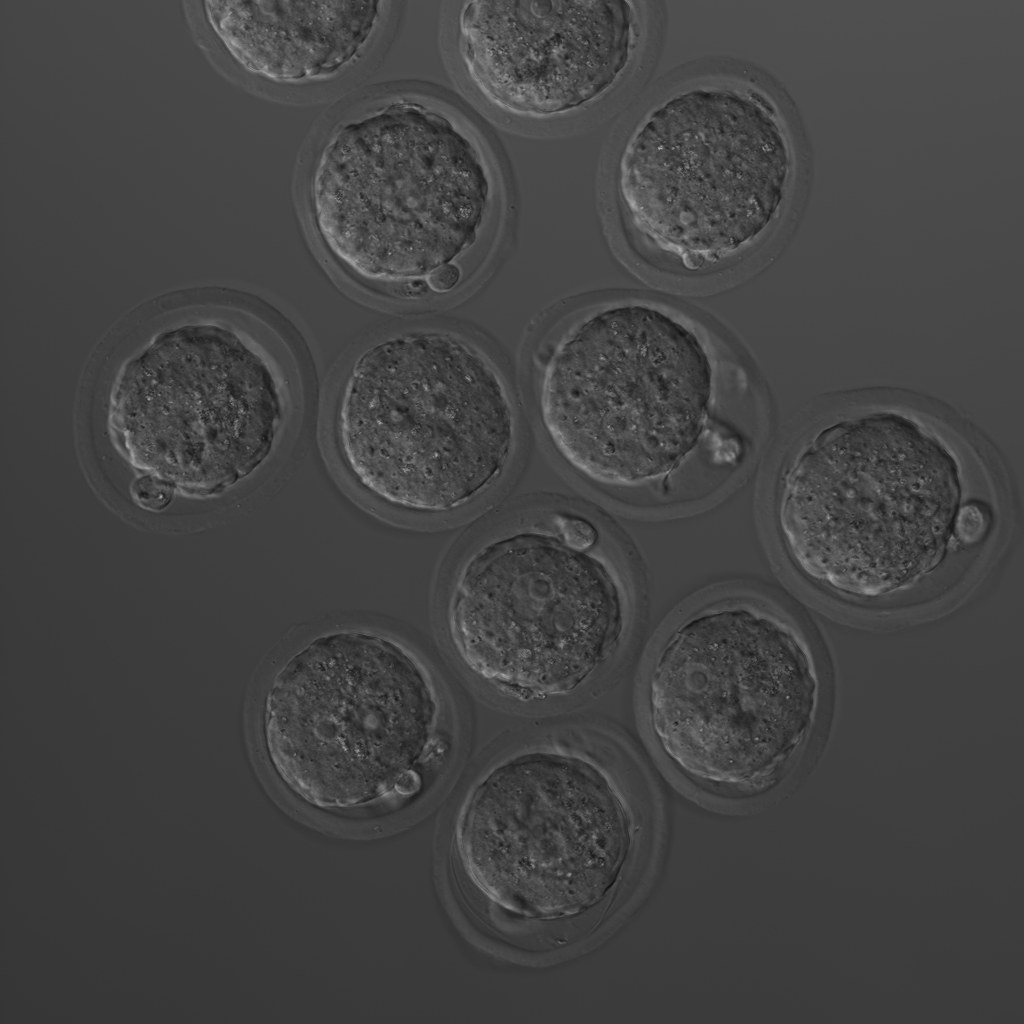

Supplement: Supplementary file 13 — Source data Fig. 2 [file 44319_2026_712_MOESM13_ESM.zip › Figure2 Source Data/2C/mKO.hCG32h.bright field.tif]

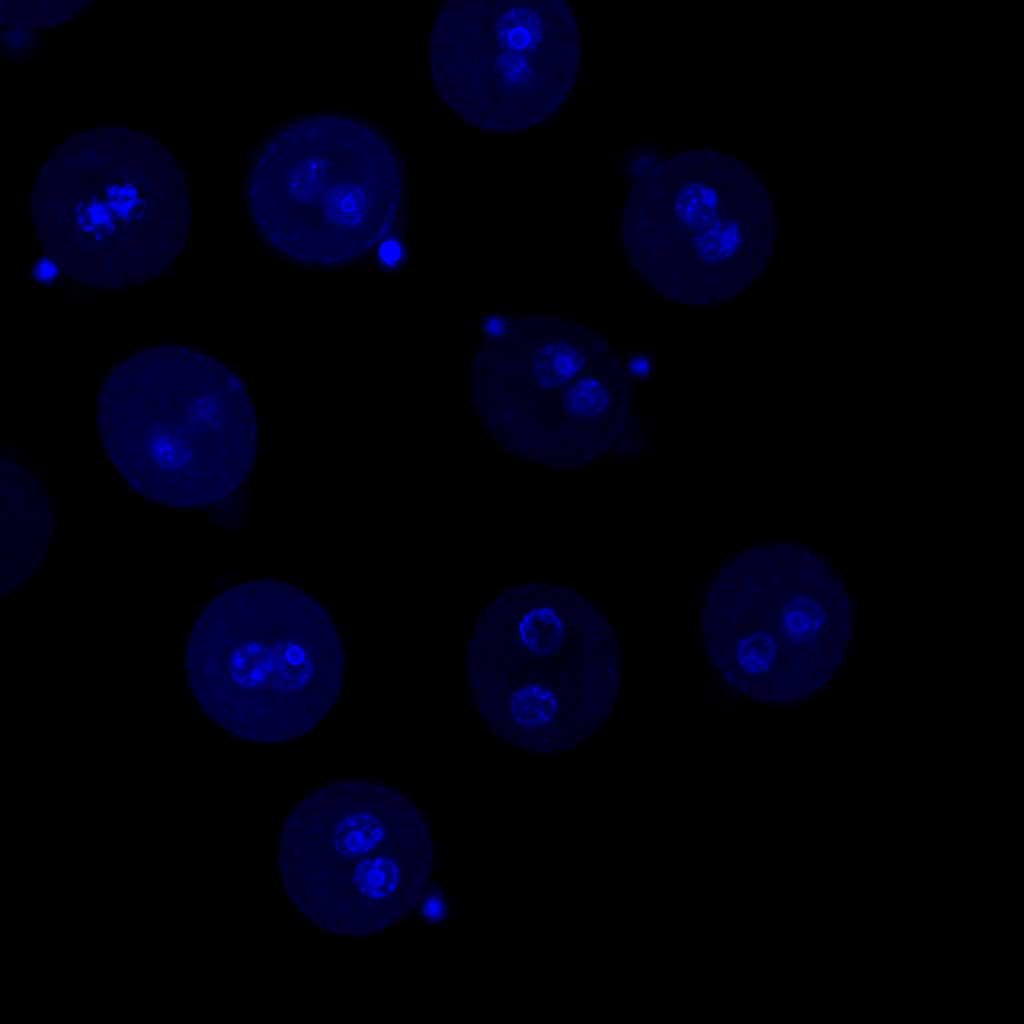

Supplement: Supplementary file 13 — Source data Fig. 2 [file 44319_2026_712_MOESM13_ESM.zip › Figure2 Source Data/2C/Ctrl.hCG32h.DAPI.tif]

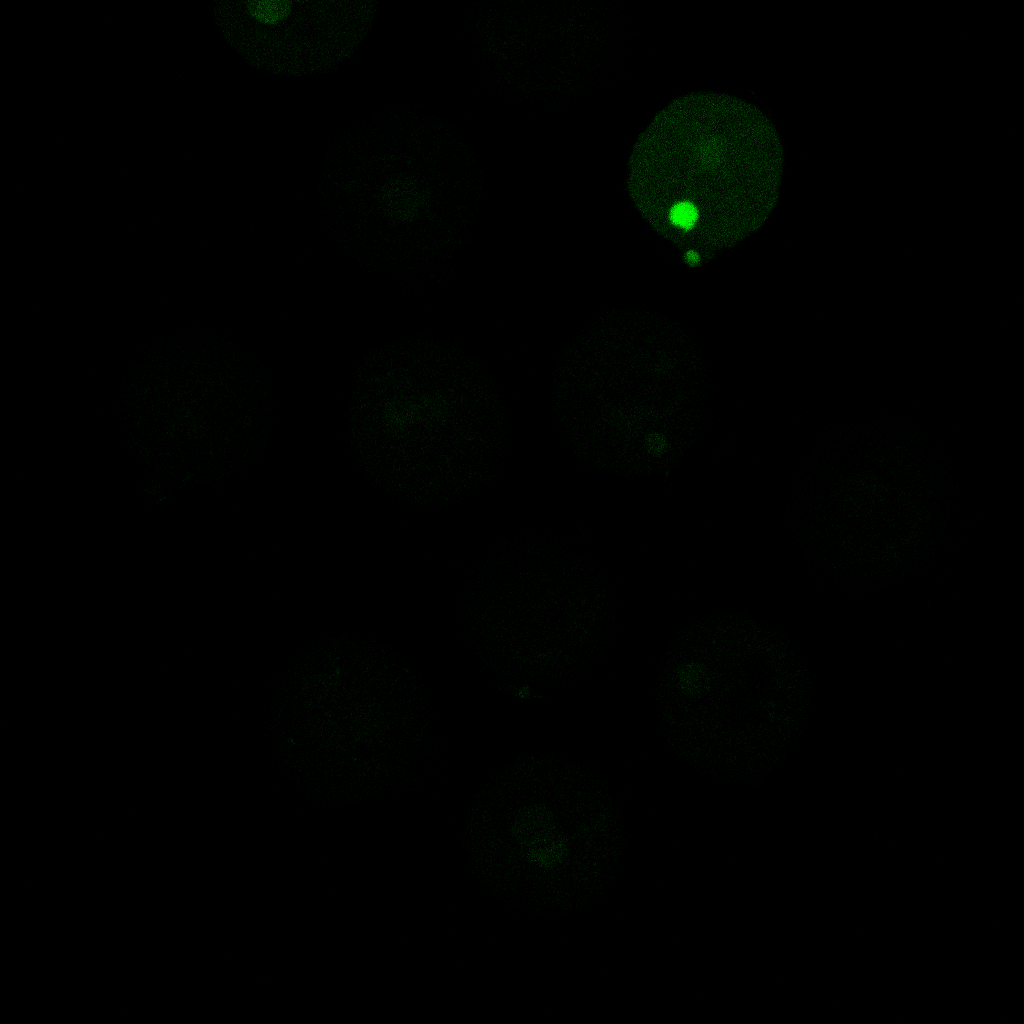

Supplement: Supplementary file 13 — Source data Fig. 2 [file 44319_2026_712_MOESM13_ESM.zip › Figure2 Source Data/2C/mKO.hCG32h.mVenus.tif]

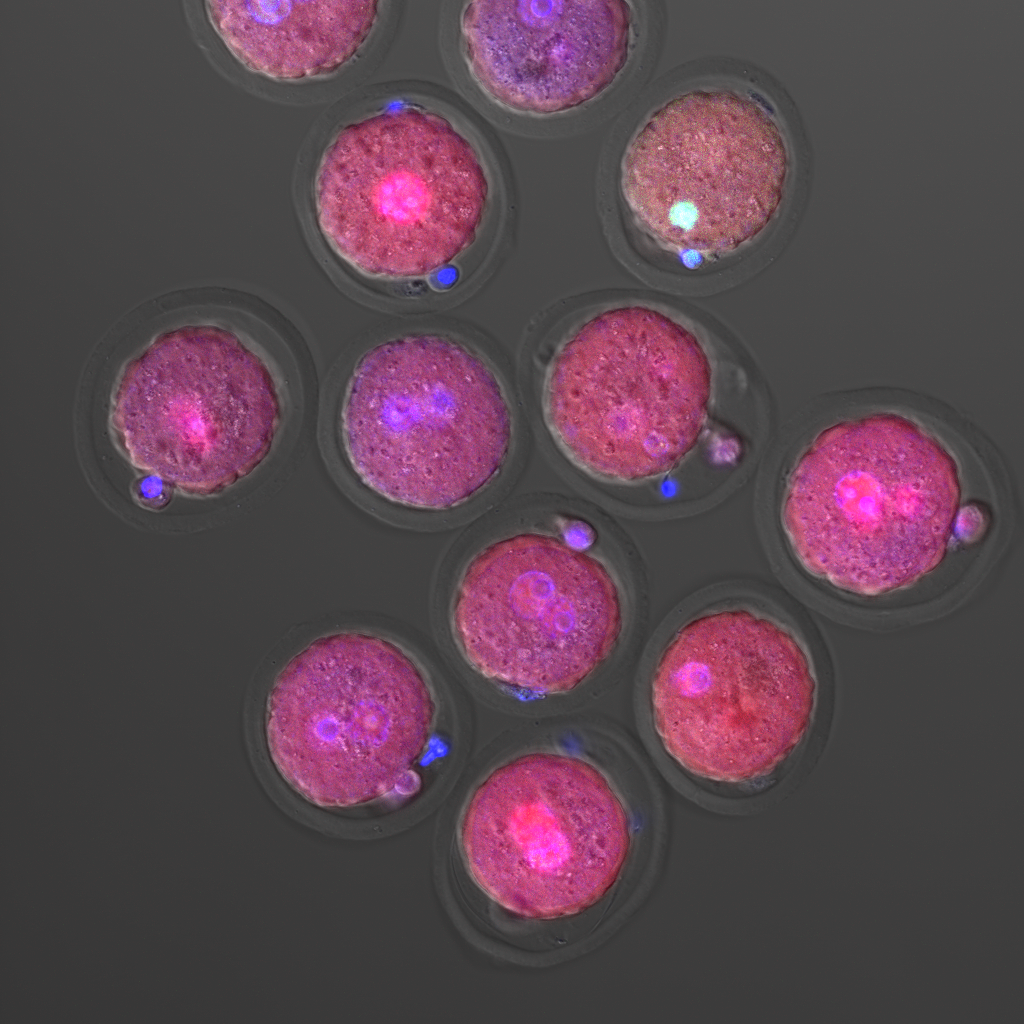

Supplement: Supplementary file 13 — Source data Fig. 2 [file 44319_2026_712_MOESM13_ESM.zip › Figure2 Source Data/2C/mKO.hCG32h.Merge.tif]

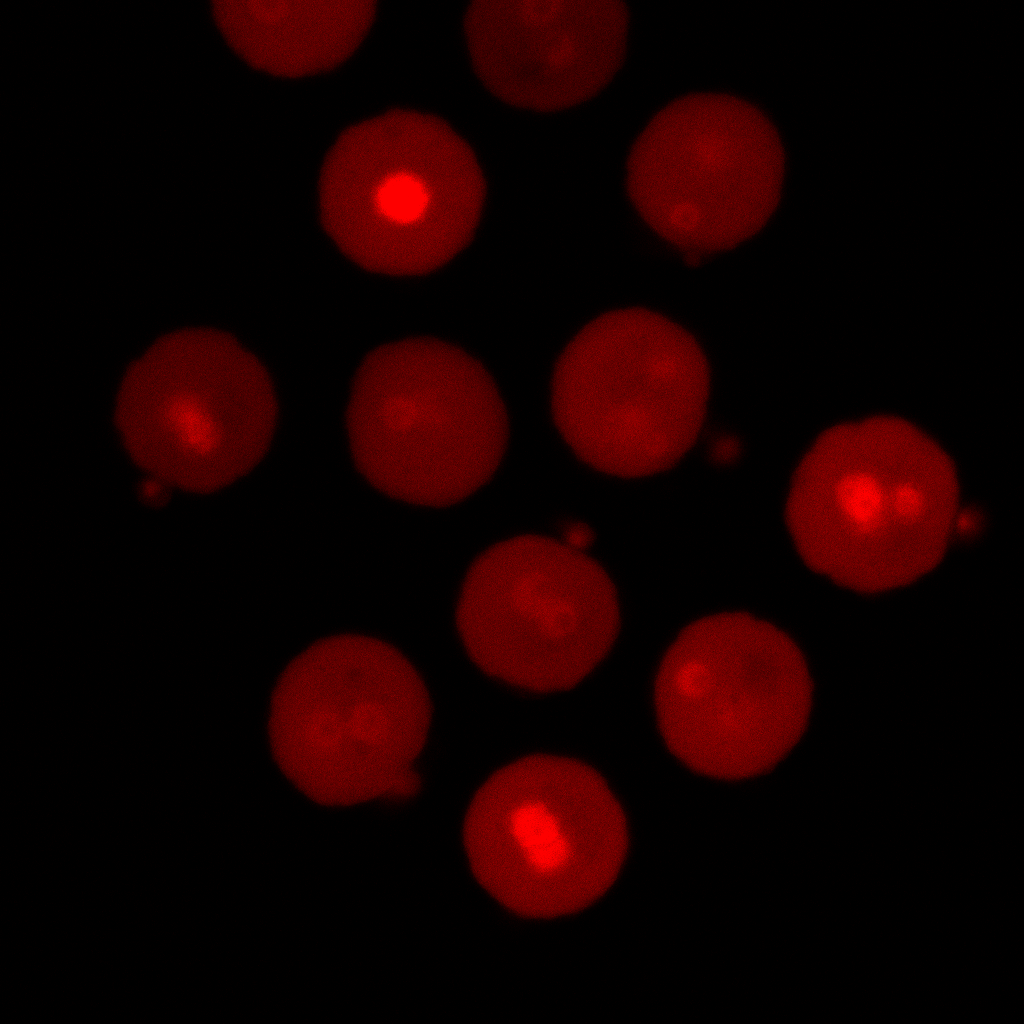

Supplement: Supplementary file 13 — Source data Fig. 2 [file 44319_2026_712_MOESM13_ESM.zip › Figure2 Source Data/2C/mKO.hCG32h.mCherry.tif]

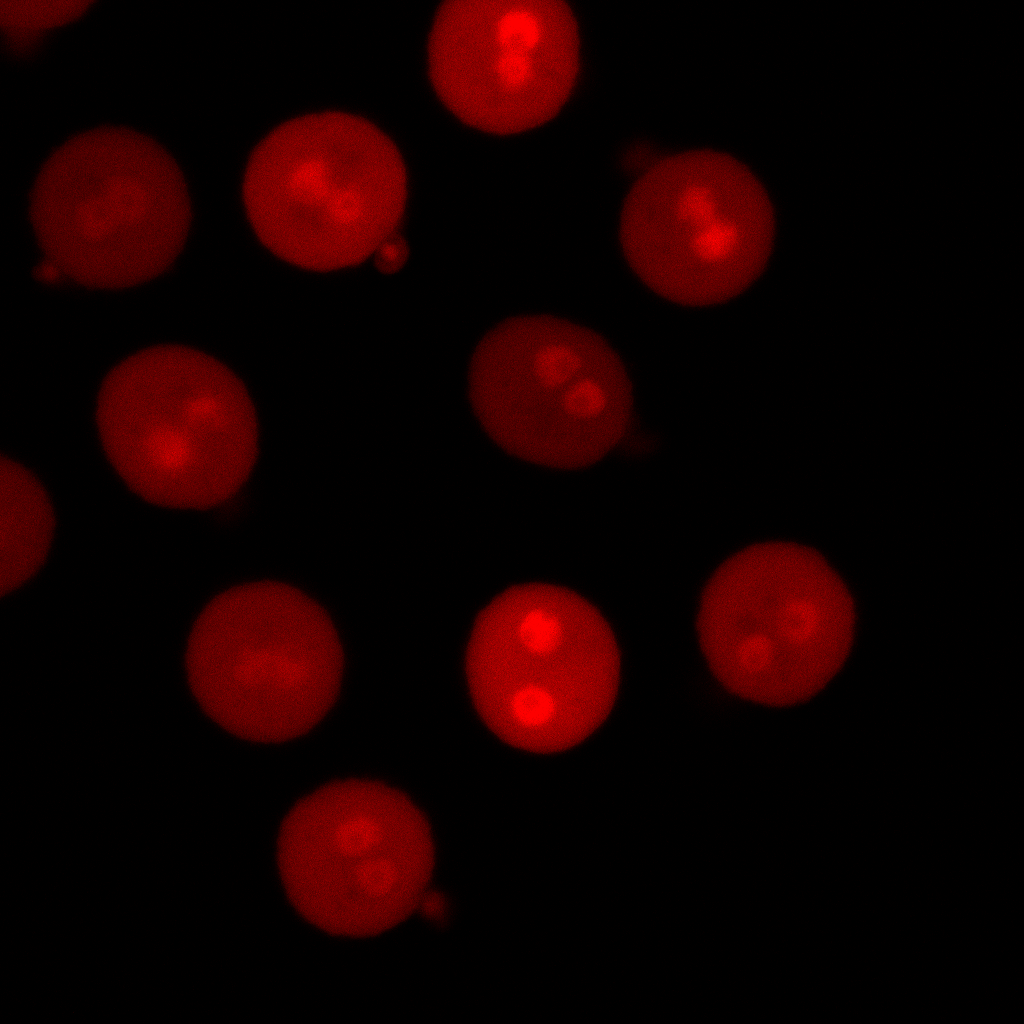

Supplement: Supplementary file 13 — Source data Fig. 2 [file 44319_2026_712_MOESM13_ESM.zip › Figure2 Source Data/2C/Ctrl.hCG32h.mCherry.tif]

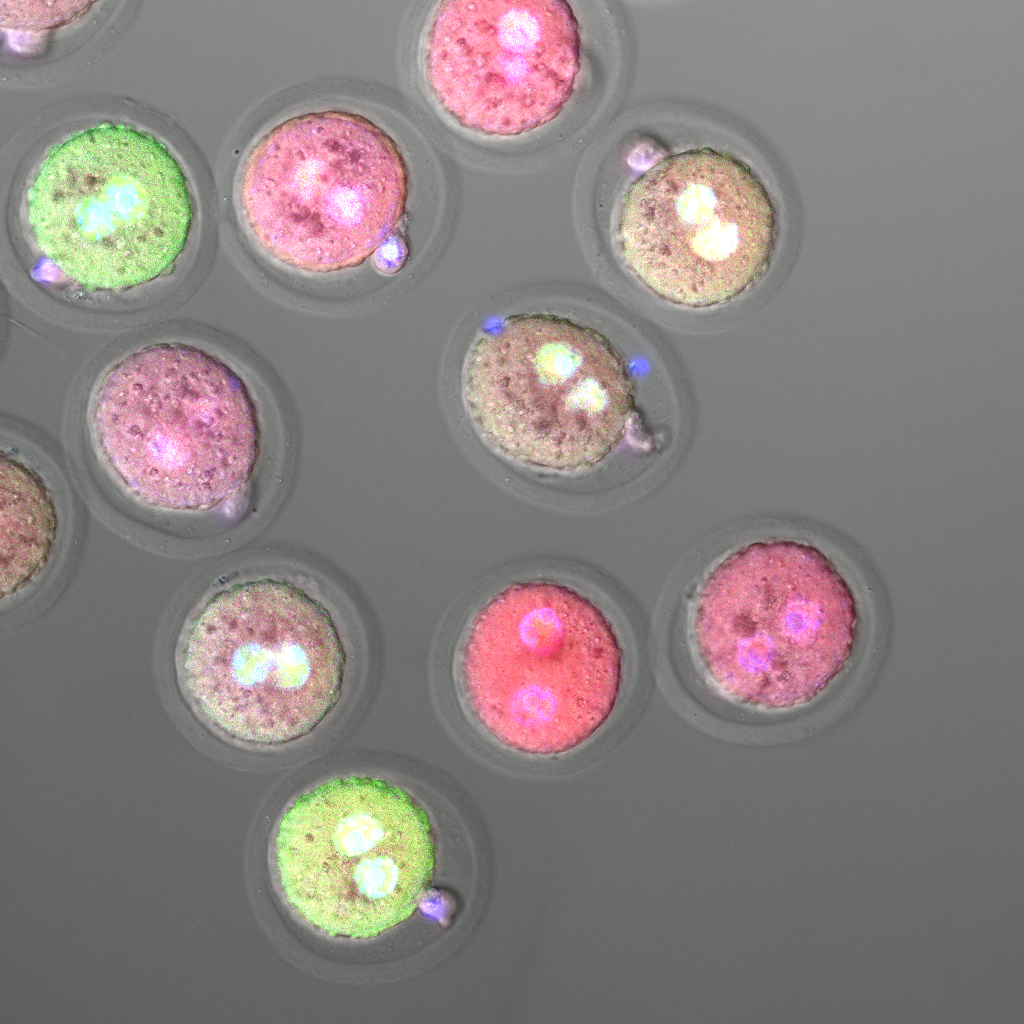

Supplement: Supplementary file 13 — Source data Fig. 2 [file 44319_2026_712_MOESM13_ESM.zip › Figure2 Source Data/2C/Ctrl.hCG32h.merge.tif]

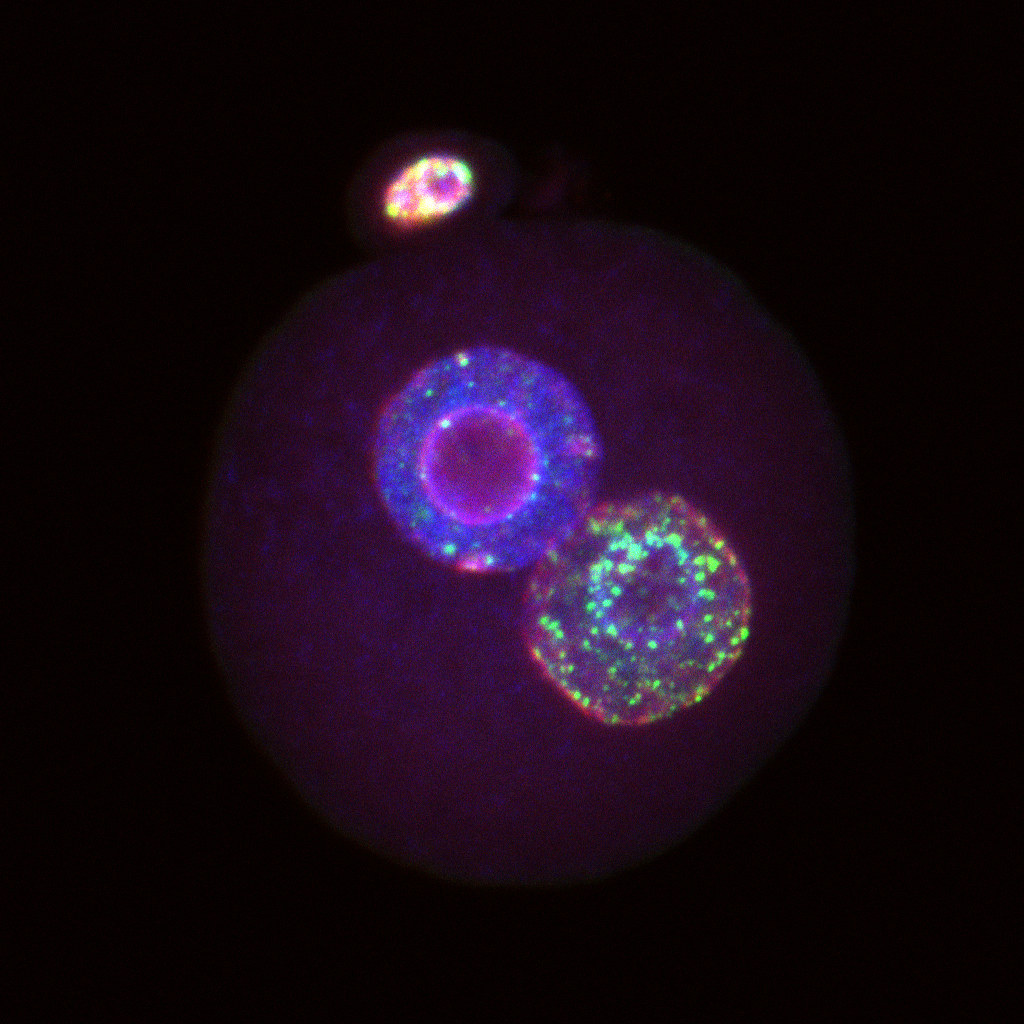

Supplement: Supplementary file 13 — Source data Fig. 2 [file 44319_2026_712_MOESM13_ESM.zip › Figure2 Source Data/2E/Ctrl_hCG27h_Merge.tif]

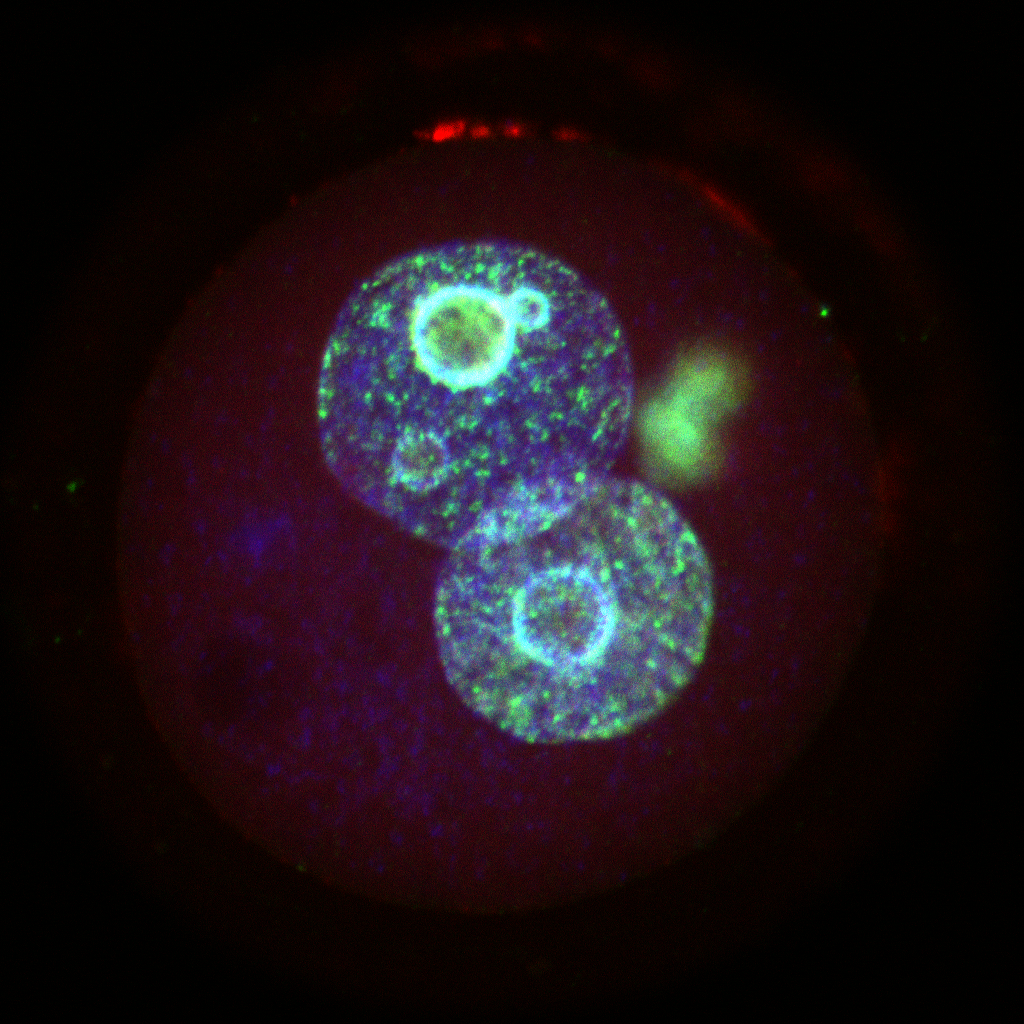

Supplement: Supplementary file 13 — Source data Fig. 2 [file 44319_2026_712_MOESM13_ESM.zip › Figure2 Source Data/2E/mKO.hCG27h.Merge.tif]

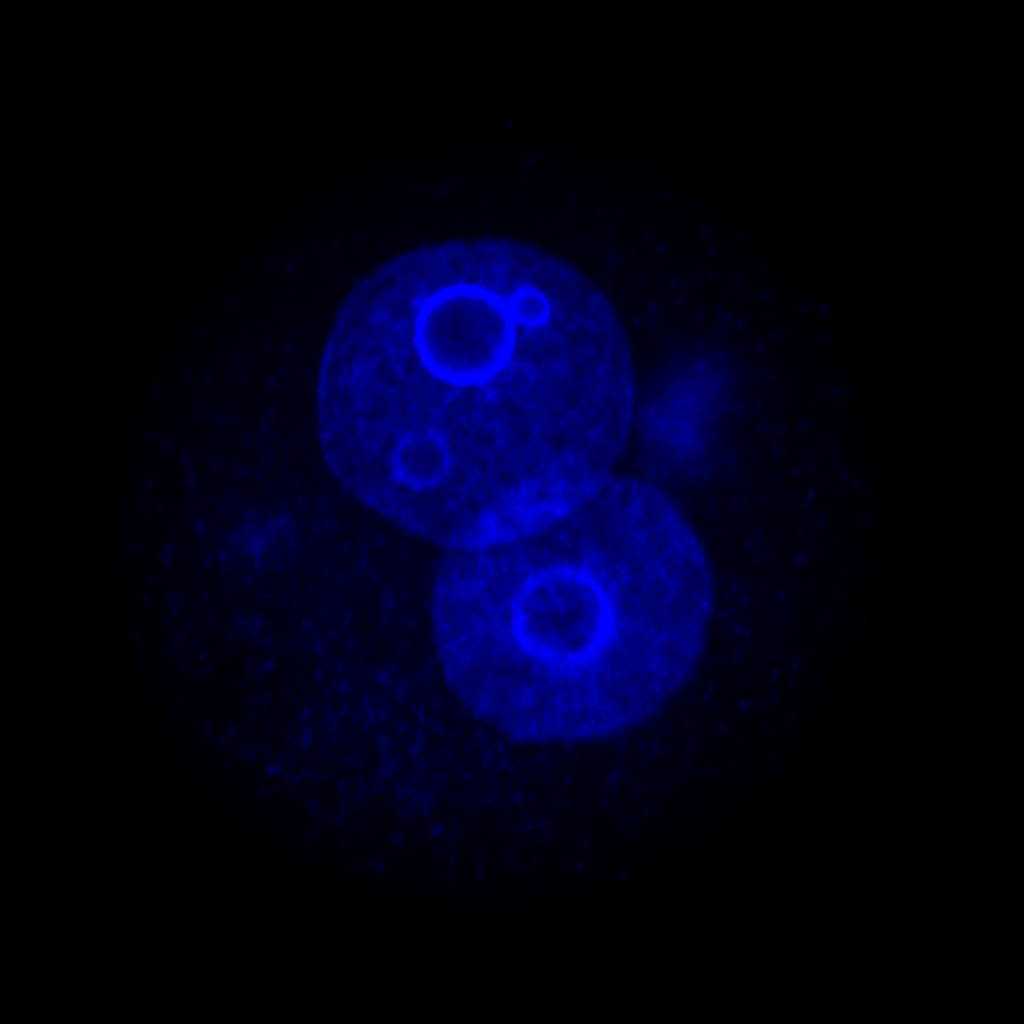

Supplement: Supplementary file 13 — Source data Fig. 2 [file 44319_2026_712_MOESM13_ESM.zip › Figure2 Source Data/2E/mKO.hCG27h.DAPI.tif]

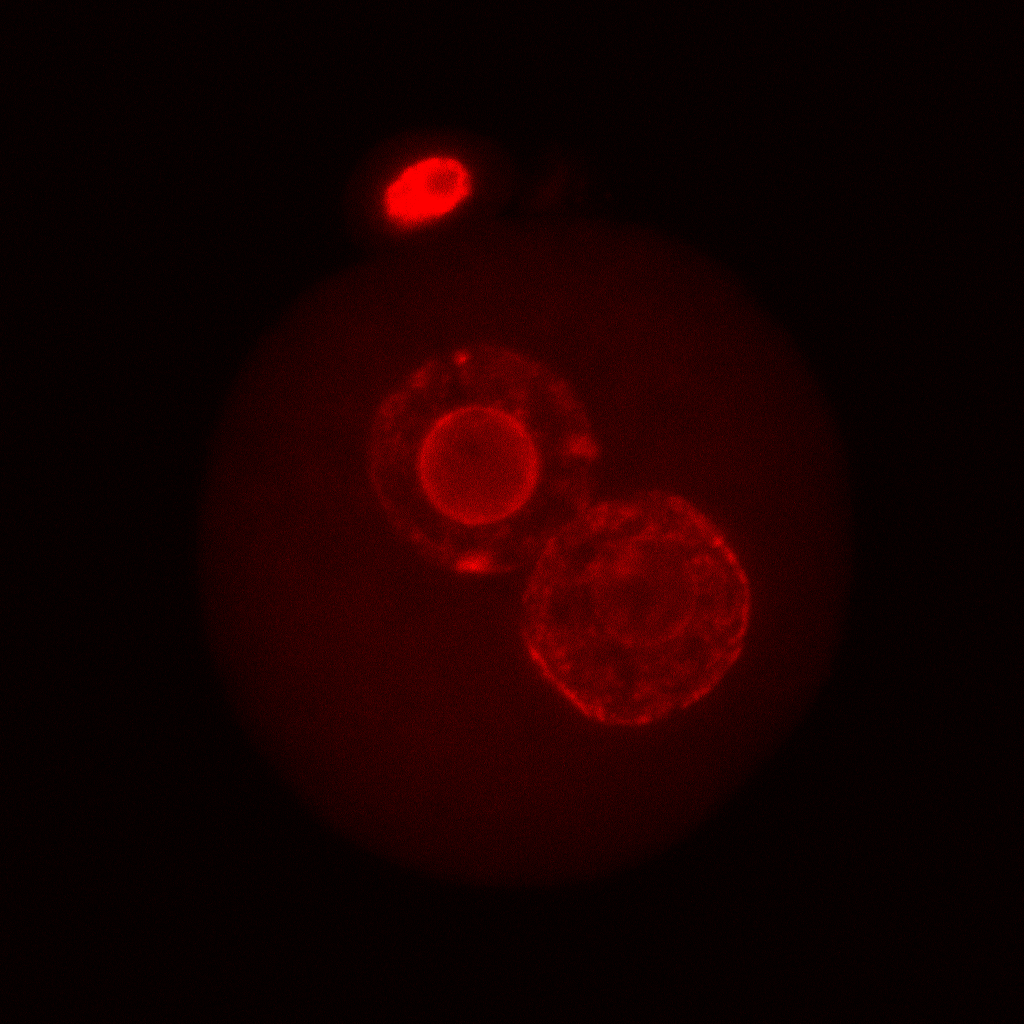

Supplement: Supplementary file 13 — Source data Fig. 2 [file 44319_2026_712_MOESM13_ESM.zip › Figure2 Source Data/2E/Ctrl_hCG27h_EdU.tif]

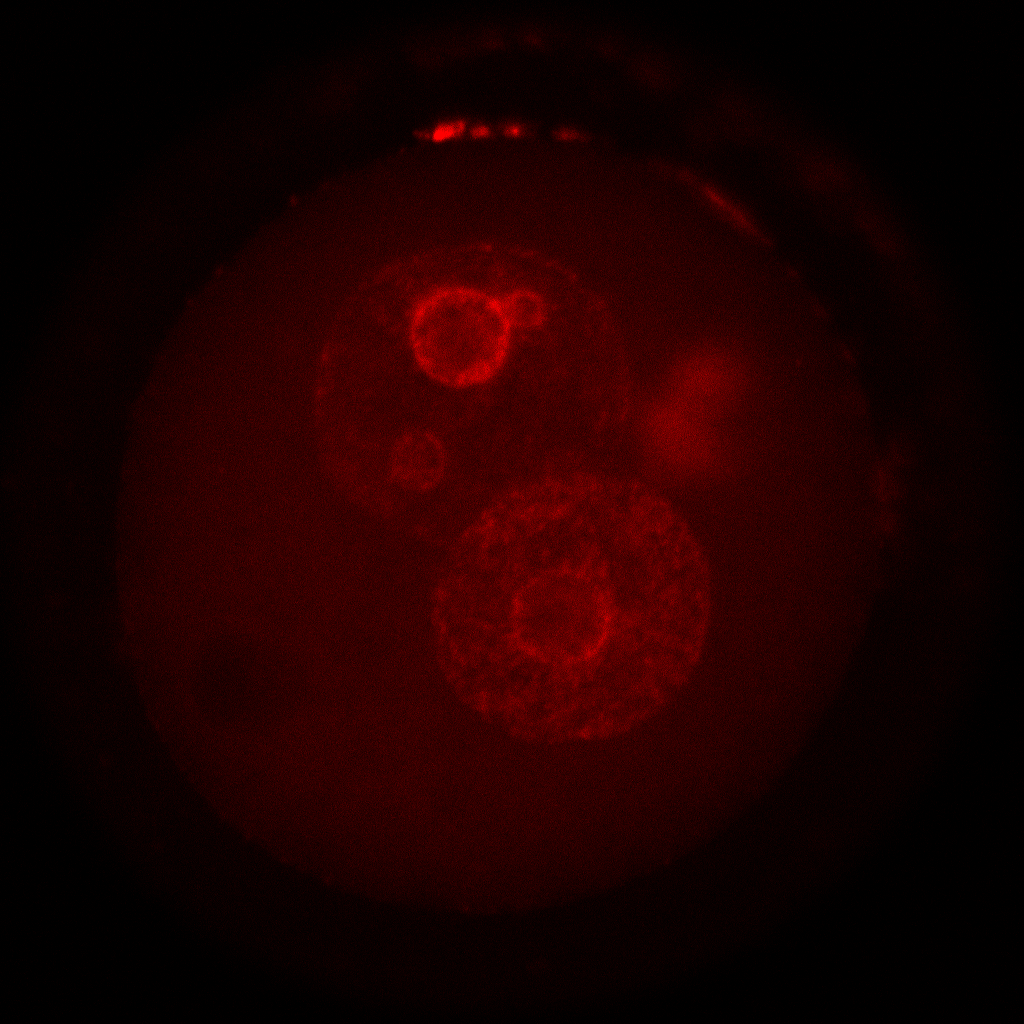

Supplement: Supplementary file 13 — Source data Fig. 2 [file 44319_2026_712_MOESM13_ESM.zip › Figure2 Source Data/2E/mKO.hCG27h.EdU.tif]

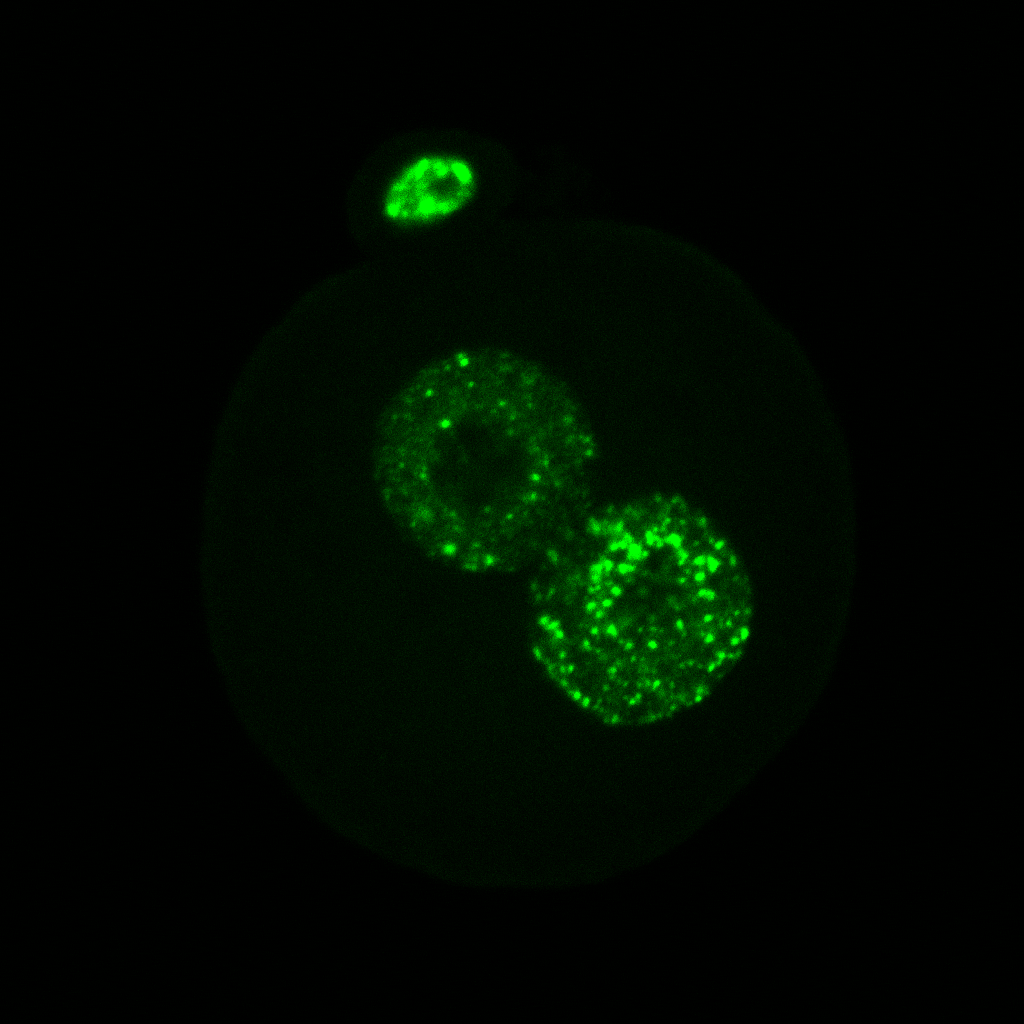

Supplement: Supplementary file 13 — Source data Fig. 2 [file 44319_2026_712_MOESM13_ESM.zip › Figure2 Source Data/2E/Ctrl_hCG27h_╬│H2AX.tif]

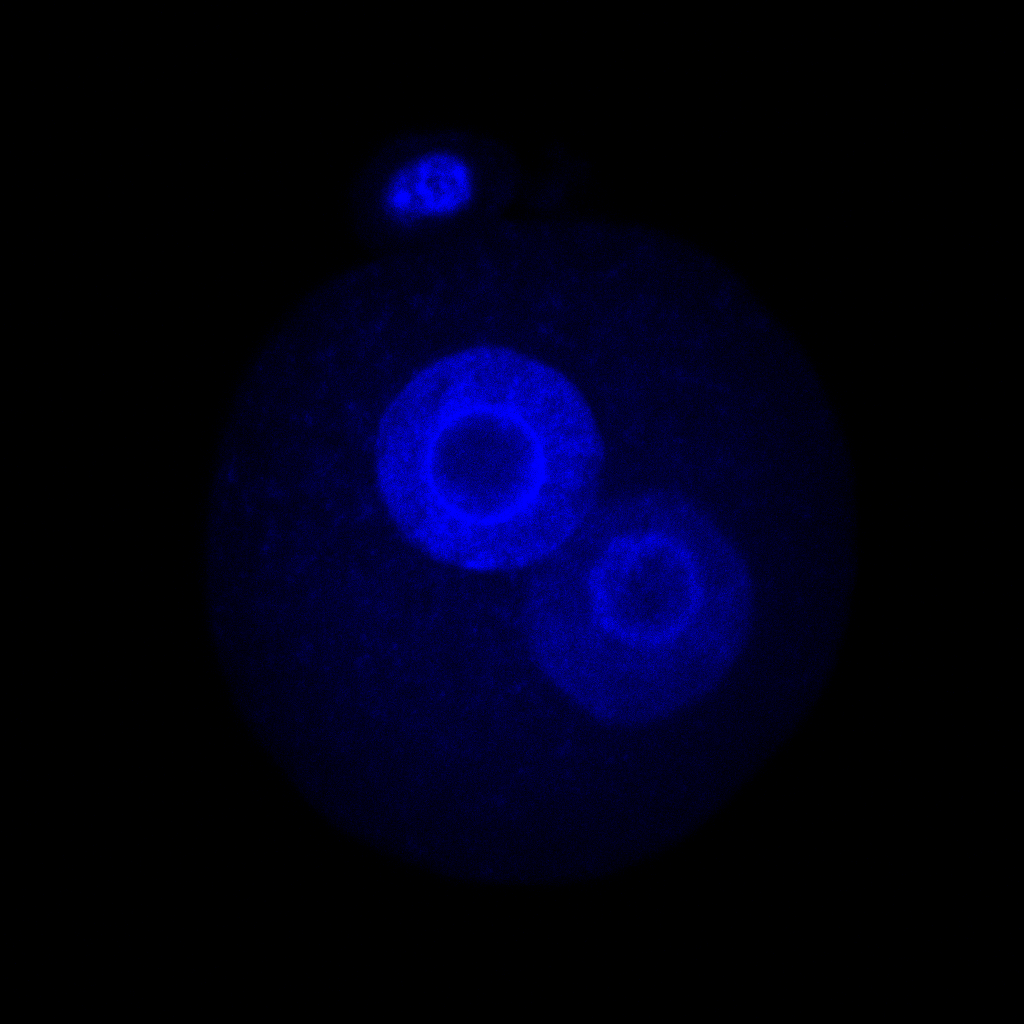

Supplement: Supplementary file 13 — Source data Fig. 2 [file 44319_2026_712_MOESM13_ESM.zip › Figure2 Source Data/2E/Ctrl_hCG27h_DAPI.tif]

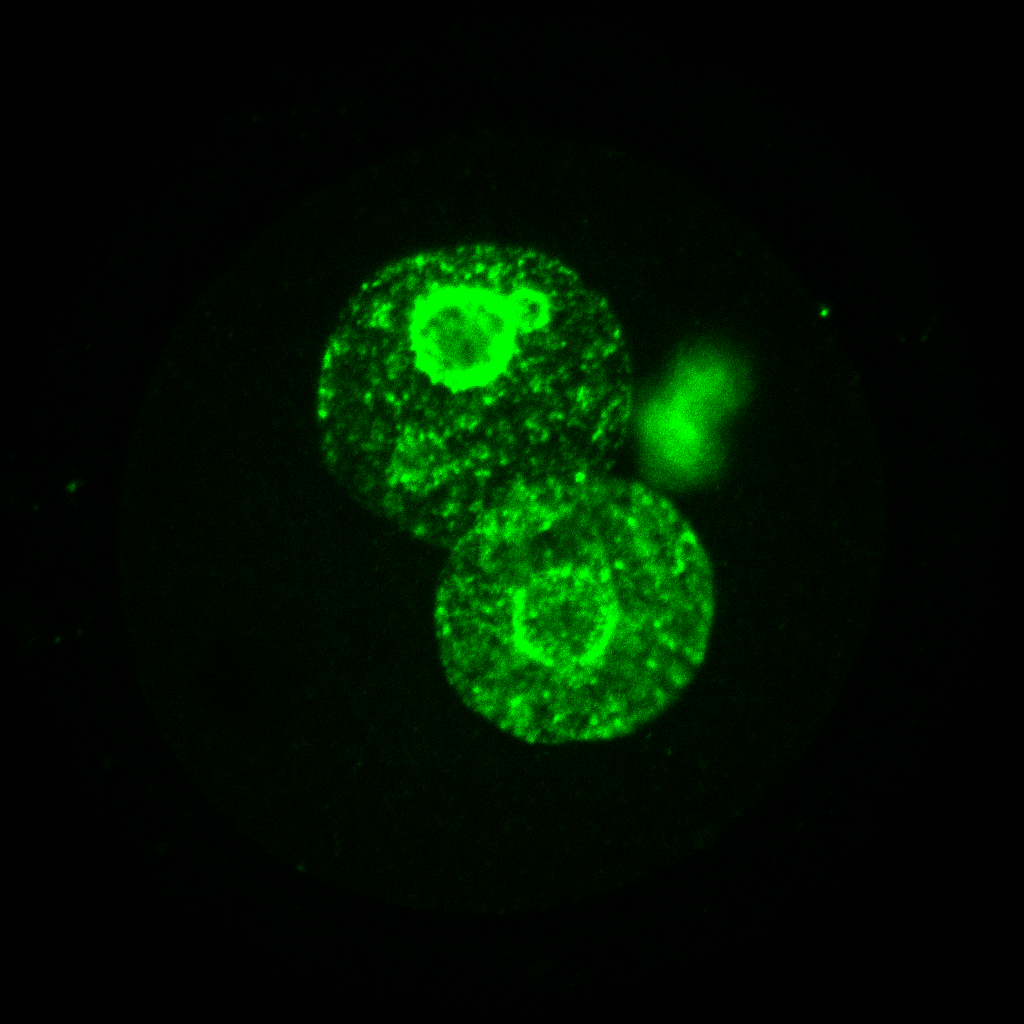

Supplement: Supplementary file 13 — Source data Fig. 2 [file 44319_2026_712_MOESM13_ESM.zip › Figure2 Source Data/2E/mKO.hCG27h.╬│H2AX.tif]

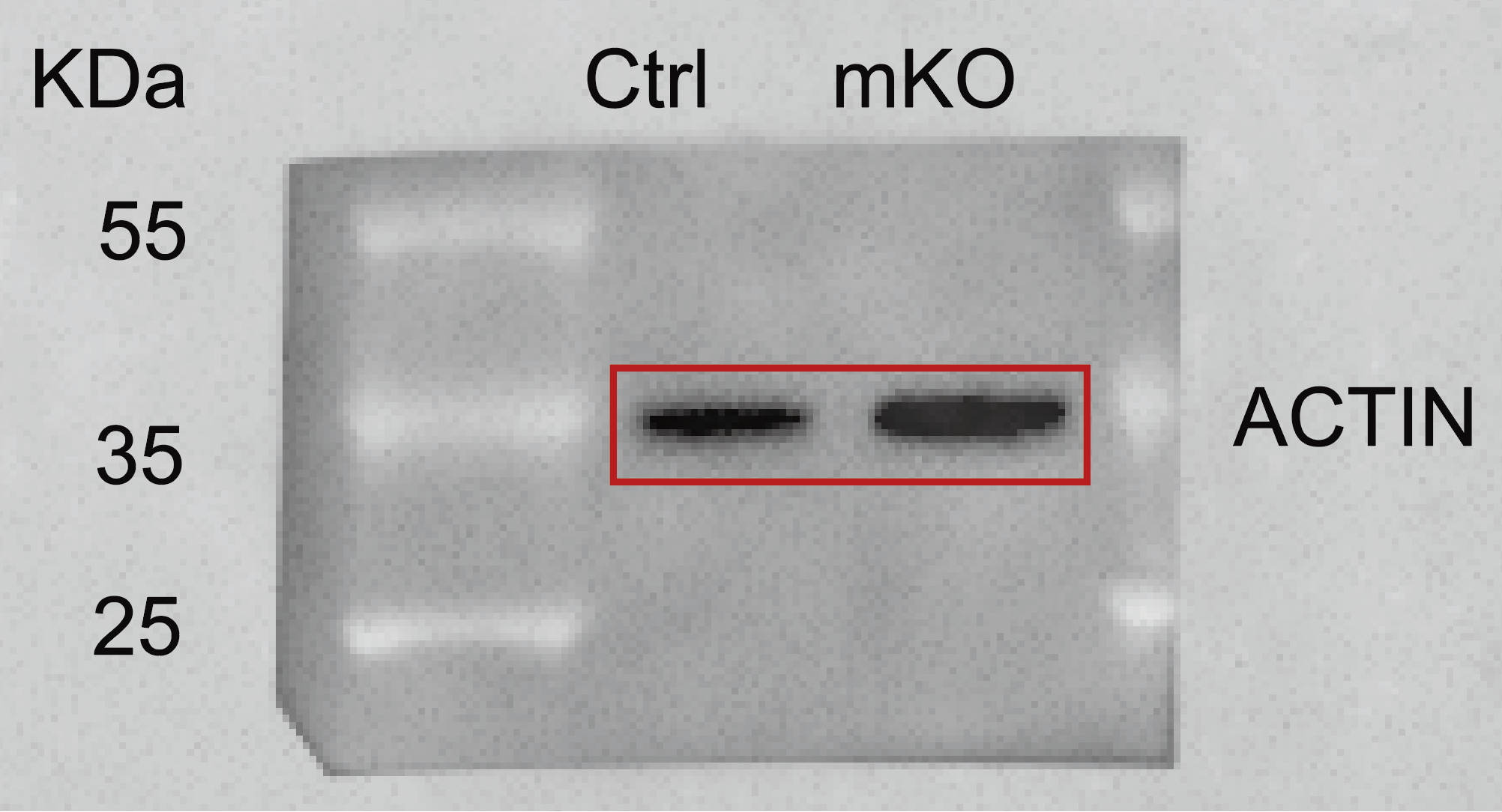

Supplement: Supplementary file 15 — Source data Fig. 4 [file 44319_2026_712_MOESM15_ESM.zip › Figure4 Source Data/4E/ACTIN.png]

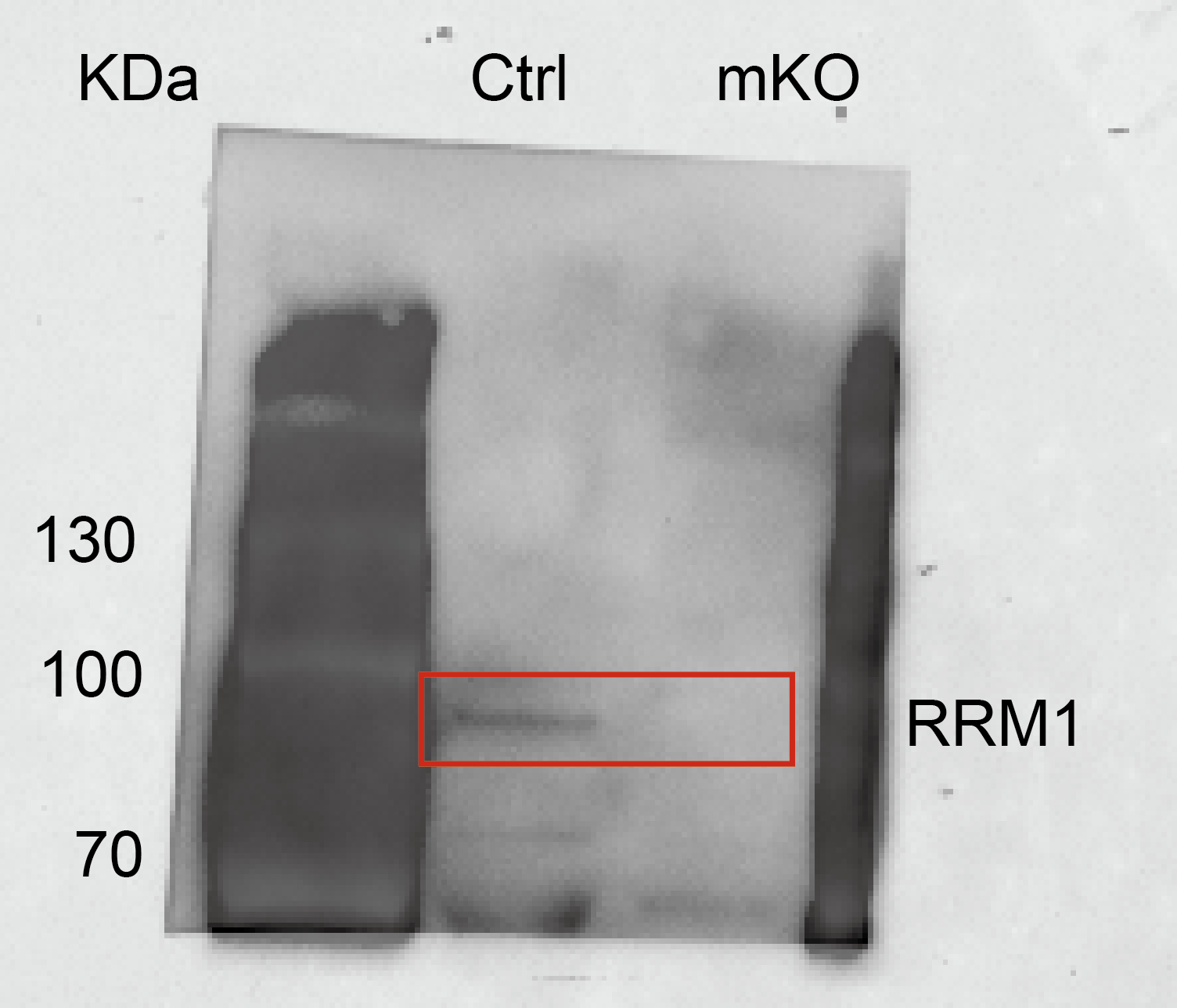

Supplement: Supplementary file 15 — Source data Fig. 4 [file 44319_2026_712_MOESM15_ESM.zip › Figure4 Source Data/4E/RRM1.png]

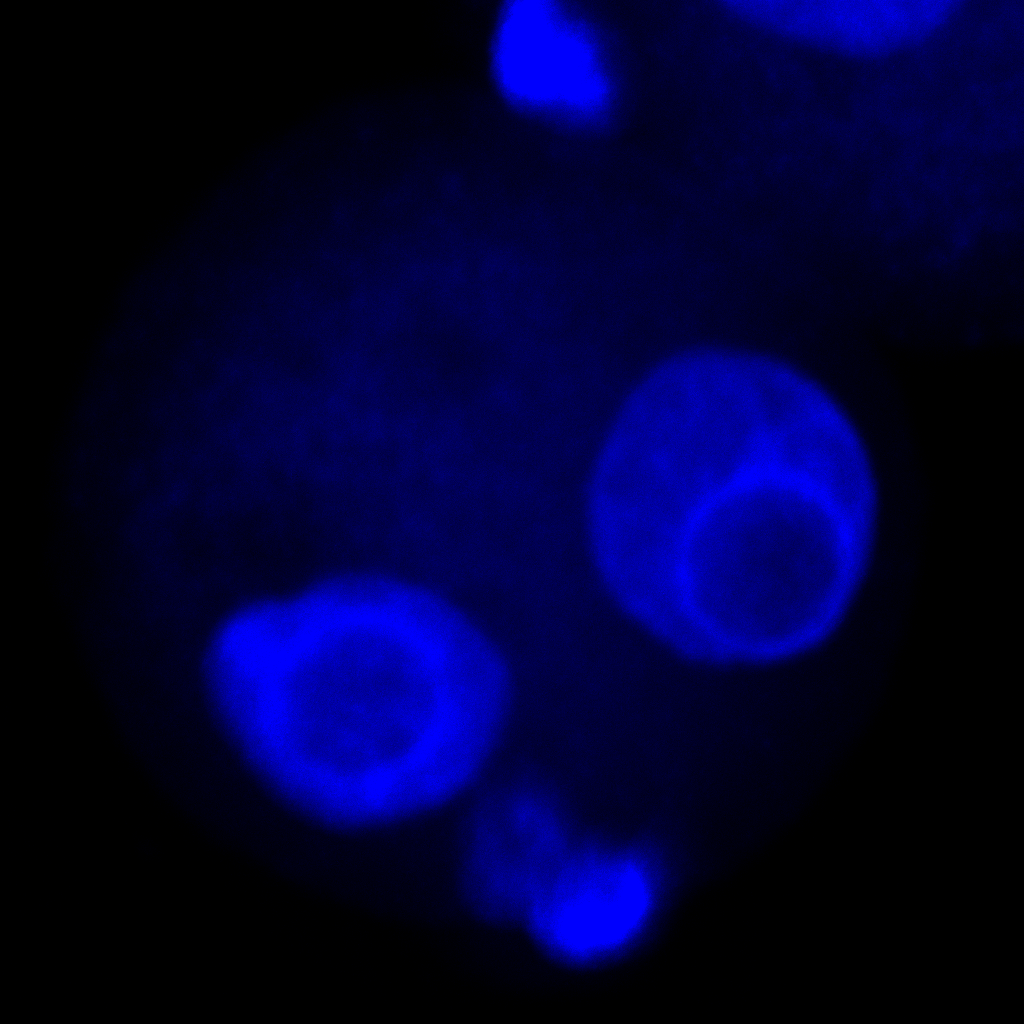

Supplement: Supplementary file 18 — Source data Fig. 7 [file 44319_2026_712_MOESM18_ESM.zip › Figure7 Source Data/7B/Ctrl_NT_DAPI.tif]

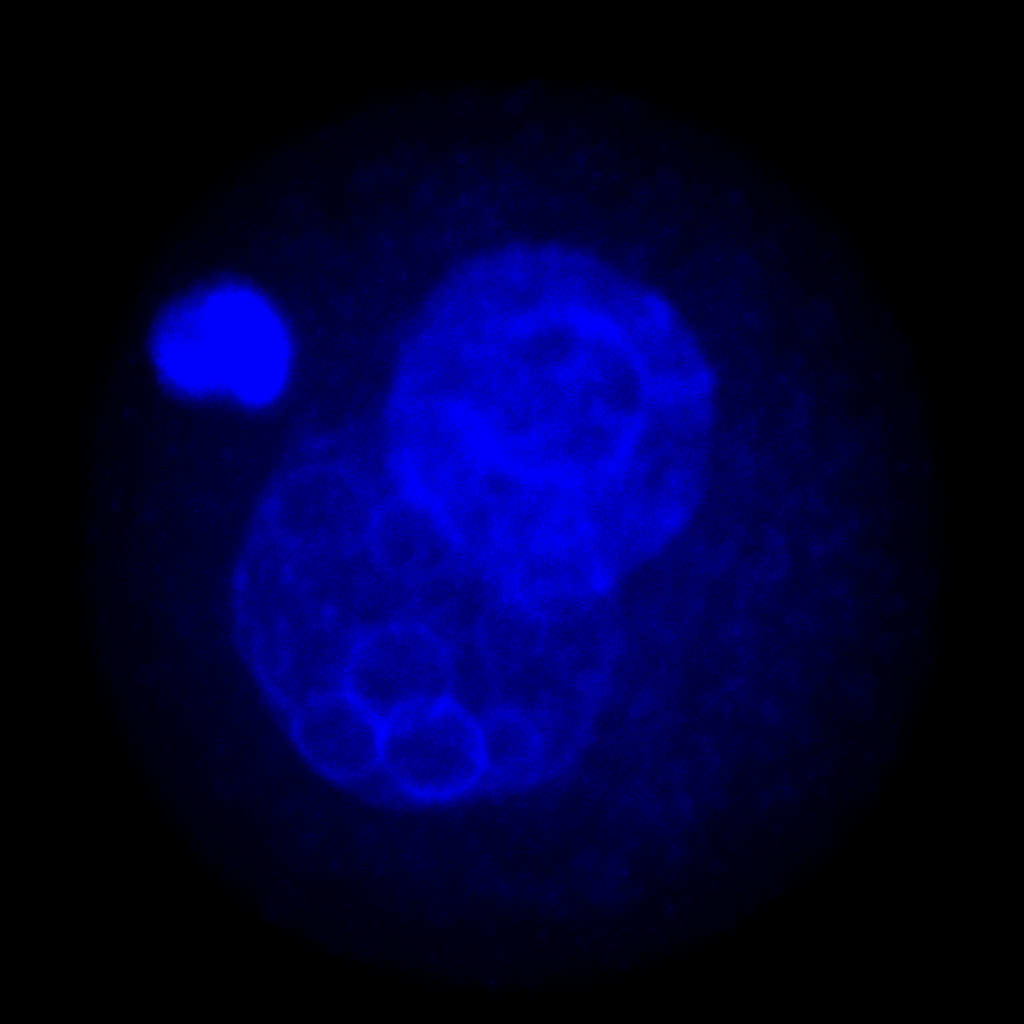

Supplement: Supplementary file 18 — Source data Fig. 7 [file 44319_2026_712_MOESM18_ESM.zip › Figure7 Source Data/7B/mKO_dNDP_DAPI.tif]

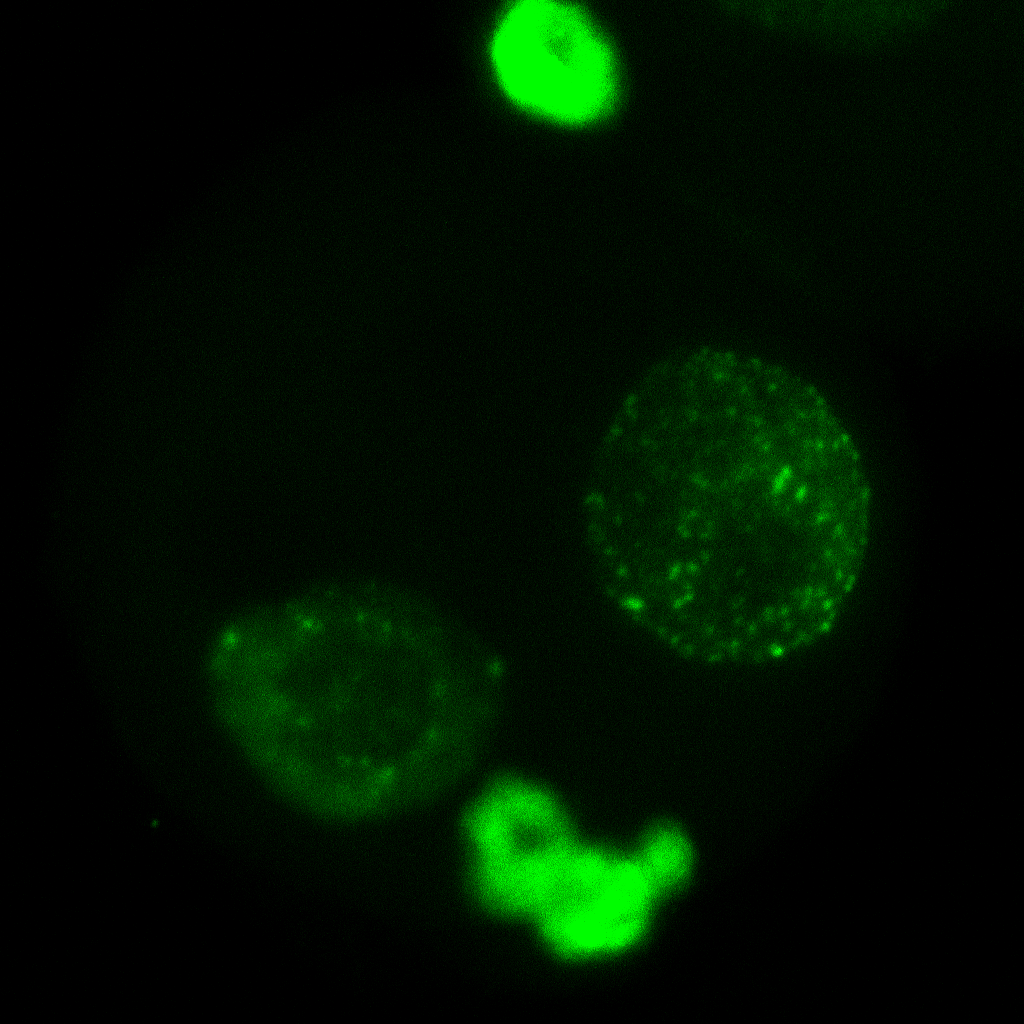

Supplement: Supplementary file 18 — Source data Fig. 7 [file 44319_2026_712_MOESM18_ESM.zip › Figure7 Source Data/7B/Ctrl_NT_╬│H2AX.tif]

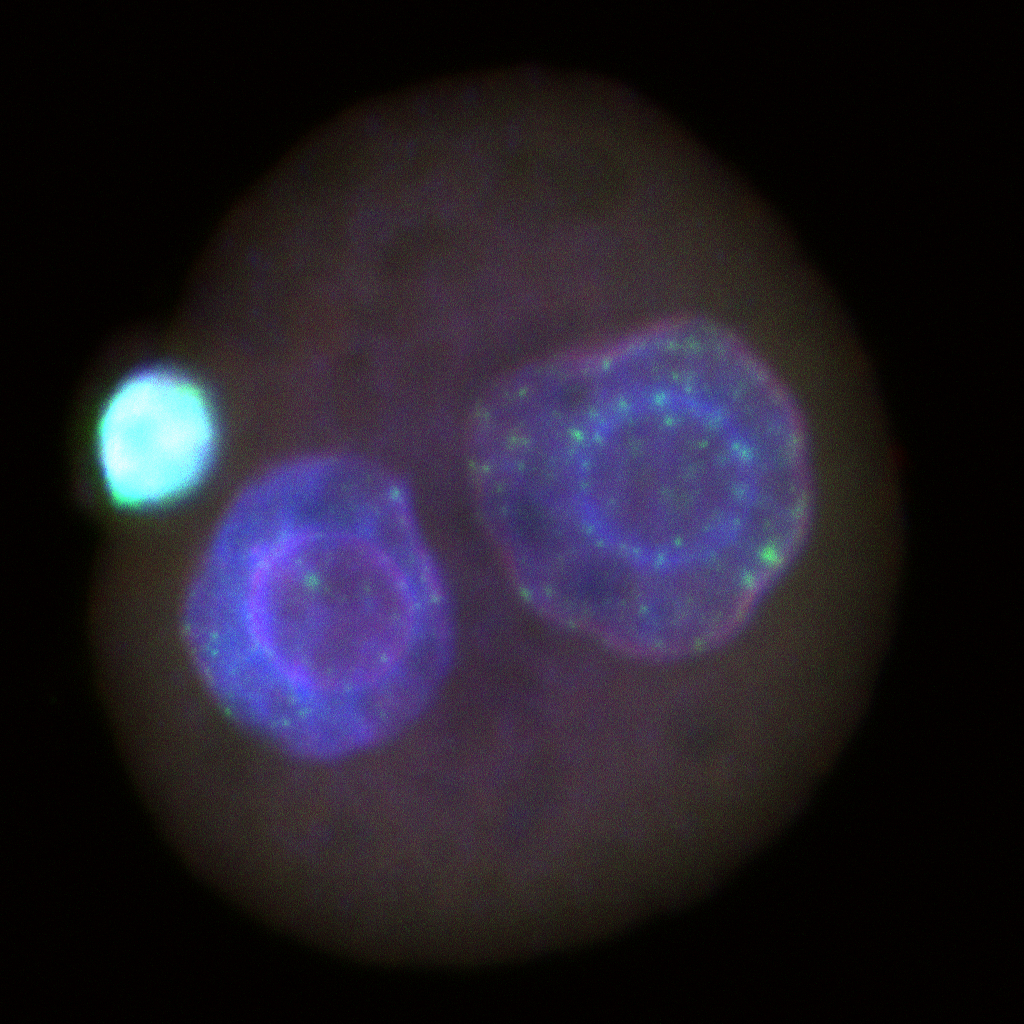

Supplement: Supplementary file 18 — Source data Fig. 7 [file 44319_2026_712_MOESM18_ESM.zip › Figure7 Source Data/7B/Ctrl_dNDP_Merge.tif]

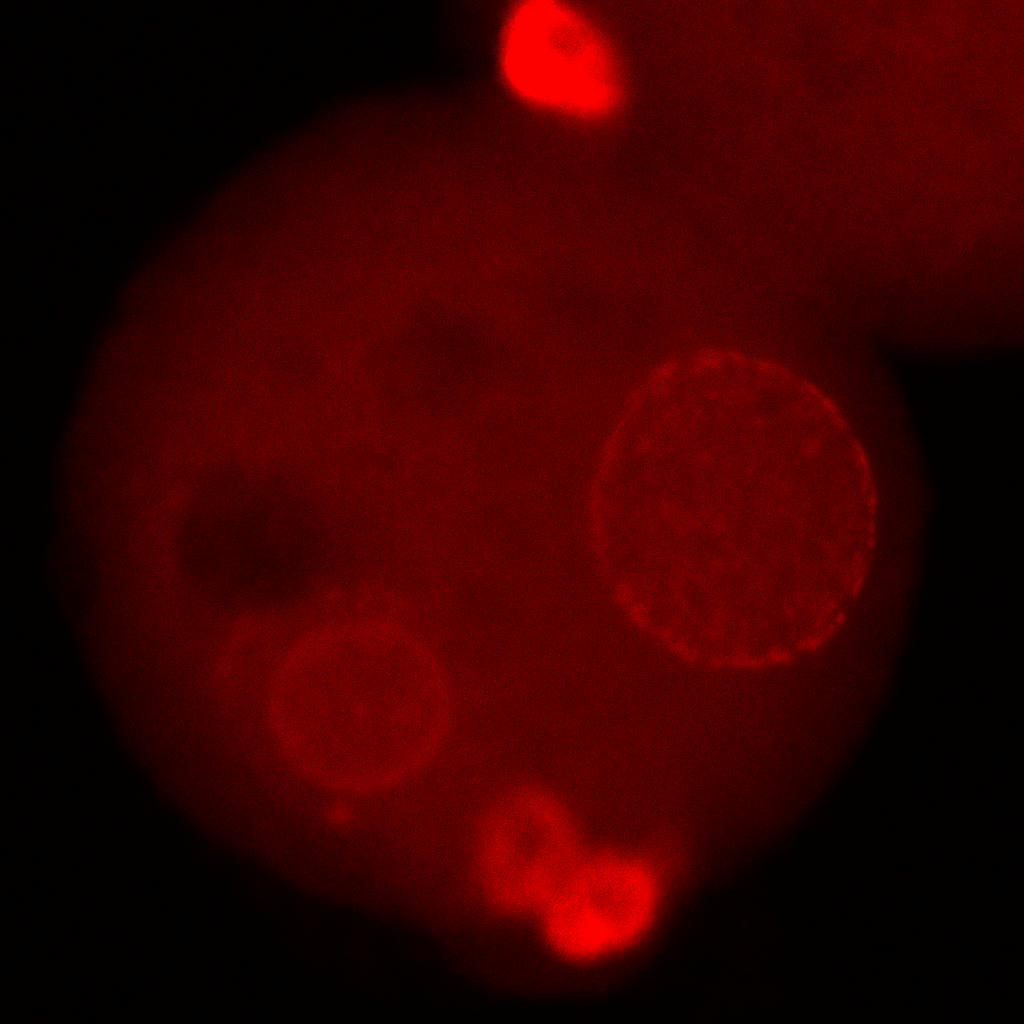

Supplement: Supplementary file 18 — Source data Fig. 7 [file 44319_2026_712_MOESM18_ESM.zip › Figure7 Source Data/7B/Ctrl_NT_EdU.tif]

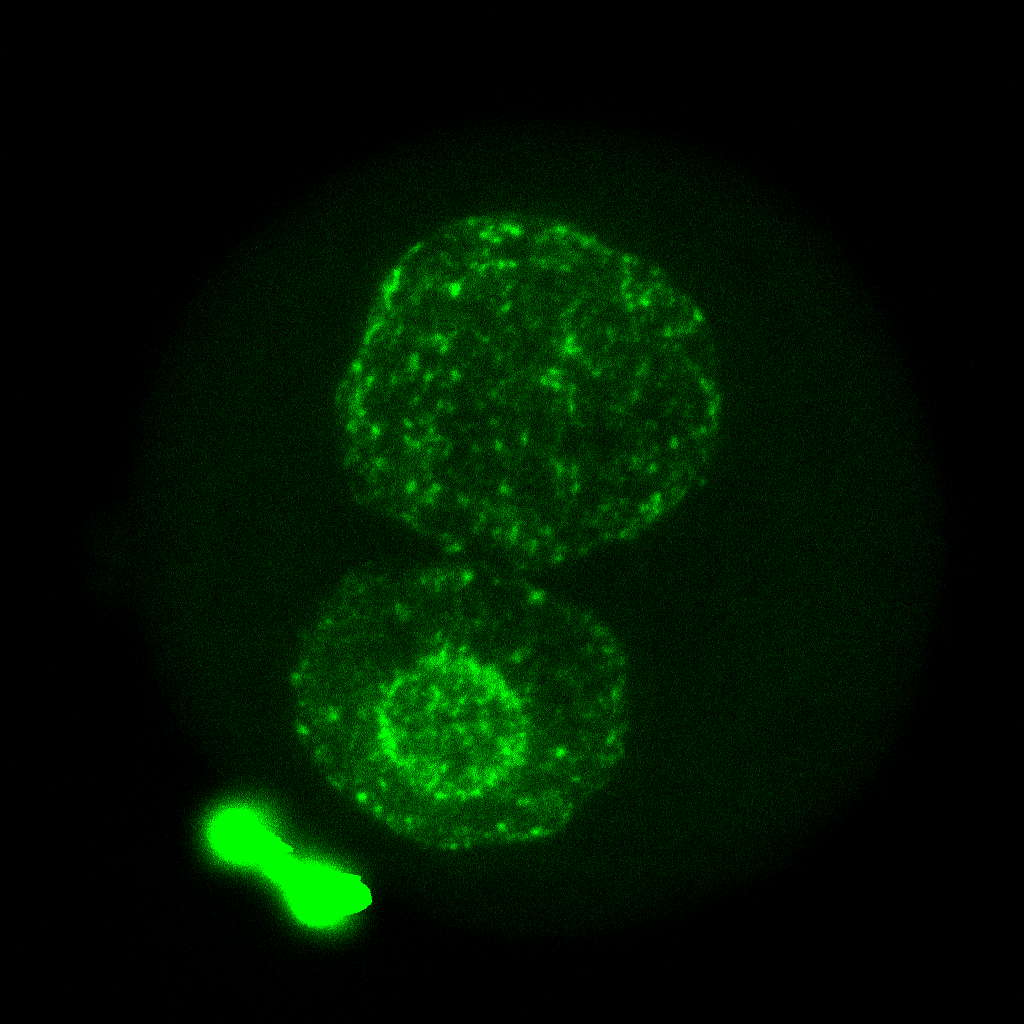

Supplement: Supplementary file 18 — Source data Fig. 7 [file 44319_2026_712_MOESM18_ESM.zip › Figure7 Source Data/7B/mKO_NT_╬│H2AX.tif]

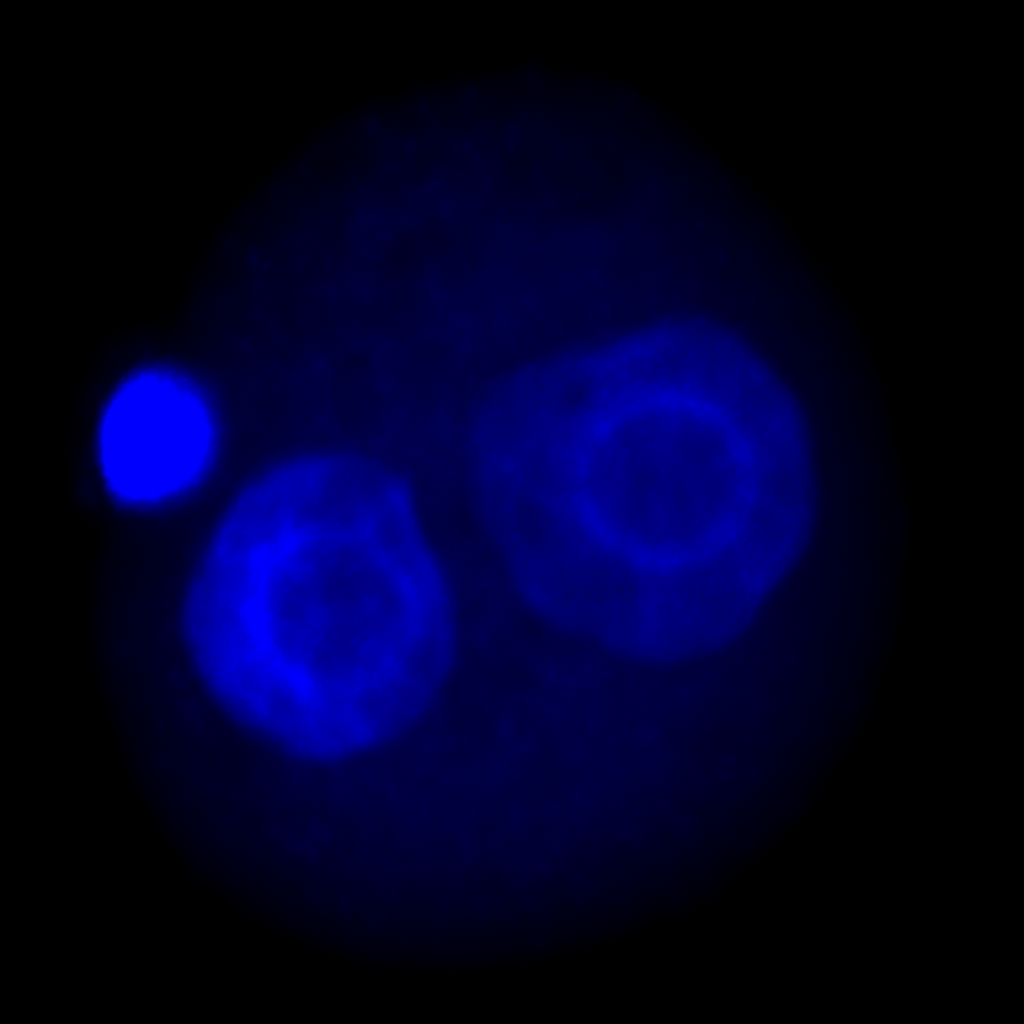

Supplement: Supplementary file 18 — Source data Fig. 7 [file 44319_2026_712_MOESM18_ESM.zip › Figure7 Source Data/7B/Ctrl_dNDP_DAPI.tif]

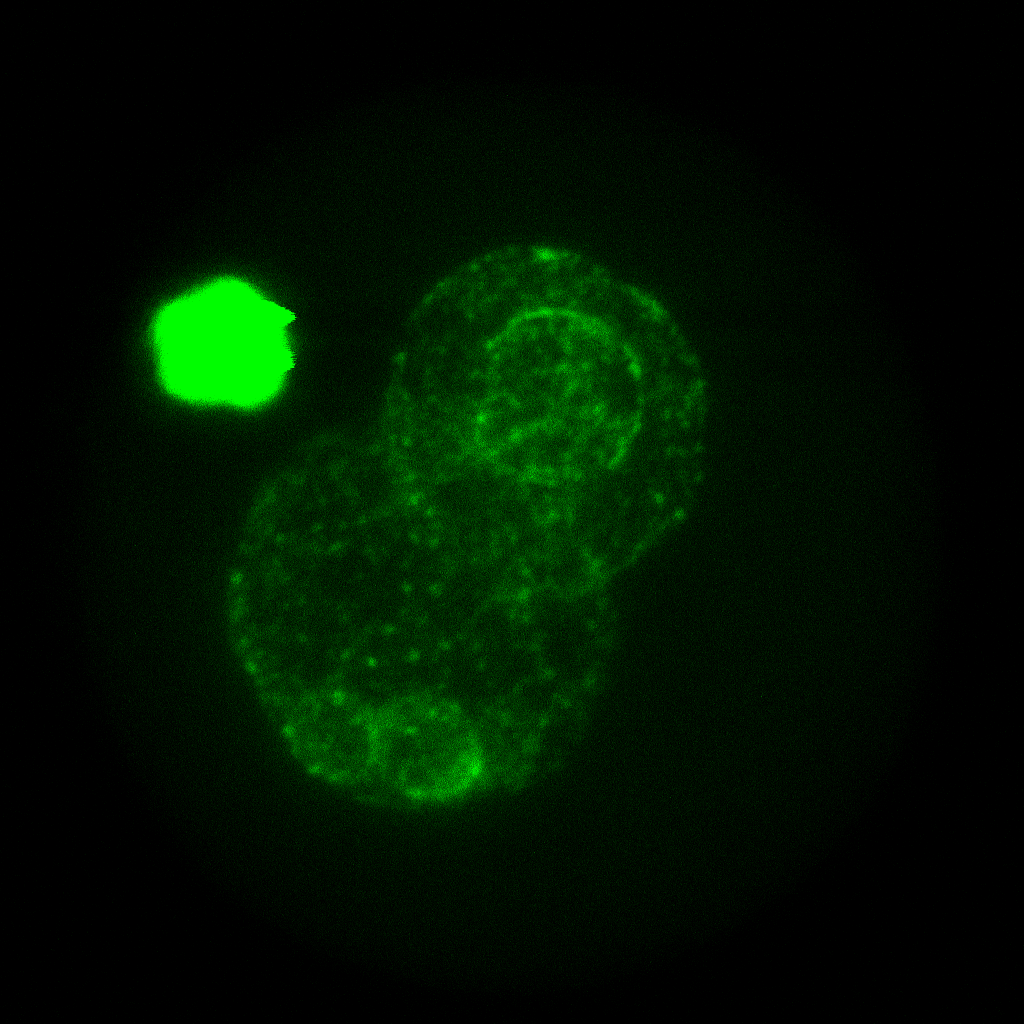

Supplement: Supplementary file 18 — Source data Fig. 7 [file 44319_2026_712_MOESM18_ESM.zip › Figure7 Source Data/7B/mKO_dNDP_╬│H2AX.tif]

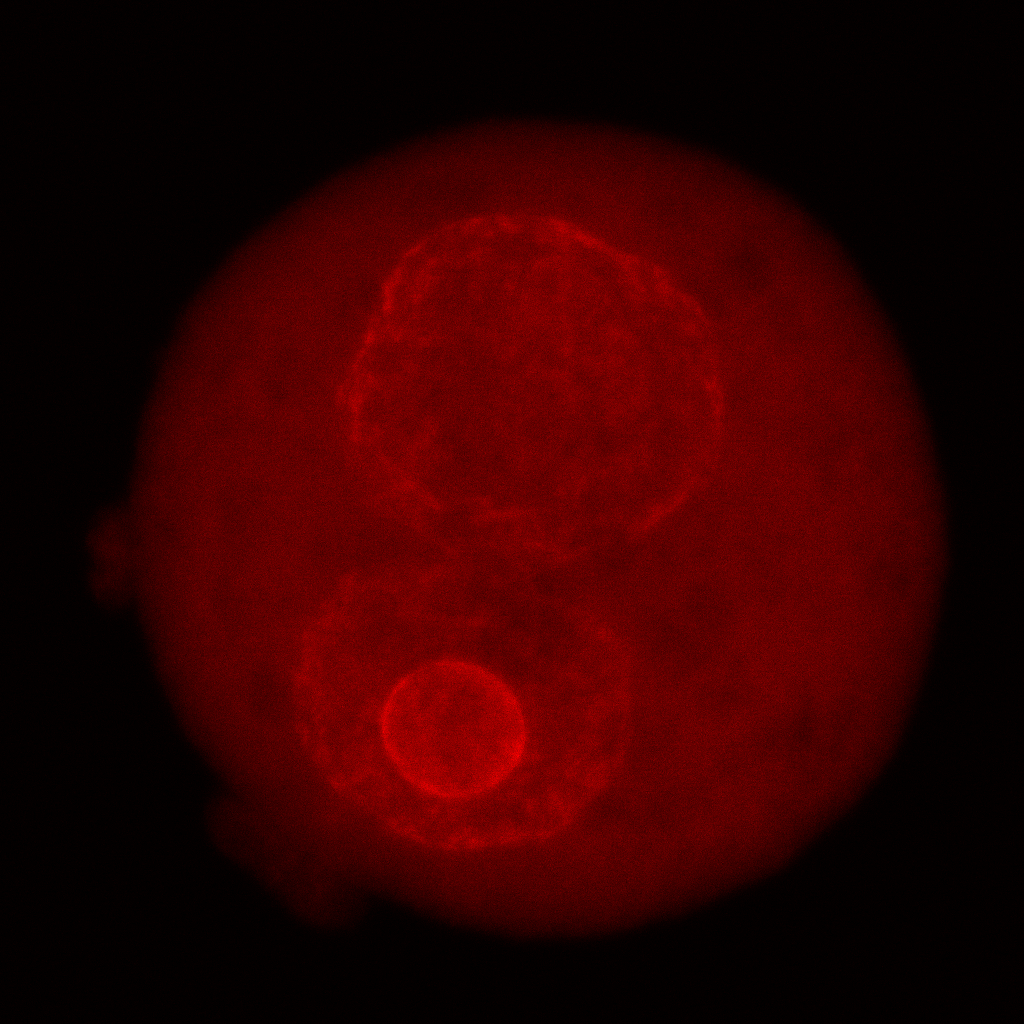

Supplement: Supplementary file 18 — Source data Fig. 7 [file 44319_2026_712_MOESM18_ESM.zip › Figure7 Source Data/7B/mKO_NT_EdU.tif]

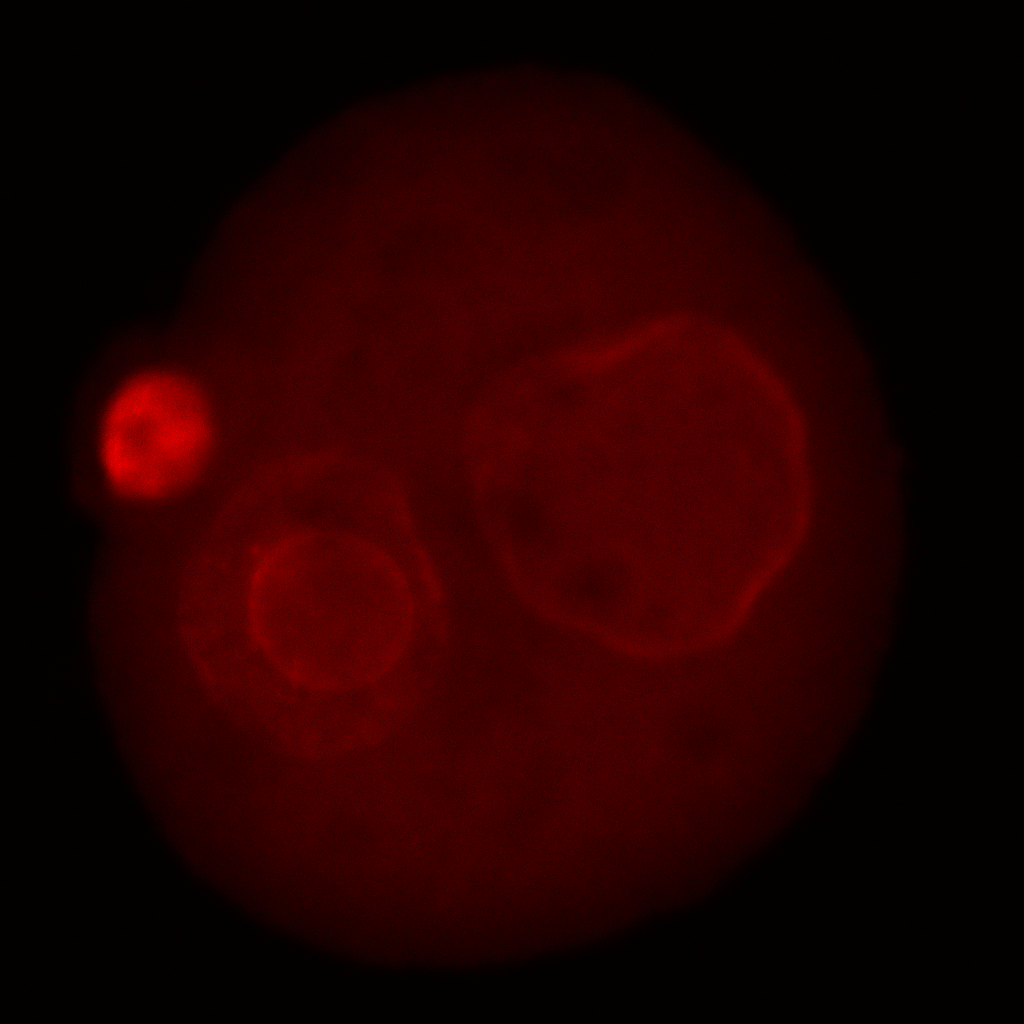

Supplement: Supplementary file 18 — Source data Fig. 7 [file 44319_2026_712_MOESM18_ESM.zip › Figure7 Source Data/7B/Ctrl_dNDP_EdU.tif]

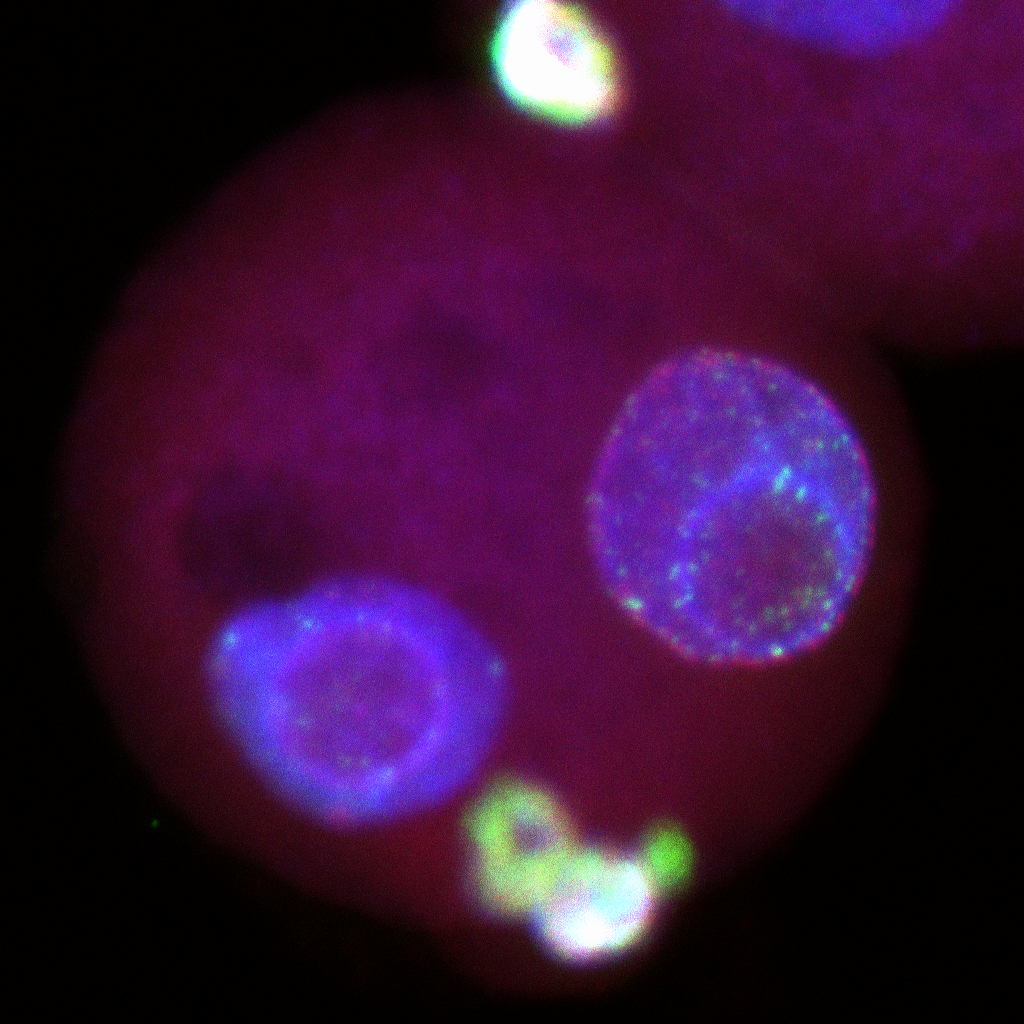

Supplement: Supplementary file 18 — Source data Fig. 7 [file 44319_2026_712_MOESM18_ESM.zip › Figure7 Source Data/7B/Ctrl_NT_Merge.tif]

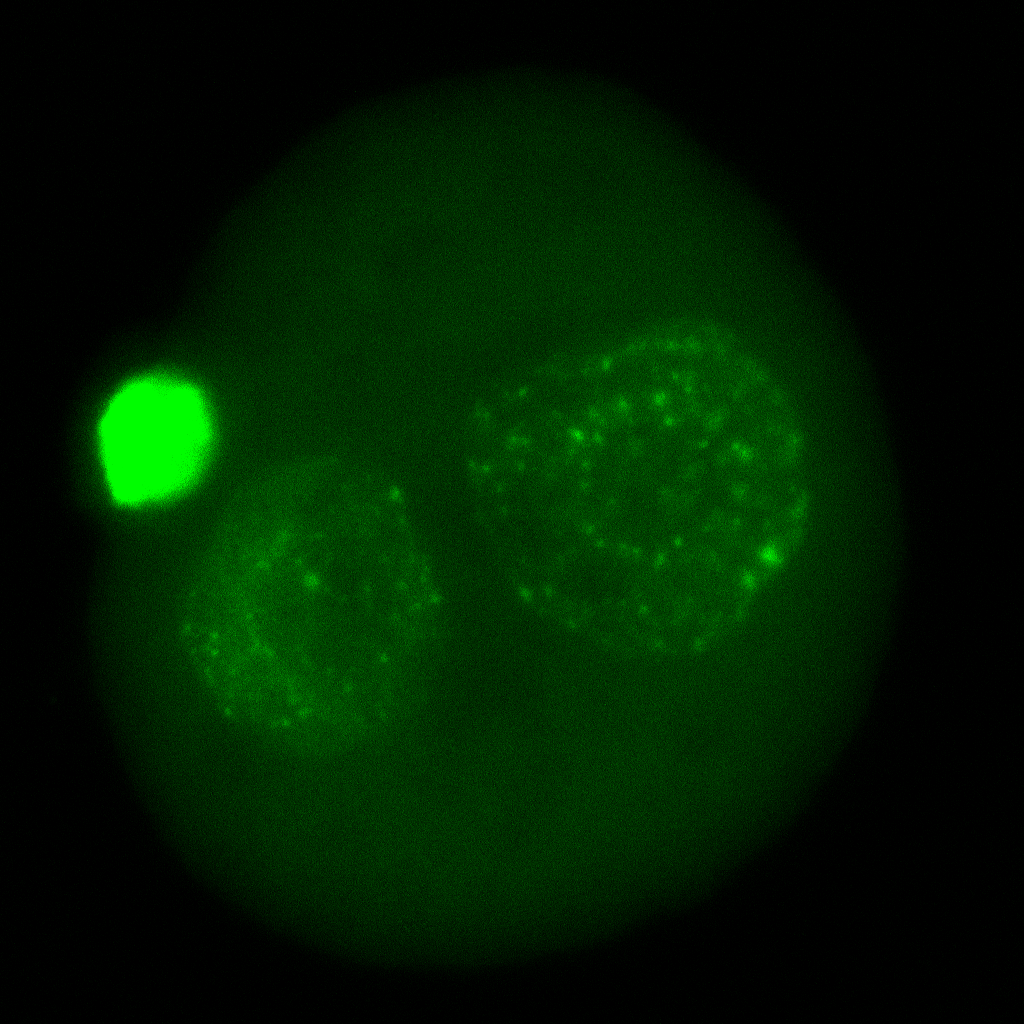

Supplement: Supplementary file 18 — Source data Fig. 7 [file 44319_2026_712_MOESM18_ESM.zip › Figure7 Source Data/7B/Ctrl_dNDP_╬│H2AX.tif]

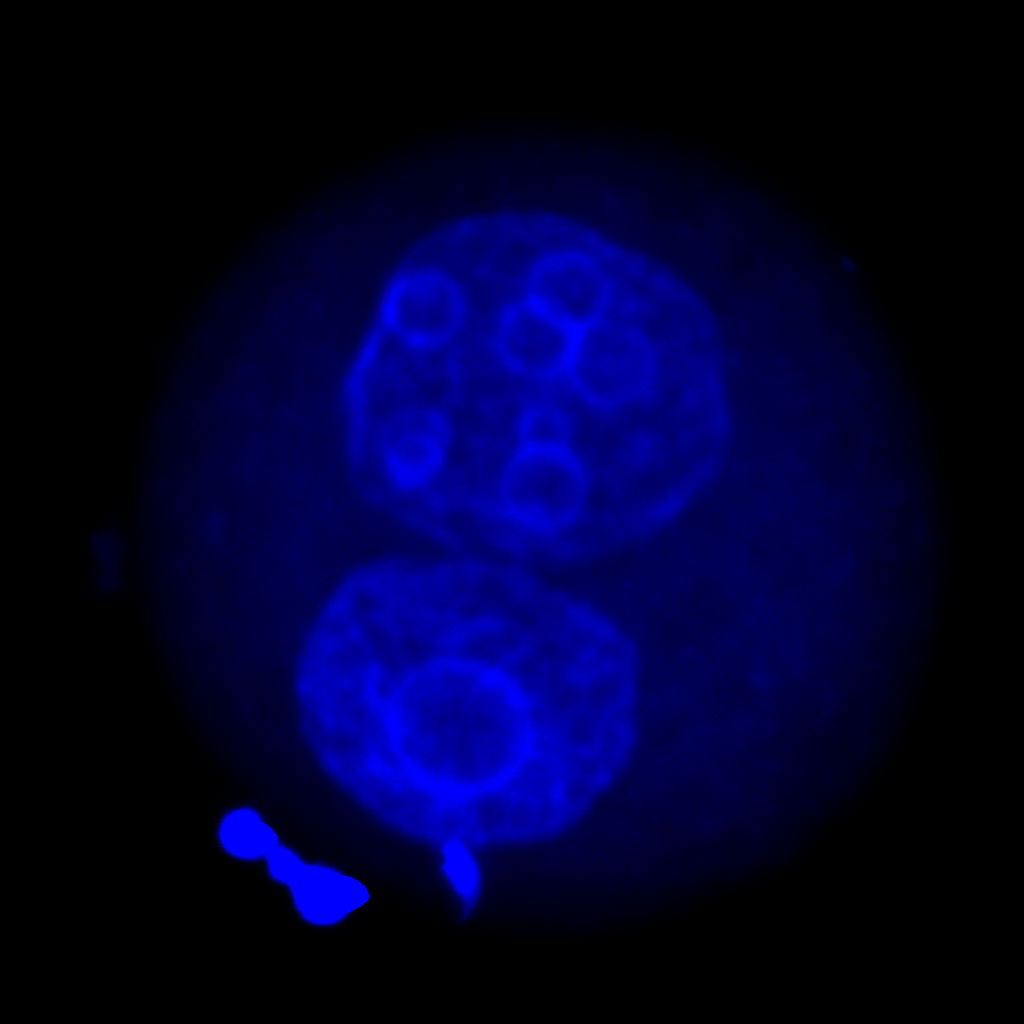

Supplement: Supplementary file 18 — Source data Fig. 7 [file 44319_2026_712_MOESM18_ESM.zip › Figure7 Source Data/7B/mKO_NT_DAPI.tif]

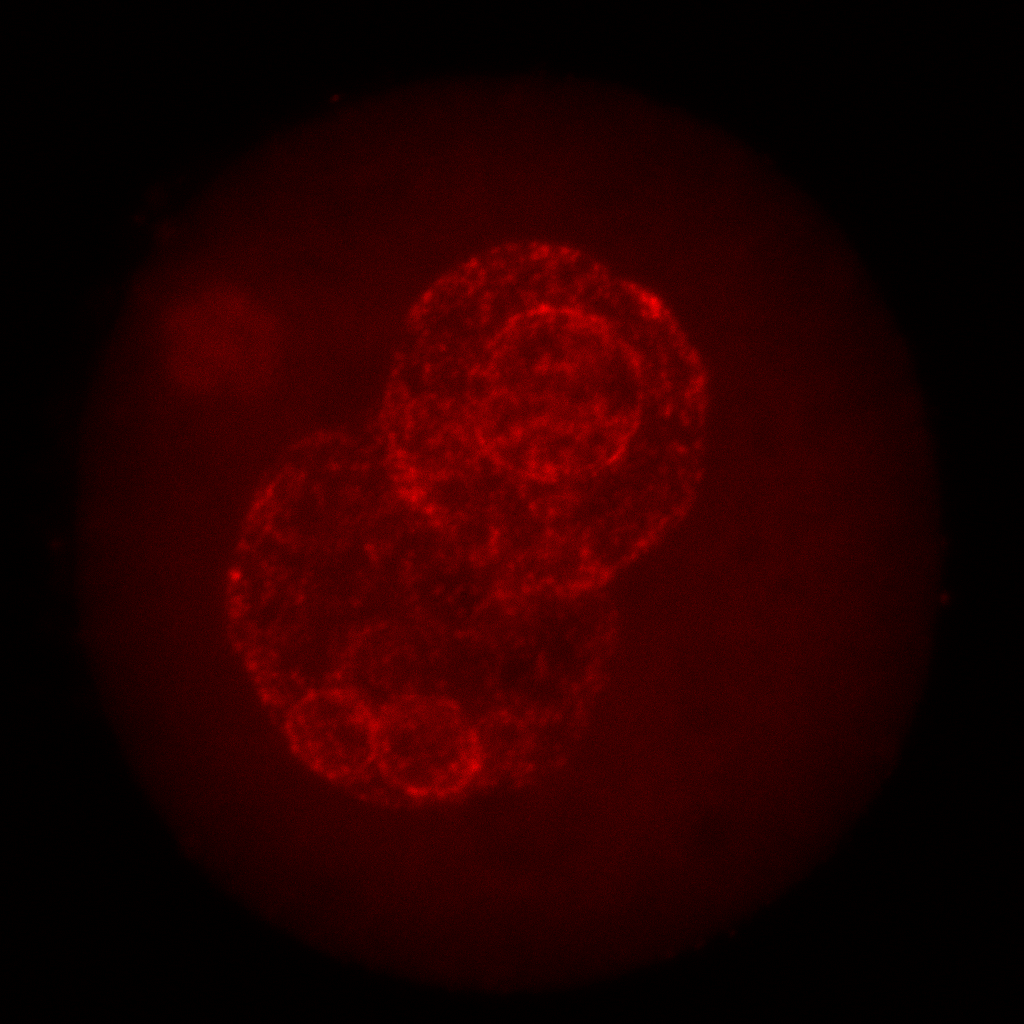

Supplement: Supplementary file 18 — Source data Fig. 7 [file 44319_2026_712_MOESM18_ESM.zip › Figure7 Source Data/7B/mKO_dNDP_EdU.tif]

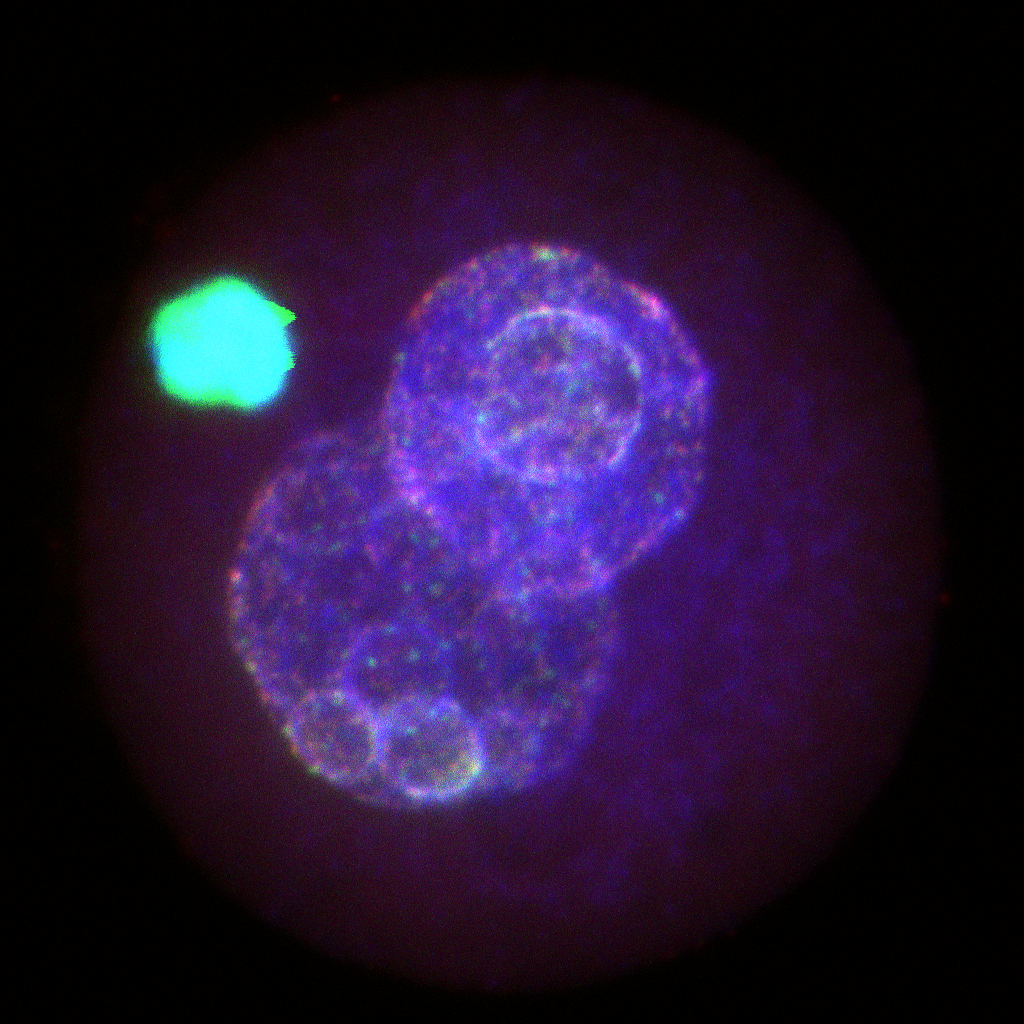

Supplement: Supplementary file 18 — Source data Fig. 7 [file 44319_2026_712_MOESM18_ESM.zip › Figure7 Source Data/7B/mKO_dNDP_Merge.tif]

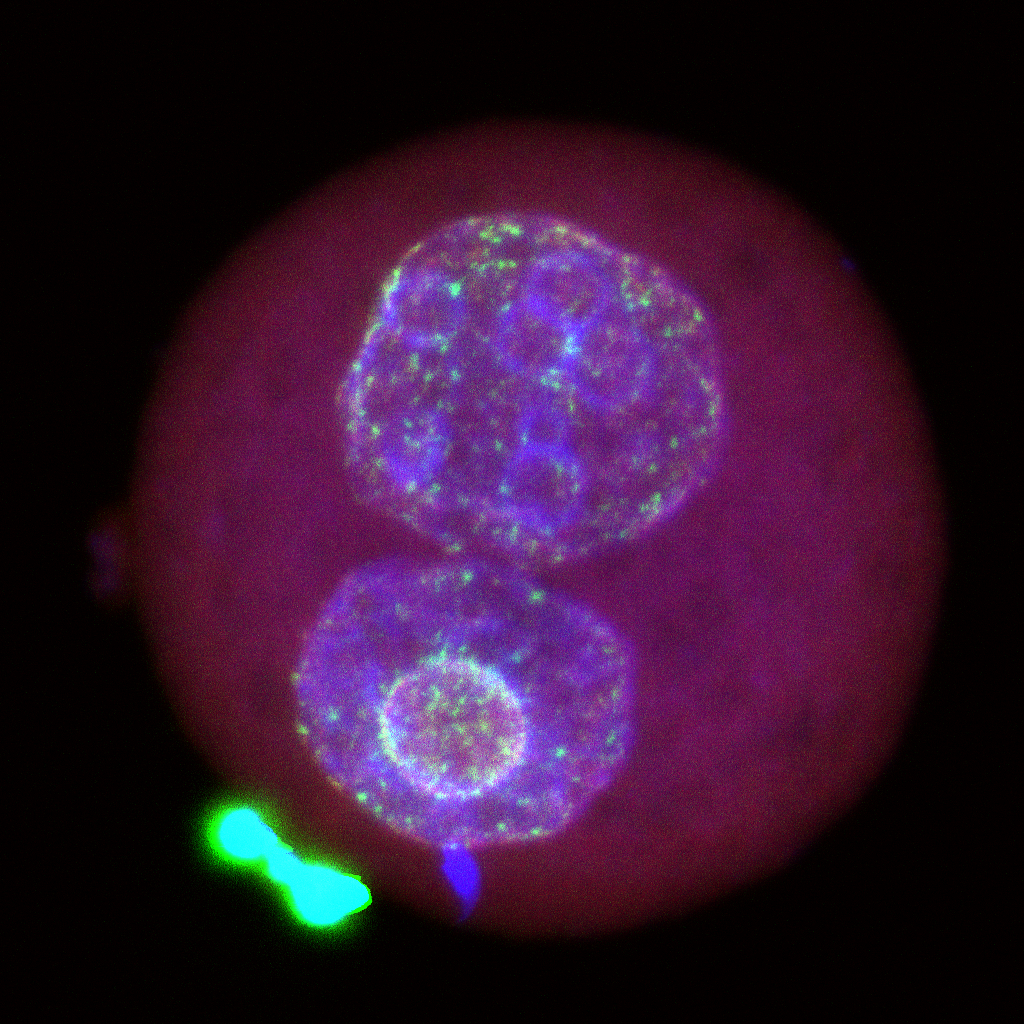

Supplement: Supplementary file 18 — Source data Fig. 7 [file 44319_2026_712_MOESM18_ESM.zip › Figure7 Source Data/7B/mKO_NT_Merge.tif]

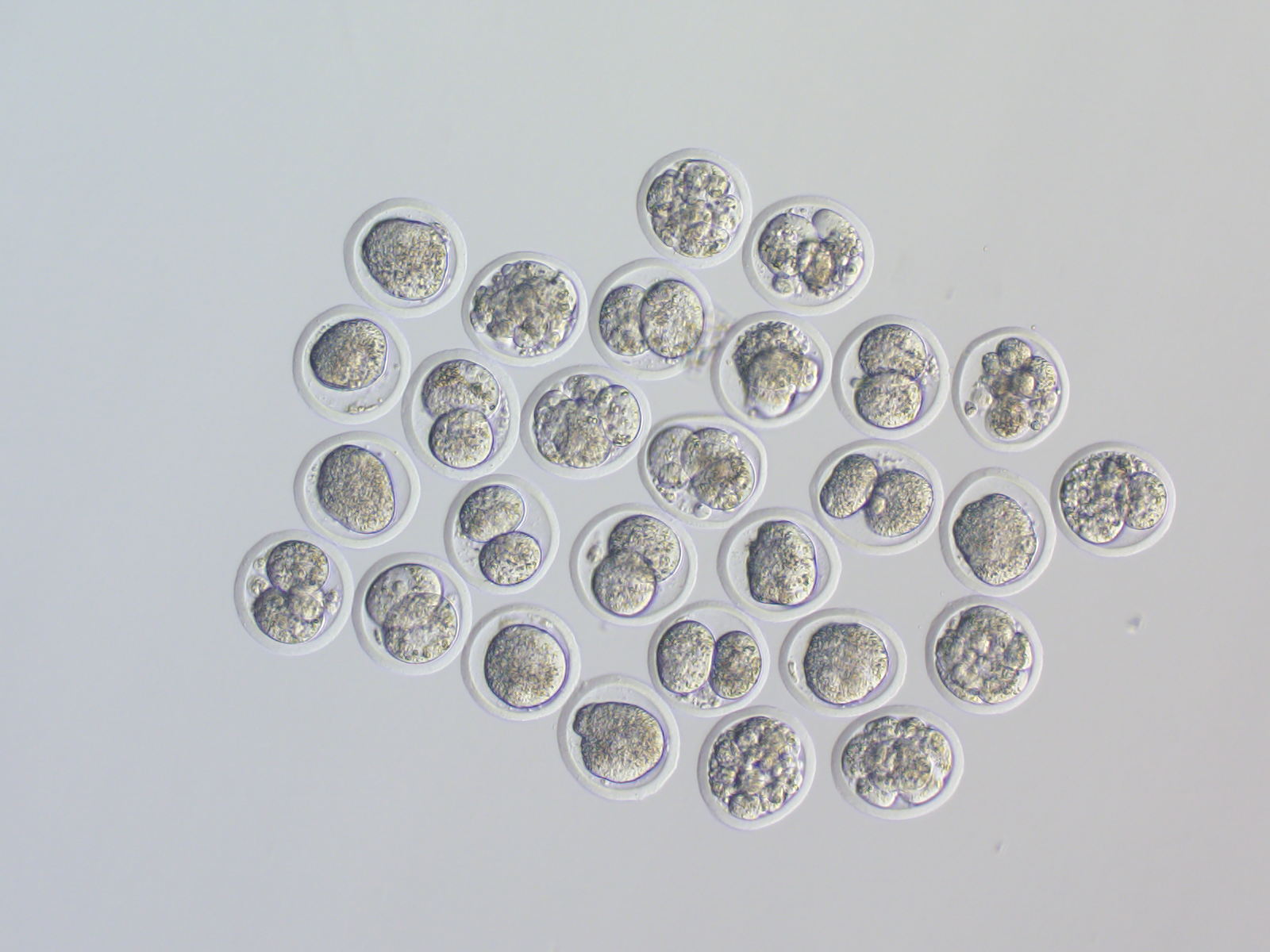

Supplement: Supplementary file 19 — Appendix Source Data [file 44319_2026_712_MOESM19_ESM.zip › Appendix source data/Appendix Figure S3/S3I/mKO_NT_PA106h_σë»μ£1⁄4.tif]

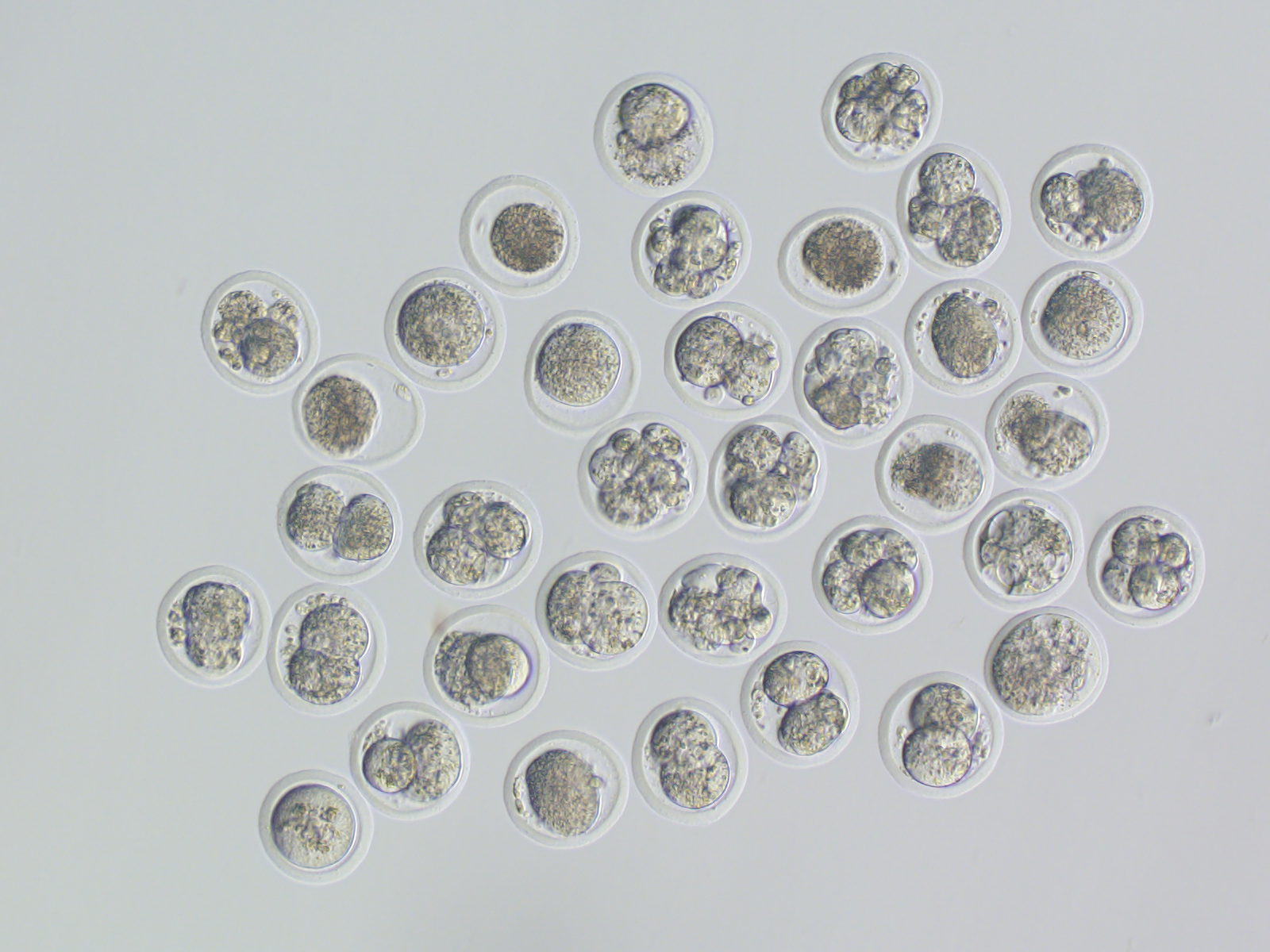

Supplement: Supplementary file 19 — Appendix Source Data [file 44319_2026_712_MOESM19_ESM.zip › Appendix source data/Appendix Figure S3/S3I/mKO_OE Thap1_PA106h_σë»μ£1⁄4.tif]

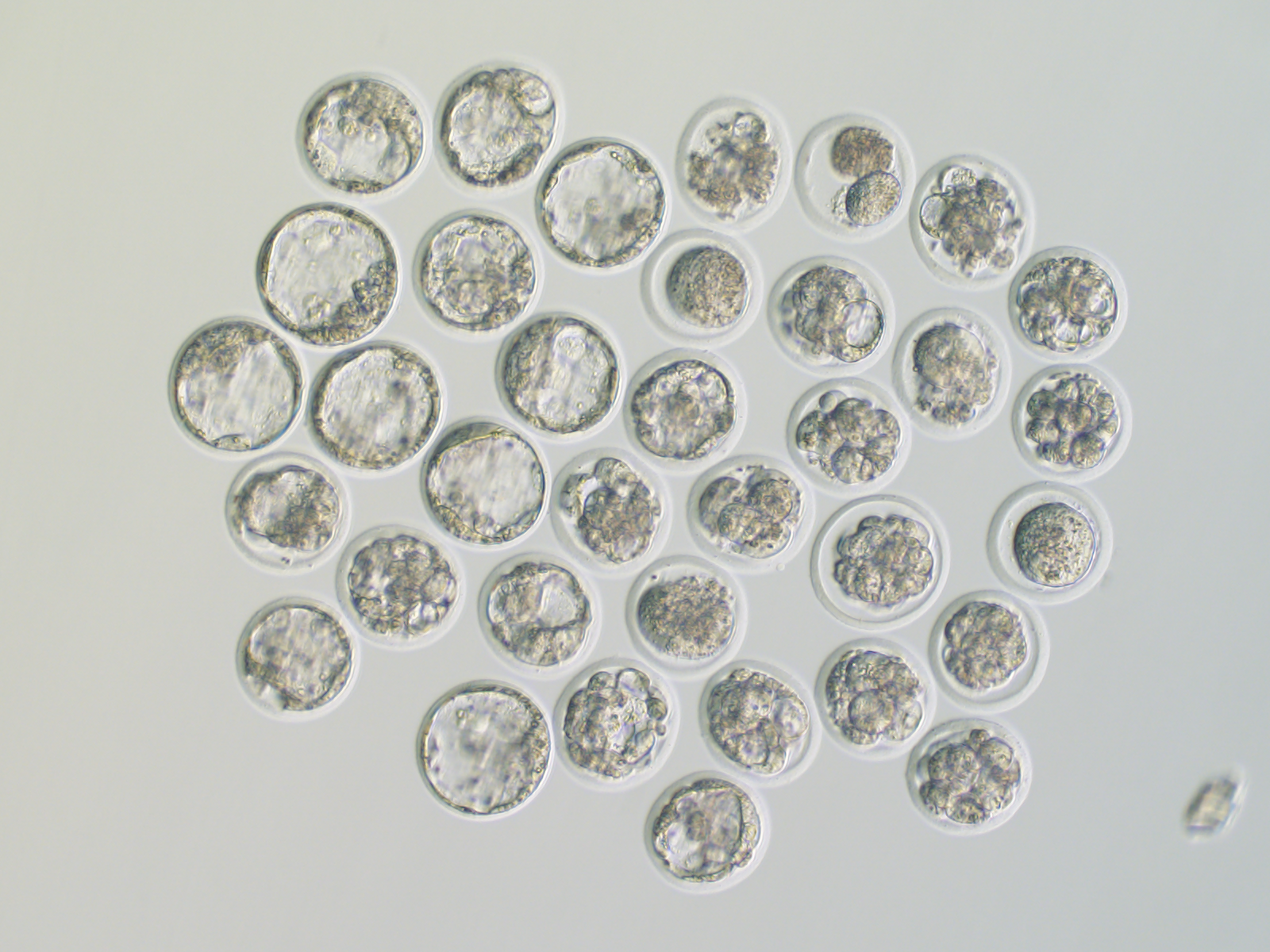

Supplement: Supplementary file 19 — Appendix Source Data [file 44319_2026_712_MOESM19_ESM.zip › Appendix source data/Appendix Figure S3/S3I/Ctrl_NT_PA106h_σë»μ£1⁄4.tif]

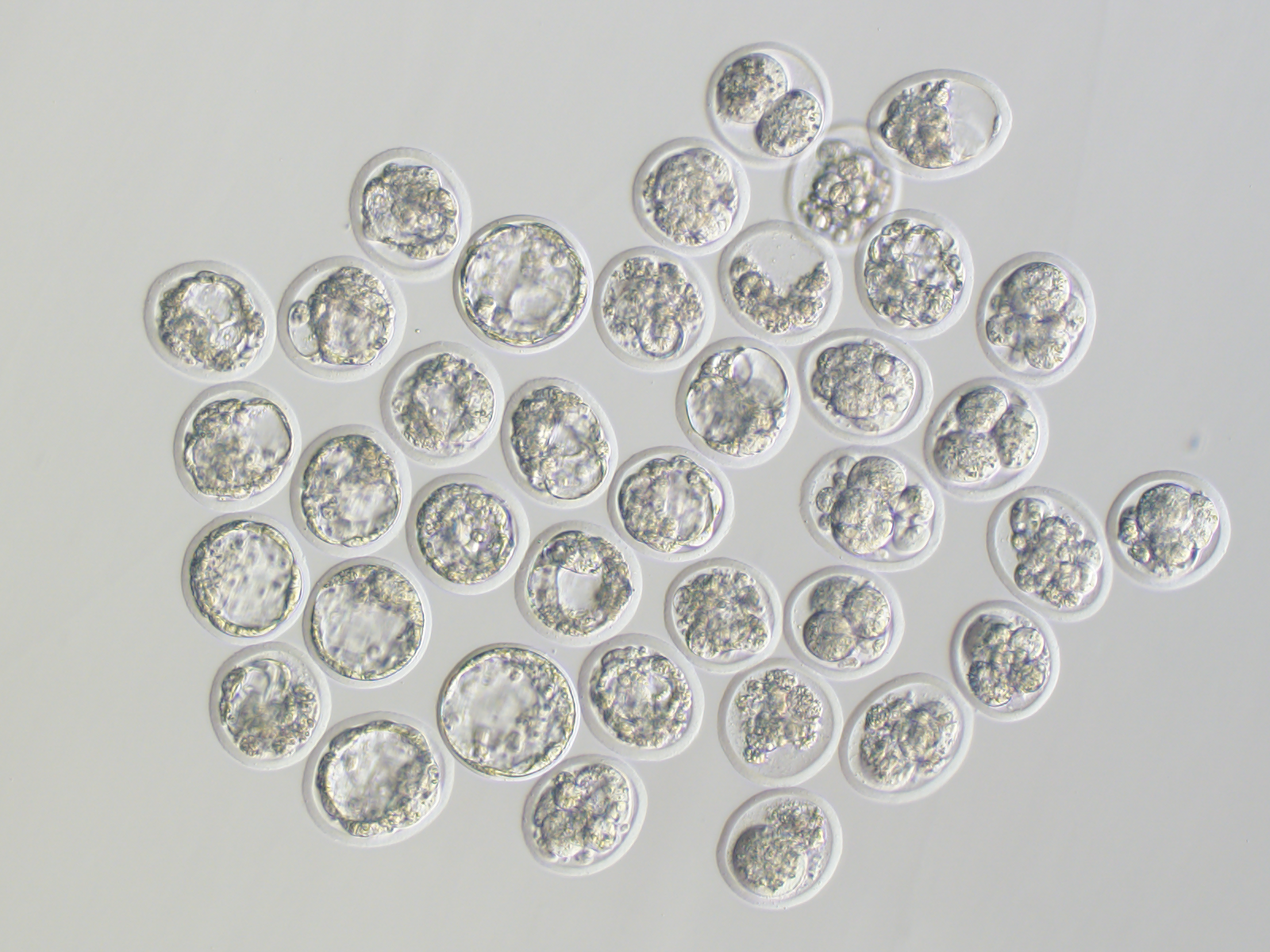

Supplement: Supplementary file 19 — Appendix Source Data [file 44319_2026_712_MOESM19_ESM.zip › Appendix source data/Appendix Figure S3/S3I/Ctrl_OE_PA106h_σë»μ£1⁄4.tif]

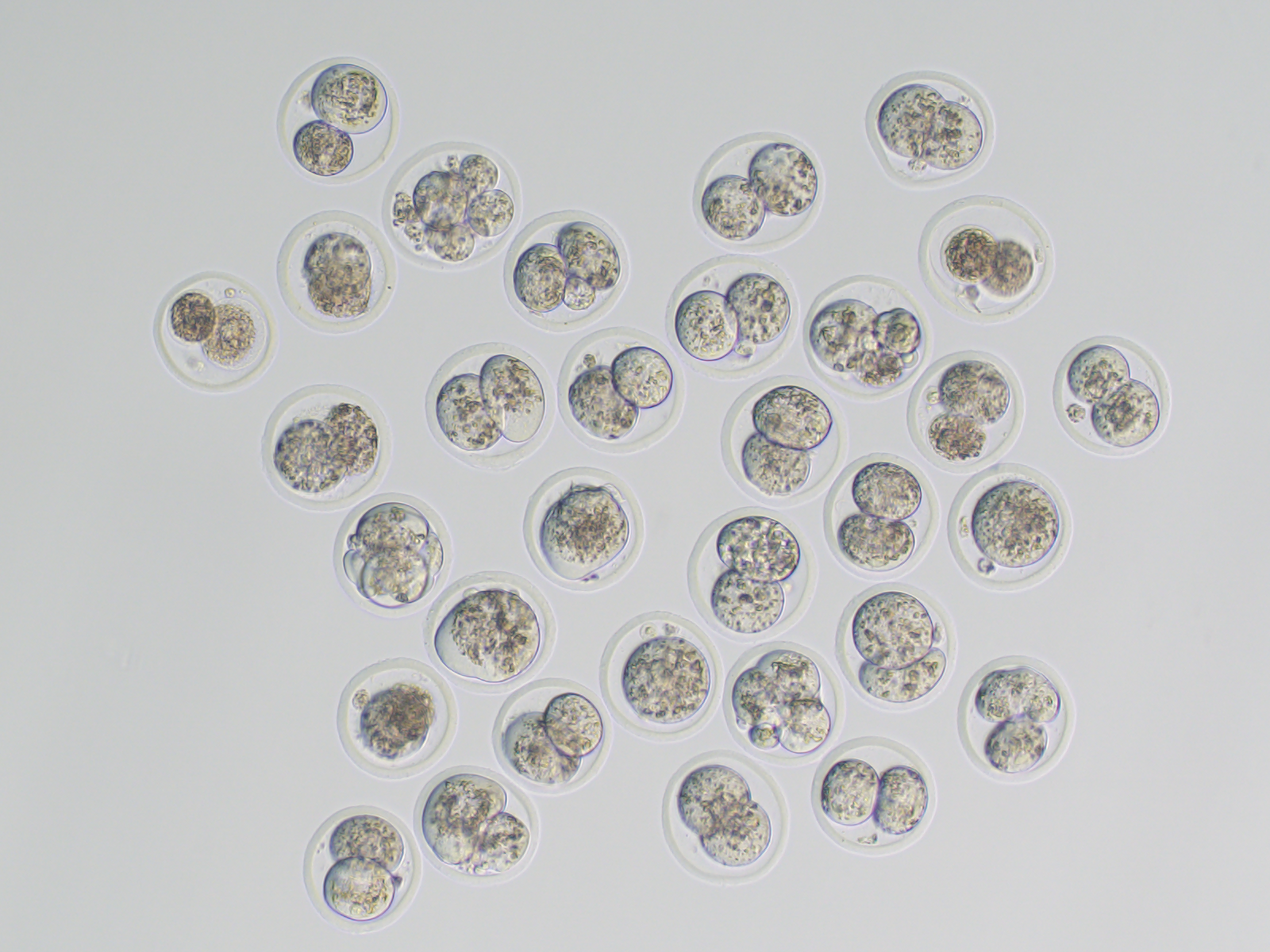

Supplement: Supplementary file 19 — Appendix Source Data [file 44319_2026_712_MOESM19_ESM.zip › Appendix source data/Appendix Figure S3/S3A/mKO_250 ng_ul.tif]

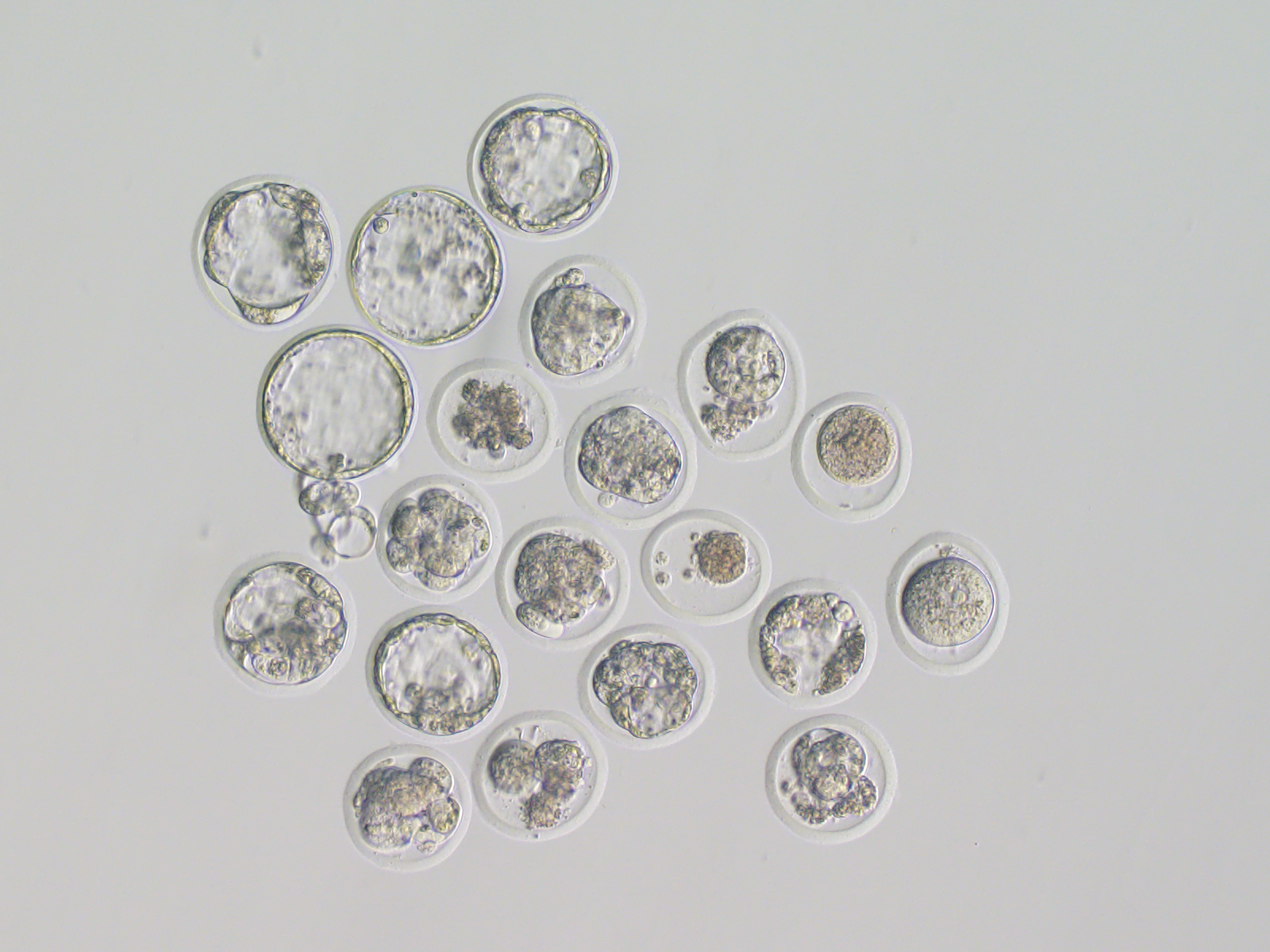

Supplement: Supplementary file 19 — Appendix Source Data [file 44319_2026_712_MOESM19_ESM.zip › Appendix source data/Appendix Figure S3/S3A/Ctrl_250 ng_ul.tif]

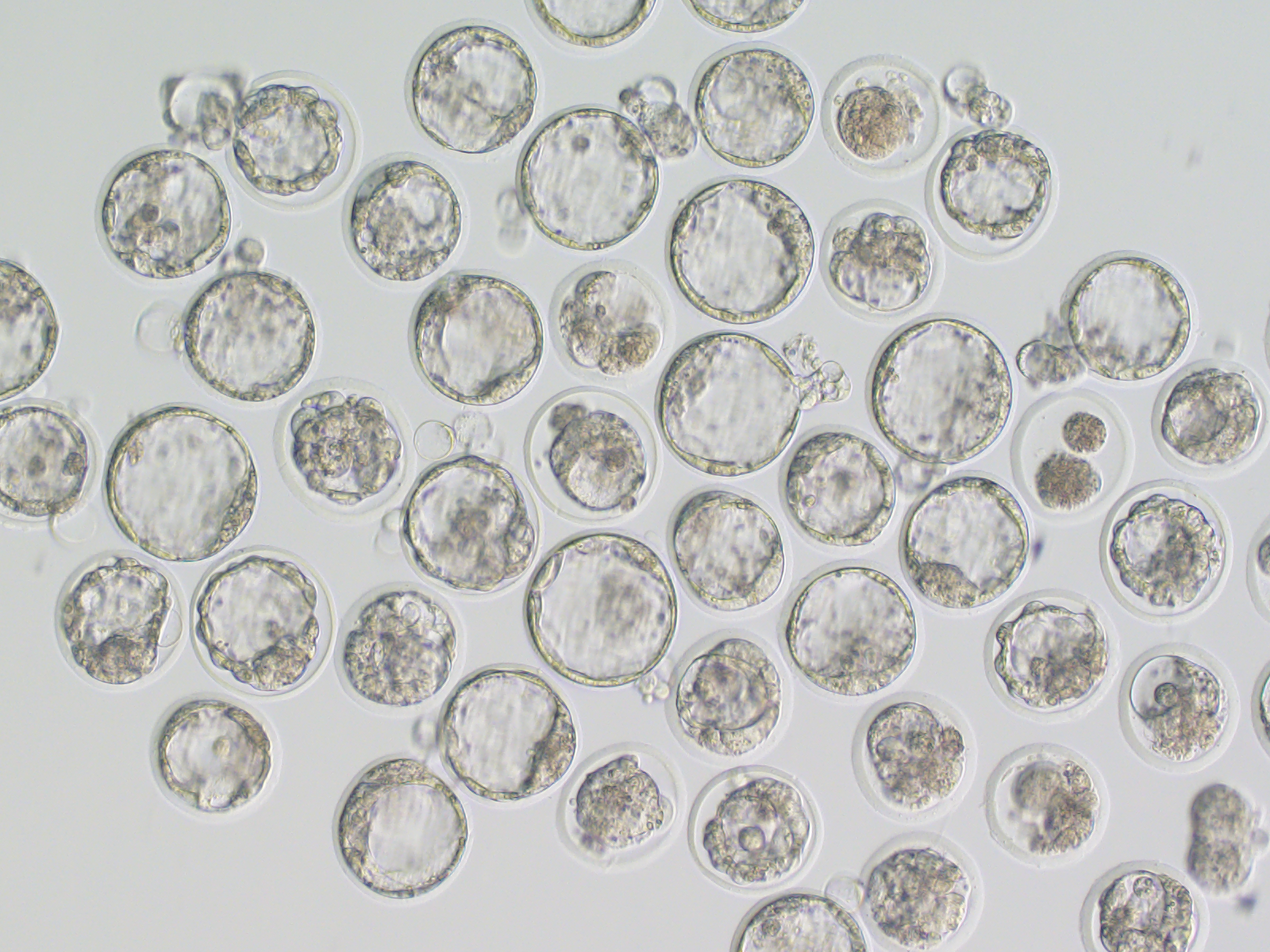

Supplement: Supplementary file 19 — Appendix Source Data [file 44319_2026_712_MOESM19_ESM.zip › Appendix source data/Appendix Figure S3/S3A/Ctrl_NT.tif]

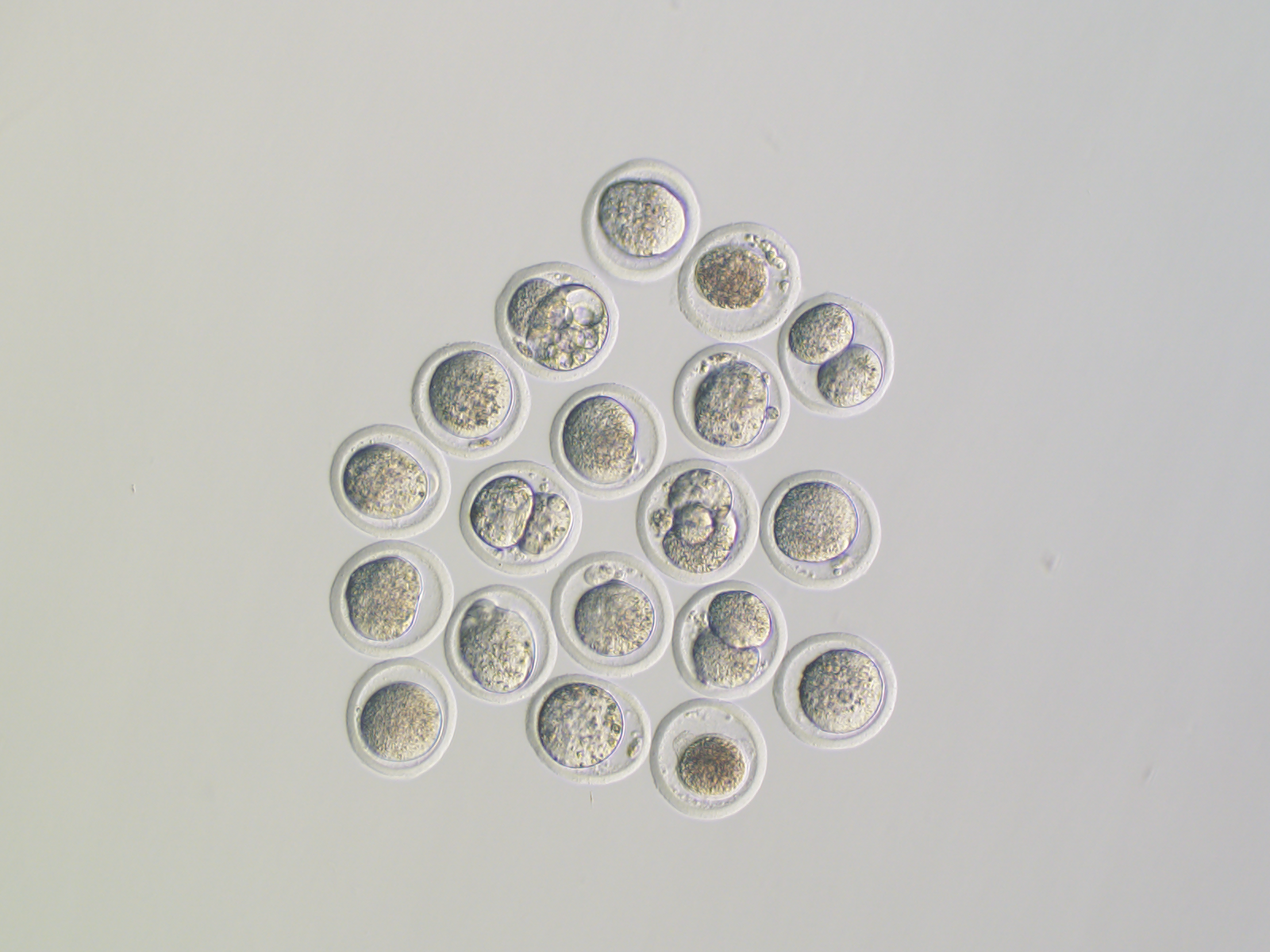

Supplement: Supplementary file 19 — Appendix Source Data [file 44319_2026_712_MOESM19_ESM.zip › Appendix source data/Appendix Figure S3/S3H/mKO_NT_PA106h_σë»μ£1⁄4.tif]

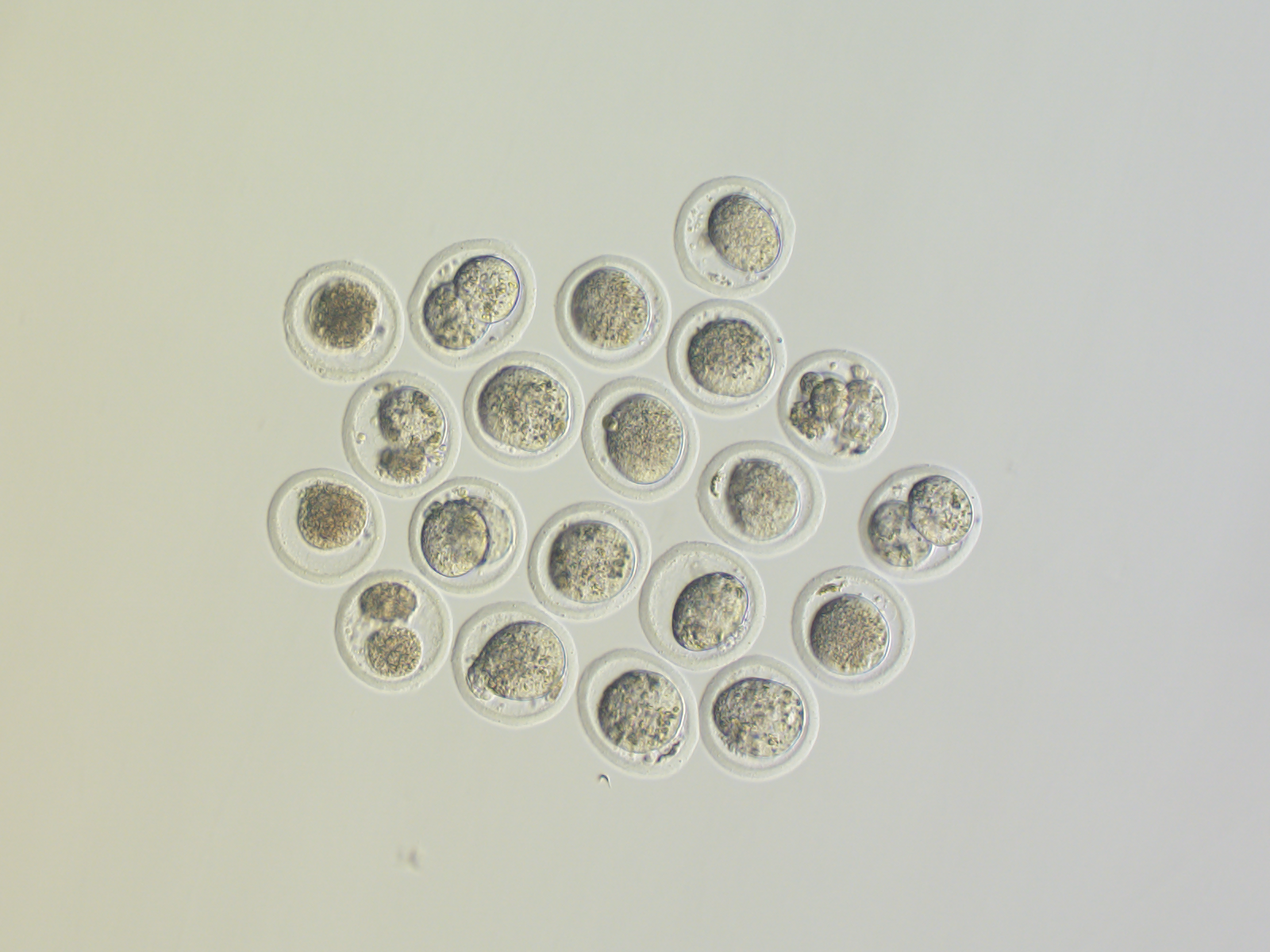

Supplement: Supplementary file 19 — Appendix Source Data [file 44319_2026_712_MOESM19_ESM.zip › Appendix source data/Appendix Figure S3/S3H/mKO_OE Thap1_PA106h_σë»μ£1⁄4.tif]

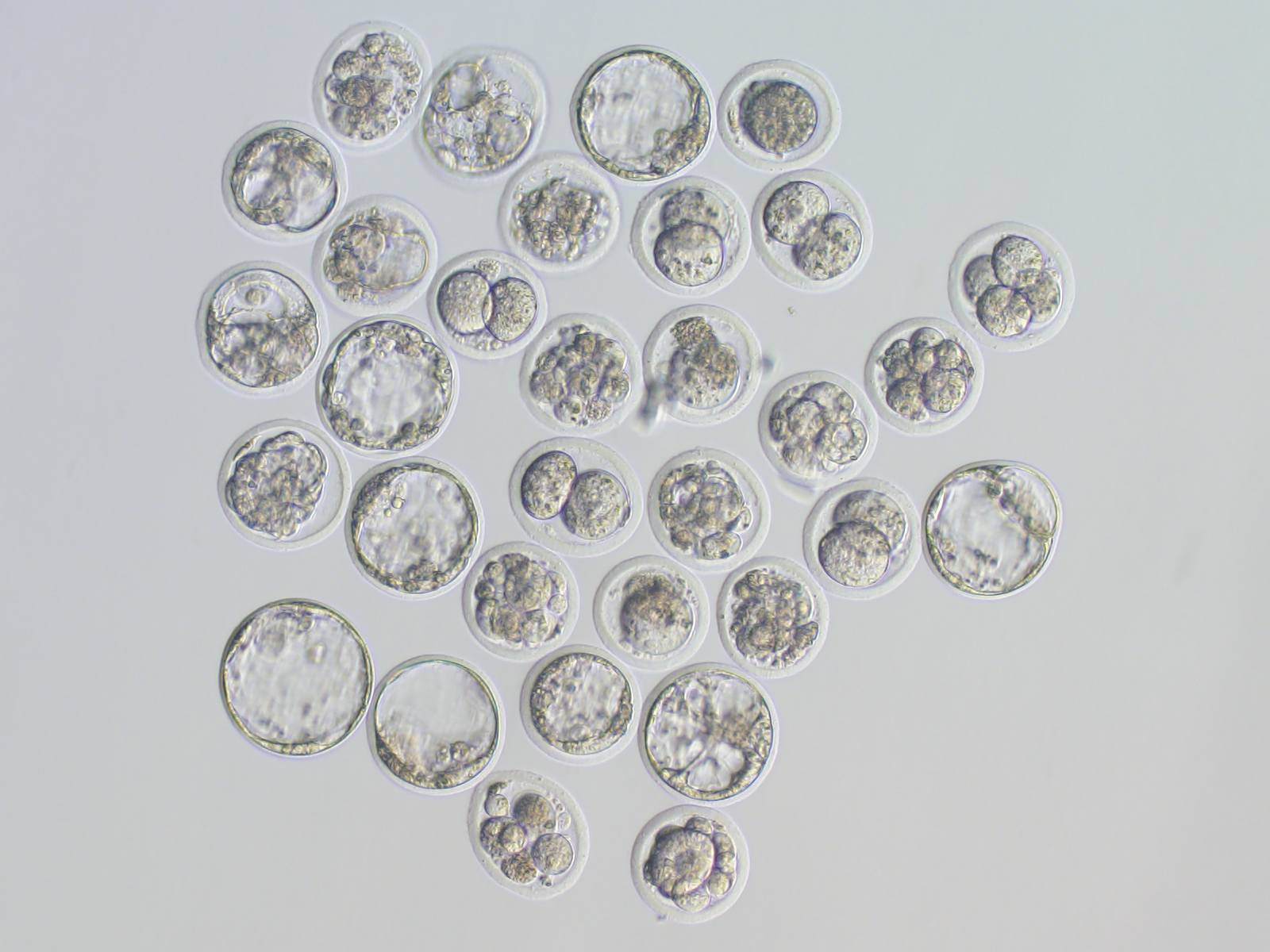

Supplement: Supplementary file 19 — Appendix Source Data [file 44319_2026_712_MOESM19_ESM.zip › Appendix source data/Appendix Figure S3/S3H/Ctrl_NT_PA106h_σë»μ£1⁄4.tif]

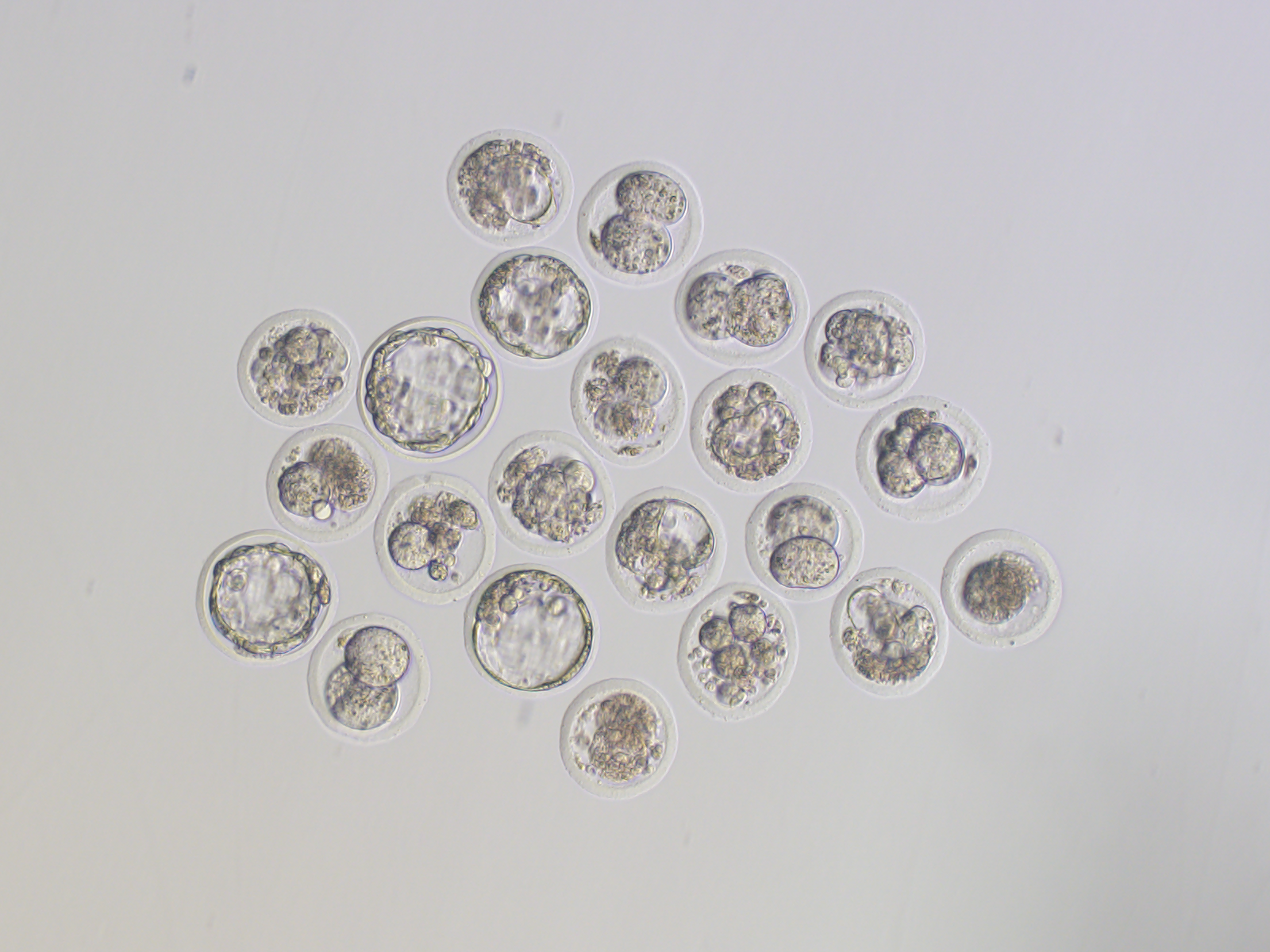

Supplement: Supplementary file 19 — Appendix Source Data [file 44319_2026_712_MOESM19_ESM.zip › Appendix source data/Appendix Figure S3/S3H/Ctrl_OE Thap1_PA106h_σë»μ£1⁄4.tif]

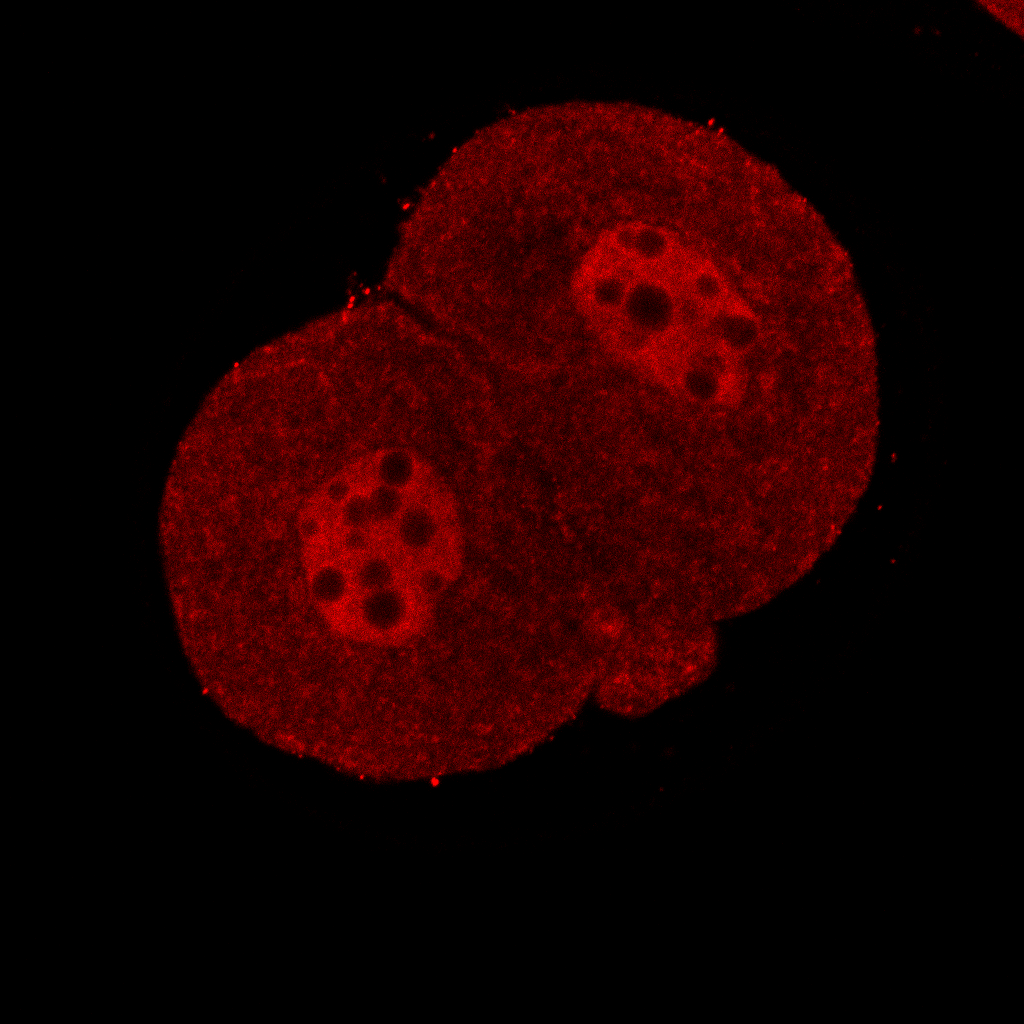

Supplement: Supplementary file 19 — Appendix Source Data [file 44319_2026_712_MOESM19_ESM.zip › Appendix source data/Appendix Figure S5/S5B/Ctrl_OE RRM1_RRM1.tif]

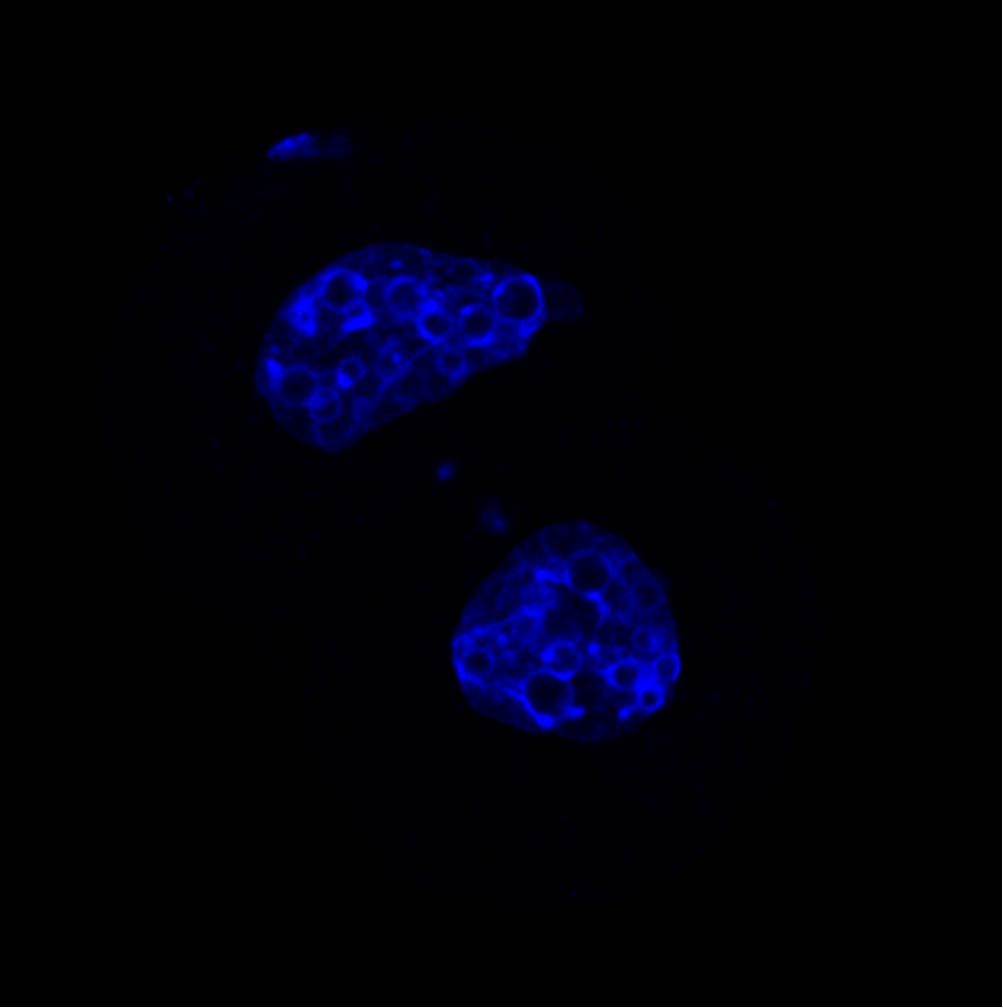

Supplement: Supplementary file 19 — Appendix Source Data [file 44319_2026_712_MOESM19_ESM.zip › Appendix source data/Appendix Figure S5/S5B/Ctrl_NT_DAPI.jpg]

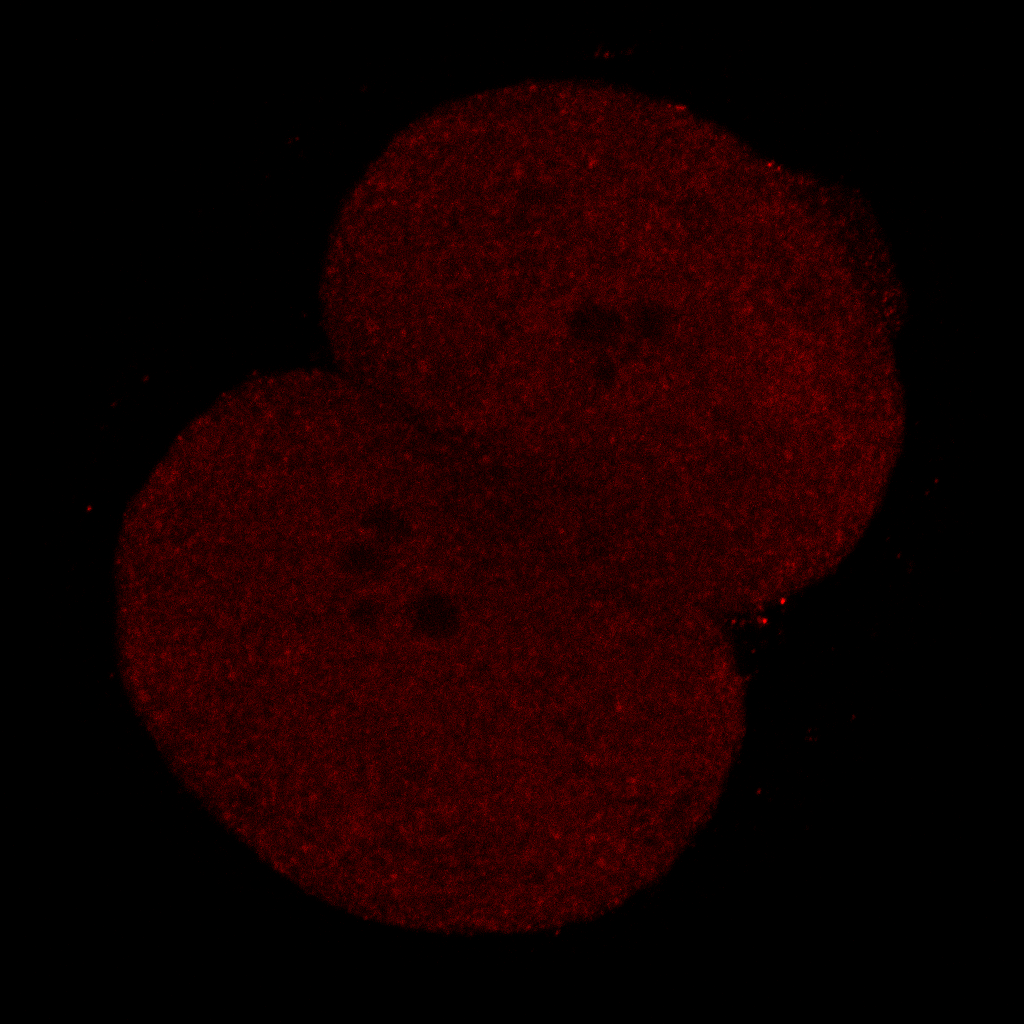

Supplement: Supplementary file 19 — Appendix Source Data [file 44319_2026_712_MOESM19_ESM.zip › Appendix source data/Appendix Figure S5/S5B/mKO_NT_RRM1.tif]

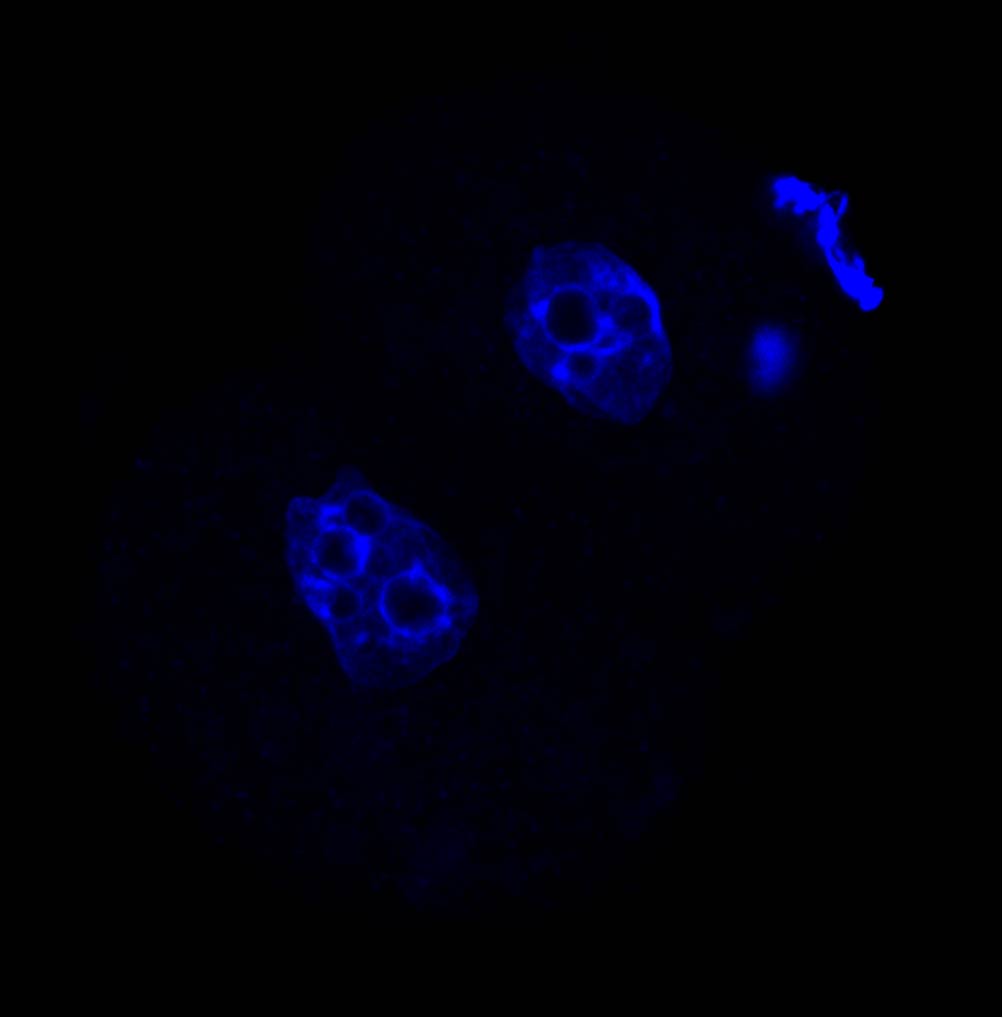

Supplement: Supplementary file 19 — Appendix Source Data [file 44319_2026_712_MOESM19_ESM.zip › Appendix source data/Appendix Figure S5/S5B/mKO_NT_DAPI.jpg]

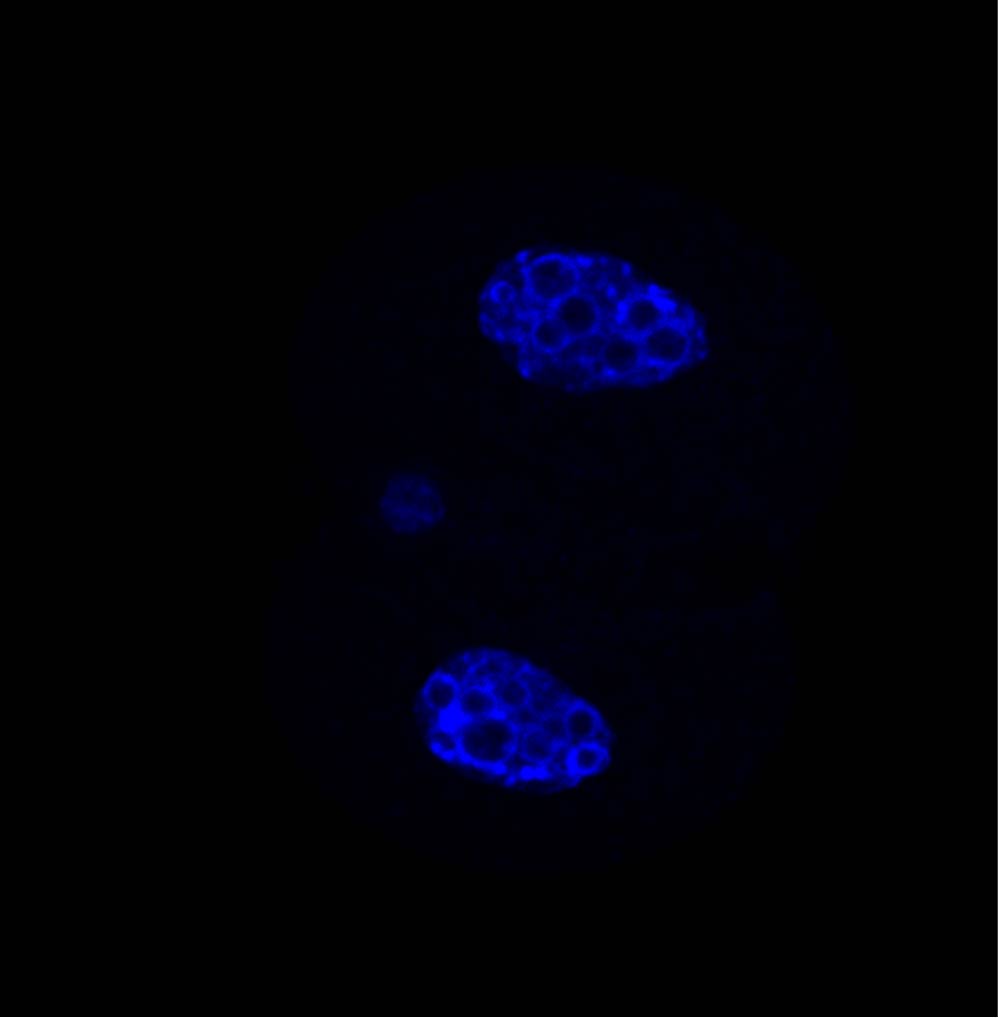

Supplement: Supplementary file 19 — Appendix Source Data [file 44319_2026_712_MOESM19_ESM.zip › Appendix source data/Appendix Figure S5/S5B/mKO_OE RRM1_DAPI.jpg]

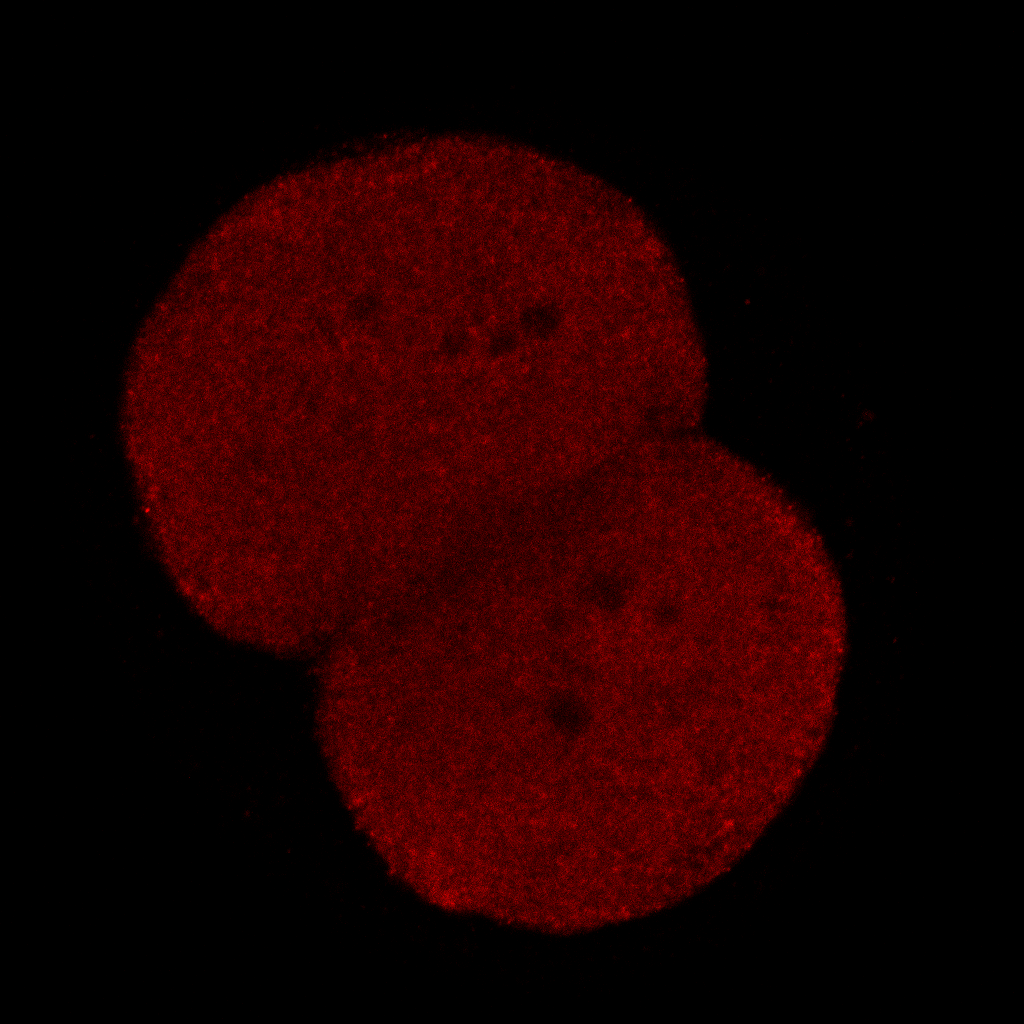

Supplement: Supplementary file 19 — Appendix Source Data [file 44319_2026_712_MOESM19_ESM.zip › Appendix source data/Appendix Figure S5/S5B/Ctrl_NT_RRM1.tif]

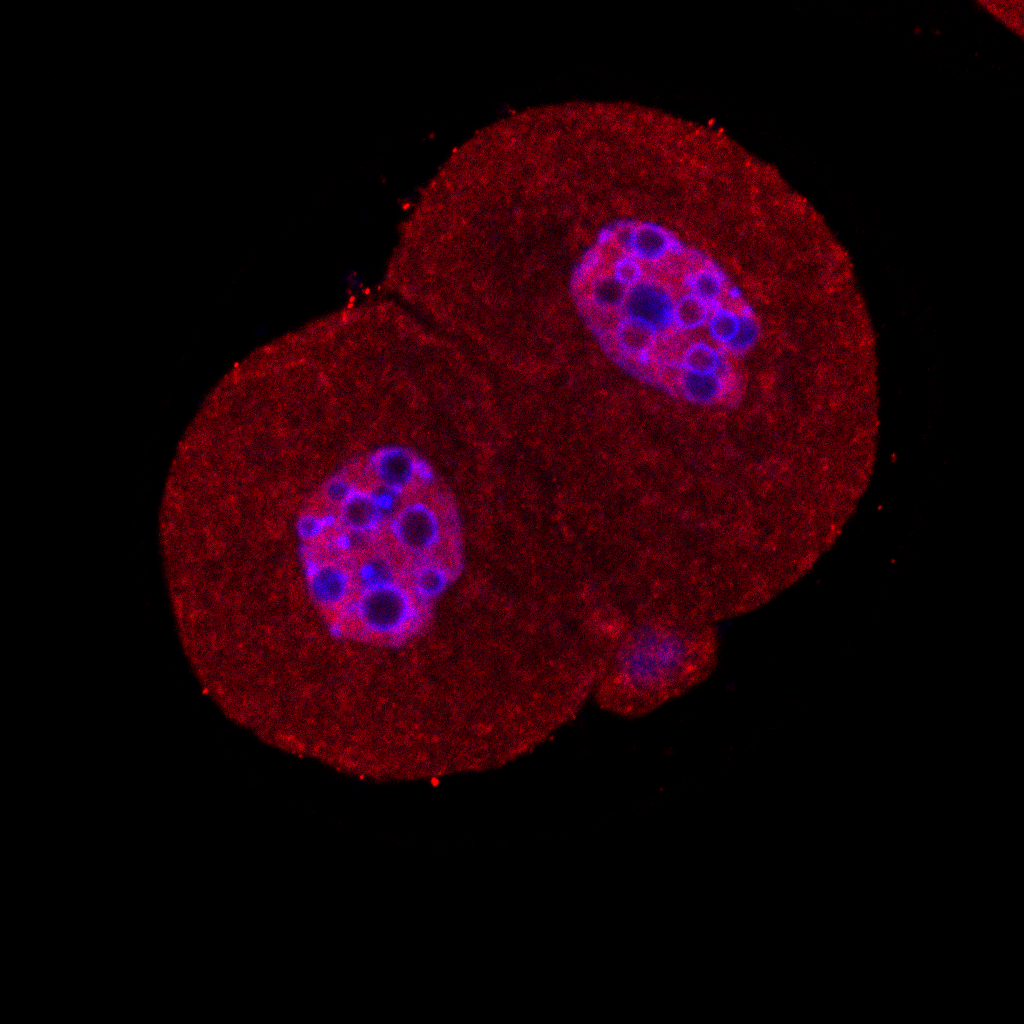

Supplement: Supplementary file 19 — Appendix Source Data [file 44319_2026_712_MOESM19_ESM.zip › Appendix source data/Appendix Figure S5/S5B/Ctrl_OE RRM1_Merge.tif]

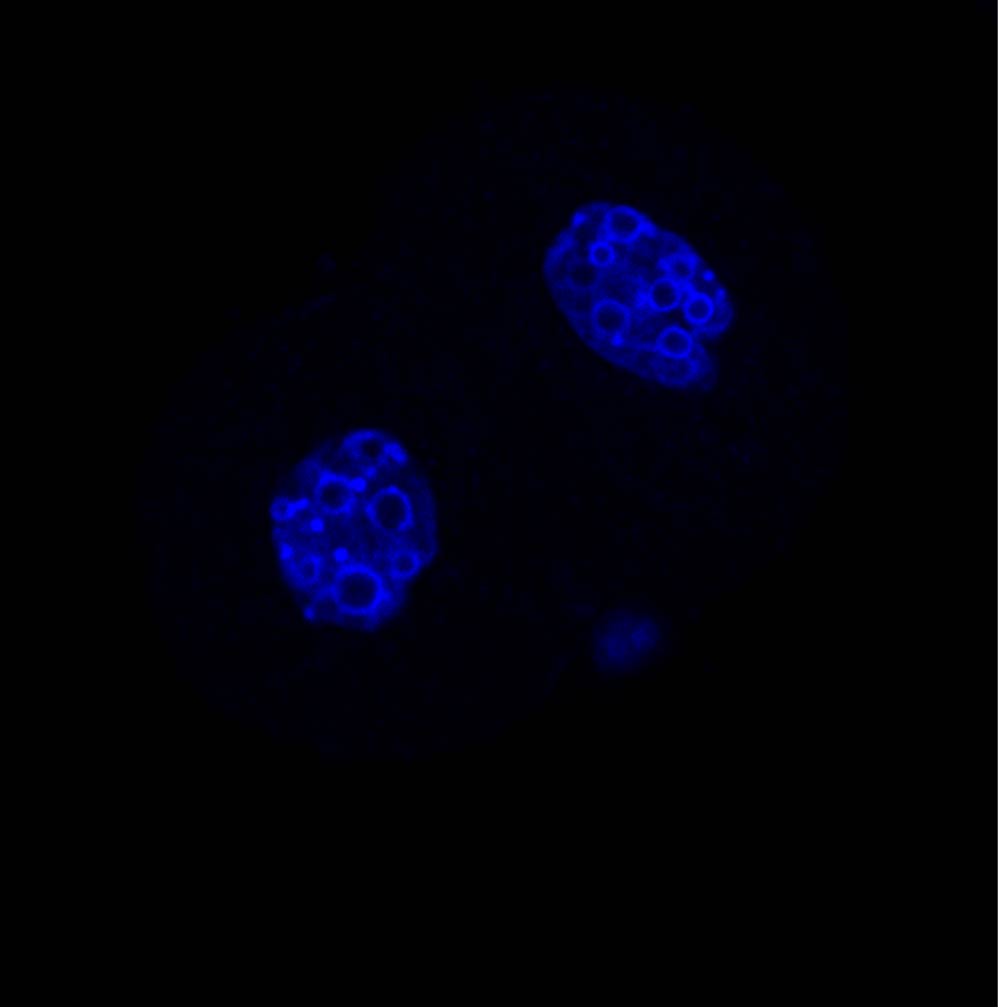

Supplement: Supplementary file 19 — Appendix Source Data [file 44319_2026_712_MOESM19_ESM.zip › Appendix source data/Appendix Figure S5/S5B/Ctrl_OE RRM1_DAPI.jpg]

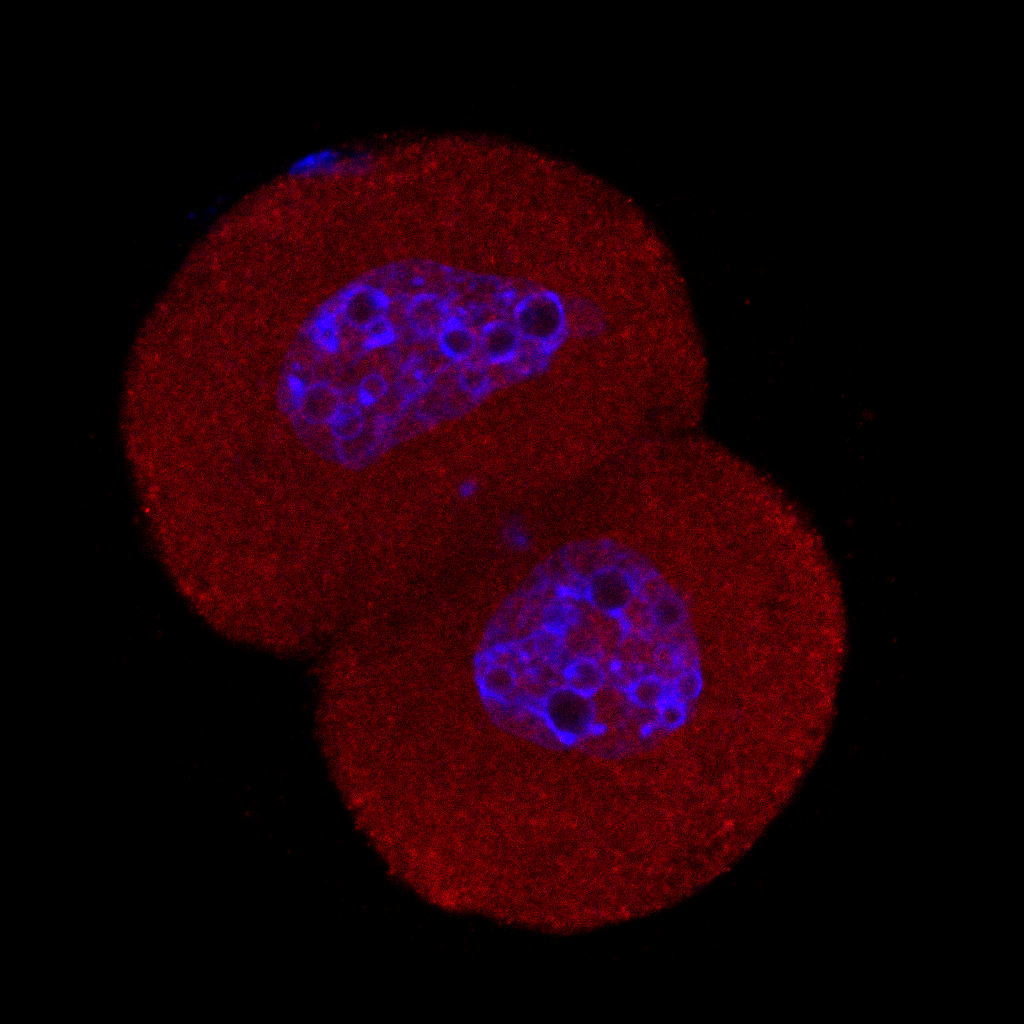

Supplement: Supplementary file 19 — Appendix Source Data [file 44319_2026_712_MOESM19_ESM.zip › Appendix source data/Appendix Figure S5/S5B/Ctrl_NT_Merge.tif]

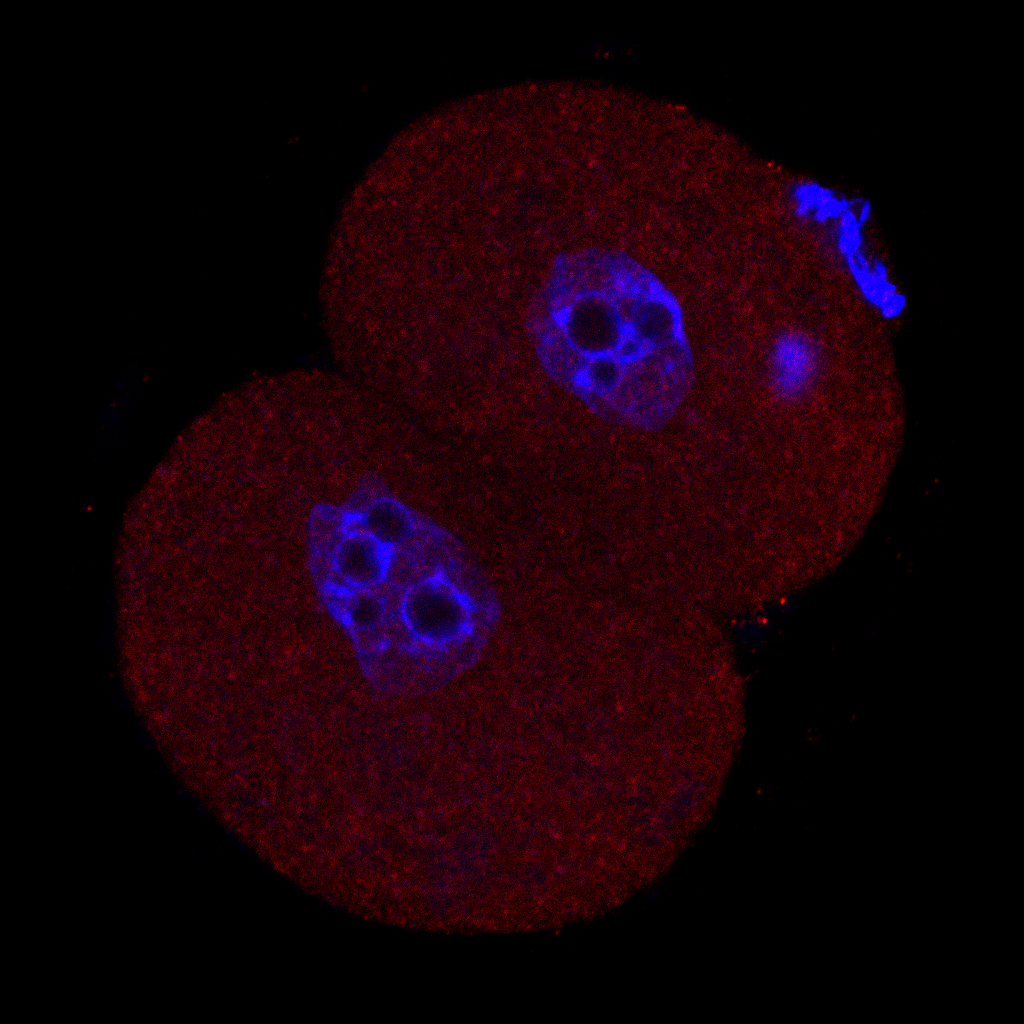

Supplement: Supplementary file 19 — Appendix Source Data [file 44319_2026_712_MOESM19_ESM.zip › Appendix source data/Appendix Figure S5/S5B/mKO_NT_Merge.tif]

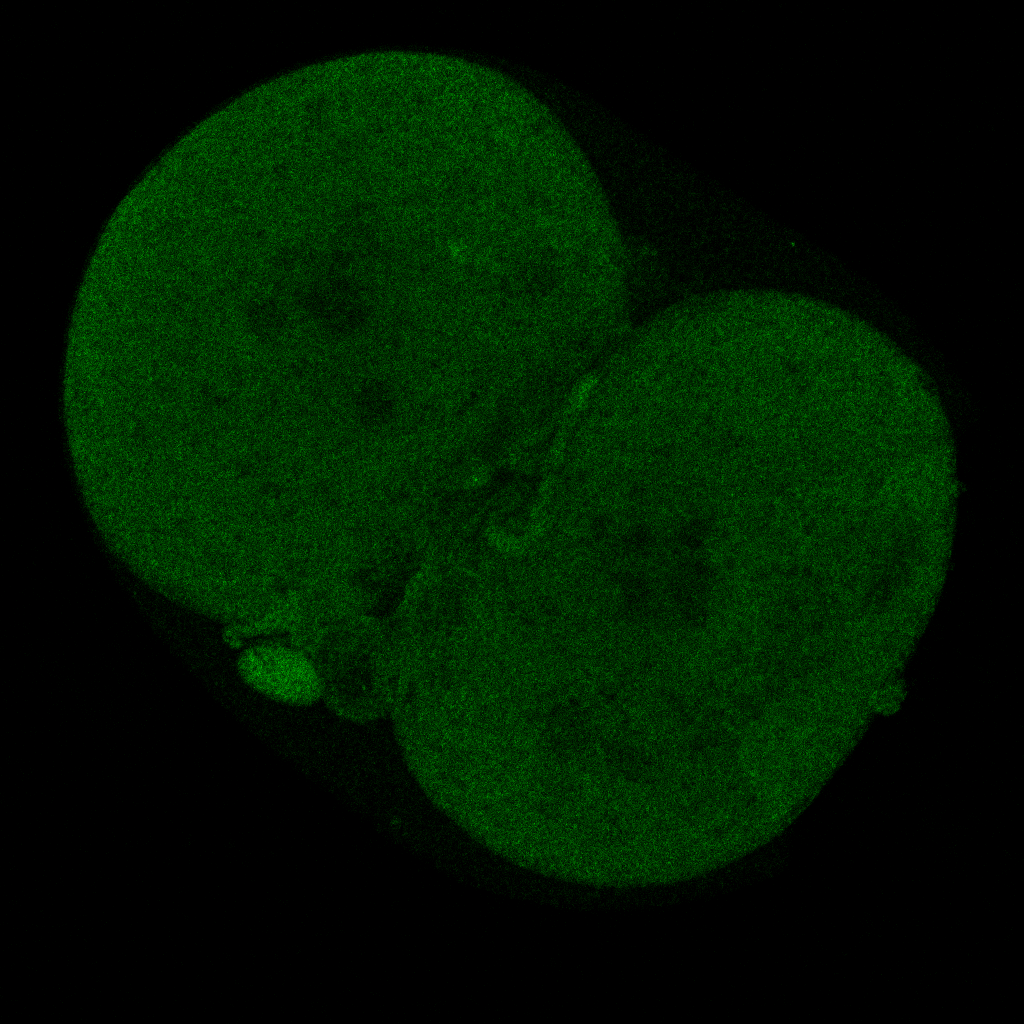

Supplement: Supplementary file 19 — Appendix Source Data [file 44319_2026_712_MOESM19_ESM.zip › Appendix source data/Appendix Figure S3/S3B/mKO-NT-L2C/mKO_NT_L2C_FLAG.tif]

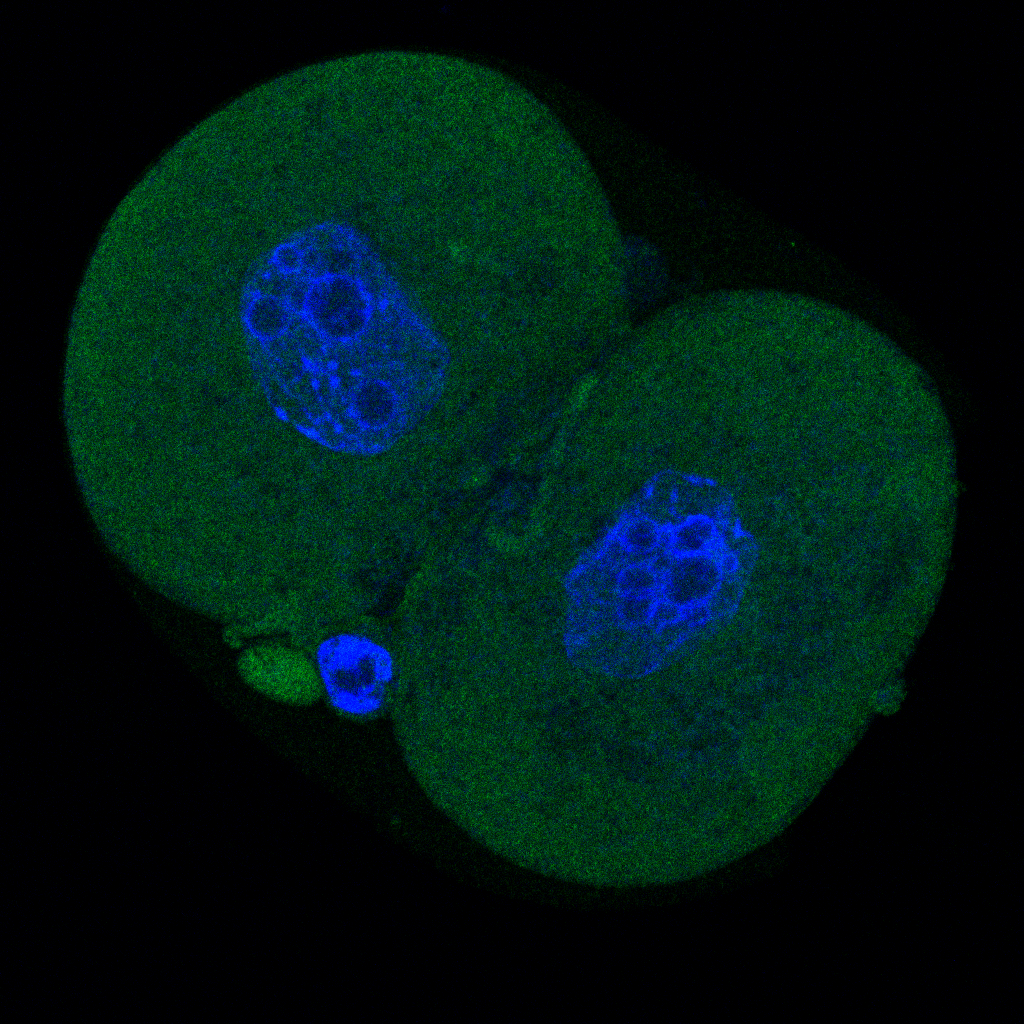

Supplement: Supplementary file 19 — Appendix Source Data [file 44319_2026_712_MOESM19_ESM.zip › Appendix source data/Appendix Figure S3/S3B/mKO-NT-L2C/mKO_NT_L2C_Merge.tif]

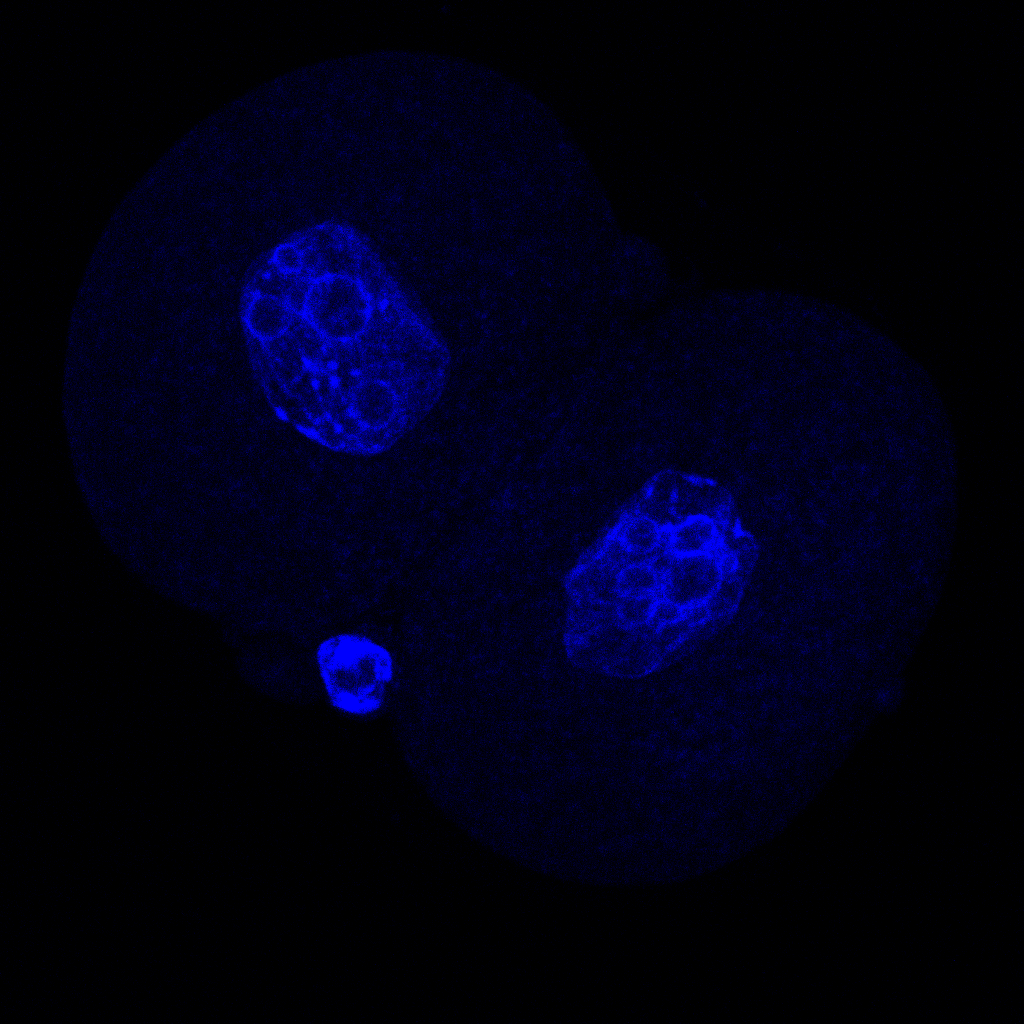

Supplement: Supplementary file 19 — Appendix Source Data [file 44319_2026_712_MOESM19_ESM.zip › Appendix source data/Appendix Figure S3/S3B/mKO-NT-L2C/mKO_NT_L2C_DAPI.tif]

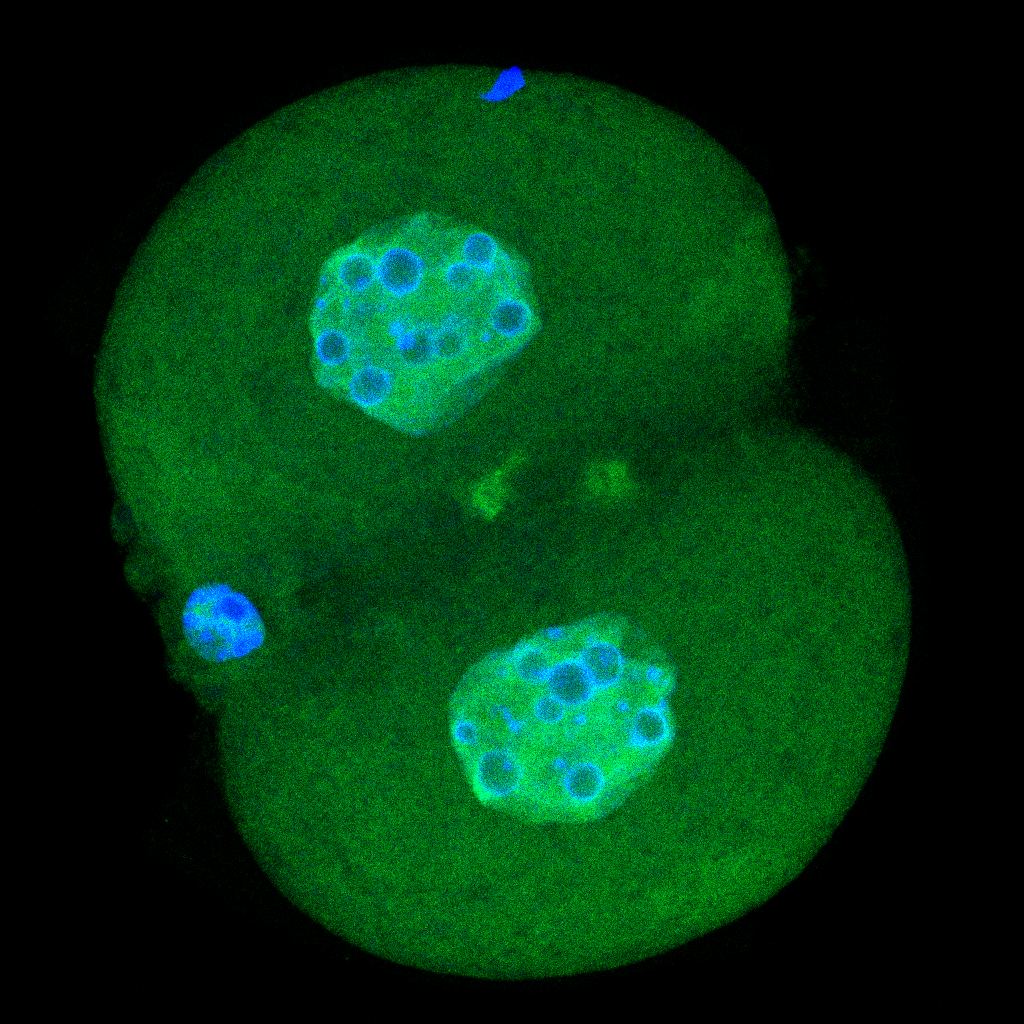

Supplement: Supplementary file 19 — Appendix Source Data [file 44319_2026_712_MOESM19_ESM.zip › Appendix source data/Appendix Figure S3/S3B/Ctrl-oe-FLAG THAP1_L2C/Ctrl_OE FLAG THAP1_Merge.tif]

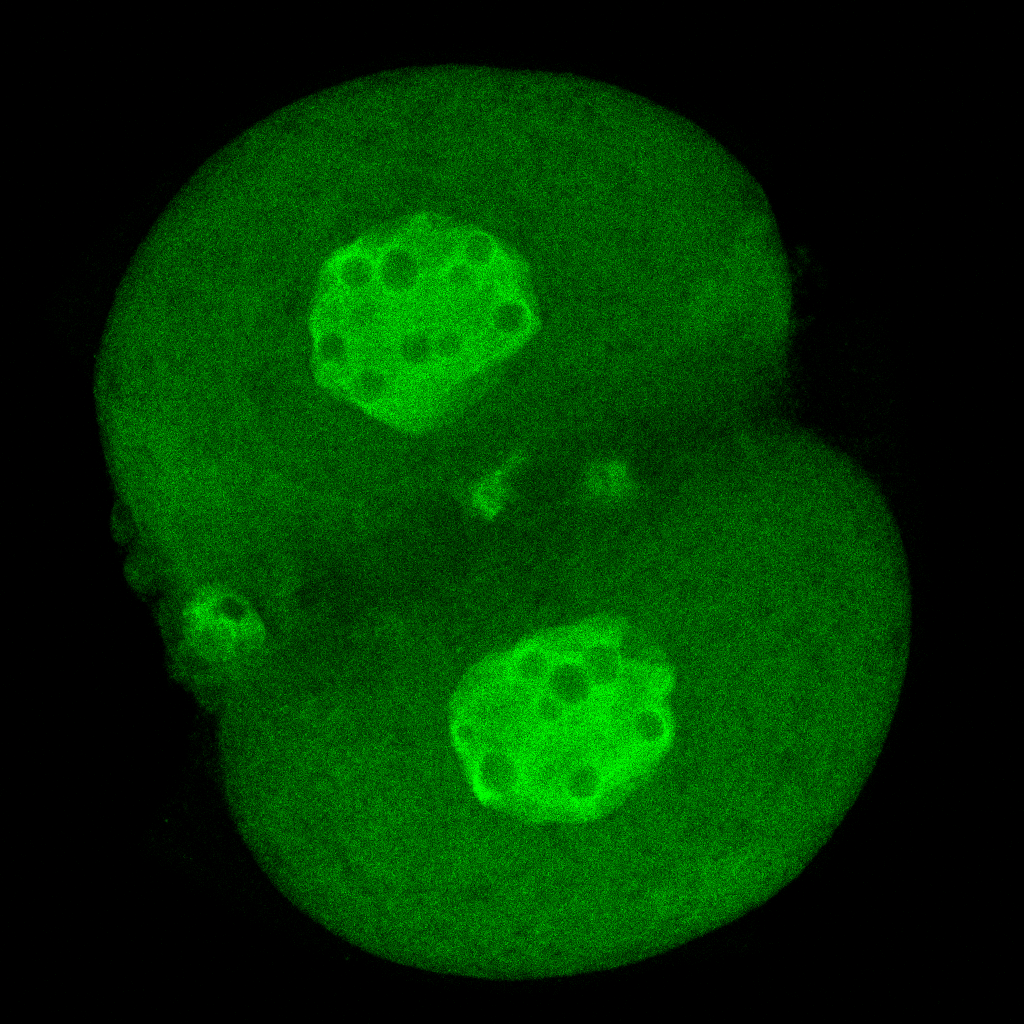

Supplement: Supplementary file 19 — Appendix Source Data [file 44319_2026_712_MOESM19_ESM.zip › Appendix source data/Appendix Figure S3/S3B/Ctrl-oe-FLAG THAP1_L2C/Ctrl_OE FLAG THAP1_FLAG.tif]

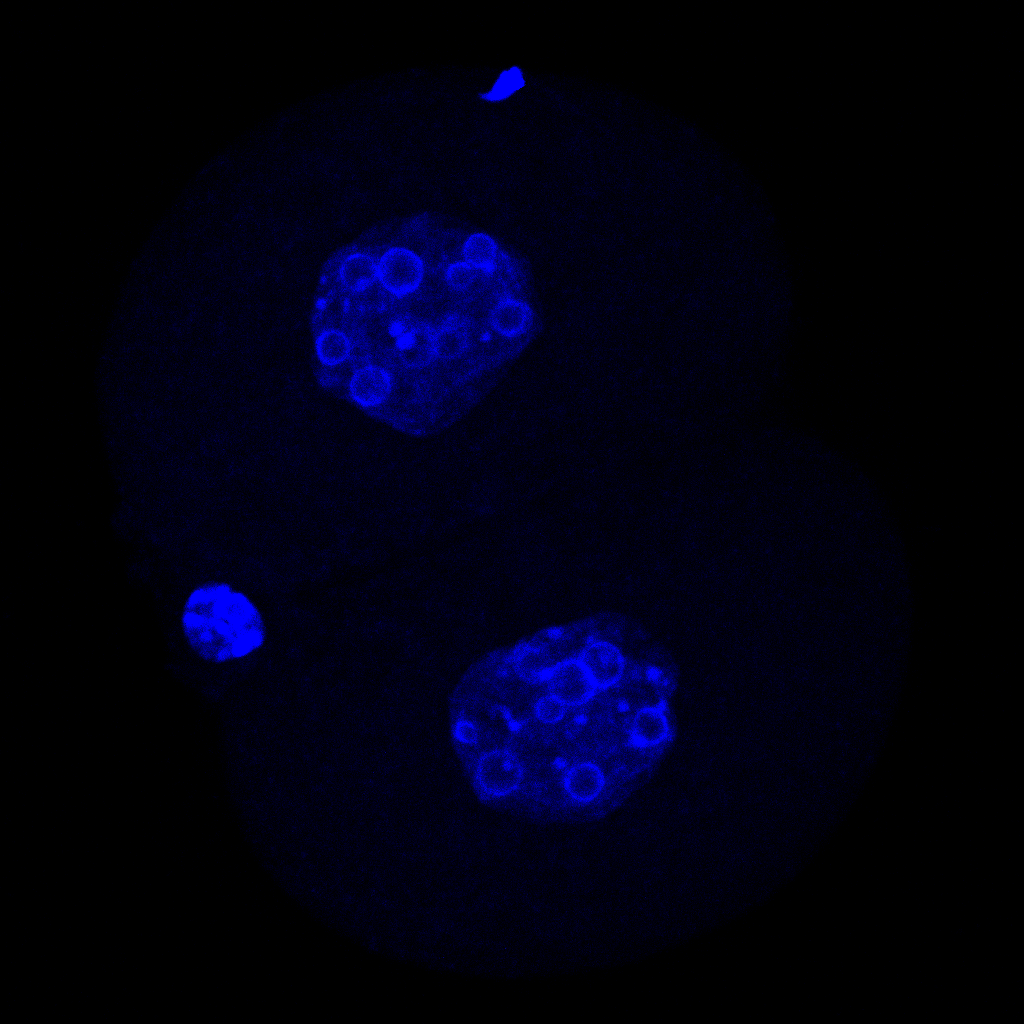

Supplement: Supplementary file 19 — Appendix Source Data [file 44319_2026_712_MOESM19_ESM.zip › Appendix source data/Appendix Figure S3/S3B/Ctrl-oe-FLAG THAP1_L2C/Ctrl_OE FLAG THAP1_DAPI.tif]

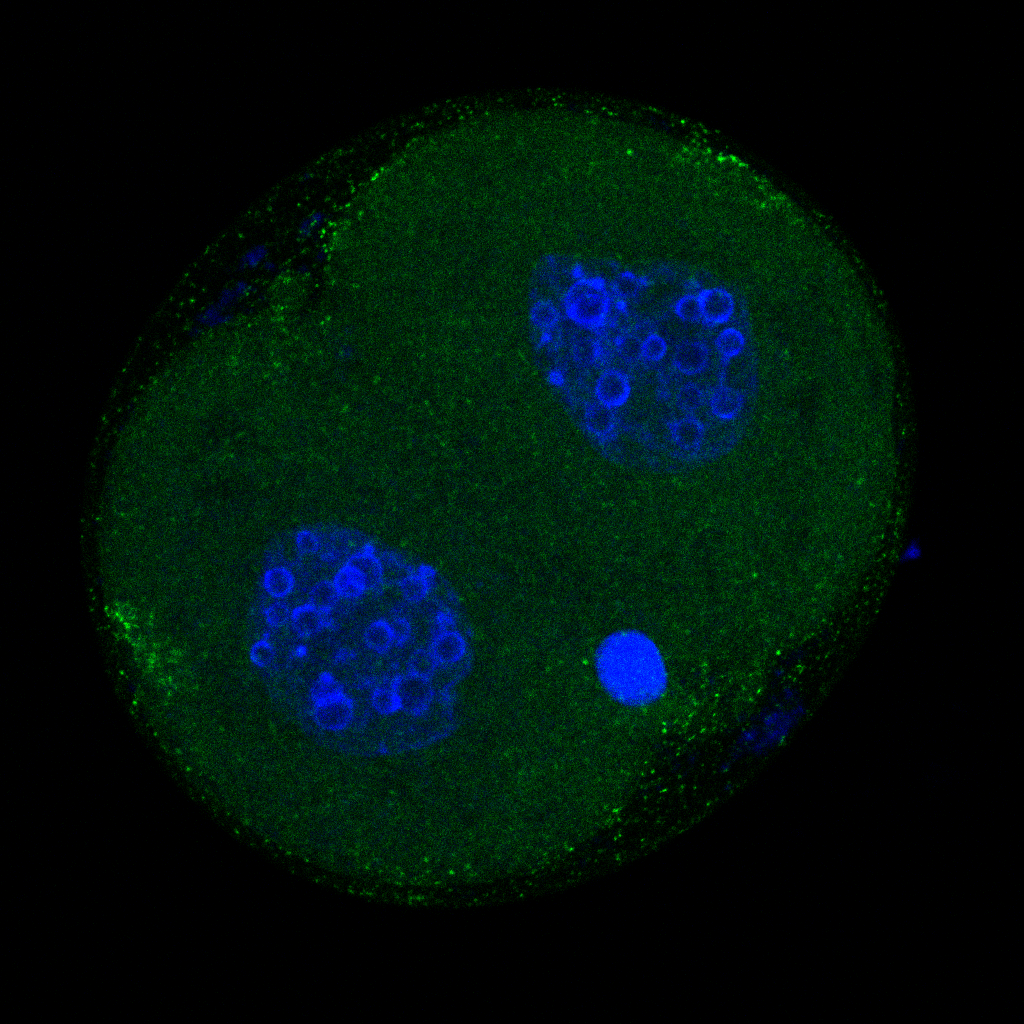

Supplement: Supplementary file 19 — Appendix Source Data [file 44319_2026_712_MOESM19_ESM.zip › Appendix source data/Appendix Figure S3/S3B/Ctrl-NT-L2C/Ctrl_NT_L2C_Merge.tif]

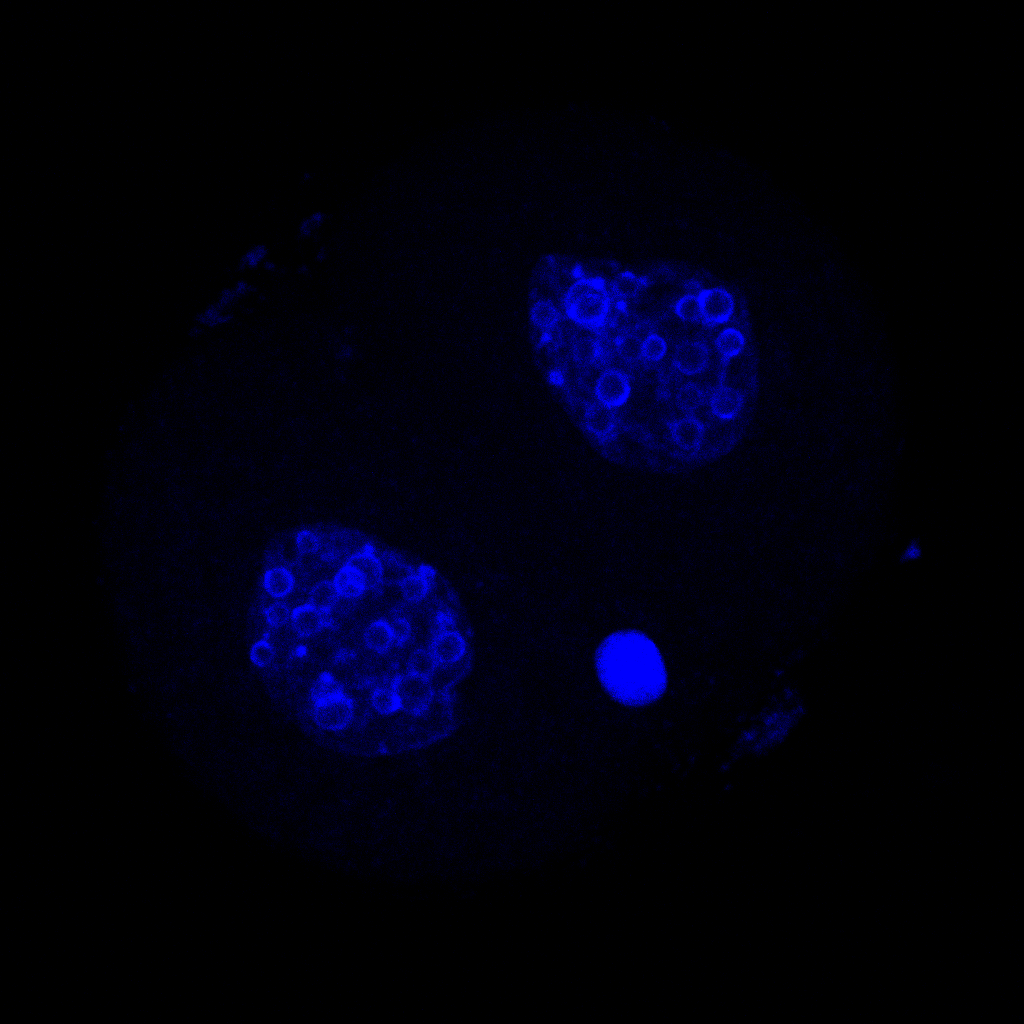

Supplement: Supplementary file 19 — Appendix Source Data [file 44319_2026_712_MOESM19_ESM.zip › Appendix source data/Appendix Figure S3/S3B/Ctrl-NT-L2C/Ctrl_NT_L2C_DAPI.tif]

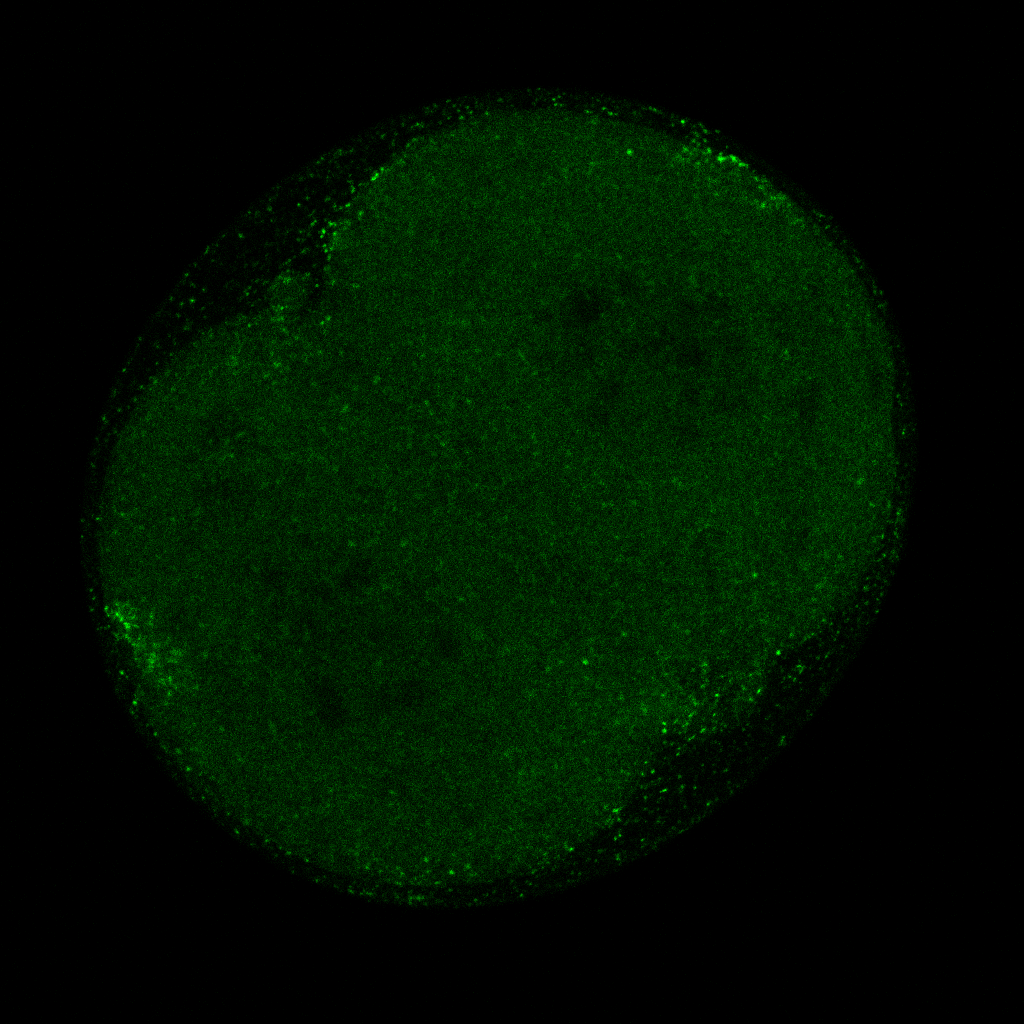

Supplement: Supplementary file 19 — Appendix Source Data [file 44319_2026_712_MOESM19_ESM.zip › Appendix source data/Appendix Figure S3/S3B/Ctrl-NT-L2C/Ctrl_NT_L2C_FLAG.tif]

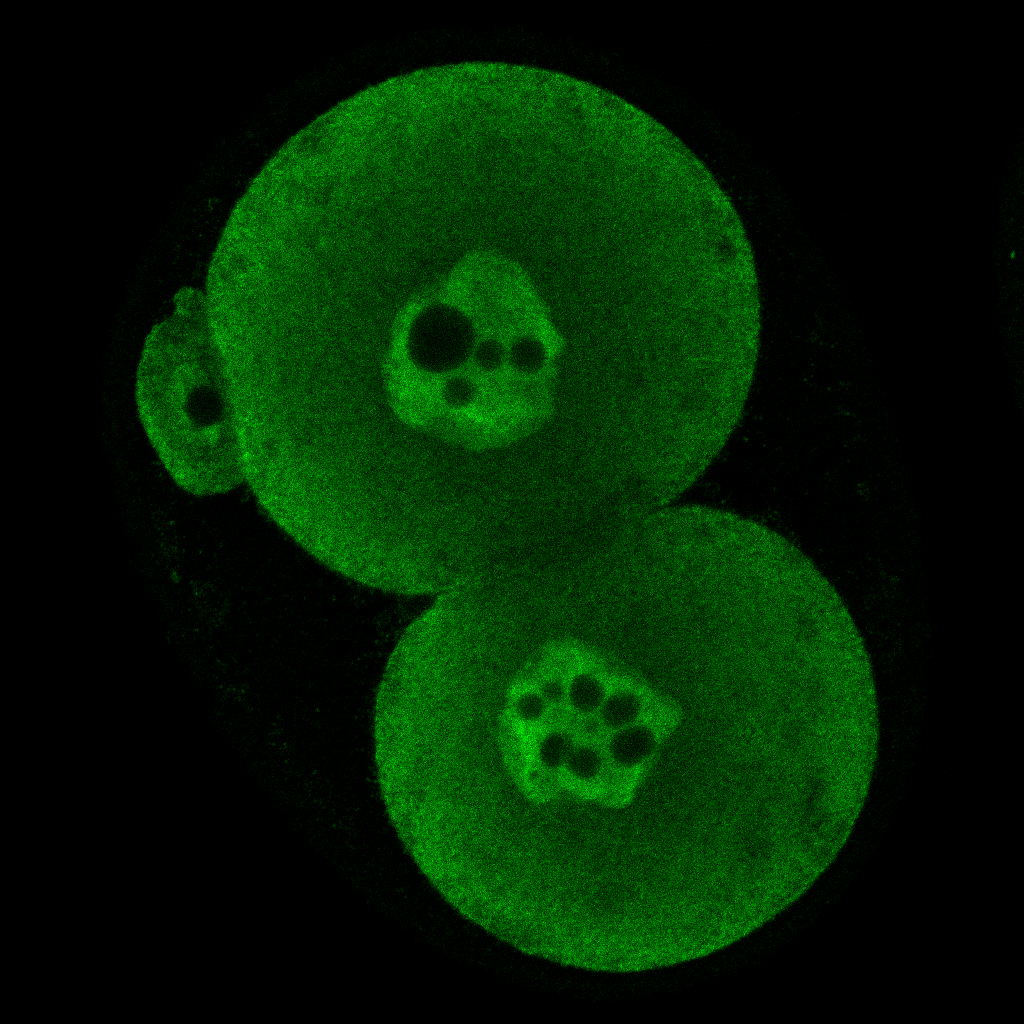

Supplement: Supplementary file 19 — Appendix Source Data [file 44319_2026_712_MOESM19_ESM.zip › Appendix source data/Appendix Figure S3/S3B/mKO-oe FLAG-THAP1-L2C/mKO_OE FLAG THAP1_FLAG.tif]

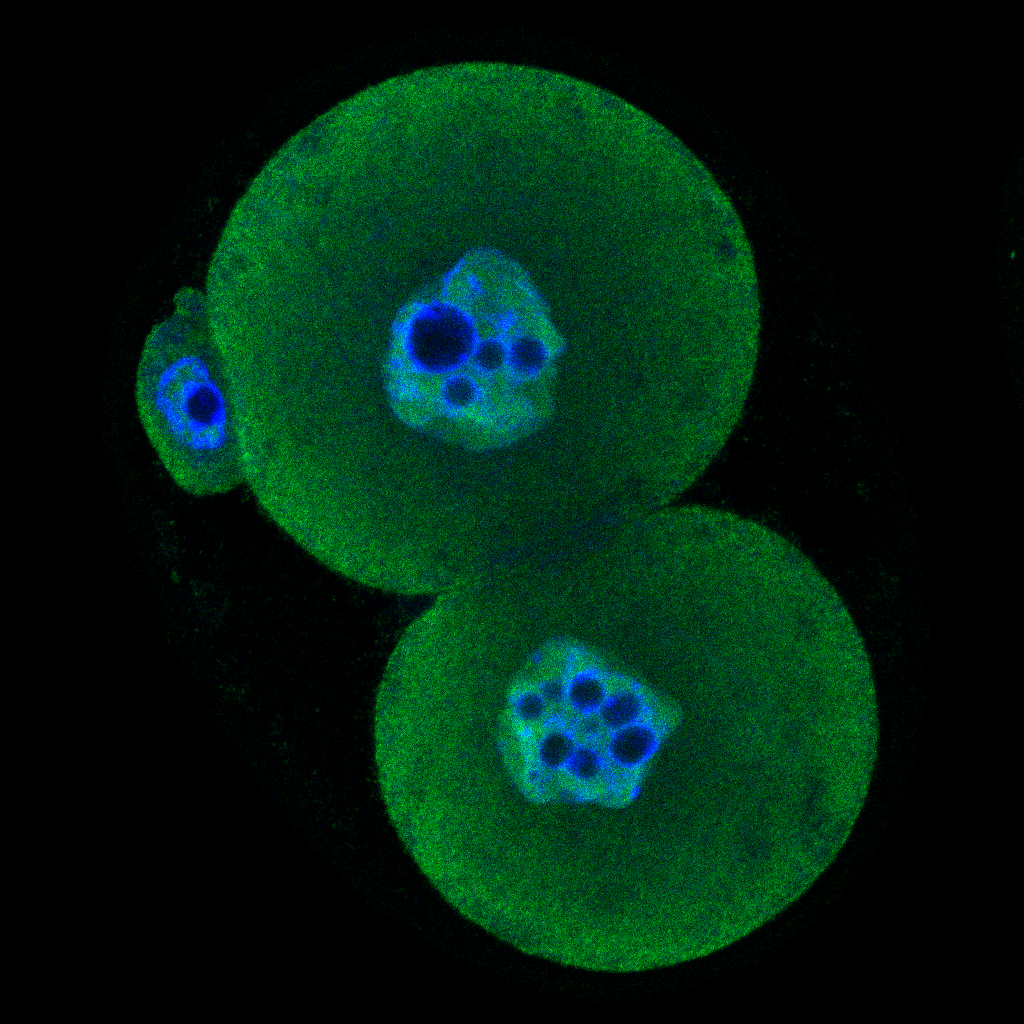

Supplement: Supplementary file 19 — Appendix Source Data [file 44319_2026_712_MOESM19_ESM.zip › Appendix source data/Appendix Figure S3/S3B/mKO-oe FLAG-THAP1-L2C/mKO_OE FLAG THAP1_Merge.tif]

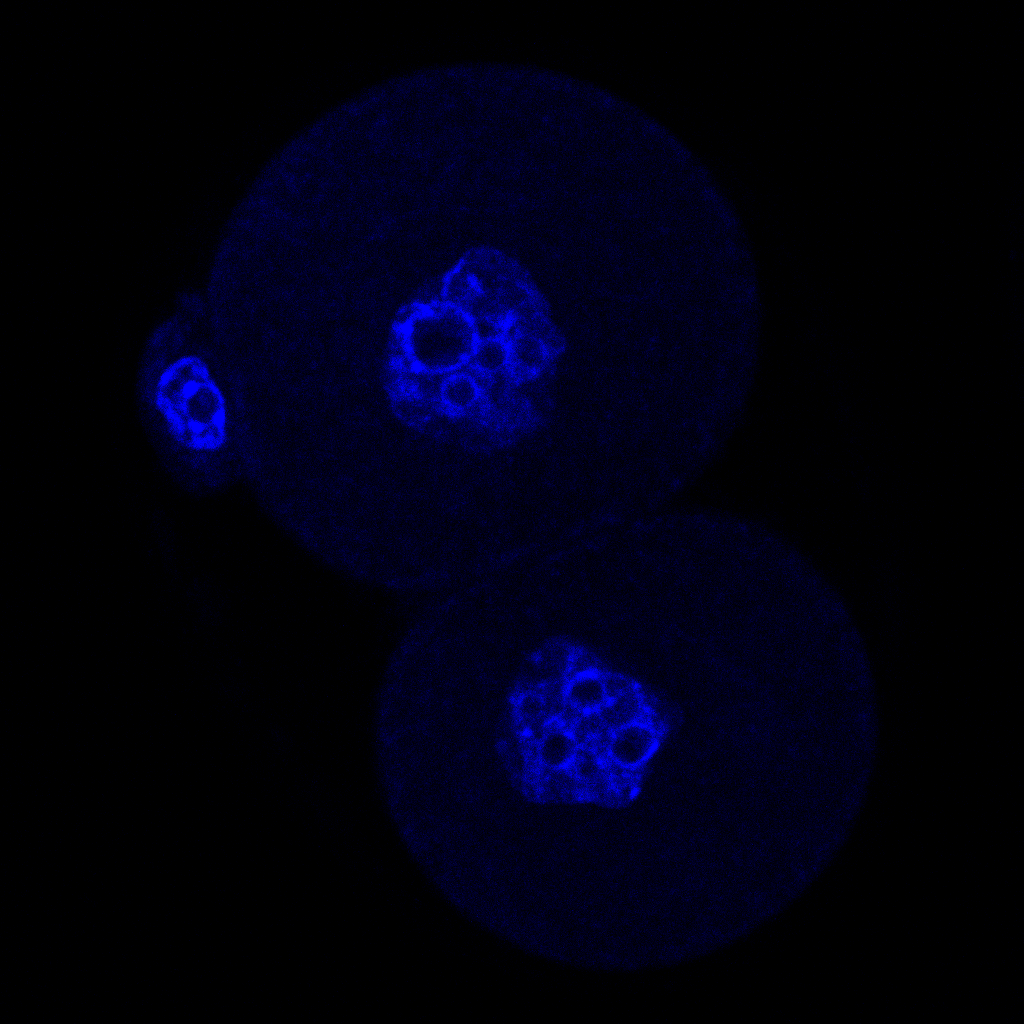

Supplement: Supplementary file 19 — Appendix Source Data [file 44319_2026_712_MOESM19_ESM.zip › Appendix source data/Appendix Figure S3/S3B/mKO-oe FLAG-THAP1-L2C/mKO_OE FLAG THAP1_DAPI.tif]

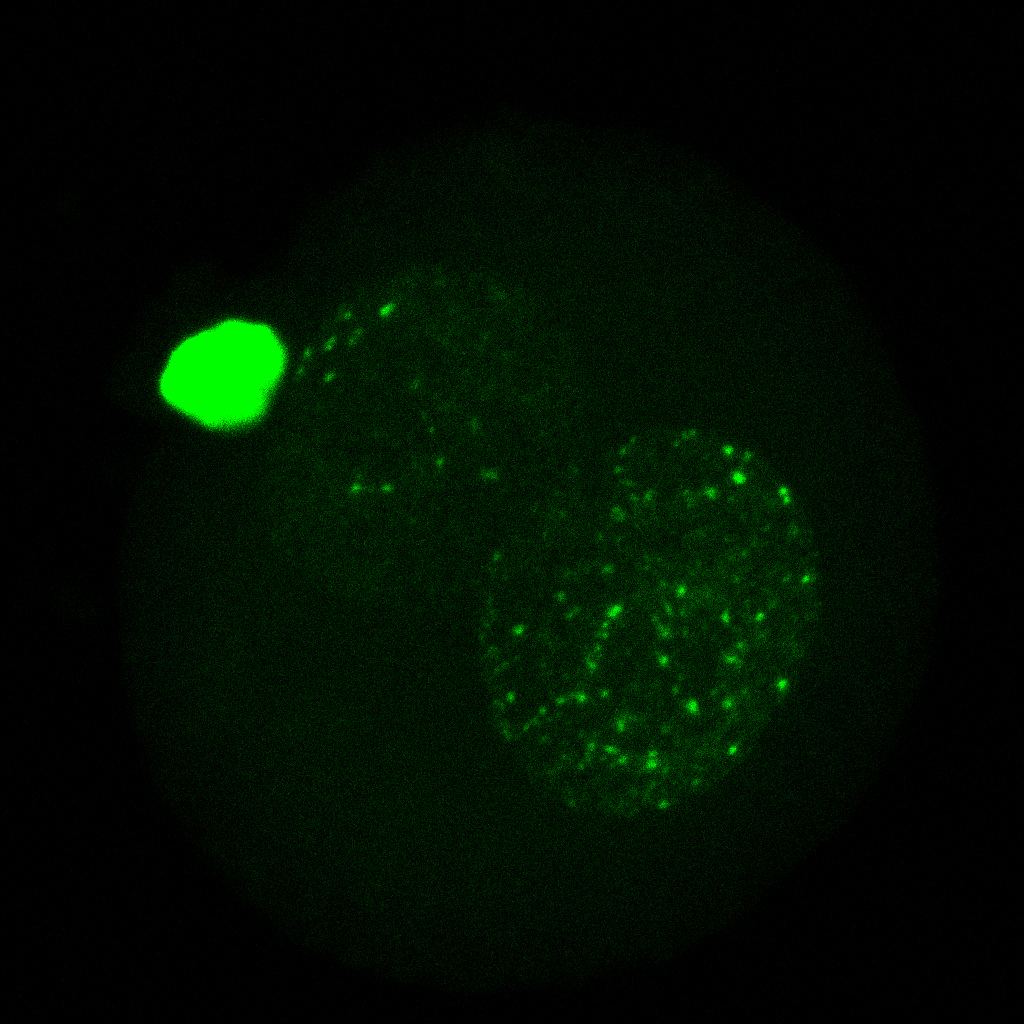

Supplement: Supplementary file 19 — Appendix Source Data [file 44319_2026_712_MOESM19_ESM.zip › Appendix source data/Appendix Figure S2/S2A/Post-S/╬│H2AX.tif]

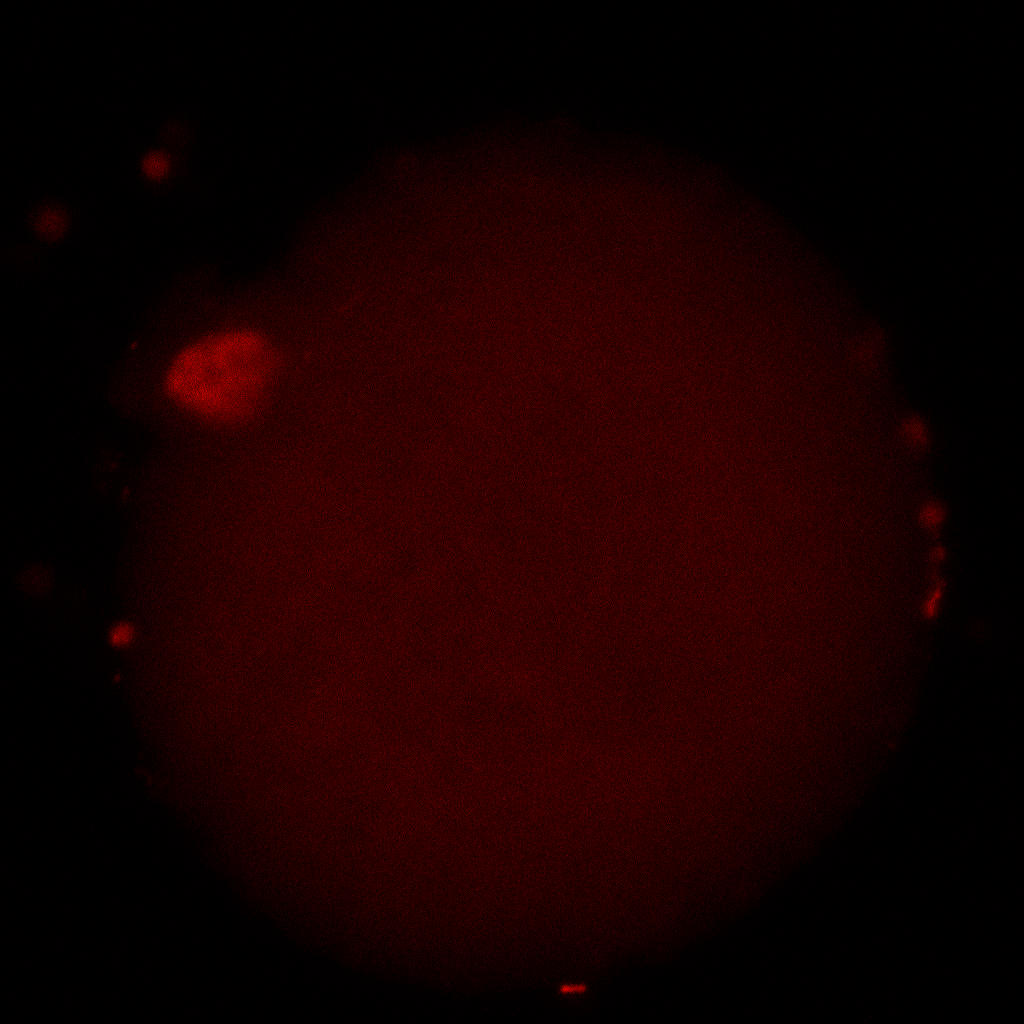

Supplement: Supplementary file 19 — Appendix Source Data [file 44319_2026_712_MOESM19_ESM.zip › Appendix source data/Appendix Figure S2/S2A/Post-S/EdU.tif]

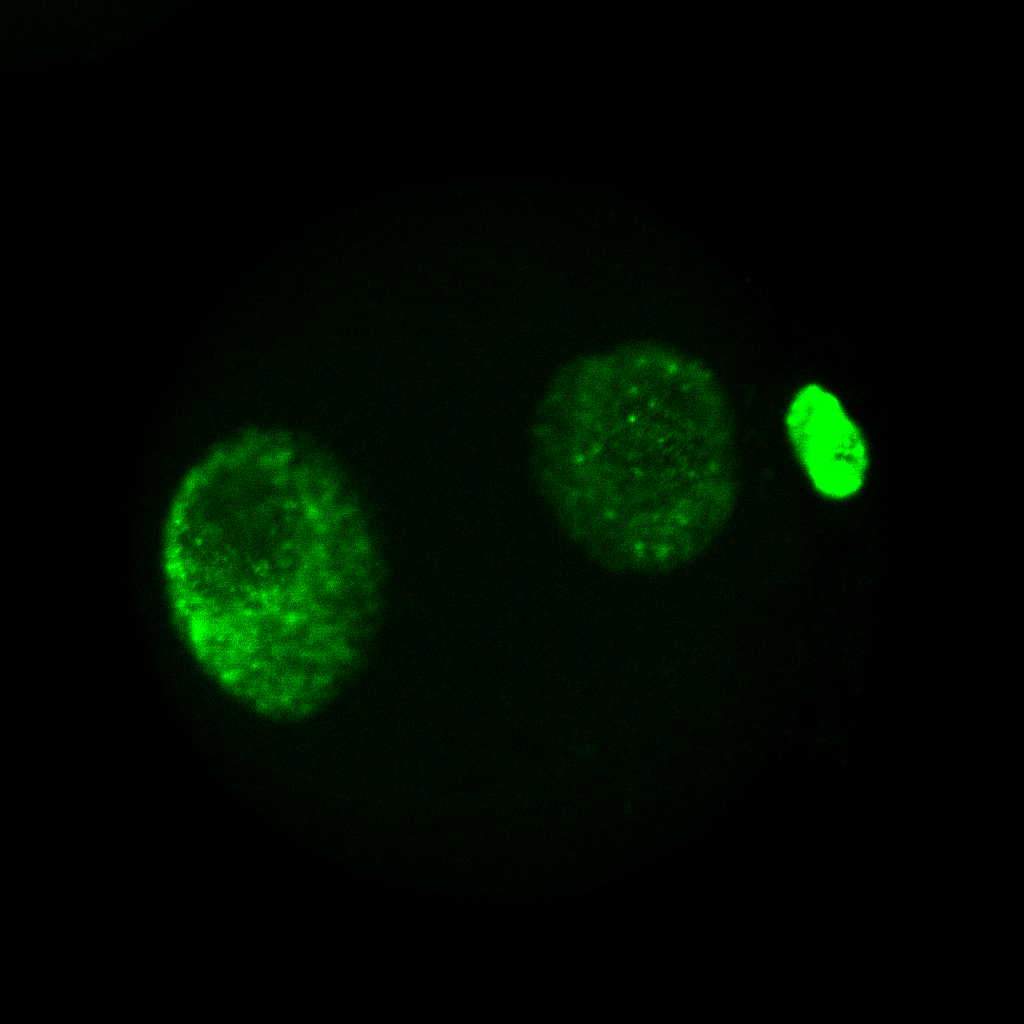

Supplement: Supplementary file 19 — Appendix Source Data [file 44319_2026_712_MOESM19_ESM.zip › Appendix source data/Appendix Figure S2/S2A/Early-S/╬│H2AX.tif]

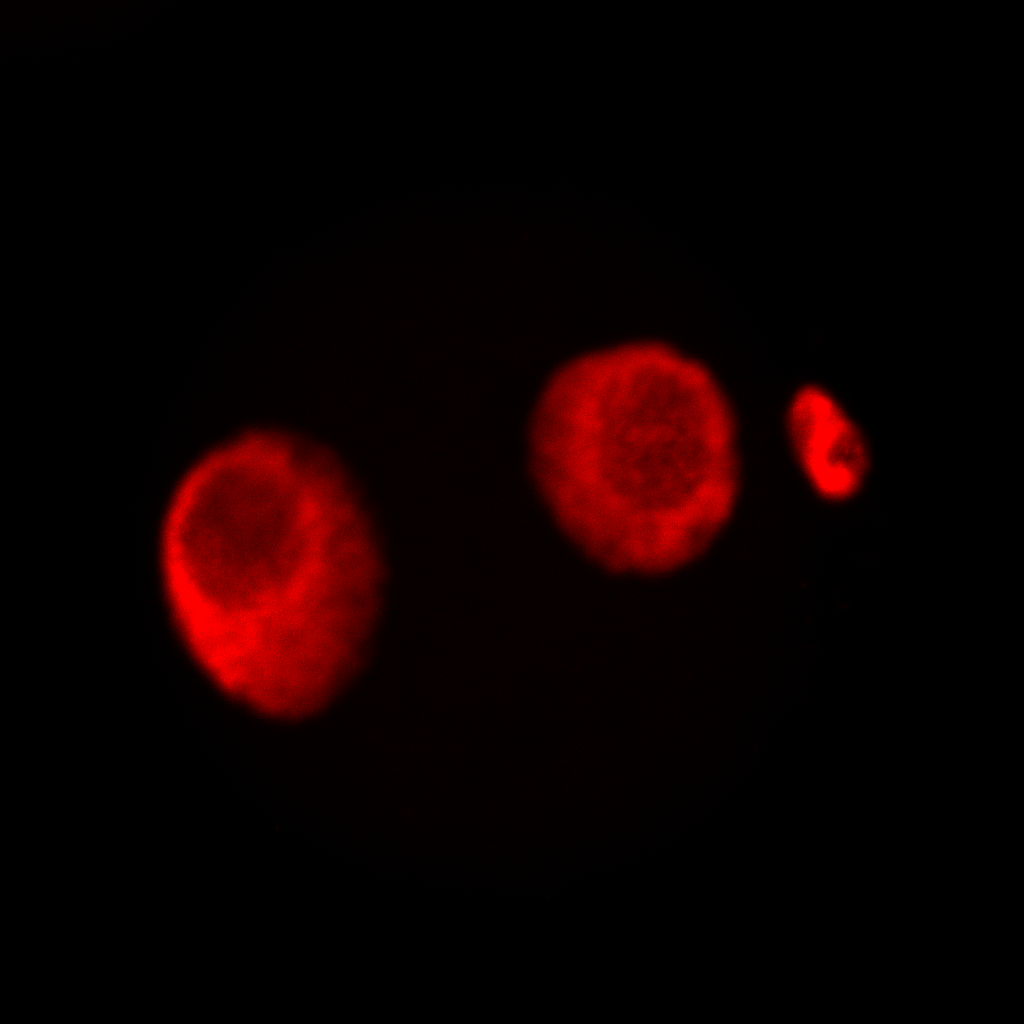

Supplement: Supplementary file 19 — Appendix Source Data [file 44319_2026_712_MOESM19_ESM.zip › Appendix source data/Appendix Figure S2/S2A/Early-S/EdU.tif]

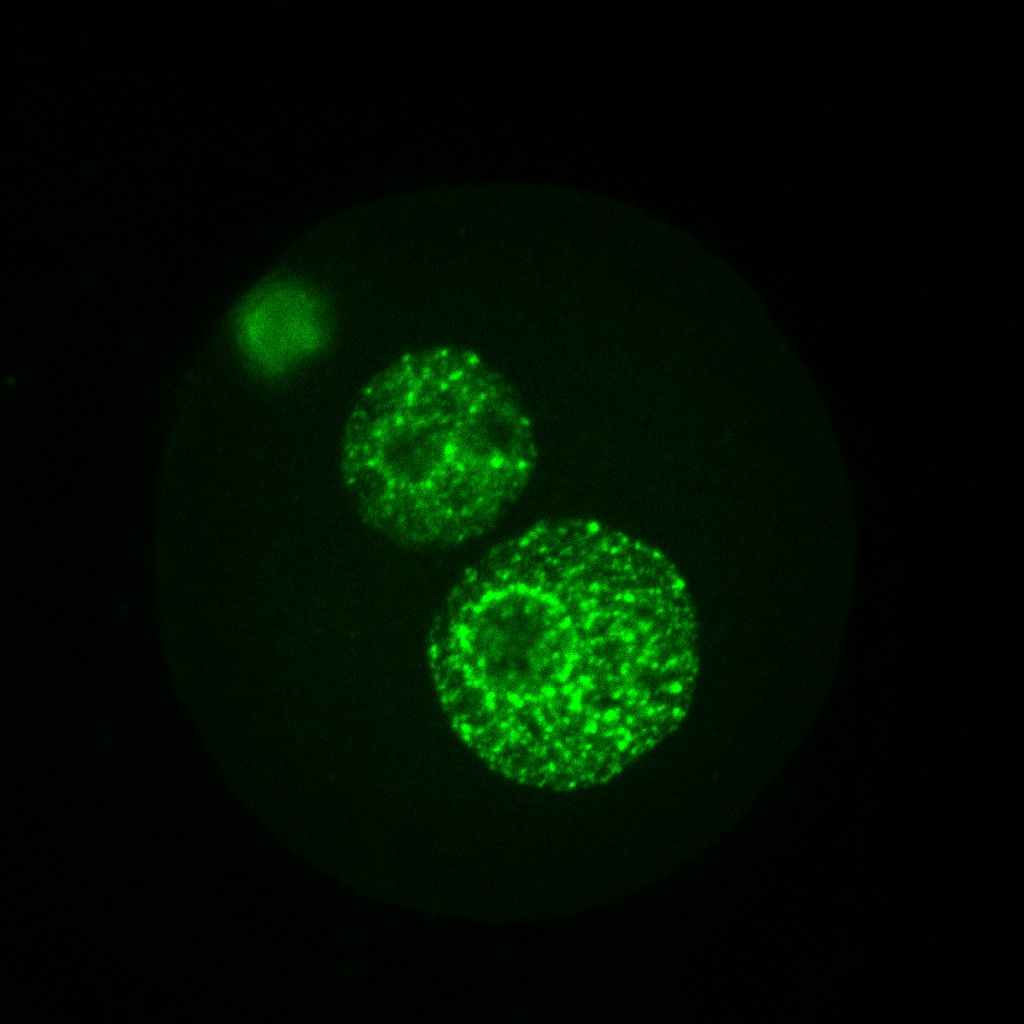

Supplement: Supplementary file 19 — Appendix Source Data [file 44319_2026_712_MOESM19_ESM.zip › Appendix source data/Appendix Figure S2/S2A/Late-S/╬│H2AX.tif]

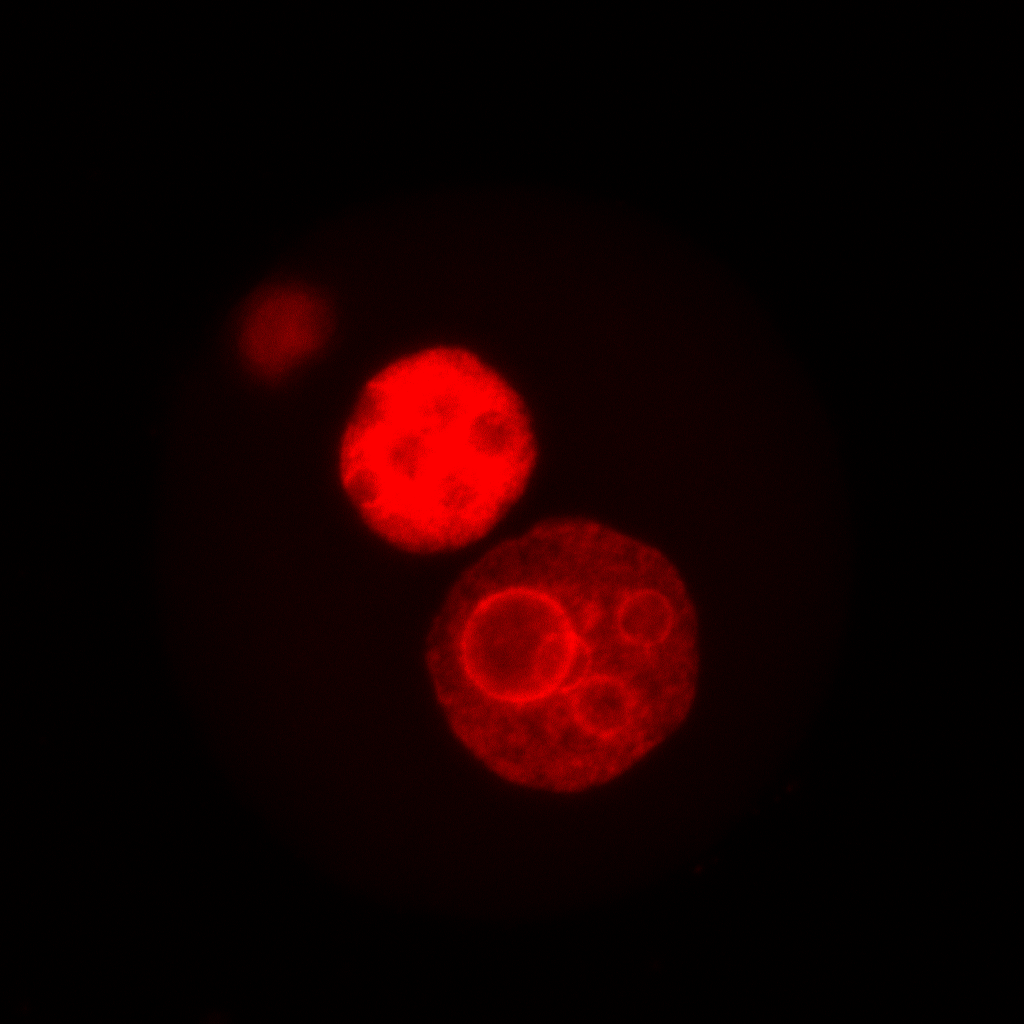

Supplement: Supplementary file 19 — Appendix Source Data [file 44319_2026_712_MOESM19_ESM.zip › Appendix source data/Appendix Figure S2/S2A/Late-S/EdU.tif]

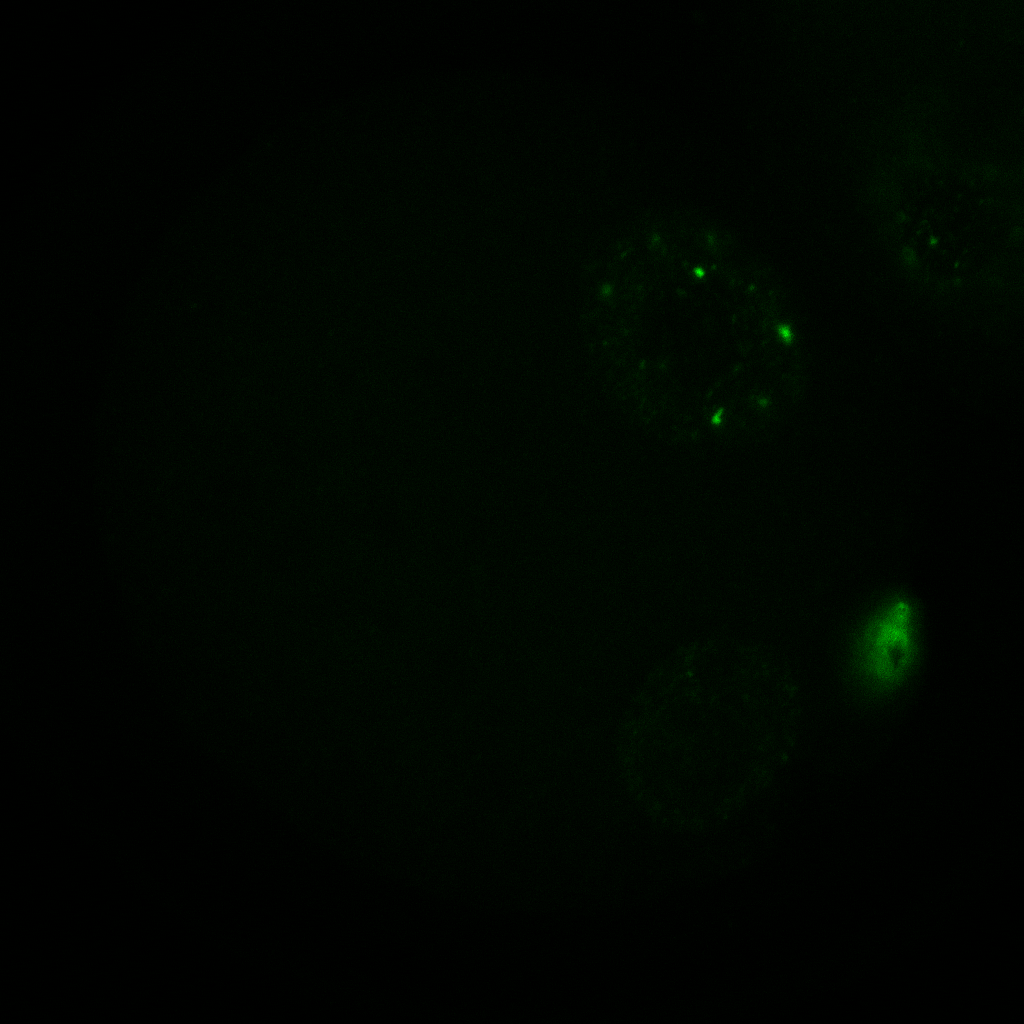

Supplement: Supplementary file 19 — Appendix Source Data [file 44319_2026_712_MOESM19_ESM.zip › Appendix source data/Appendix Figure S2/S2A/Pre-S/╬│H2AX.tif]

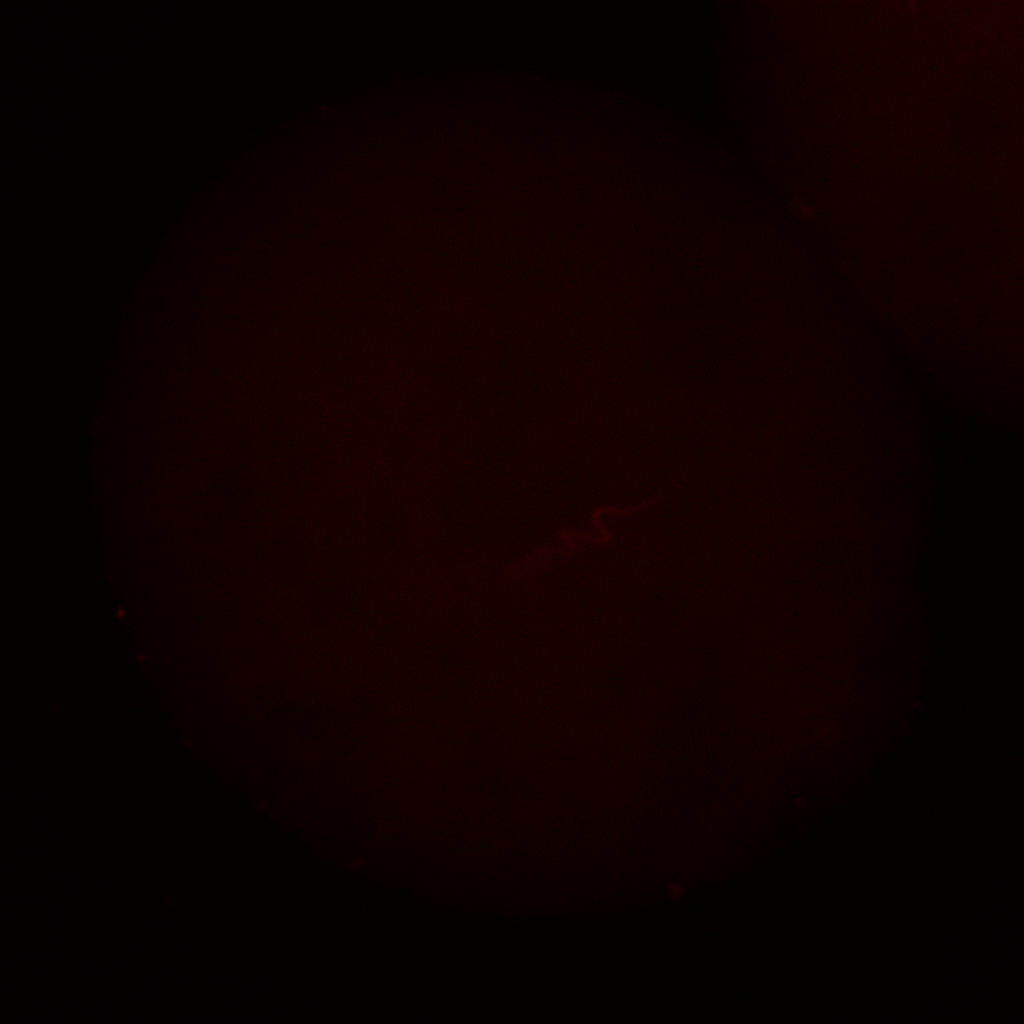

Supplement: Supplementary file 19 — Appendix Source Data [file 44319_2026_712_MOESM19_ESM.zip › Appendix source data/Appendix Figure S2/S2A/Pre-S/EdU.tif]

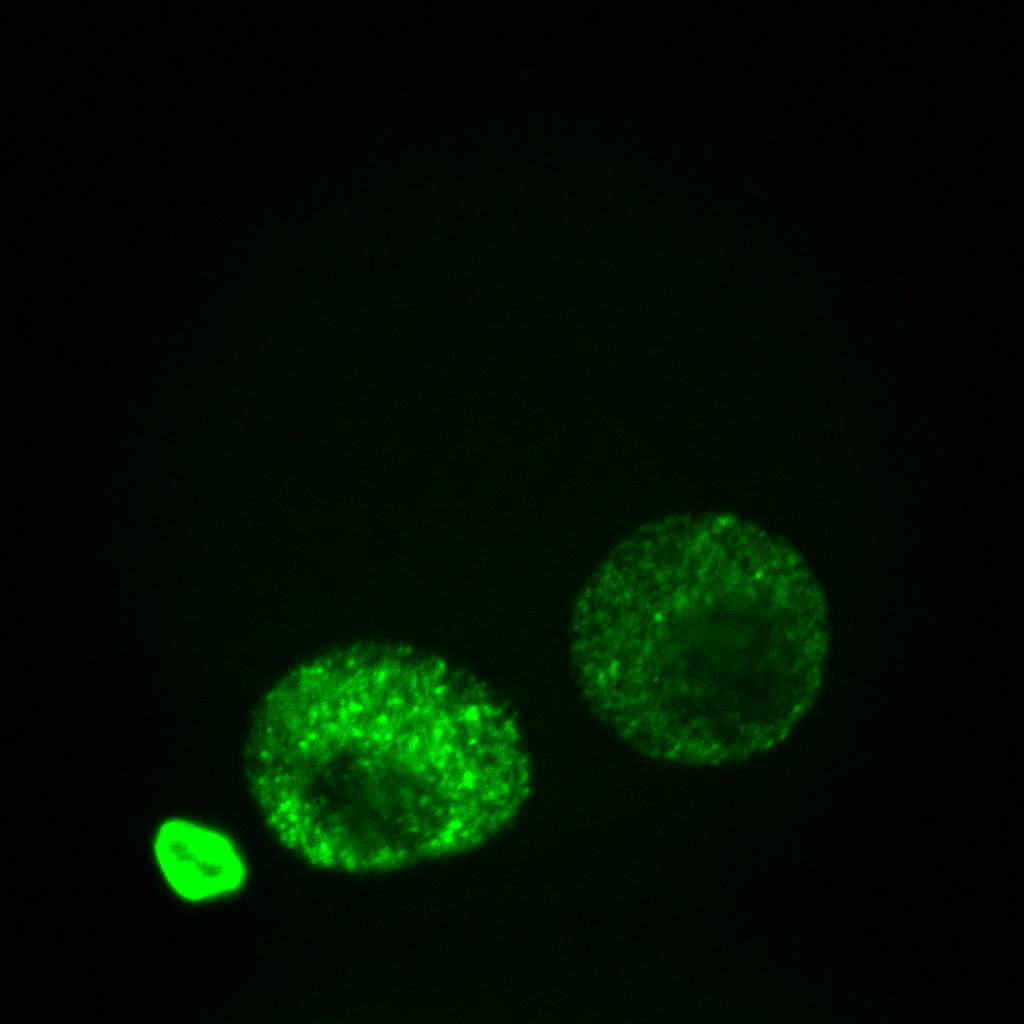

Supplement: Supplementary file 19 — Appendix Source Data [file 44319_2026_712_MOESM19_ESM.zip › Appendix source data/Appendix Figure S2/S2B/Ctrl_hCG21h/Ctrl_hCG21h_╬│H2AX.tif]

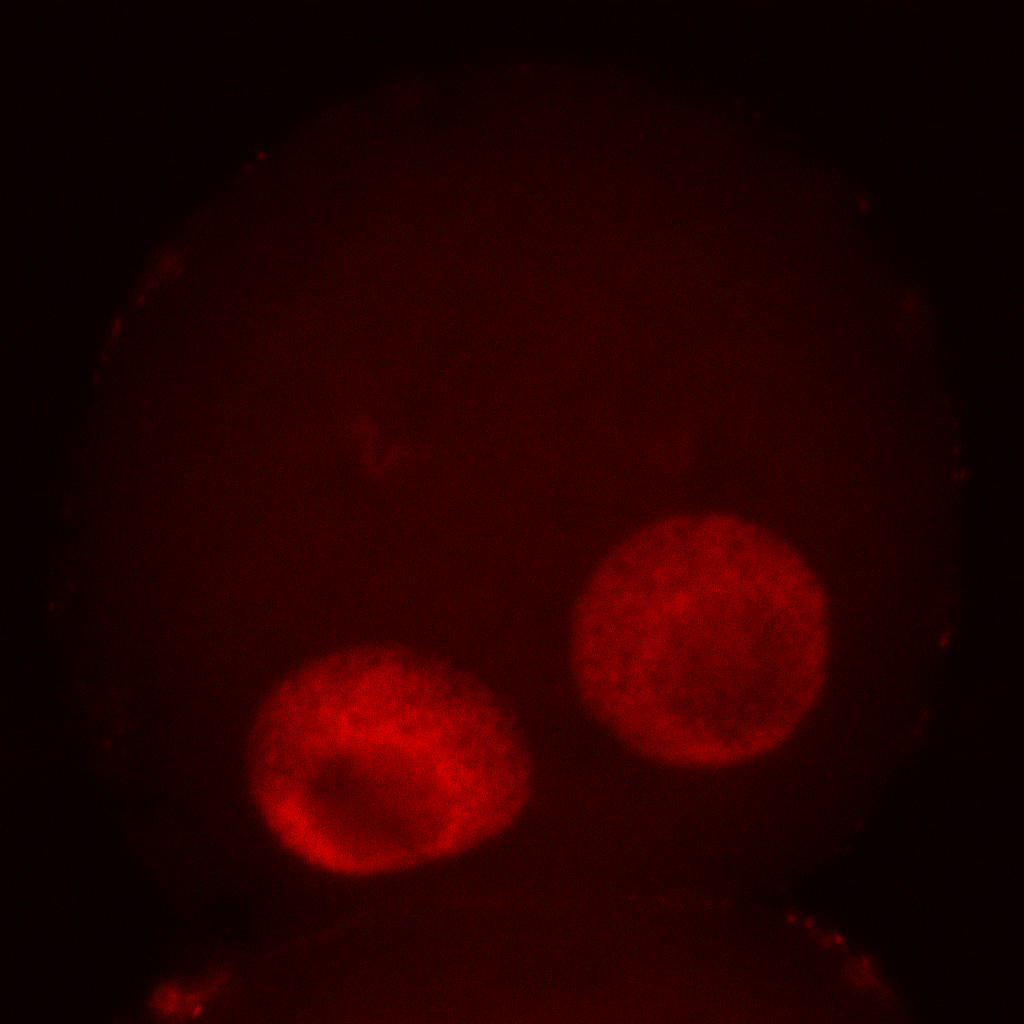

Supplement: Supplementary file 19 — Appendix Source Data [file 44319_2026_712_MOESM19_ESM.zip › Appendix source data/Appendix Figure S2/S2B/Ctrl_hCG21h/Ctrl_hCG21h_EdU.tif]

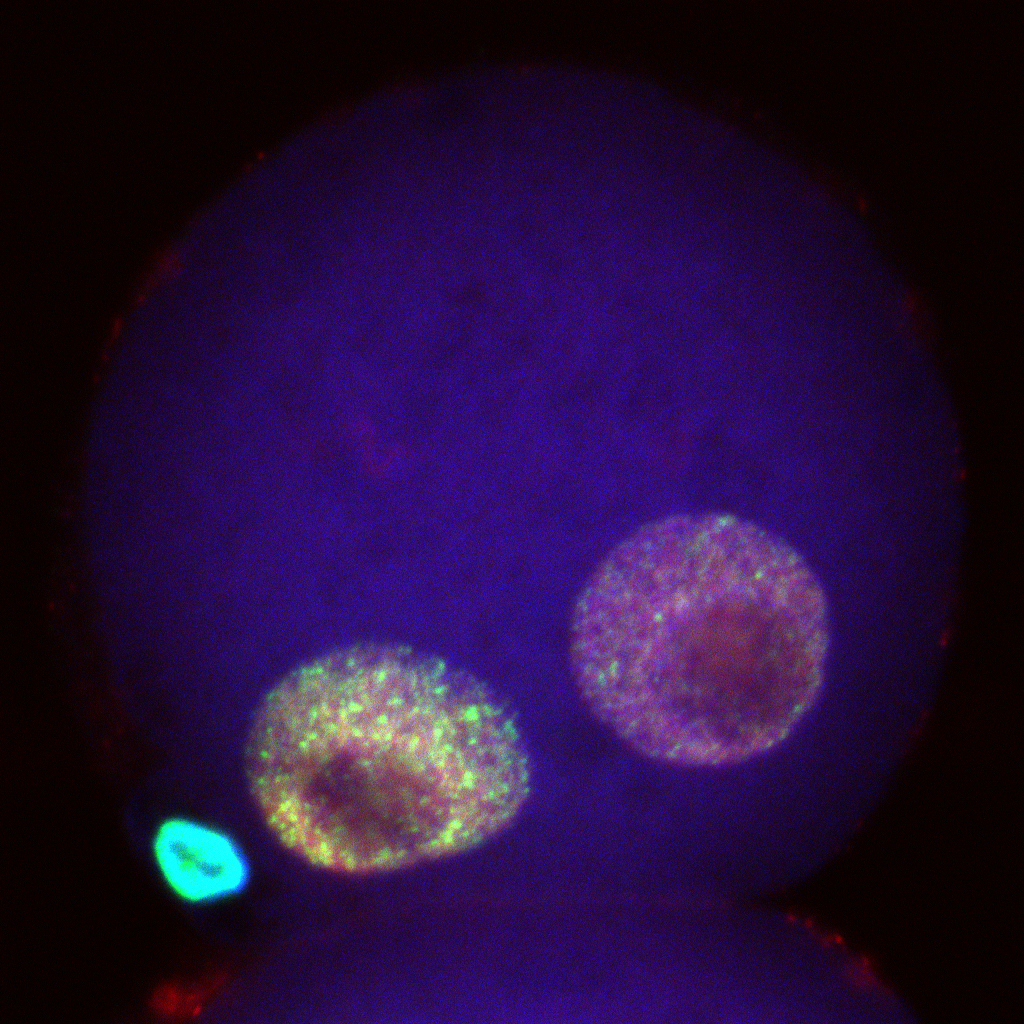

Supplement: Supplementary file 19 — Appendix Source Data [file 44319_2026_712_MOESM19_ESM.zip › Appendix source data/Appendix Figure S2/S2B/Ctrl_hCG21h/Ctrl_hCG21h_Merge.tif]
