# Supplementary figures and images for: THAP1 is a maternal effect factor required for the first cell cycle via Rrm1 in early mouse embryos (part 2 of 2)
Source: EMBO Rep. 2026 Feb 23;27(7):1813–29. doi: 10.1038/s44319-026-00712-9 (PMC13077089; doi:10.1038/s44319-026-00712-9)

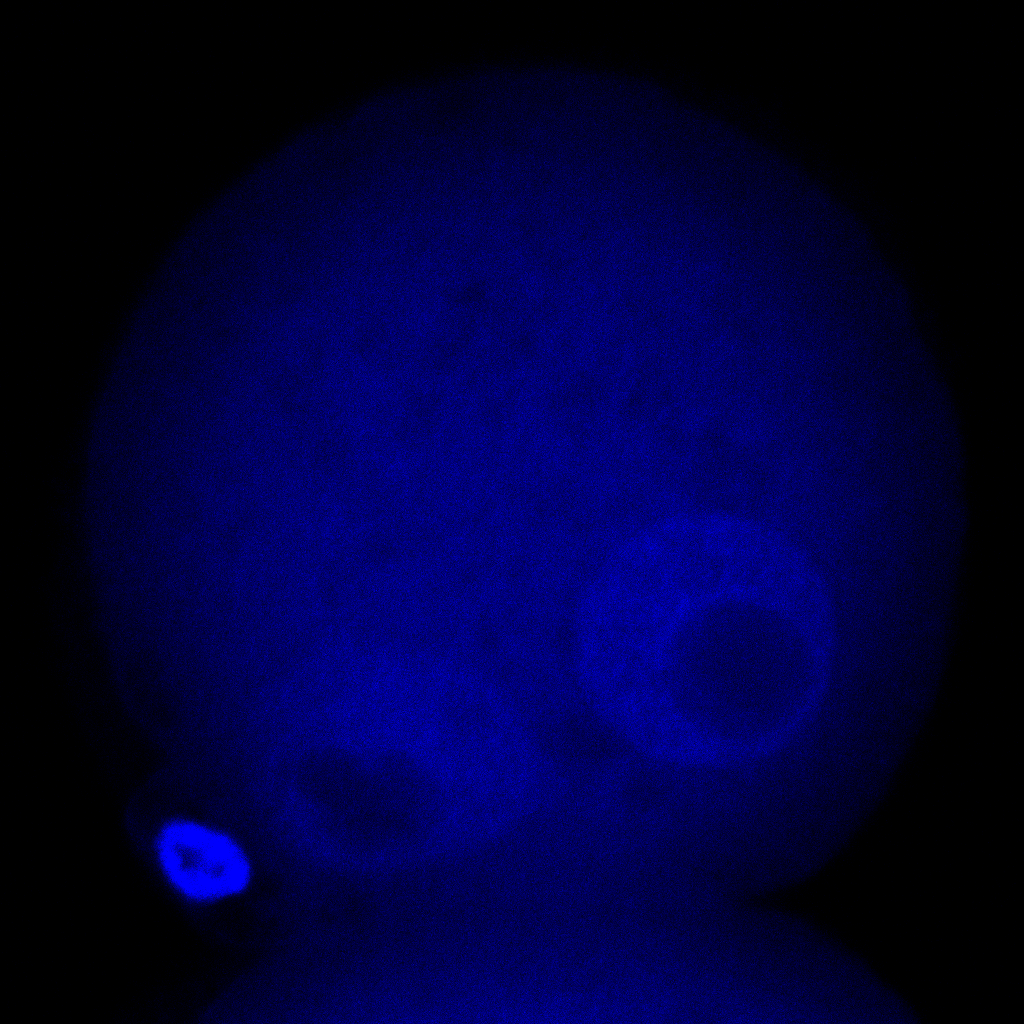

Supplement: Supplementary file 19 — Appendix Source Data [file 44319_2026_712_MOESM19_ESM.zip › Appendix source data/Appendix Figure S2/S2B/Ctrl_hCG21h/Ctrl_hCG21h_DAPI.tif]

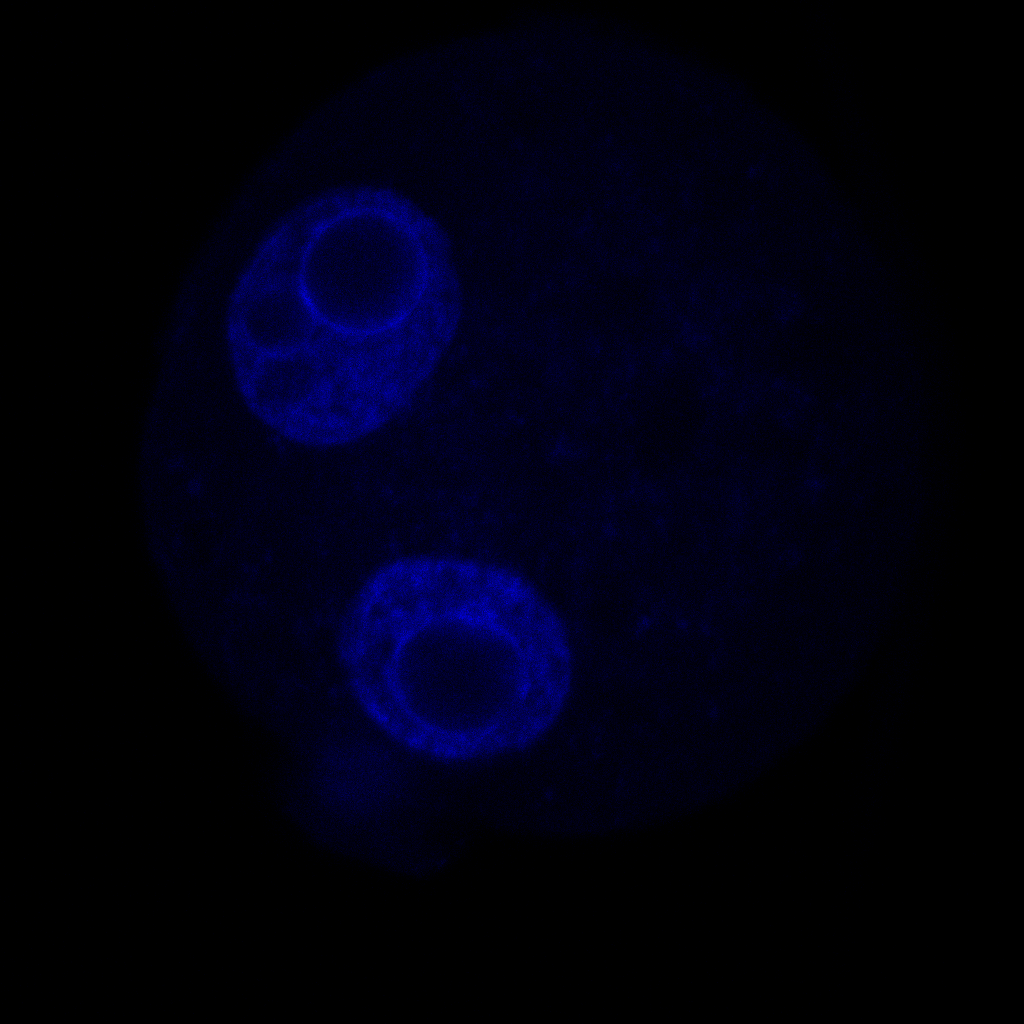

Supplement: Supplementary file 19 — Appendix Source Data [file 44319_2026_712_MOESM19_ESM.zip › Appendix source data/Appendix Figure S2/S2B/mKO_hCG21h/mKO_hCG21h_DAPI.tif]

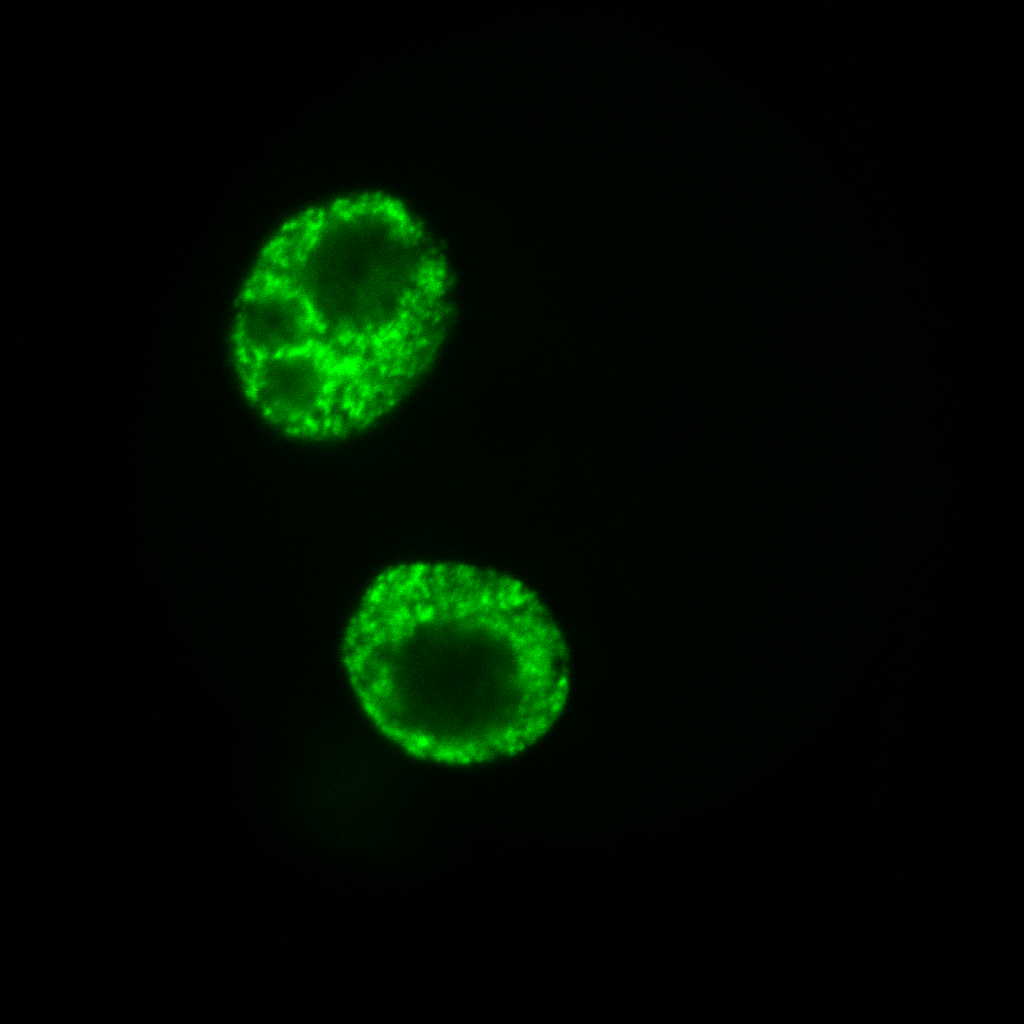

Supplement: Supplementary file 19 — Appendix Source Data [file 44319_2026_712_MOESM19_ESM.zip › Appendix source data/Appendix Figure S2/S2B/mKO_hCG21h/mKO_hCG21h_╬│H2AX.tif]

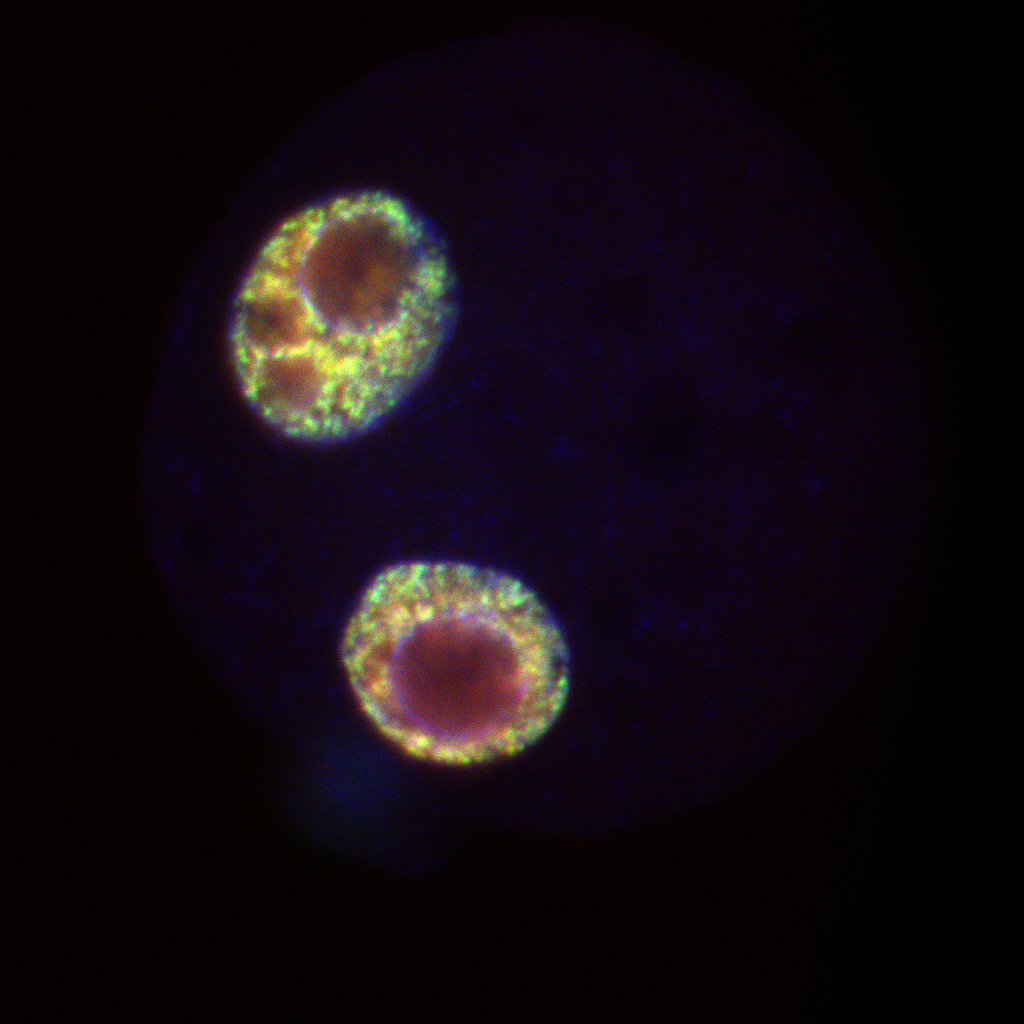

Supplement: Supplementary file 19 — Appendix Source Data [file 44319_2026_712_MOESM19_ESM.zip › Appendix source data/Appendix Figure S2/S2B/mKO_hCG21h/mKO_hCG21h_Merge.tif]

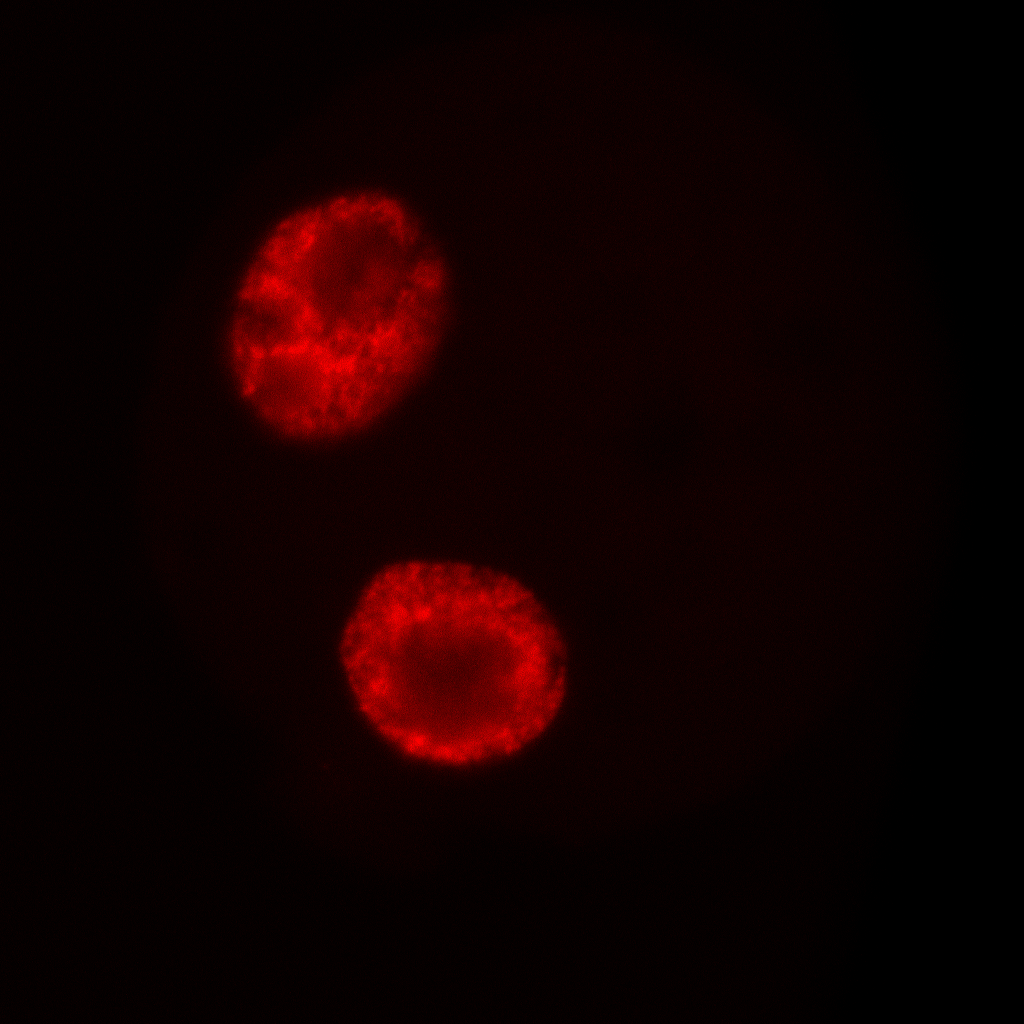

Supplement: Supplementary file 19 — Appendix Source Data [file 44319_2026_712_MOESM19_ESM.zip › Appendix source data/Appendix Figure S2/S2B/mKO_hCG21h/mKO_hCG21h_EdU.tif]

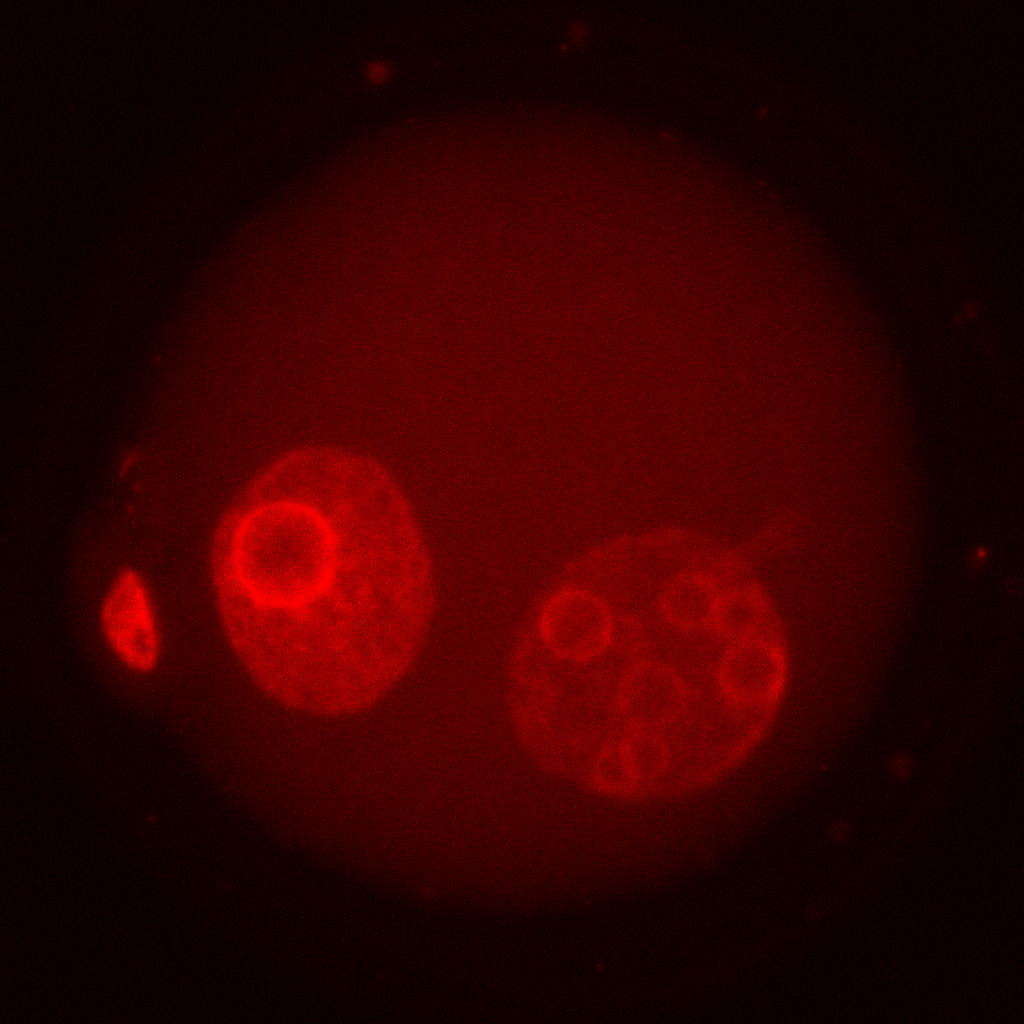

Supplement: Supplementary file 19 — Appendix Source Data [file 44319_2026_712_MOESM19_ESM.zip › Appendix source data/Appendix Figure S2/S2C/Ctrl_Late-S_hCG24h/Ctrl_Late-S_hCG24h_EdU.tif]

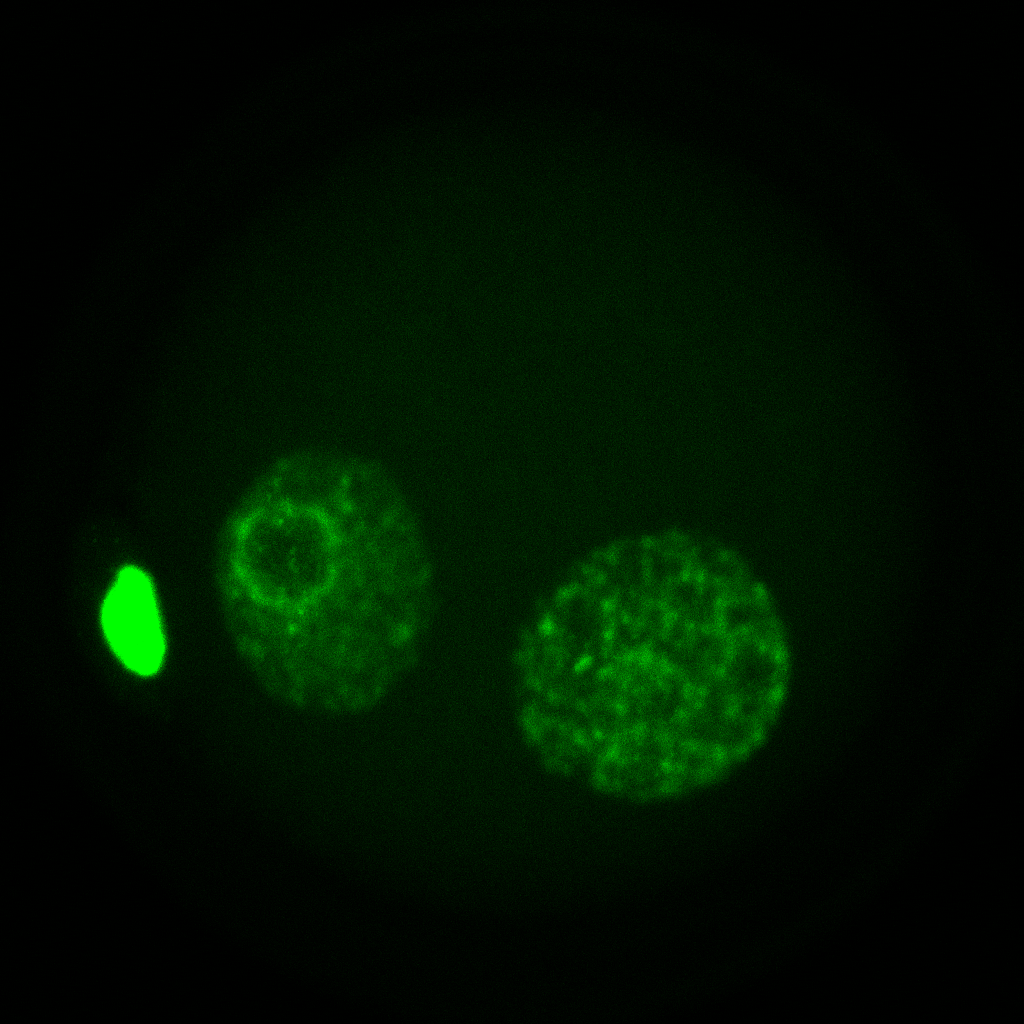

Supplement: Supplementary file 19 — Appendix Source Data [file 44319_2026_712_MOESM19_ESM.zip › Appendix source data/Appendix Figure S2/S2C/Ctrl_Late-S_hCG24h/Ctrl_Late-S_hCG24h_╬│H2AX.tif]

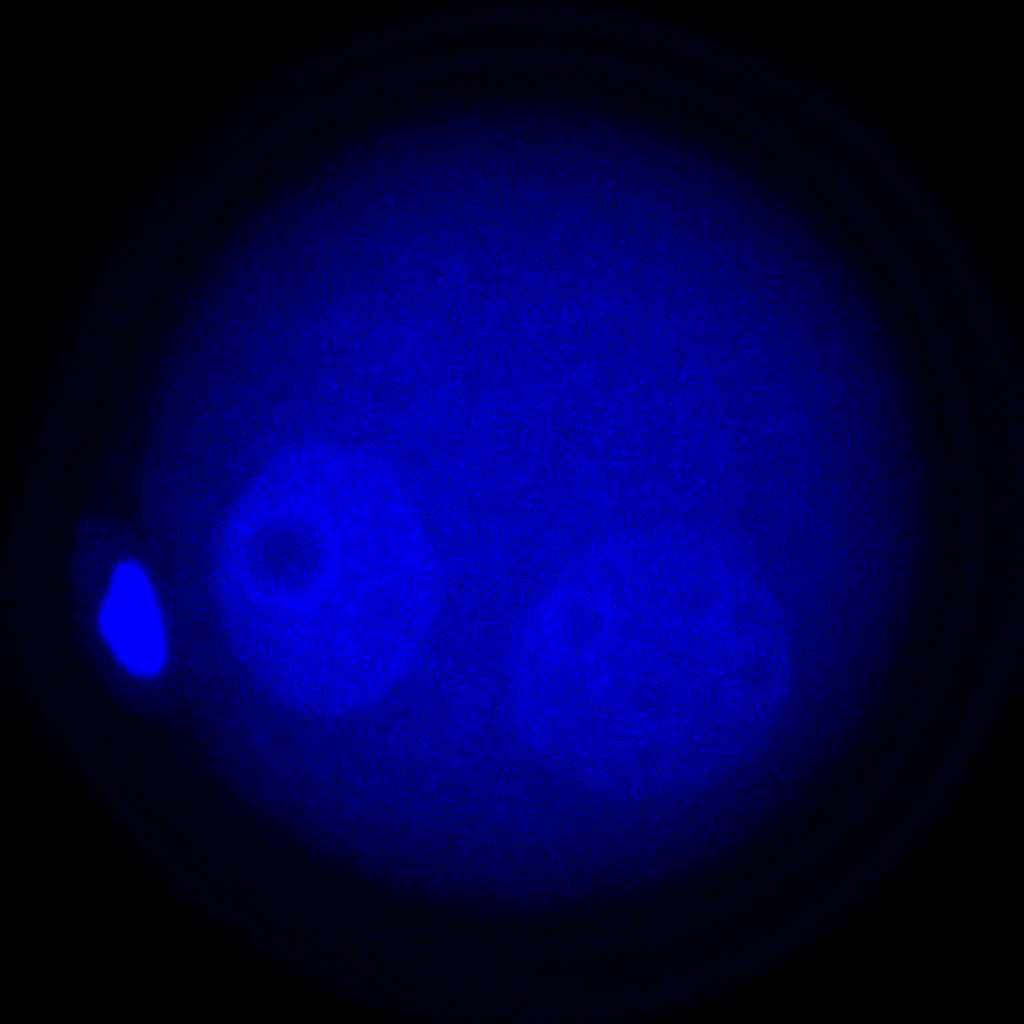

Supplement: Supplementary file 19 — Appendix Source Data [file 44319_2026_712_MOESM19_ESM.zip › Appendix source data/Appendix Figure S2/S2C/Ctrl_Late-S_hCG24h/Ctrl_Late-S_hCG24h_DAPI.tif]

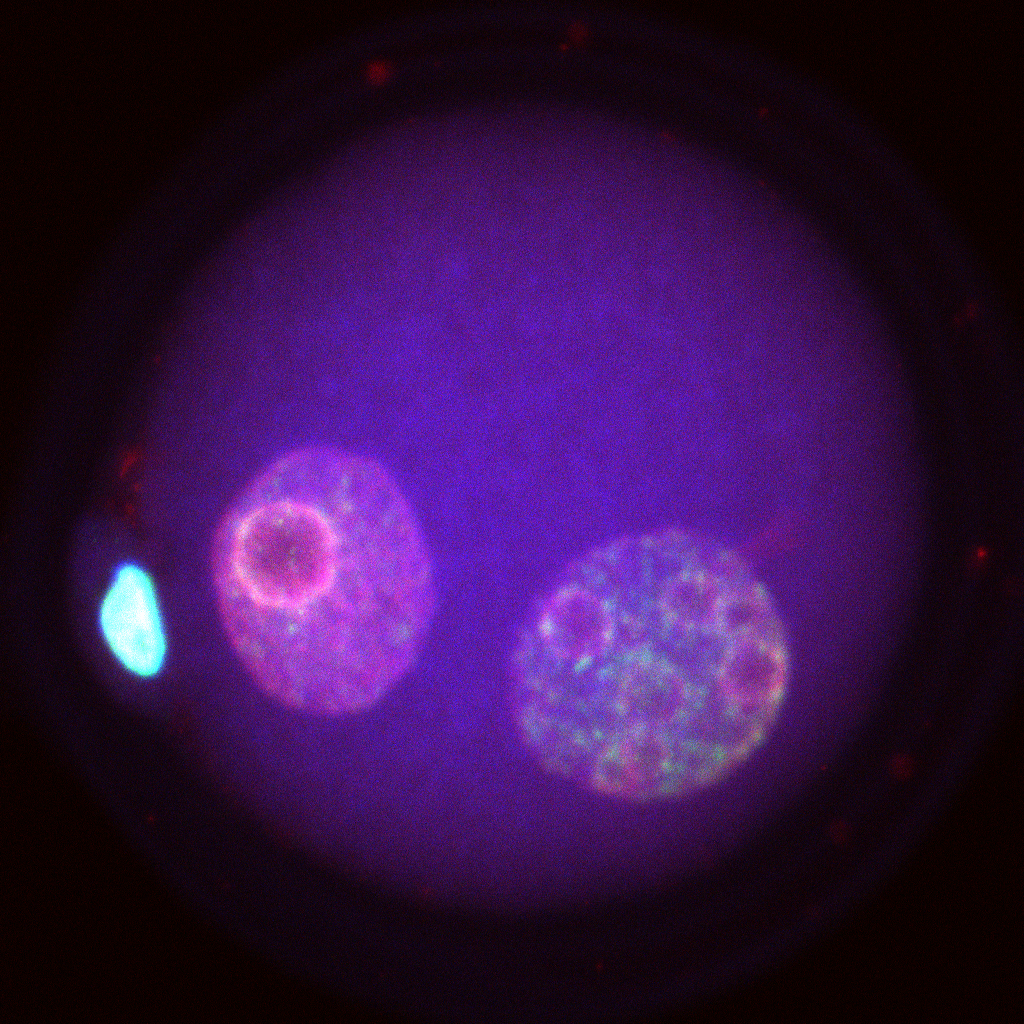

Supplement: Supplementary file 19 — Appendix Source Data [file 44319_2026_712_MOESM19_ESM.zip › Appendix source data/Appendix Figure S2/S2C/Ctrl_Late-S_hCG24h/Ctrl_Late-S_hCG24h_Merge.tif]

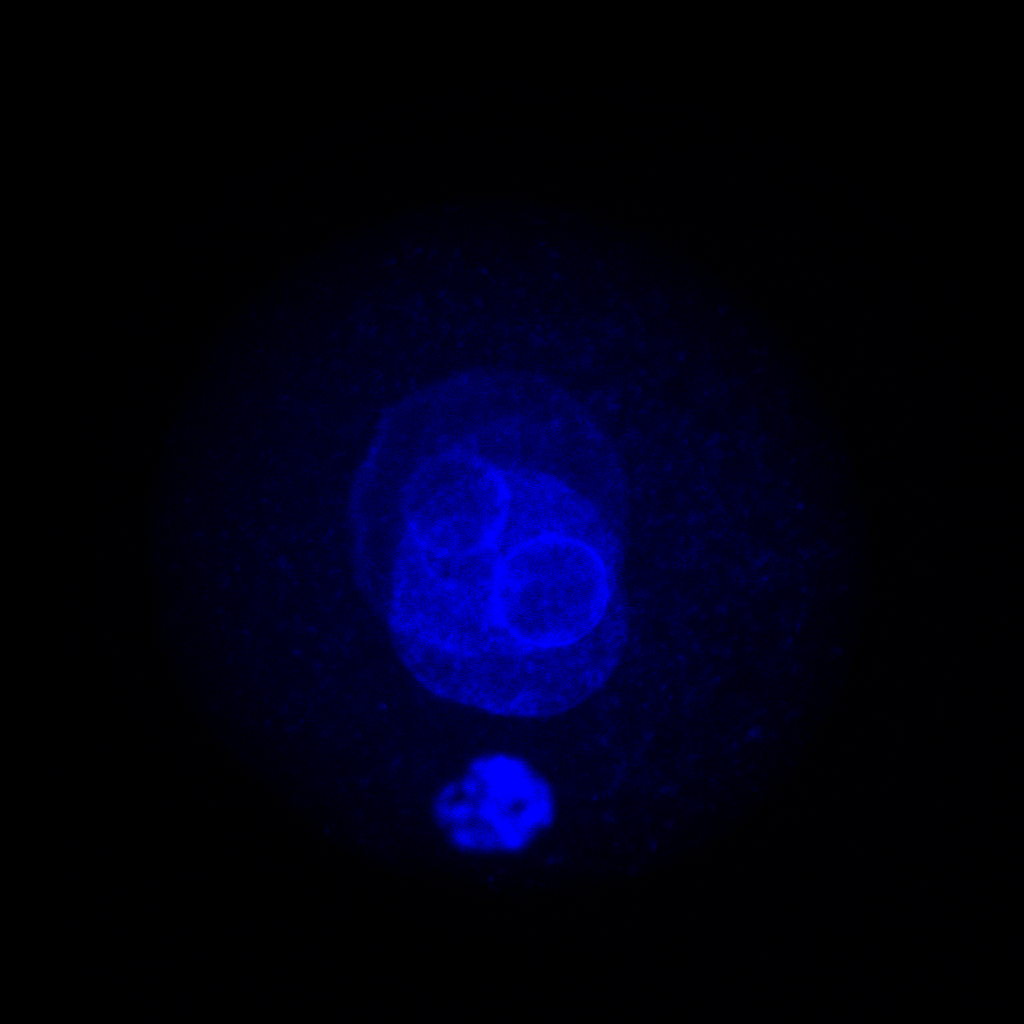

Supplement: Supplementary file 19 — Appendix Source Data [file 44319_2026_712_MOESM19_ESM.zip › Appendix source data/Appendix Figure S2/S2C/mKO_Late-S_hCG27h/mKO_hCG27h_DAPI.tif]

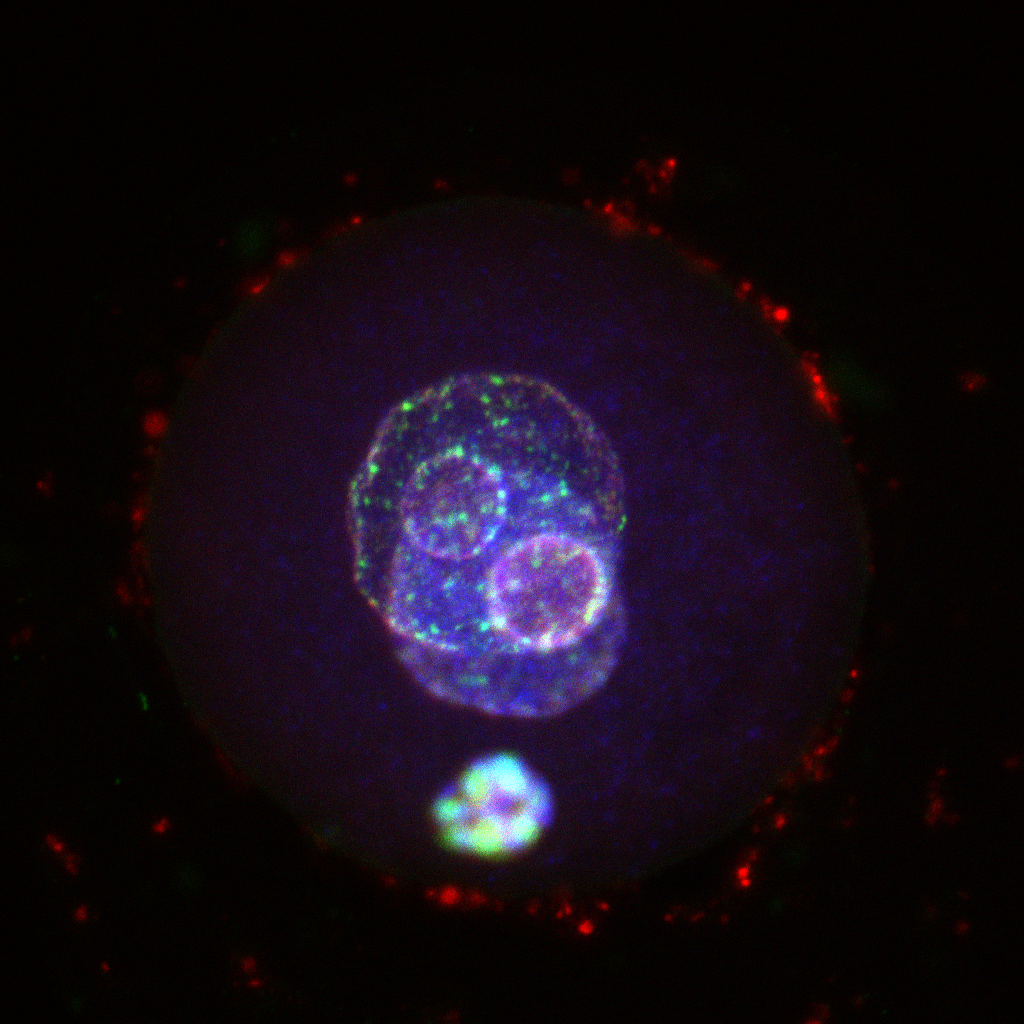

Supplement: Supplementary file 19 — Appendix Source Data [file 44319_2026_712_MOESM19_ESM.zip › Appendix source data/Appendix Figure S2/S2C/mKO_Late-S_hCG27h/mKO_hCG27h_Merge.tif]

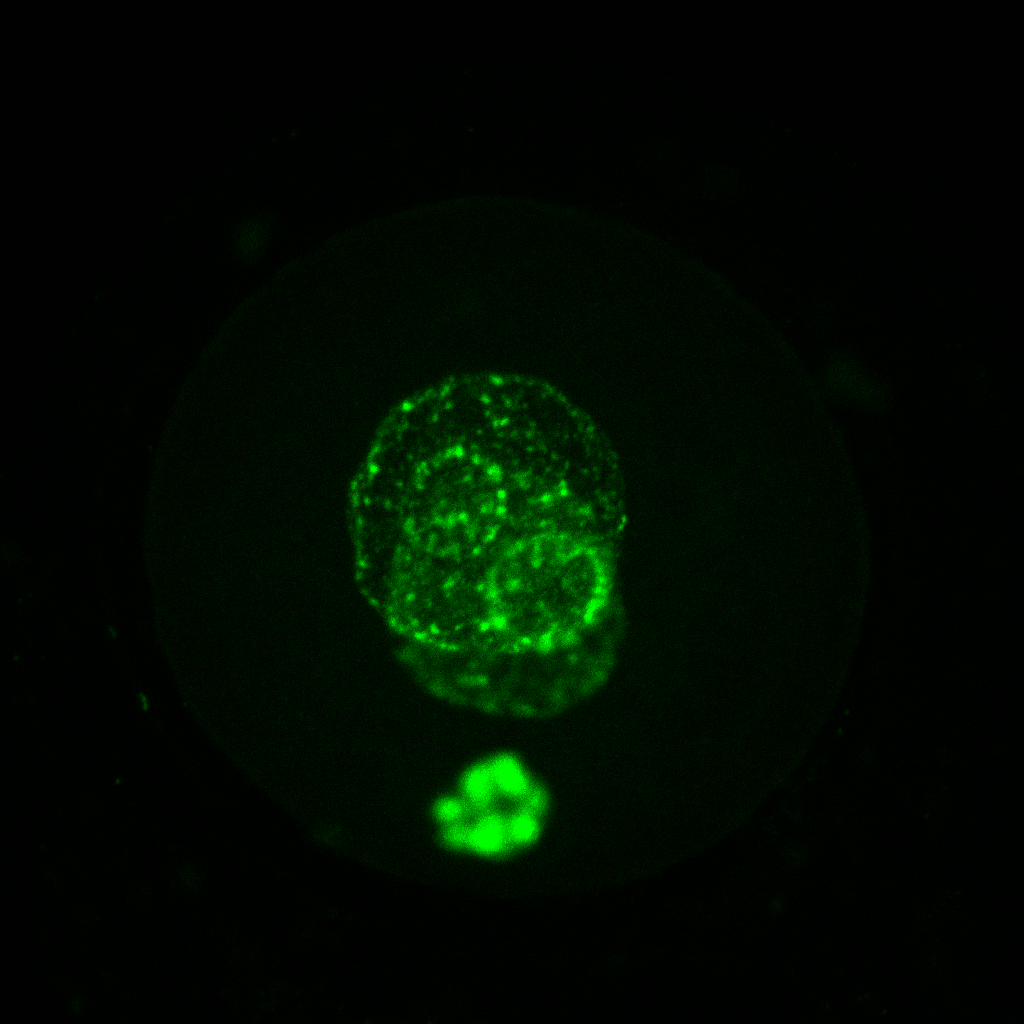

Supplement: Supplementary file 19 — Appendix Source Data [file 44319_2026_712_MOESM19_ESM.zip › Appendix source data/Appendix Figure S2/S2C/mKO_Late-S_hCG27h/mKO_hCG27h_╬│H2AX.tif]

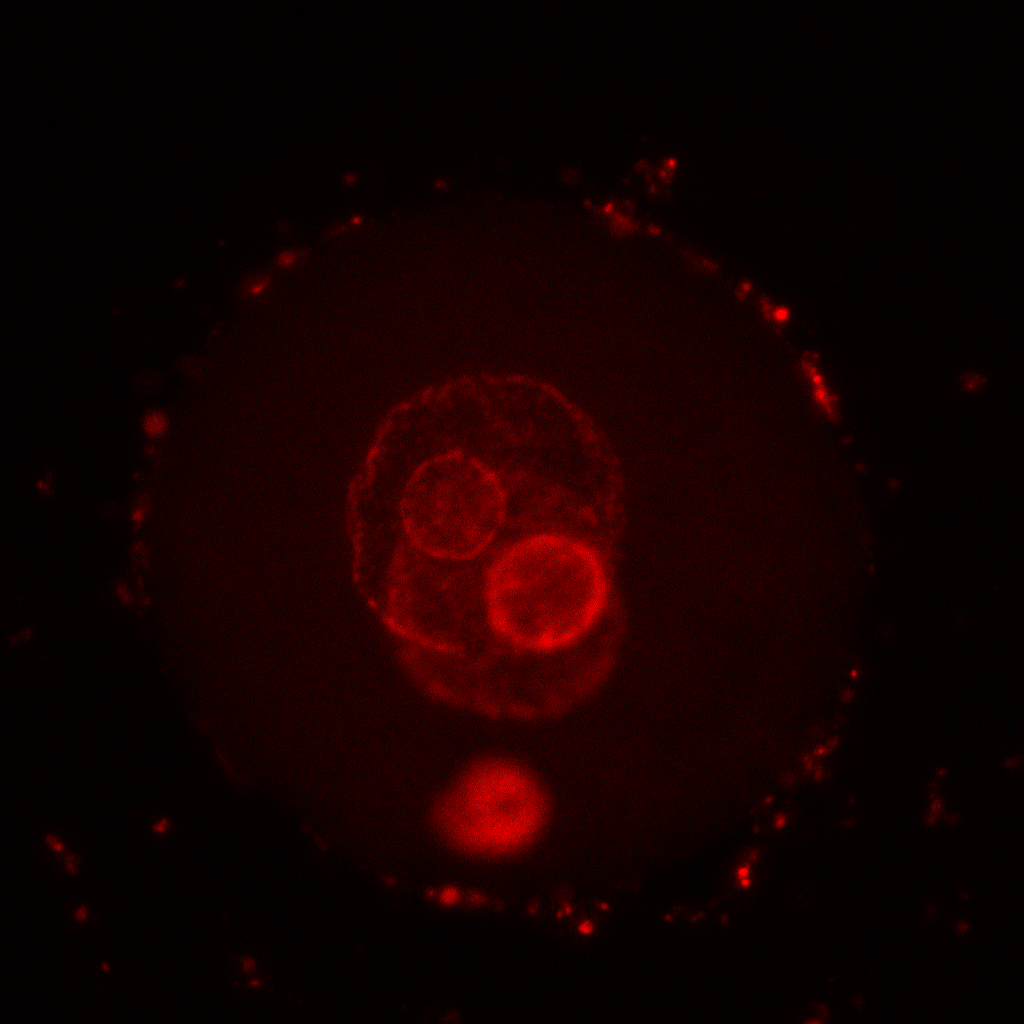

Supplement: Supplementary file 19 — Appendix Source Data [file 44319_2026_712_MOESM19_ESM.zip › Appendix source data/Appendix Figure S2/S2C/mKO_Late-S_hCG27h/mKO_hCG27h_EdU.tif]

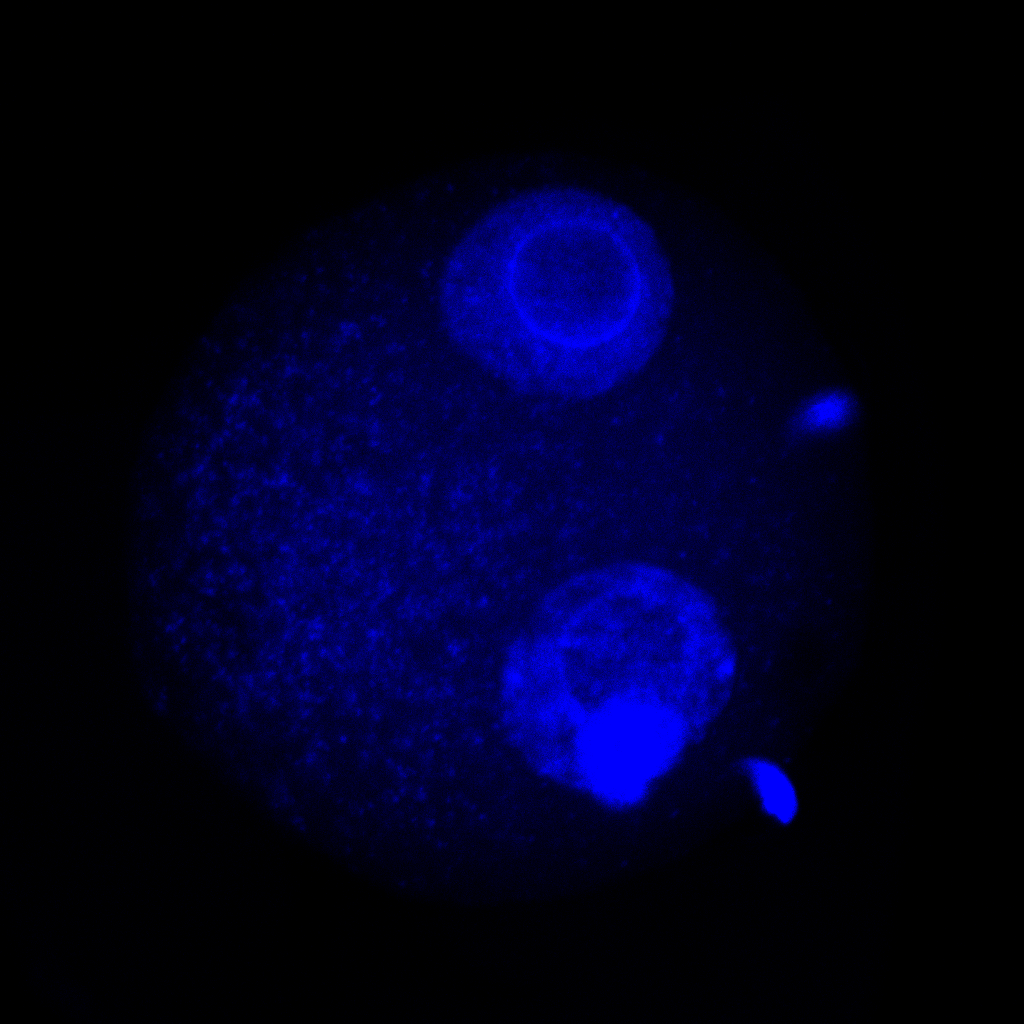

Supplement: Supplementary file 19 — Appendix Source Data [file 44319_2026_712_MOESM19_ESM.zip › Appendix source data/Appendix Figure S2/S2C/mKO_Early-S_hCG27h/mKO_hCG27h_DAPI.tif]

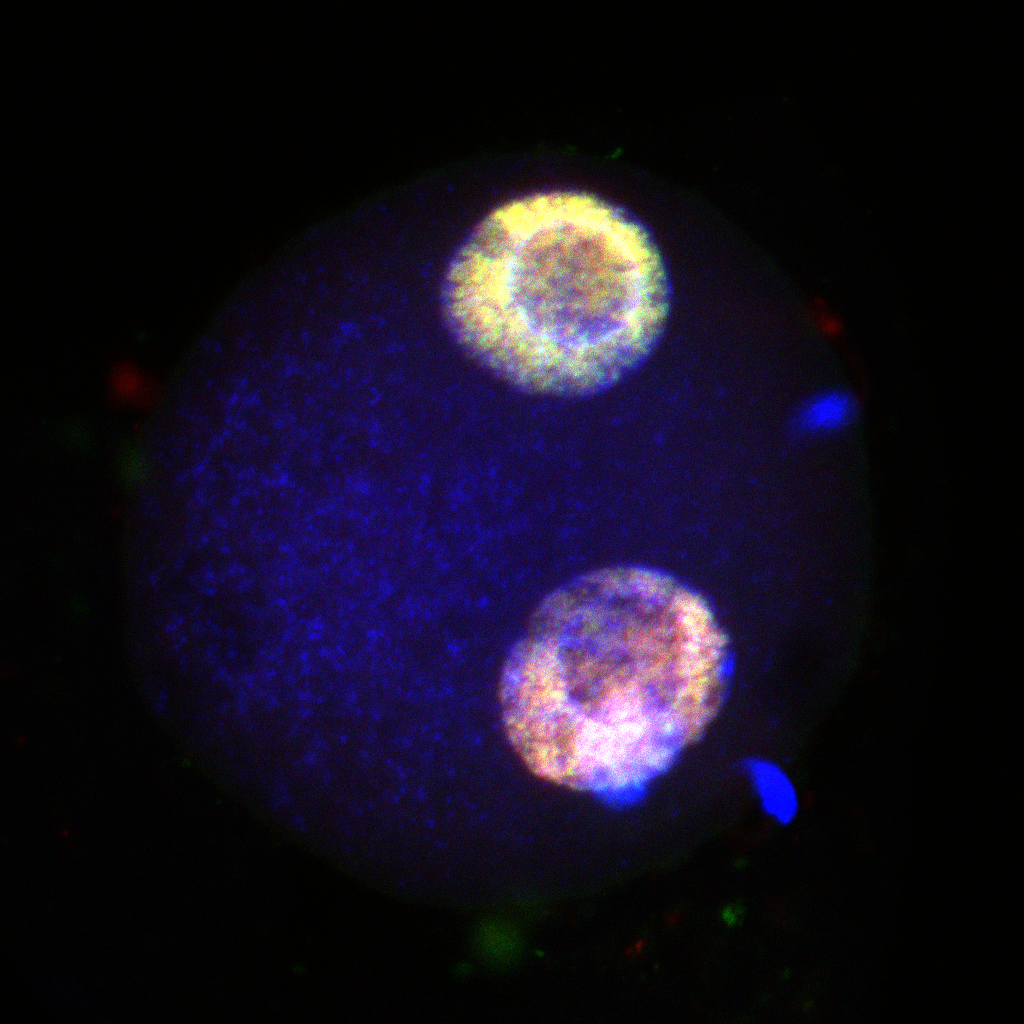

Supplement: Supplementary file 19 — Appendix Source Data [file 44319_2026_712_MOESM19_ESM.zip › Appendix source data/Appendix Figure S2/S2C/mKO_Early-S_hCG27h/mKO_hCG27h_Merge.tif]

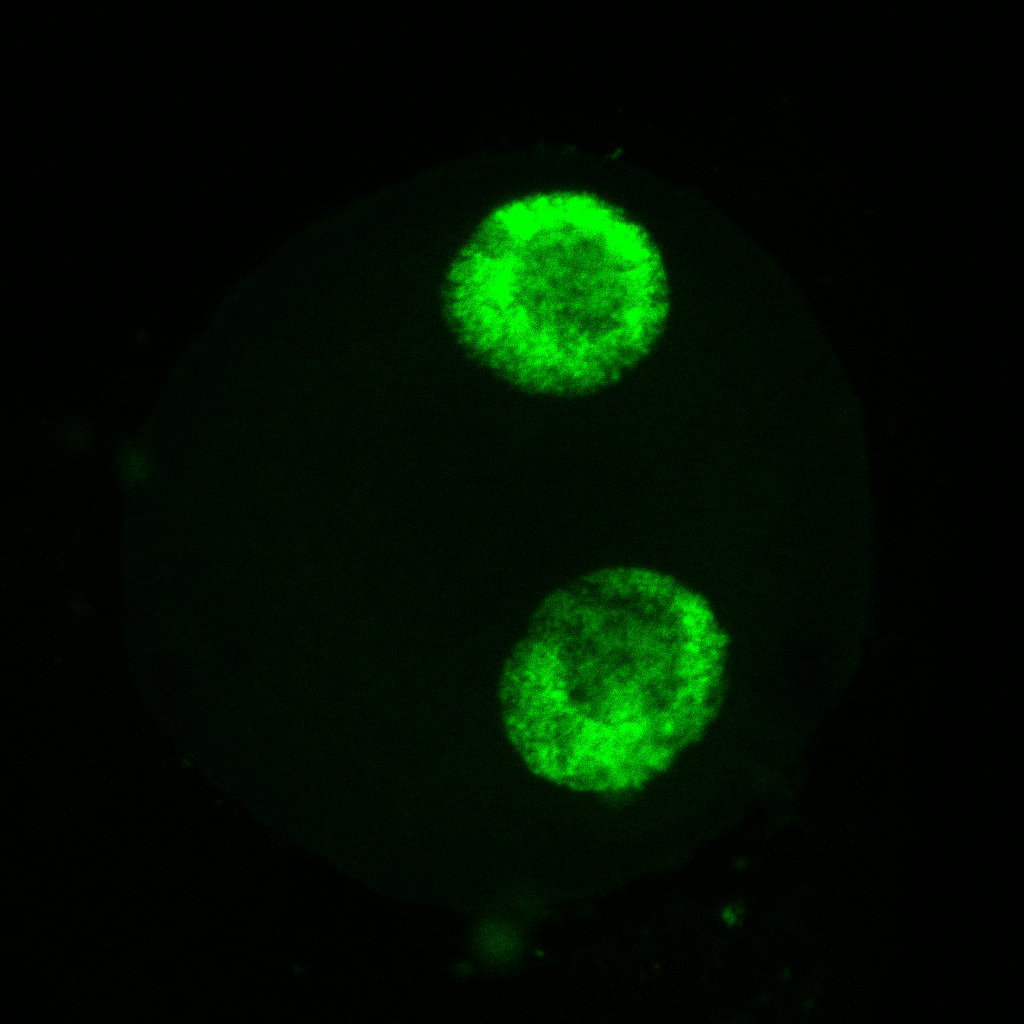

Supplement: Supplementary file 19 — Appendix Source Data [file 44319_2026_712_MOESM19_ESM.zip › Appendix source data/Appendix Figure S2/S2C/mKO_Early-S_hCG27h/mKO_hCG27h_╬│H2AX.tif]

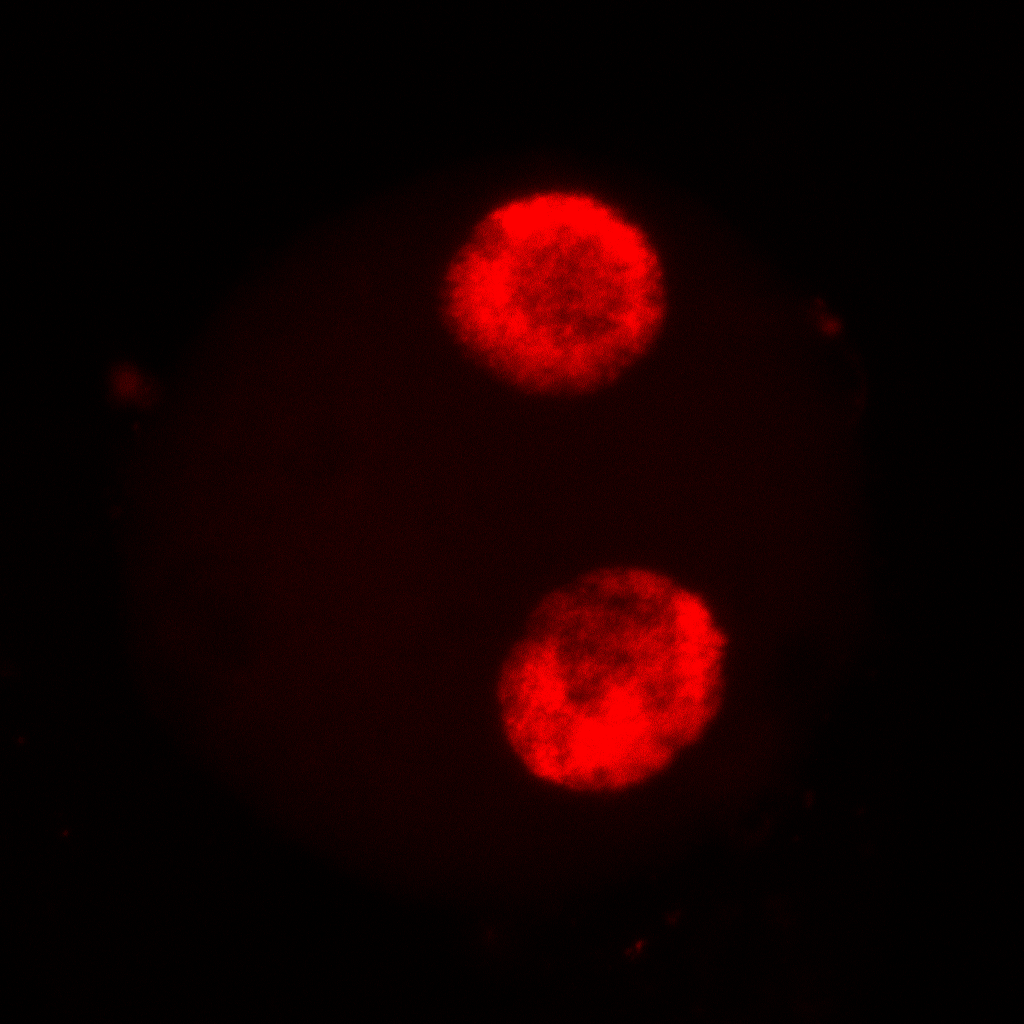

Supplement: Supplementary file 19 — Appendix Source Data [file 44319_2026_712_MOESM19_ESM.zip › Appendix source data/Appendix Figure S2/S2C/mKO_Early-S_hCG27h/mKO_hCG27h_EdU.tif]

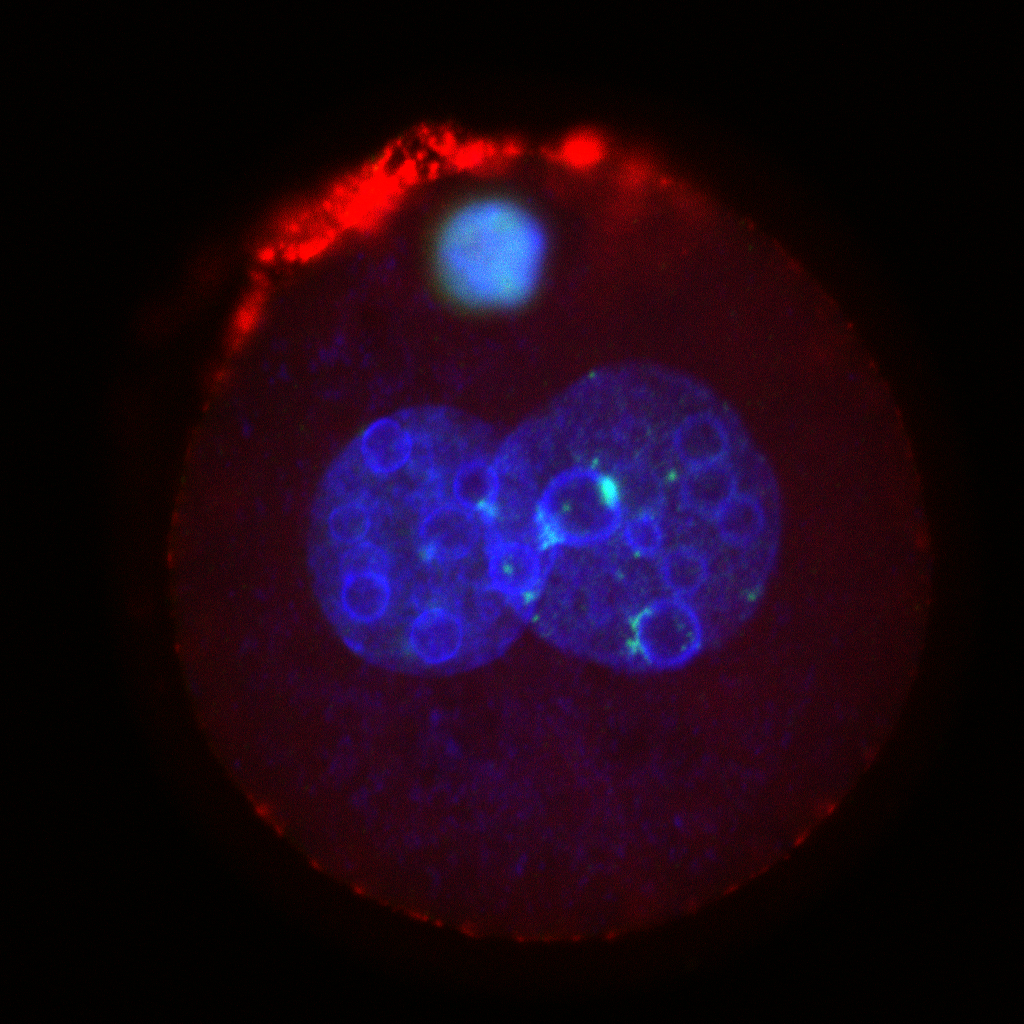

Supplement: Supplementary file 19 — Appendix Source Data [file 44319_2026_712_MOESM19_ESM.zip › Appendix source data/Appendix Figure S2/S2D/Ctrl_Post-S_hCG27h/Ctrl_hCG27h_Merge.tif]

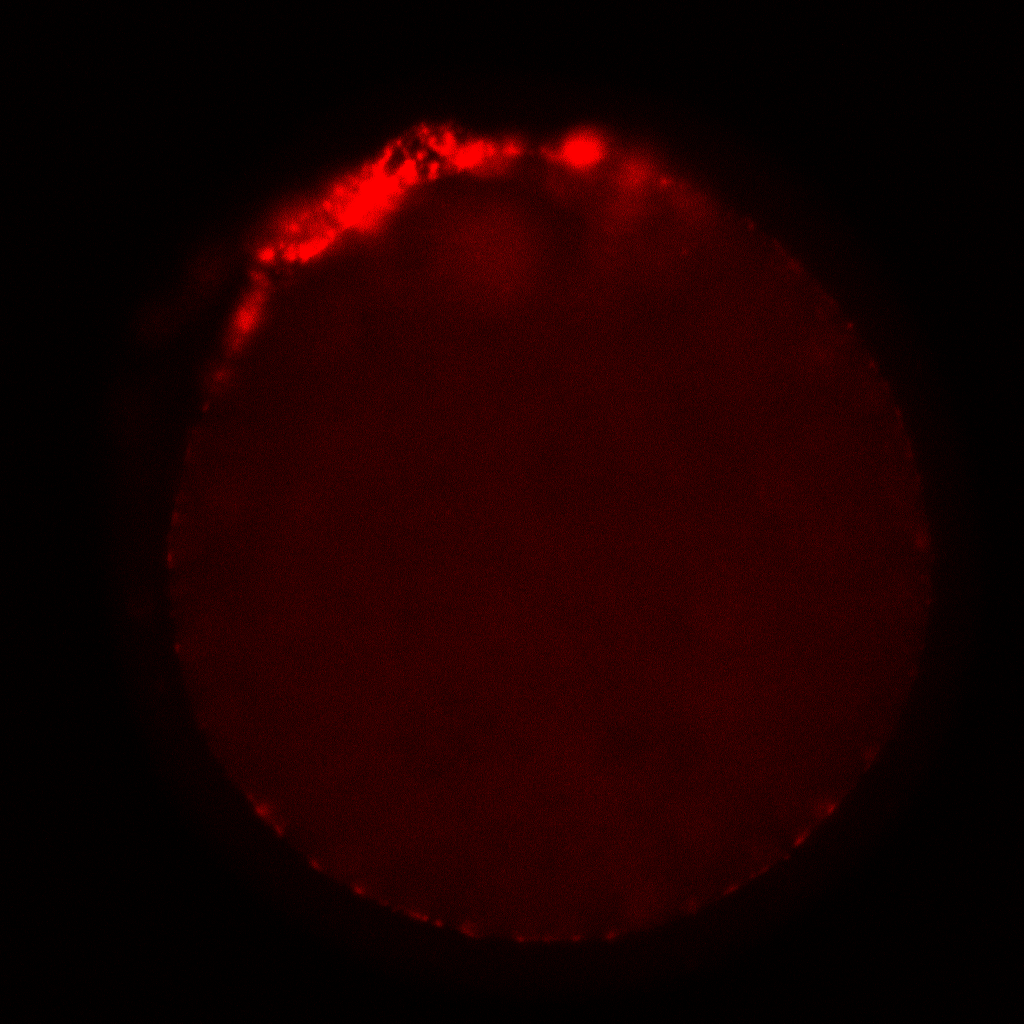

Supplement: Supplementary file 19 — Appendix Source Data [file 44319_2026_712_MOESM19_ESM.zip › Appendix source data/Appendix Figure S2/S2D/Ctrl_Post-S_hCG27h/Ctrl_hCG27h_EdU.tif]

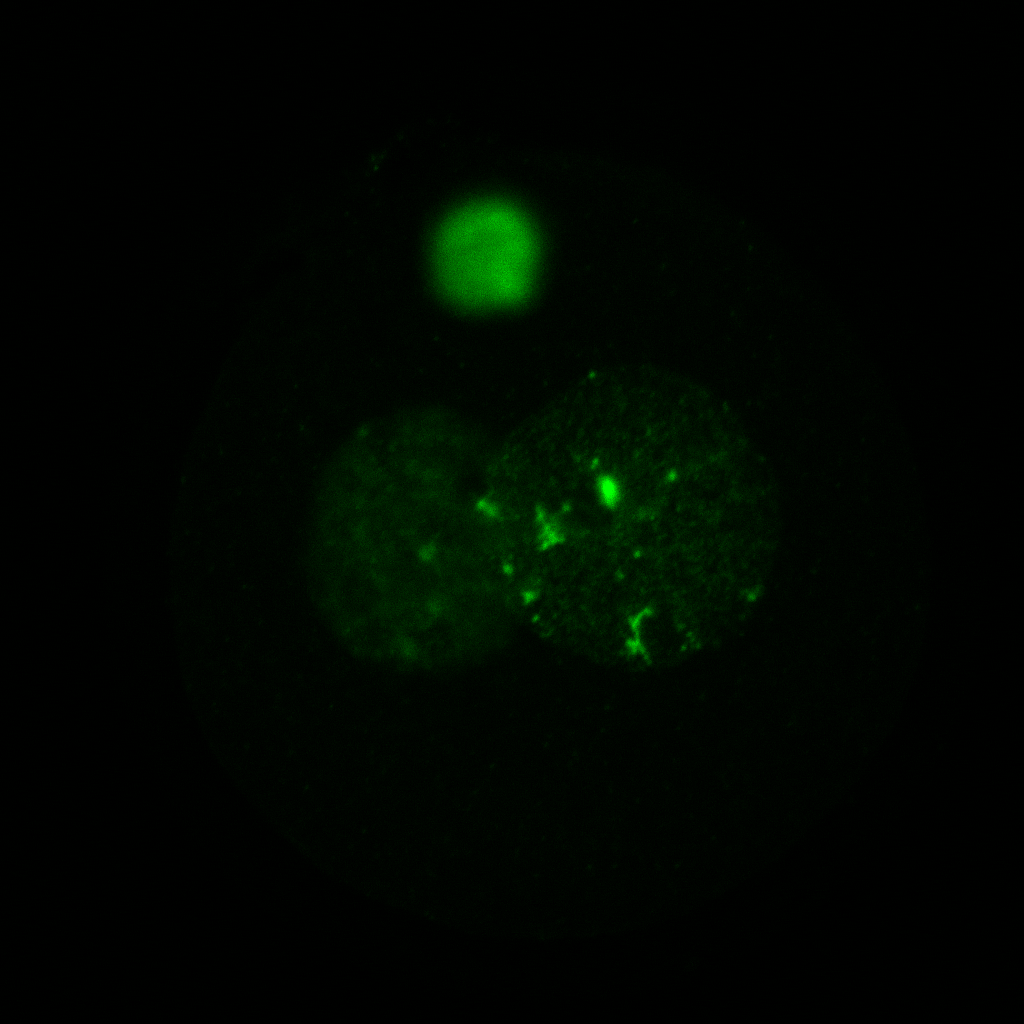

Supplement: Supplementary file 19 — Appendix Source Data [file 44319_2026_712_MOESM19_ESM.zip › Appendix source data/Appendix Figure S2/S2D/Ctrl_Post-S_hCG27h/Ctrl_hCG27h_╬│H2AX.tif]

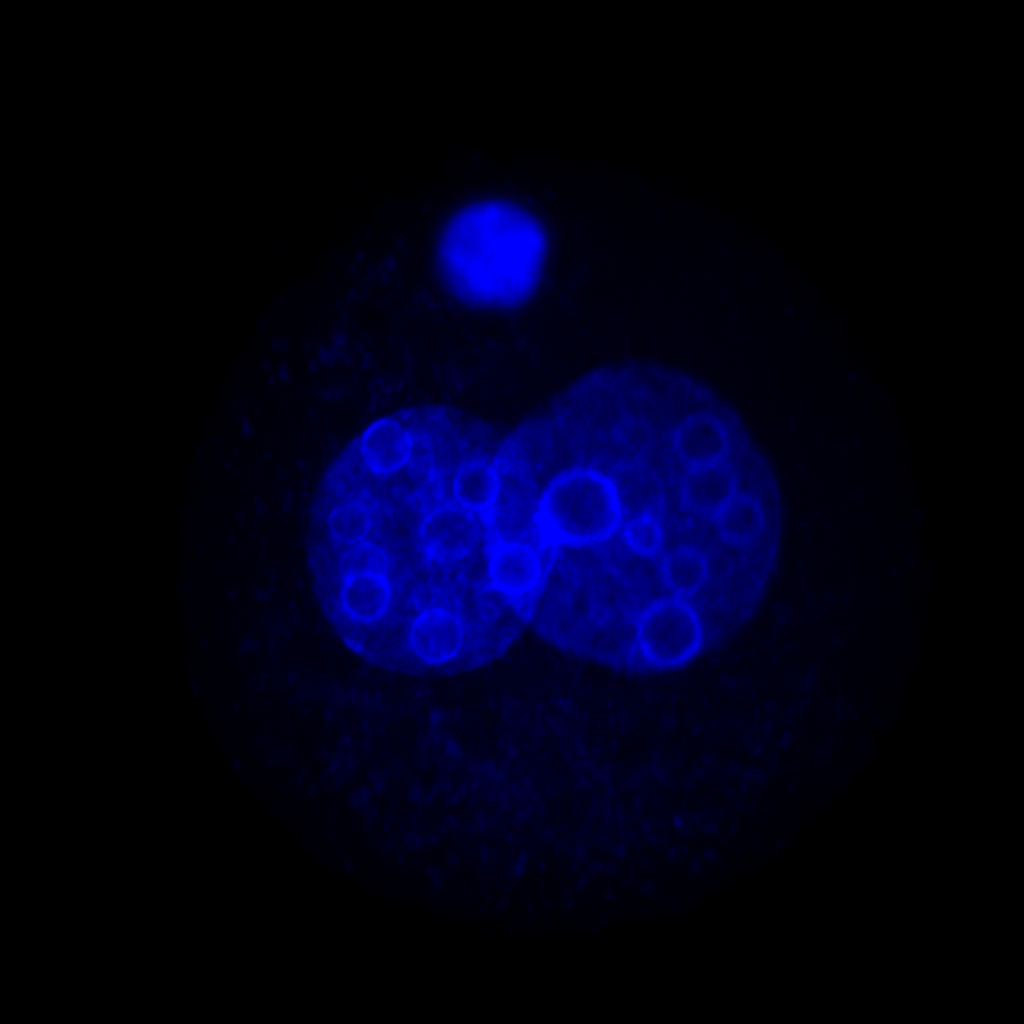

Supplement: Supplementary file 19 — Appendix Source Data [file 44319_2026_712_MOESM19_ESM.zip › Appendix source data/Appendix Figure S2/S2D/Ctrl_Post-S_hCG27h/Ctrl_hCG27h_DAPI.tif]

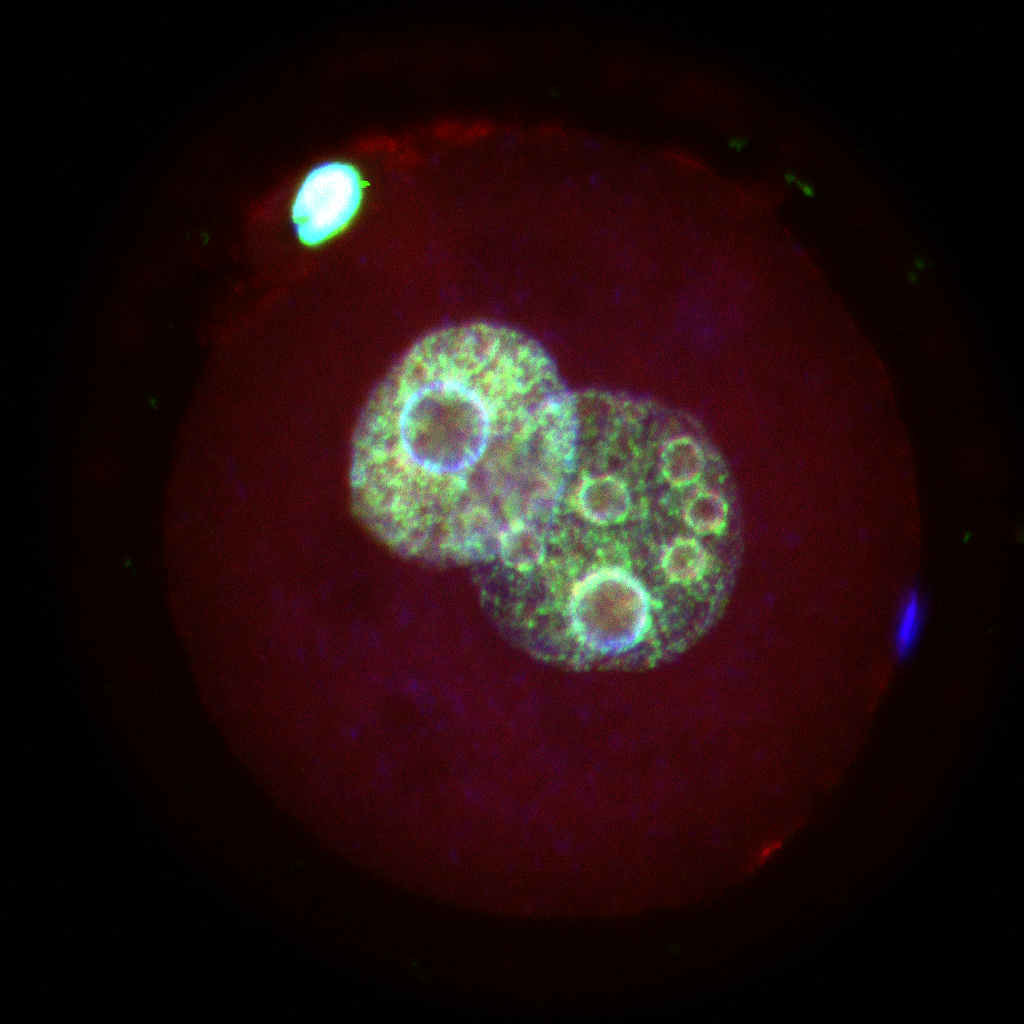

Supplement: Supplementary file 19 — Appendix Source Data [file 44319_2026_712_MOESM19_ESM.zip › Appendix source data/Appendix Figure S2/S2D/mKO_Late-S_hCG27h/mKO.hCG27h.Merge.tif]

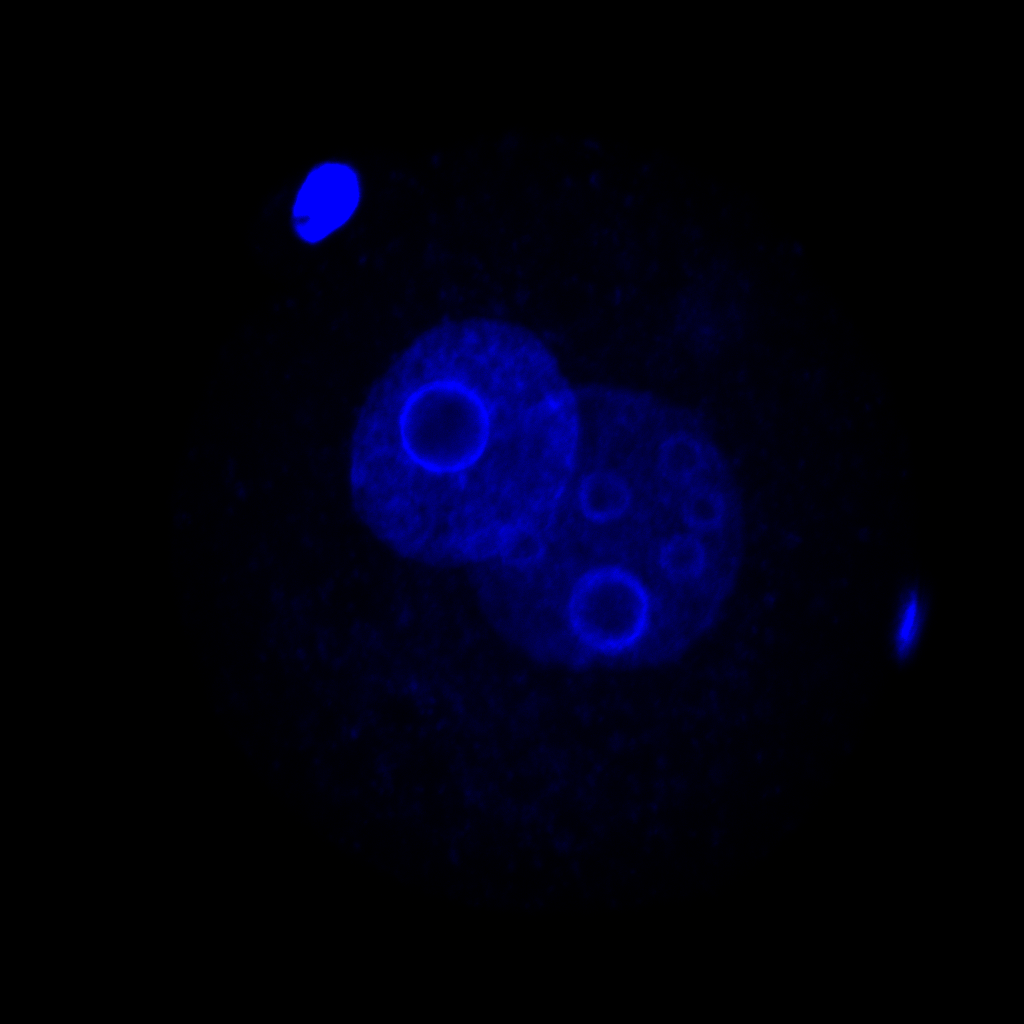

Supplement: Supplementary file 19 — Appendix Source Data [file 44319_2026_712_MOESM19_ESM.zip › Appendix source data/Appendix Figure S2/S2D/mKO_Late-S_hCG27h/mKO.hCG27h.DAPI.tif]

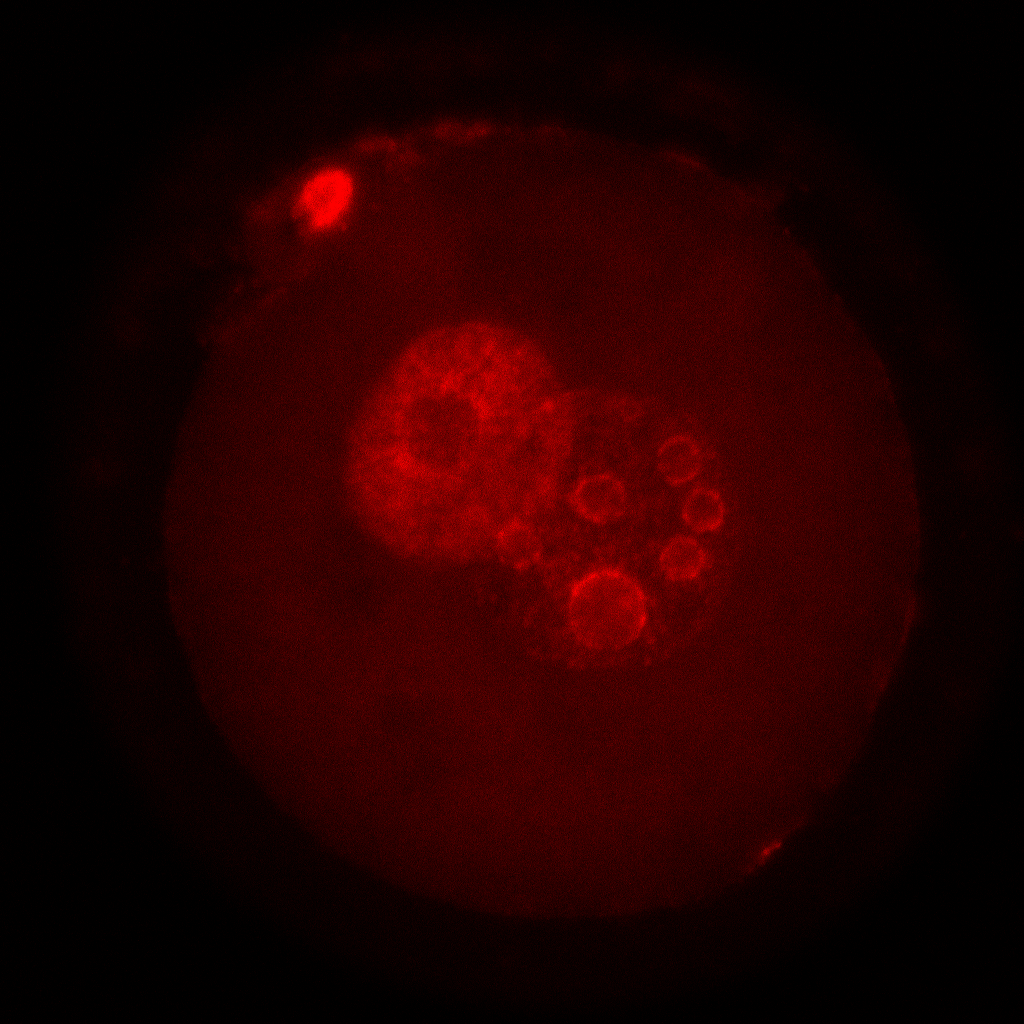

Supplement: Supplementary file 19 — Appendix Source Data [file 44319_2026_712_MOESM19_ESM.zip › Appendix source data/Appendix Figure S2/S2D/mKO_Late-S_hCG27h/mKO.hCG27h.EdU.tif]

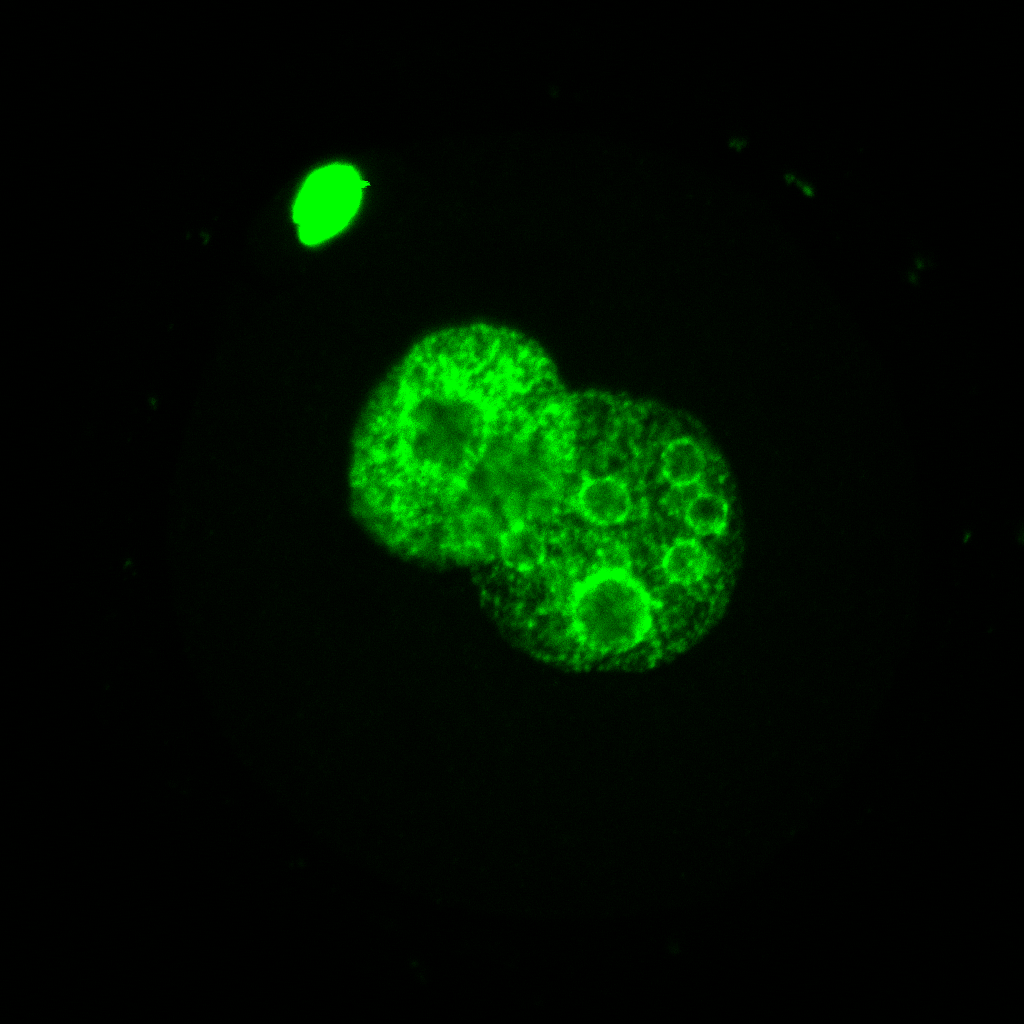

Supplement: Supplementary file 19 — Appendix Source Data [file 44319_2026_712_MOESM19_ESM.zip › Appendix source data/Appendix Figure S2/S2D/mKO_Late-S_hCG27h/mKO.hCG27h.╬│H2AX.tif]

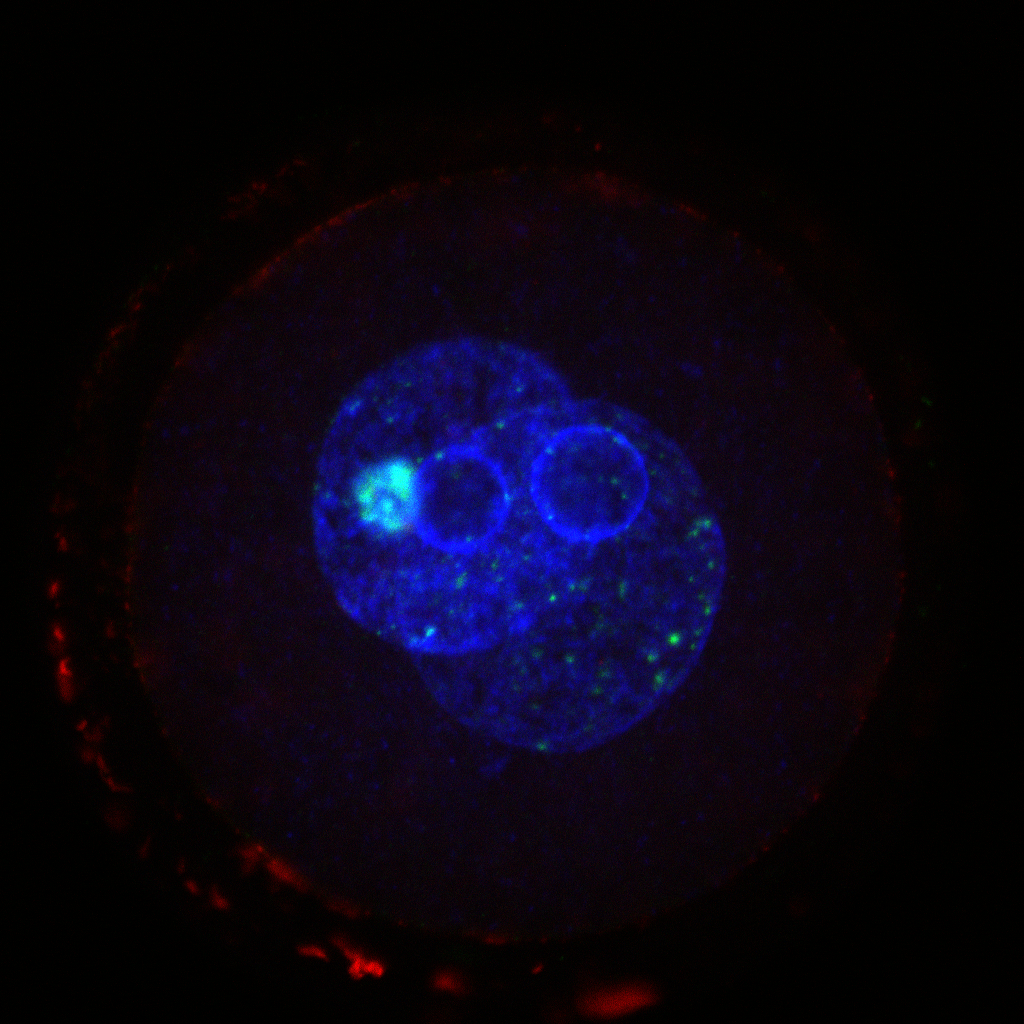

Supplement: Supplementary file 19 — Appendix Source Data [file 44319_2026_712_MOESM19_ESM.zip › Appendix source data/Appendix Figure S2/S2D/mKO_Post-S_hCG27h/mKO.hCG27h.Merge.tif]

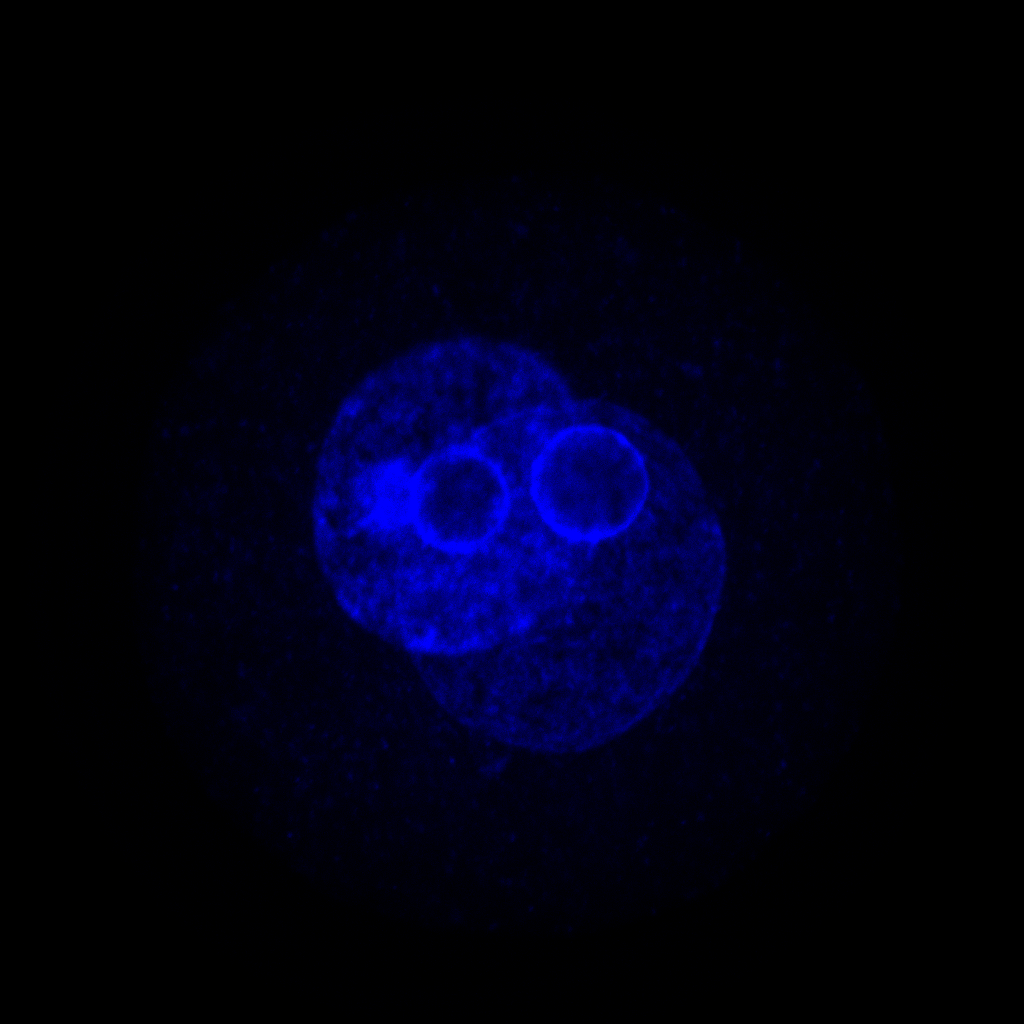

Supplement: Supplementary file 19 — Appendix Source Data [file 44319_2026_712_MOESM19_ESM.zip › Appendix source data/Appendix Figure S2/S2D/mKO_Post-S_hCG27h/mKO.hCG27h.DAPI.tif]

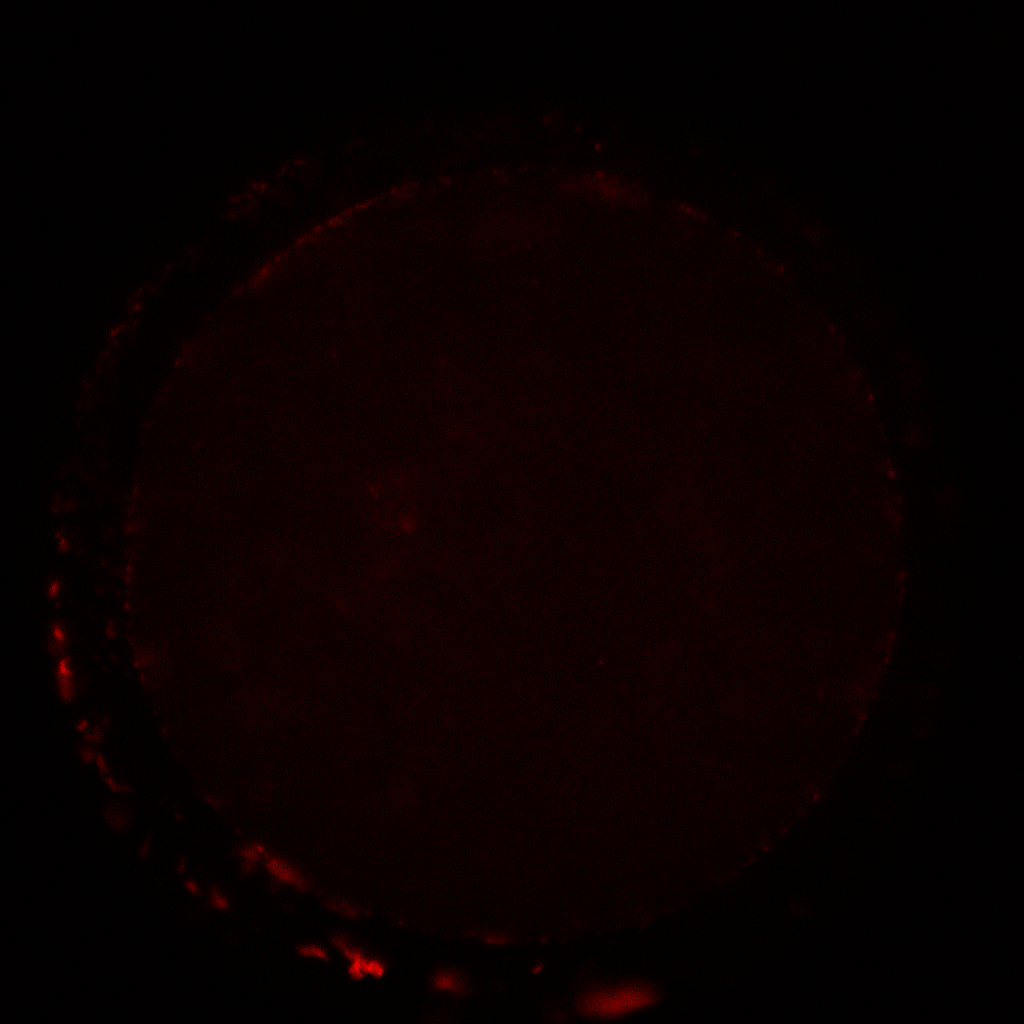

Supplement: Supplementary file 19 — Appendix Source Data [file 44319_2026_712_MOESM19_ESM.zip › Appendix source data/Appendix Figure S2/S2D/mKO_Post-S_hCG27h/mKO.hCG27h.EdU.tif]

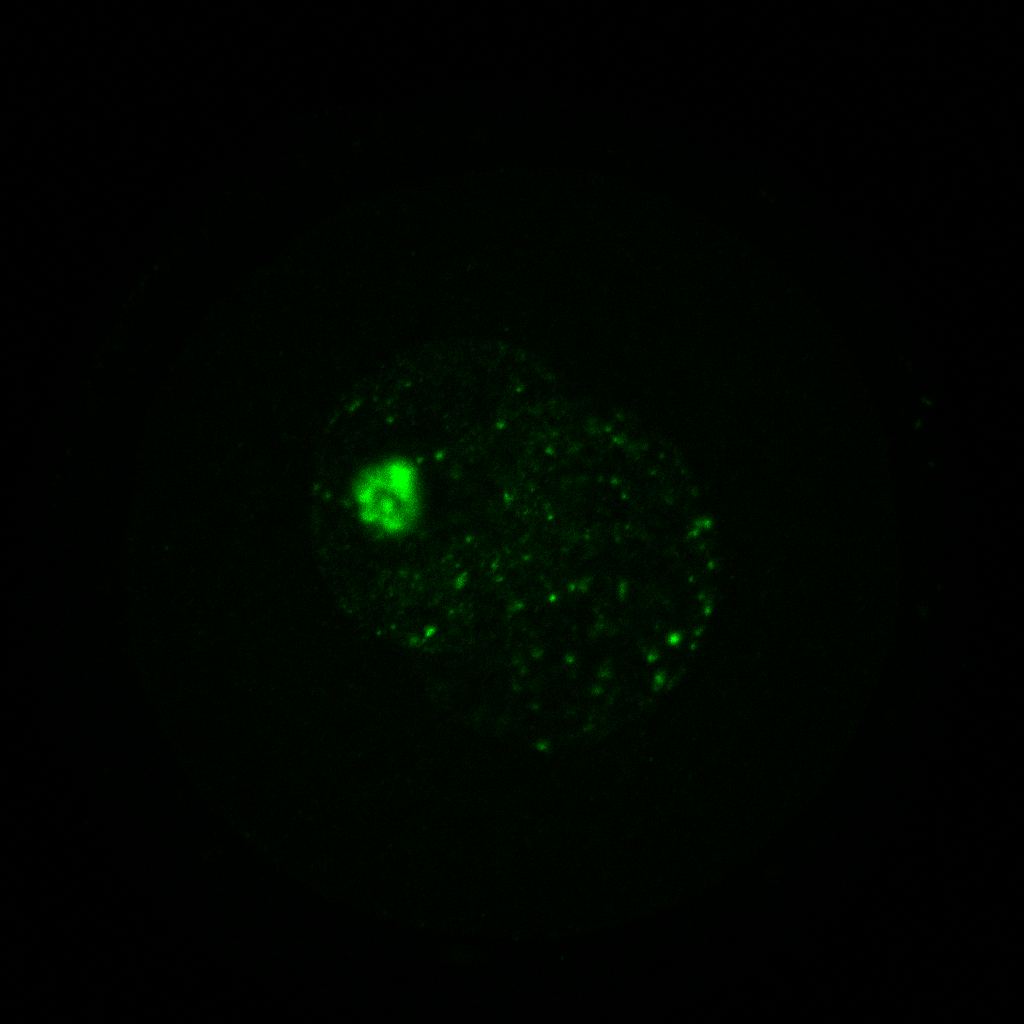

Supplement: Supplementary file 19 — Appendix Source Data [file 44319_2026_712_MOESM19_ESM.zip › Appendix source data/Appendix Figure S2/S2D/mKO_Post-S_hCG27h/mKO.hCG27h.╬│H2AX.tif]
